# Reactivity of a phosphaalumene towards heteroallenes

**DOI:** 10.1039/d6sc04181g

**Published:** 2026-08-31

**Authors:** Tim Wellnitz, Jakob Boardman, Paul Fritsche, Yannick Konrad, Diana Bröllos, Samuel Nees, Holger Braunschweig, Christian Hering-Junghans

**Affiliations:** a Institute for Inorganic Chemistry, Julius-Maximilians-Universität Würzburg Am Hubland 97074 Würzburg Germany h.braunschweig@uni-wuerzburg.de; b Institute for Sustainable Chemistry & Catalysis with Boron, Julius-Maximilians-Universität Würzburg Am Hubland 97074 Würzburg Germany; c Leibniz Institute for Catalysis e.V. (LIKAT) A.-Einstein-Str. 29a 18059 Rostock Germany christian.hering-junghans@catalysis

## Abstract

Exposure of ^Dip^TerPAlCp* (1) to CO_2_ at ambient temperature results in the formation of ^Dip^TerPCO (2) along with the formation of (Cp*AlO)_*x*_. Isolating 2 in crystalline form is challenging, often resulting in mixtures with its dimer (^Dip^TerPCO)_2_ (2dim), which was crystallographically characterized. A [2 + 2] cyclization product of 1 and 2 was crystallographically identified as the cyclic diphosphaurea derivative [(^Dip^TerP)_2_(µ-AlCp*)(µ-CO)] (3), which shows a planar P_2_AlC ring. 2 also reacts with an NHC-stabilized boraketene *via* CO-elimination, giving the oxaborirane 4, featuring a planar BOCP unit with a localized P=C double bond. Additionally, the reactivity of 1 towards diisopropylcarbodiimide (DIC) was explored. Treatment of 1 with DIC resulted in the formation of ^Dip^TerP[C(NiPr)_2_]Al[C(NiPr)_2_]Cp* (5), featuring a PCNAl four-membered ring with an additional Cp*-substituted amidinate ligand on the Al atom. This amidinate substituent results from insertion of a second DIC molecule into the Al–Cp* bond. Heating a solution of 5 results in a thermodynamically driven rearrangement to aluminium phosphaguanidinate complex 6 with a P=C double bond. Compound 6 is best classified as an phosphaguanidinate or as an inversely polarized phosphaalkene, which is supported by NMR data and DFT calculations. Upon hydrolysis 5 and 6 decompose cleanly to give phosphaguanidine ^Dip^TerP(H)C(NHiPr)NiPr (7) with the concomitant formation of a trimeric aluminoxan. This study demonstrates the potential of phosphaalumene 1 as a synthon for aryl phosphaketenes, which themselves enable the synthesis of novel heterocyclic structures. Moreover, the reactivity towards DIC differs considerably from that of related phosphagallenes, enabled by the lability of the Al–Cp* bond.

## Abstract 2

The reactivity of a phosphaalumene towards CO_2_ and carbodiimides is described. While a phosphaketene, which engages in cyclization reactions, is formed with CO_2_, phosphaguanidate complexes are afforded with carbodiimides.

## Graphical abstract

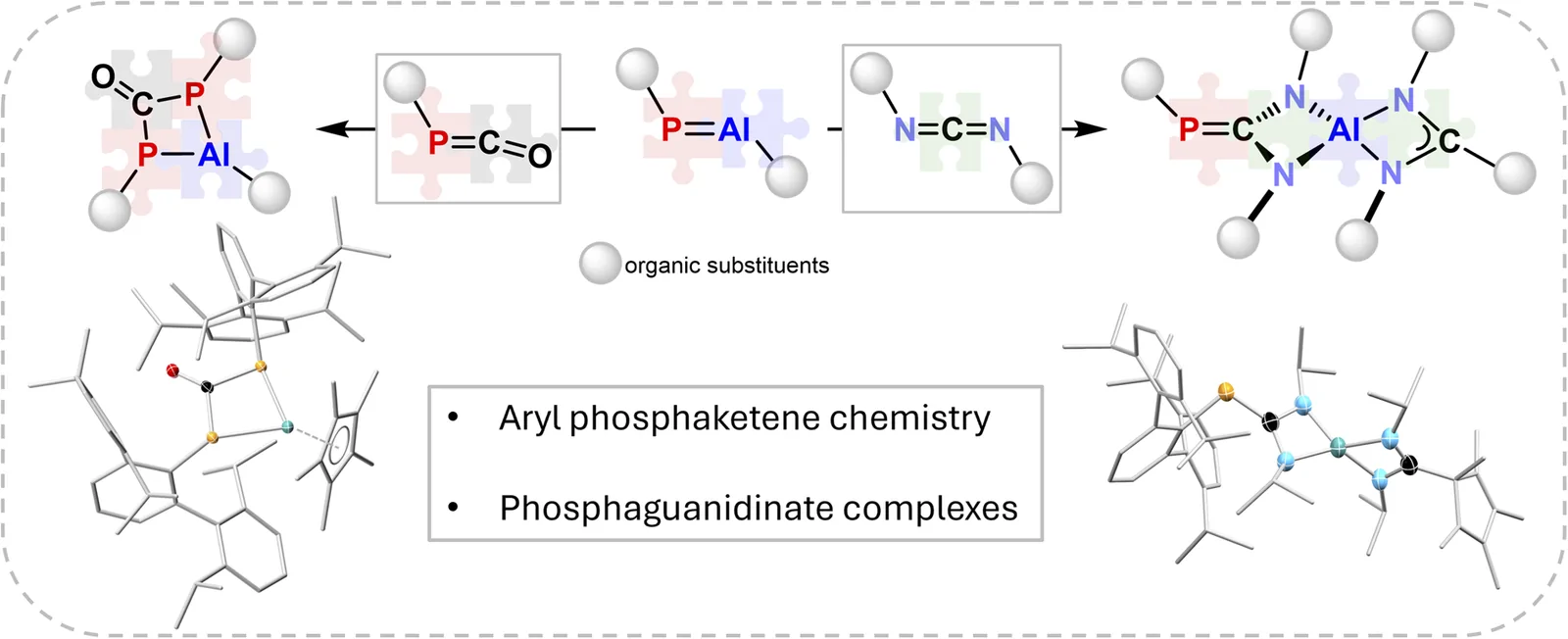

## Introduction

2-Phosphaethynolate salts have emerged as versatile reagents in main group chemistry^1^ for the introduction of the phosphaketenyl group, generating phosphaketenes of the general form R–PCO. An intriguing aspect of phosphaketenes is their propensity to readily decarbonylate, giving access to the corresponding phosphinidenes R–P. Generally, phosphaketenes are obtained through salt metathesis of [Na(PCO)] with element halide precursors. Bertrand and co-workers showed that the sterically overcrowded [^s^P*]PCO ([^s^P*] = (H_2_CNAr*)_2_P; Ar* = 2,6-bis[(4-*tert*-butylphenyl)methyl]-4-methylphenyl) readily releases CO upon UV-irradiation, giving access to the stable resonance-stabilized phosphino-phosphinidene [^s^P*]–P (A, Scheme 1, middle).^2^ Using smaller [^s^P]PCO ([^s^P] = (H_2_CNDip)_2_P, Dip = 2,6-iPr_2_C_6_H_3_) derivatives, facile [^s^P]P transfer has been reported.^3^ This renders phosphanyl phosphaketenes ideal precursors to access element–element multiply bonded species, in particular so-called pnictatrielenes RE^13^=E^15^R′. Goicoechea and co-workers used [^s^P]PCO in combination with ^Dip^NacnacGa (^Dip^Nacnac = HC[C(Me)NDip]_2_) to access phosphanyl phosphagallenes,^4^ while with ^Dip^NacnacAl a labile phosphaalumene was formed (B, Scheme 1).^5^ By a similar approach the group of Schulz reported gallium-substituted phosphagallenes, obtained in the reaction of (^Dip^Nacnac)Ga(PCO)Cl with ^Dip^NacnacGa.^6,7^

**Scheme 1:**
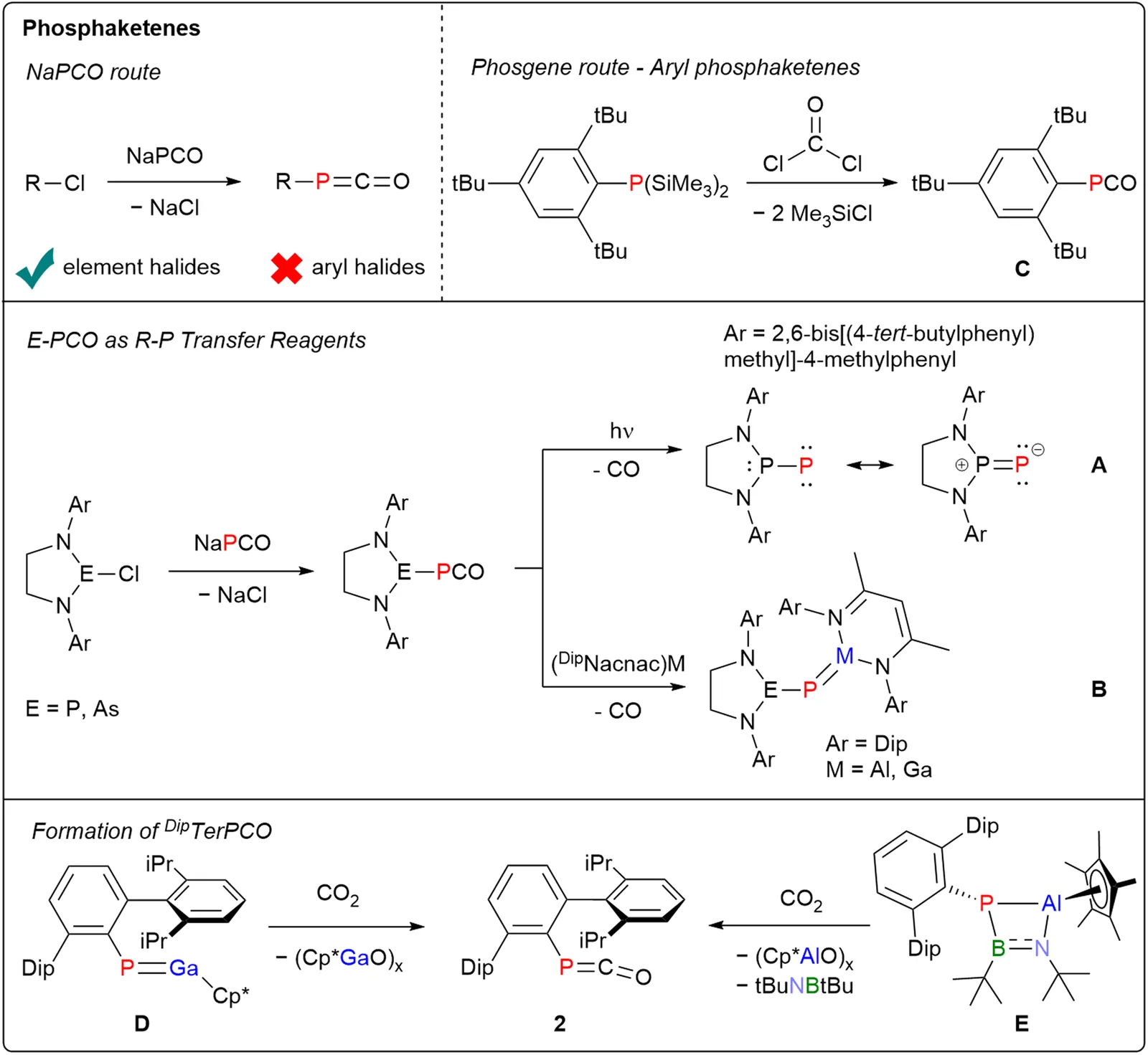
(Top) Synthesis of phosphaketenes and phosphinidene transfer using E-PCO species. (Bottom) Reactivity of a phosphagallene and a BNAlP ring system towards CO_2_.

Despite the abundance of element-substituted phosphaketenes, rather simple arylphosphaketenes are difficult to access. Salt metathesis of [Na(PCO)] with aryl halides has so far not been described and the only arylphosphaketene with synthetic relevance, Mes*PCO (C) (Scheme 1, top; Mes* = 2,4,6-*t*Bu_3_-C_6_H_2_),^8^ was prepared from Mes*P(SiMe_3_)_2_ and phosgene. We have previously shown that ^Dip^TerPGaCp* (D, Scheme 1, bottom) reacted with CO_2_ under elimination of (Cp*GaO)_*x*_ giving ^Dip^TerPCO (2) with a characteristic ^31^P NMR signal at −201 ppm.^9^ Likewise, when BNAlP-heterocycle E was reacted with CO_2_, ^Dip^TerPCO was afforded as the main product. This indicates a cycloreversion and facile reaction of ^Dip^TerPAlCp* with CO_2_ (Scheme 1, bottom).^10,11^

The practicality of these approaches is hampered by the metastable nature of the phosphagallene precursor and the rather tedious synthesis of the BNAlP heterocycle. The transient phosphaalumene [^s^P]–P=Al[^Dip^Nacnac] reacted with CO_2_ in an FLP-type manner, giving a five-membered P_2_AlCO species (F, Scheme 2),^5^ which mimics the reactivity of the related phosphagallene [^s^P]–P=Ga[^Dip^Nacnac].^4^

**Scheme 2:**
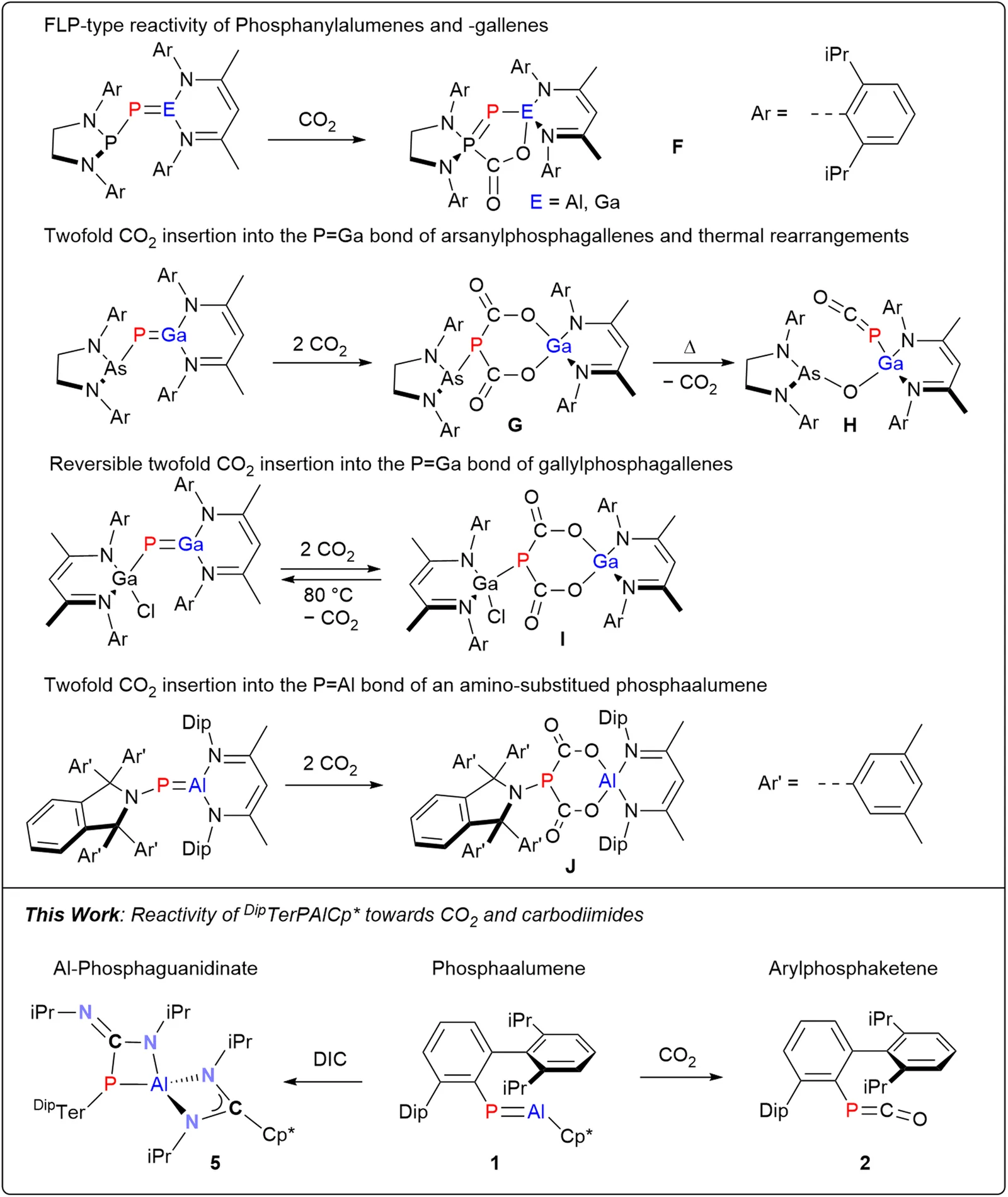
Reactivity of pnictatrielenes towards CO_2_ and summary of this work.

Interestingly, a related arsanyl-phosphagallene showed twofold insertion into the Ga=P bond (G, Scheme 2) and rearranges to give a gallyl-phosphaketene upon heating and concomitant release of one equivalent CO_2_ (H, Scheme 2).^12^ By contrast, Schulz and co-workers noted twofold insertion of CO_2_ into the P=Ga bond in [^Dip^Nacnac]Ga(Cl)–P=Ga[^Dip^Nacnac], giving a six-membered Ga(OC)_2_P heterocycle (I, Scheme 2), which releases CO_2_ reversibly upon heating.^7^ A similar twofold insertion of CO_2_ into the P–Al bond of an amino-substituted phosphaalumene (J, Scheme 2) has been recently reported.^13^

Here, a straightforward route towards ^Dip^TerPCO is outlined and its reactivity is studied, giving access to a unique P_2_AlC-ring species and a phosphaalkene-appended oxaborirane. Moreover, the reactivity of ^Dip^TerPAlCp* towards carbodiimides was evaluated.

## Results & discussion

When an *n*-pentane solution of the purple phosphaalumene ^Dip^TerPAlCp* (1)^11^ is exposed to CO_2_ (1.4 atm) at ambient temperature, an orange solution is afforded, from which minimal amounts of a colorless solid precipitate. After filtration, ^31^P NMR spectroscopy in toluene-d_8_ revealed a new species at −201.3 ppm, while in the ^13^C NMR spectrum a doublet at 198.7 ppm (^1^*J*_C–P_ = 104 Hz) confirmed the formation of ^Dip^TerPCO (2) (Scheme 3). In the ^1^H NMR spectrum a broad feature is detected between 1.5 and 2.2 ppm, indicating a complex mixture of (Cp*AlO)_*x*_ forming as by-products. One sharp peak at 2.0 ppm indicates the presence of (Cp*AlO)_4_, which has previously been described to form when (Cp*Al)_4_ is exposed to O_2_.^14^ The isolation of 2 in crystalline form is difficult and various crystallization attempts always yielded mixtures of 2 and its dimer. This dimer, (^Dip^TerPCO)_2_ (2dim) could be identified by picking some crystals suitable for single crystal X-ray diffraction (scXRD) experiments. 2dim crystallizes in the triclinic space group *P*1̄ or as an *n*-hexane solvate in the monoclinic space group *P*2_1_/*c* (2dim_b, *cf.* Fig. S1 and S2), as a centrosymmetric dimer showing the expected planar P_2_C_2_ ring, with trigonal pyramidal P atoms and P–C_CO_ single bonds (*P*1̄: 1.849(3), 1.851(3); *P*2_1_/*c*: 1.857(3), 1.860(3) Å; *cf.* Σ*r*_cov_(P–C) = 1.86 Å),^15^ while the C–O distance (*P*1̄: 1.210(3); *P*2_1_/*c*: 1.201(3) Å) is in line with the formulation as a 1,3-diphosphetane-2,4-dione (*cf.* (TritPCO)_2_*d*(P–C) = 1.858(3), *d*(C–O) = 1.205(3) Å; Trit = Ph_3_C).^16^ When immediately evaporating an *n*-hexane or *n*-pentane solution of 2, an orange solid is obtained. The IR spectrum of this solid is in line with a terminal PCO unit (*ν̄* = 1944 cm^−1^) (Fig. S16), and HRMS data of 2 suggest facile decarbonylation in the gas phase (Fig. S17). It needs to be noted that when the reaction of 1 with CO_2_ is carried out at lower CO_2_ concentrations a species with a ^31^P NMR signal at *ca.* −30 ppm is formed, which was identified as the [2 + 2] cyclization product of 1 and 2. Full conversion of this competing product to 2 can be achieved after applying CO_2_ pressure again. Considering the quantitative formation of 2 in aliphatic solvents (best monitored by ^31^P NMR spectroscopy), we carried out reactivity studies by generating 2*in situ*, assuming quantitative conversion.

**Scheme 3:**
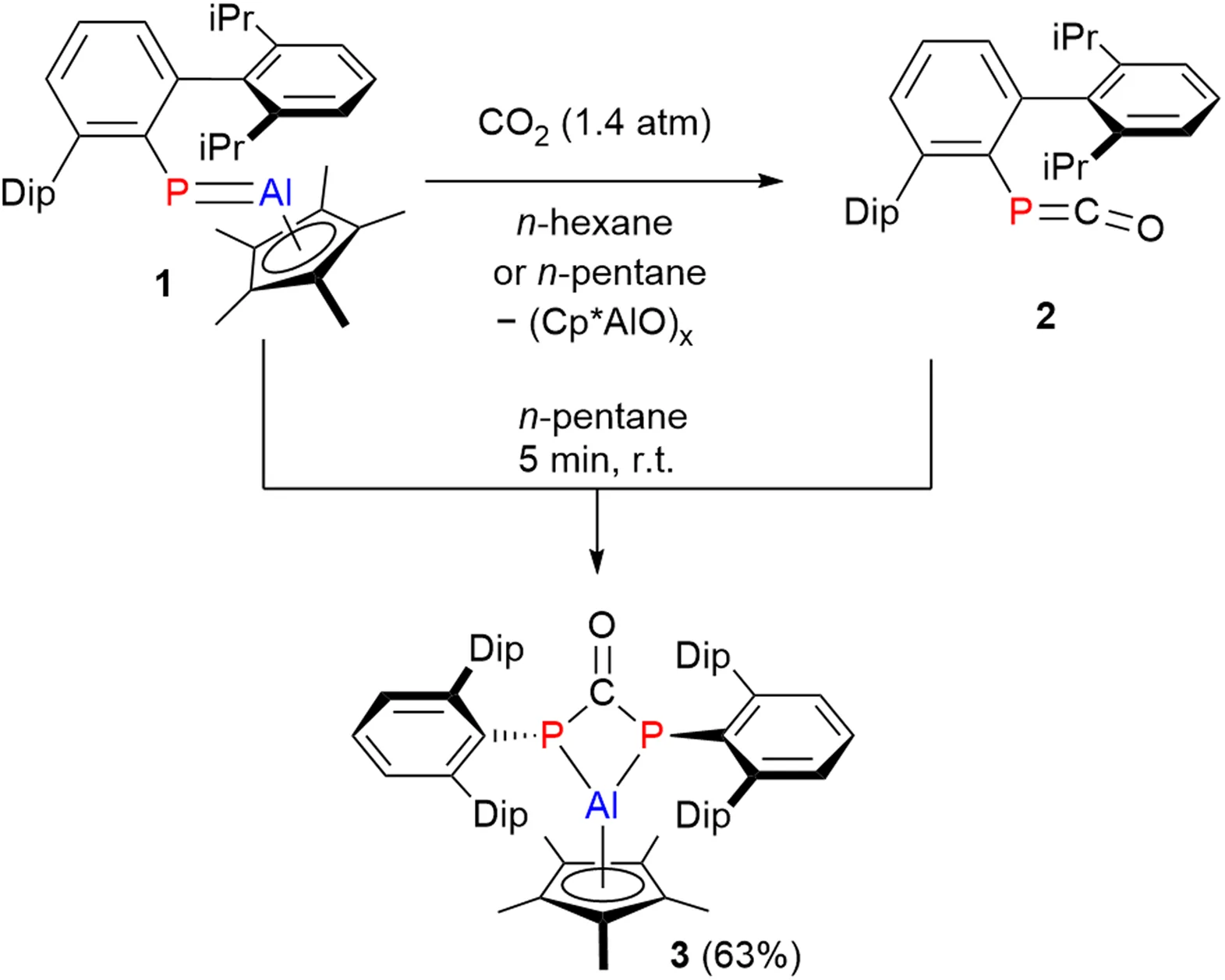
Synthesis of phosphaketene 2 and reactivity of 2 towards 1.

Even though 2 is thermally stable in aliphatic or aromatic solvents, solutions should be handled in the absence of light, as CO extrusion occurs when exposed to ambient light, resulting in the formation of a known phosphafluorene as the deactivation product of the phosphinidene ^Dip^Ter–P (*cf.* Fig. S15).^9,17^ It needs to be noted that (Cp*AlO)_*x*_ does not interfere with the reactivity of 2.

When an *n*-pentane solution of 2 was treated with one equivalent of 1 a colour change to yellow was noted and quantitative conversion towards a new species with a ^31^P NMR signal at −27.0 ppm was detected. The same ^31^P NMR shift was observed for the competing product in the synthesis of 2 at lower CO_2_ pressure. Crystals suitable for scXRD revealed the formation of [(^Dip^TerP)_2_(µ-AlCp*)(µ-CO)] (3), which is best described as a cyclic diphosphaurea derivative with a bridging Al(iii) center or as an Al(iii) diphosphaureylene complex. The formation of 3 is thermodynamically driven and exergonic (Δ_R_*G*° = −57.3 kJ mol^−1^) with an activation barrier of 43.3 kJ mol^−1^, in line with a facile transformation at ambient temperature. The transition state structure can be interpreted as due to nucleophilic attack of the P atom in ^Dip^TerPAlCp* at the C atom of the PCO-unit in 2 (*cf.* Fig. S60).

Mononuclear diphosphaureylene complexes of Pt and Pd have been reported and were formed in the reaction of Mes*PCO with zero-valent Pt and Pd precursors upon decarbonylation.^18,19^3 crystallizes as a mixed benzene/*n*-pentane solvate in the monoclinic space group *C*2/*c* with eight formula units in the unit cell (Fig. 1). The central ^Dip^TerP_2_(µ-AlCp*)(µ-CO) fragment shows a planar (P3–C1–P2–Al1 = 0.03(8) °) kite-shaped P_2_AlC four-membered ring with nearly equidistant P–Al (2.3600(7), 2.3693(7) Å; *cf.* Σ*r*_cov_(P–Al) = 2.37 Å),^15^ and minimally different P–C distances (P2–C1 1.820(2), P3–C1 1.843(2) Å; *cf.* Σ*r*_cov_(P–C) = 1.86 Å)^15^ in the range of typical single bonds and elongated compared to a related diphosphaureylene palladium complex (*cf.* Pd(dppe)[Mes*PC(O)PMes*],^19^ dppe = (Ph_2_PCH_2_)_2_*d*(P–C) = 1.805(12), 1.794(12) Å). The average Al–C_Cp*_ distances (*ca.* 2.19 Å) are reflective of an η^5^-coordinated Cp* group, while the C–O distance (1.223(2) Å; *cf.* Σ*r*_cov_(C=O) = 1.24 Å)^15^ is now longer compared to 2 (*cf. d*(C–O) = 1.156(1) Å)^9^ and in the range of a double bond (*cf.*I, Scheme 2; *d*(C–O) = 1.234(8) Å).^7^

**Fig. 1:**
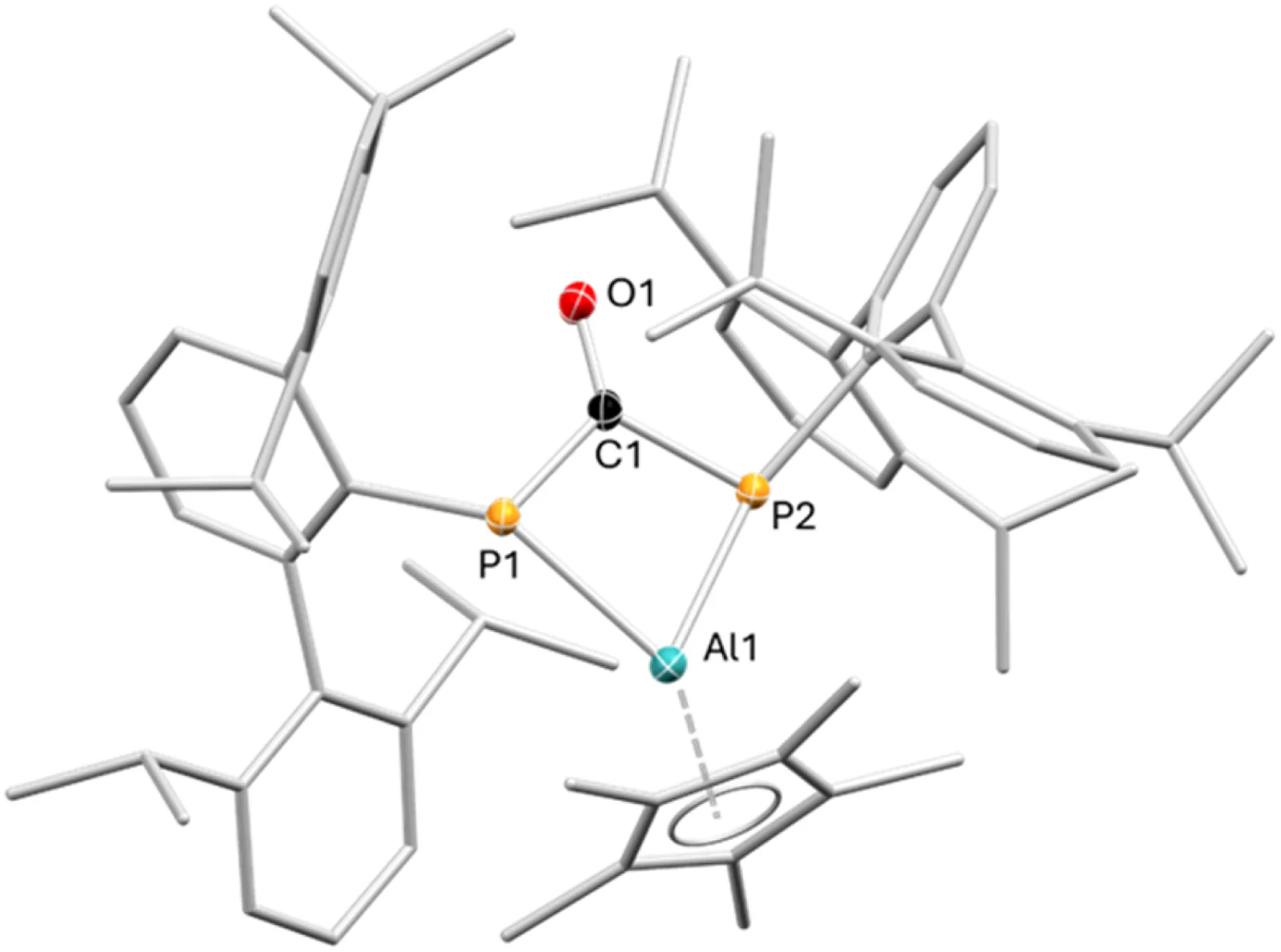
Molecular structure of 3. Ellipsoids drawn at 50% probability with C–H-atoms and solvent molecules omitted. Substituents rendered as a wire-frame for clarity. Selected bond lengths [Å] and angles [°]: P1–C1 1.820(2), P1–C2 1.843(2), Al1–P1 2.3693(7), Al1–P2 2.3600(7), O1–C1 1.223(2), P1–P2 2.9780(6), C1–Al1 2.904(2); P1–C1–O1 126.5(1), P2–C1–O1 124.7(1), P1–C1–P2 108.8(1), P1–Al1–P2 78.06(2), C1–P1–Al1 86.71(6), C1–P2–Al1 86.46(6); P1–C1–P2–Al1 0.03(8), O1–C1–P1–Al1 179.6(2), O1–C1–P2–Al1 179.6(2).

Both P atoms are in flattened trigonal pyramidal coordination environments (*Σ*(<P1) = 337.6(2), *Σ*(<P2) = 327.0(2) °) indicating P(iii) atoms. Moreover, the keto-function is detected in the IR spectrum at 1550 cm^−1^, in line with previous reports on diphosphaurea derivatives.^18,19^ In solution compound 3 shows *C*_1_ symmetry with four signals for the methine protons and overall eight doublets for the Me-groups of the Dip–iPr groups. Isolated 3 was re-dissolved in benzene-d_6_ and was exposed to CO_2_, resulting in a color change from yellow to orange and clean conversion into 2 according to ^31^P NMR spectroscopy (*cf.* Fig. S52), underlining its role in the formation of 2. 3 is thermally labile when heated to 80 °C over a period of 16 h in benzene-d_6_ solution showing the presence of ^Dip^TerPCO (2) and a so far unknown major product (*cf.* Fig. S23).

Next, we elucidated the potential of 2 to react with an NHC-stabilized boraketene,^20^ potentially opening the path to an NHC-coordinated phosphaborene through phosphinidene borylene recombination after twofold CO extrusion. Such species are usually generated by NHC-induced Me_3_SiCl elimination from the respective RP(SiMe_3_)B(Cl)R′ precursors.^21,22^ When 2 was combined with (IPr)B(CO)Cy (IPr = (HCNDip)_2_C; Cy = cyclohexyl) in C_6_D_6_, ^31^P NMR spectroscopy indicated the formation of two new species at 102.3 and 20.6 ppm (Fig. S30), of which the signal at 102.3 ppm vanished after further heating to 80 °C for 60 h. The intermediate is proposed to be a [2 + 2]-cyclization product of 2 with the BCO unit in (IPr)B(CO)Cy without CO release.

After work-up and re-crystallization from *n*-hexane compound 4 was afforded as a colourless crystalline solid and its molecular structure was determined by scXRD experiments (Scheme 4). The ^31^P NMR signal of 4 in toluene-d_8_ is detected at 18.9 ppm. NMR experiments were performed at −60 °C due to rotational hindrance and show overall *C*_1_ symmetry in solution as indicated by inequivalence of all iPr-groups of the Dip-substituents in the ^Dip^Ter- and IPr-moiety. In the ^11^B NMR spectrum a broad resonance is detected at −12.0 ppm. 4 is best described as an oxaborirane, of which only one derivative is known to date, afforded by CO insertion into an unsymmetrical diborane (*cf. δ*(^11^B) = −14.7 ppm).^23^ Interestingly, the three-membered BCO ring in 4 is part of a phosphaalkene (PA) unit. 4 crystallizes in the triclinic space group *P*1̄ with two molecules in the unit cell (Fig. 2). 4 has a localized P=C double bond (1.679(2) Å; *cf.* Σ*r*_cov_(P=C) = 1.69 Å)^15^ with a C1–P1–C^Ar^ angle of 107.82(8) °. The central BCO three-membered ring is asymmetric with shortened C–B (1.547(2) Å) and C–O (1.346(2) Å) single bonds (Σ*r*_cov_(C–B) = 1.60 Å; Σ*r*_cov_(C–O) = 1.38 Å), and an elongated B–O single bond (1.558(2) Å; *cf.* Σ*r*_cov_(B–O) = 1.48 Å).^15^ The bond parameters in the BCO-ring agree with those reported for Kinjo's oxaborirane.^23^ The boron atom is in a distorted tetrahedral coordination environment according to a *τ*_4_-value of 0.84.^24^ C1 is in a trigonal planar coordination environment (*Σ* < (C1) = 359.7(4)°), suggesting an sp^2^ hybridization.

**Scheme 4:**
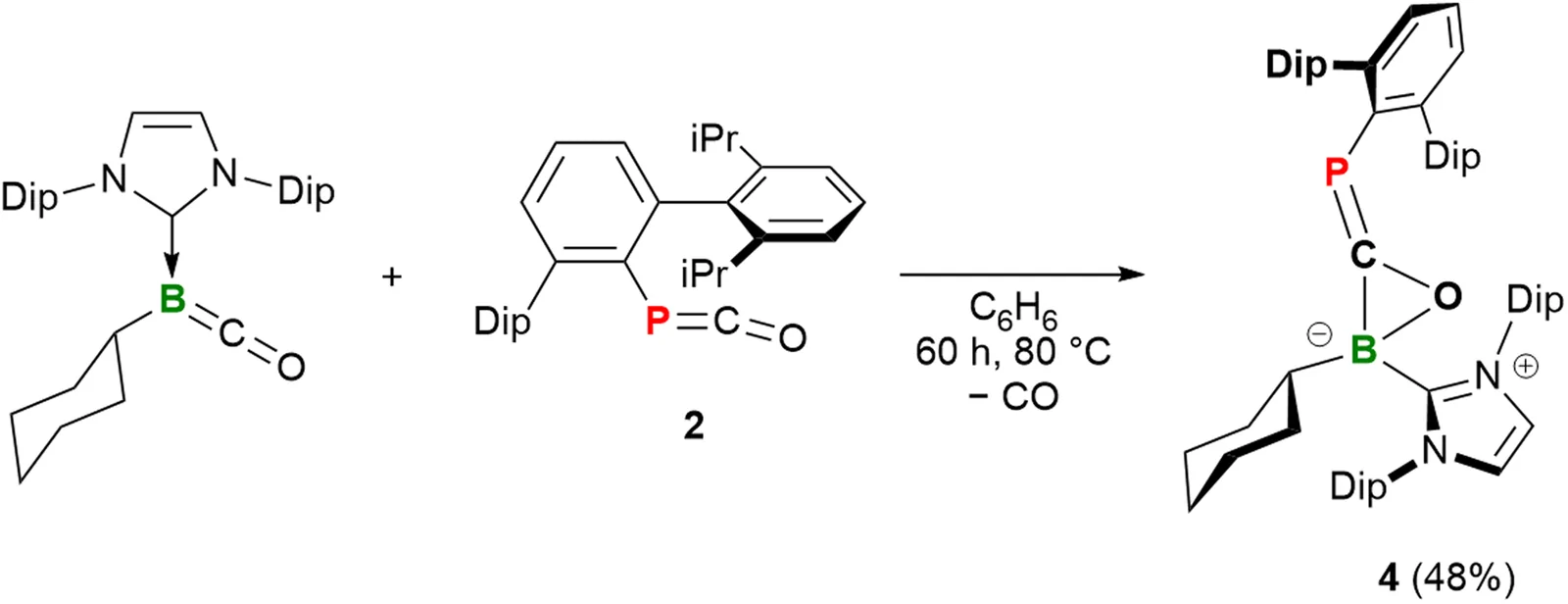
Reactivity of 2 towards boraketene (IPr)B(CO)Cy giving oxaborirane 4.

**Fig. 2:**
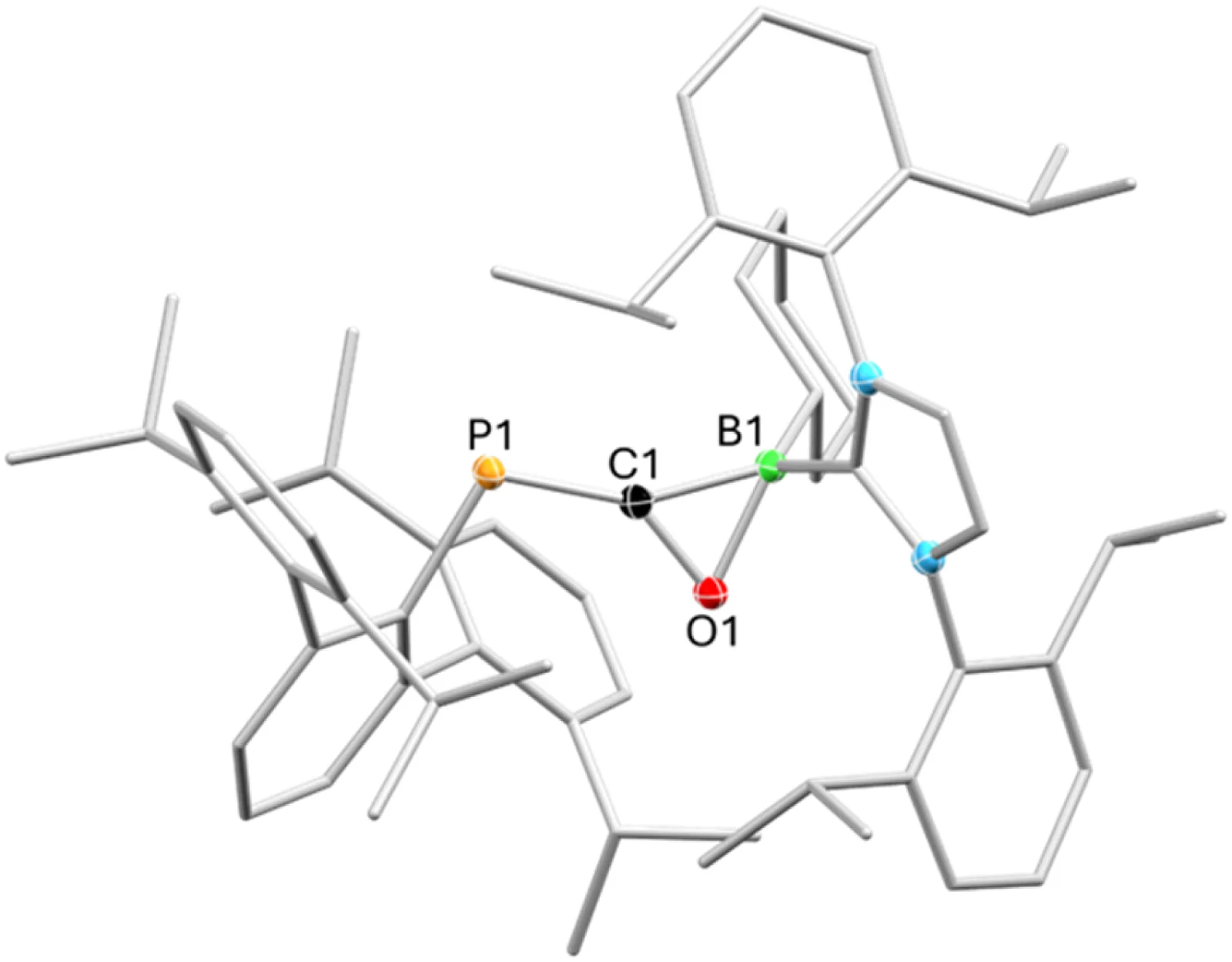
Molecular structure of 4. Ellipsoids drawn at 50% probability with C–H-atoms and solvent molecules omitted. Substituents rendered as a wire-frame for clarity. Selected bond lengths [Å] and angles [°]: P1–C1 1.679(2), O1–C1 1.346(2), O1–B1 1.558(2), C1–B1 1.547(2); C1–O1–B1 63.89(10), O1–C1–B1 64.73(11), O1–C1–P1 140.8(1), B1–C1–P1 154.2(1), C1–B1–O1 51.39(9), B1–O1–C1–P1 174.8(2).

This results in a planar BOCP unit (B1–O1–C1–P1: 174.8(2)°; QMW of the deviation of the atoms from the least-squares plane: 0.0175 Å). To further elucidate the electronic structure the Kohn–Sham orbitals were investigated at the PBE0-D3/def2-TZVP level of theory and reveal a HOMO that mainly represents the P–C π-bond, while the HOMO−1 shows large contributions from an s-type lone pair on the P atom, both in line with the formulation as a phosphaalkene (PA) (Fig. 3, top). Furthermore, NBO analyses, including inspection of the Wiberg Bond Indices (WBI), paint a similar picture.

**Fig. 3:**
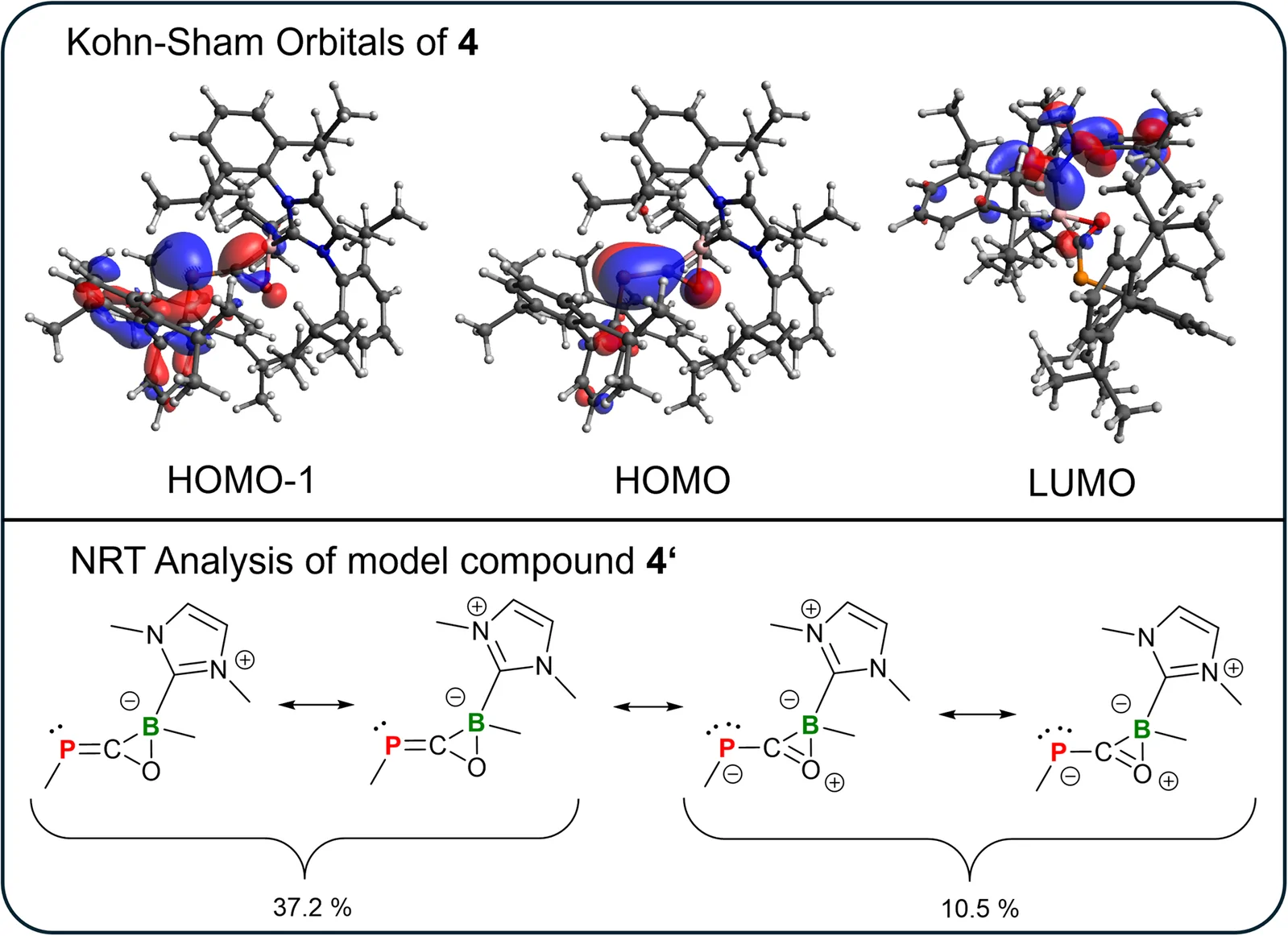
Kohn–Sham orbitals of 4 at the PBE0-D3/def2-TZVP level of theory (top) and the NRT scheme showing the leading resonance structures of model compound 4′ (bottom, only structures with a weight > 2% were considered).

The P–C bond is best characterized as a double bond (WBI = 1.63) with polarized σ- (P: 35, C: 65%) and π-components (P: 58, C: 42%). This contrasts for example pyridyl-phosphaalkenes (*cf.* py-CH=PMes, py = 2-pyridyl; π(P=C): P 41, C 59%), rendering the P atom in 4 rather electron-rich, which is also reflected in the shielded ^31^P NMR resonance at 18.9 ppm (*δ*_calc_(^31^P) = 20.6 ppm), which is in the range of carbene phosphinidene adducts of strongly π-accepting NHCs such as {H_2_C(H_2_CNDip)_2_}C=PPh (*cf. δ*(^31^P) = 14.8 ppm).^25,26^ The natural charges in the BOCP framework indicate strong polarization within the ring (B: 0.45, C: −0.21, O: −0.59*e*) with polarized covalent C–O (WBI: 1.11) and C–B (0.87) single bonds. The B–O bond has a high ionic character as indicated by a rather low WBI (0.67) and strong polarization towards the O atom (B: 21, O: 79%). Using a truncated model (4′, replacing the ^Dip^Ter with Me and IPr with IMe_2,_ IMe_2_ = (HNCMe)_2_C) showed that compound 4 is best described as a phosphaalkene appended to a BCO three-membered ring, while a minor resonance form suggests the description as inversely polarized PA with an additional C=O bond in the ring, through delocalization of one of the LP on the O atom (Fig. 3, bottom). This underlines the potential of 2 not only as a phosphinidene transfer reagent, but as a building block for P-containing heterocycles.

Next the reactivity of phosphaalumene 1 towards another class of heteroallenes, carbodiimides, was evaluated. When 1 was treated with one equivalent of C(NiPr)_2_ (DIC) the characteristic purple colour of 1 faded; however, in the ^31^P NMR spectrum 1 was detected along with a new species with a singlet at −56.1 ppm in a 1 : 1 ratio. This pointed to incorporation of more than one equivalent of DIC and the reaction was repeated with an excess of DIC (Scheme 5), resulting in rapid decolouration at ambient temperature, with a single product in the ^31^P NMR spectrum at −56.1 ppm. At room temperature the signals are significantly broadened, pointing to hindered rotation in solution. In the ^1^H NMR spectrum (at −60 °C in toluene-d_8_) overall seven signals for the methine CH-protons of the iPr-groups pointed to the incorporation of two equivalents of DIC.

**Scheme 5:**
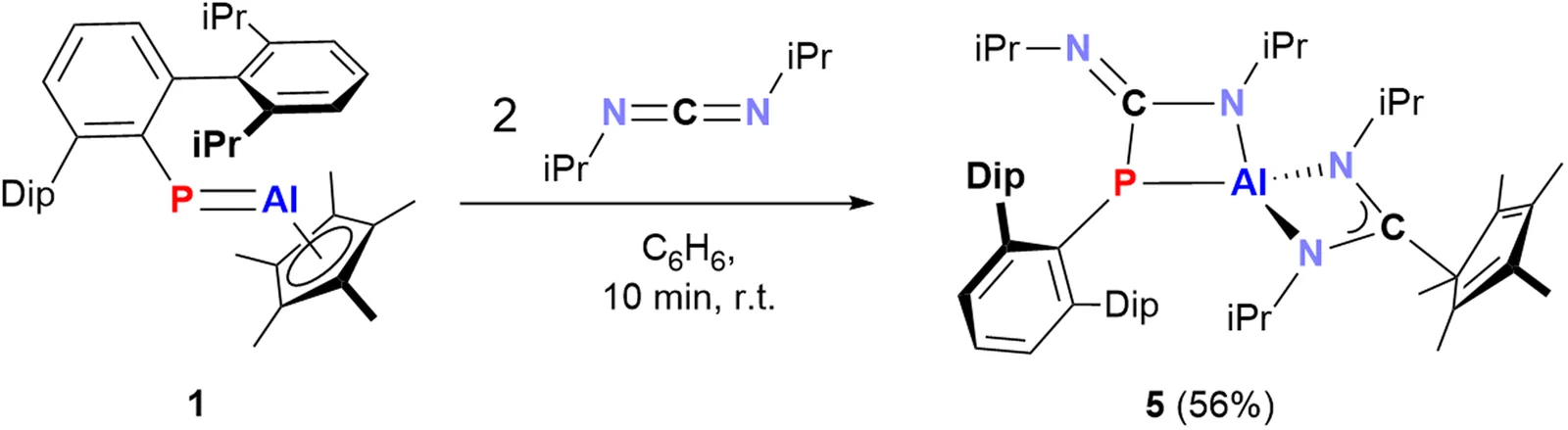
Reactivity of phosphaalumene 1 towards DIC giving the phosphaguanidinate species 5.

Crystals suitable for scXRD experiments were afforded by storing a saturated *n*-pentane solution of the crude product at −30 °C. This revealed the formation of compound ^Dip^TerP[C(NiPr)_2_]Al[C(NiPr)_2_]Cp* (5), which is best described as the [2 + 2]-cycloaddition product of 1 and DIC with formation of a PCNAl-four-membered ring, which can also be described as Al-κ*P*-κ*N*-phosphaguanidinate. Additionally, insertion of a second carbodiimide into the AlCp* bond gives a Cp*-substituted amidinate ligand coordinated to the Al center in 5.^27^

Compound 5 was isolated in 56% yield as a colourless crystalline solid and crystallizes in the monoclinic space group *P*2_1_/*n* with four molecules in the unit cell (Fig. 4). The P atom is in a distorted trigonal pyramidal coordination environment (*Σ*(<P) = 301.9(5)°), while the central Al atom is in a coordination environment that is best described as distorted tetrahedral (*τ*_4_ = 0.63).^24^ The P1–C1 (1.925(1) Å), C1–N2 (1.401(2) Å) and Al1–N2 distances (1.827(1) Å) correspond to single bonds, while the exocyclic C1–N1 distance is rather short (1.277(2) Å) in line with formulation as a double bond. Both C1 and N1 are in distorted trigonal planar coordination environments, which results in a distorted PCNAl ring that features a P1–Al1 single bond (2.3443(5) Å), nearly matching that in the related phosphaalumetidine ^Dip^TerP(H_2_CCHPh)AlCp* (*cf. d*(P–Al) = 2.3992(5) Å).^28^ The newly formed amidinate ligand in 4 shows minimally different C2–N distances (C2–N3 1.333(2), C2–N4 1.361(2) Å) with a C2–C_Cp*_ distance that indicates a single bond and an η^1^-Cp* substituent, which is further supported by ^1^H NMR data. The Al–N3/4 distances (1.900(1), 1.891(1) Å) are rather long and shorter than those reported for the related Al(iii) amidinate complex {*t*BuC(NCy)_2_}AlMe_2_*d*(Al–N) = 1.923(2), 1.926(2) Å.^29^

**Fig. 4:**
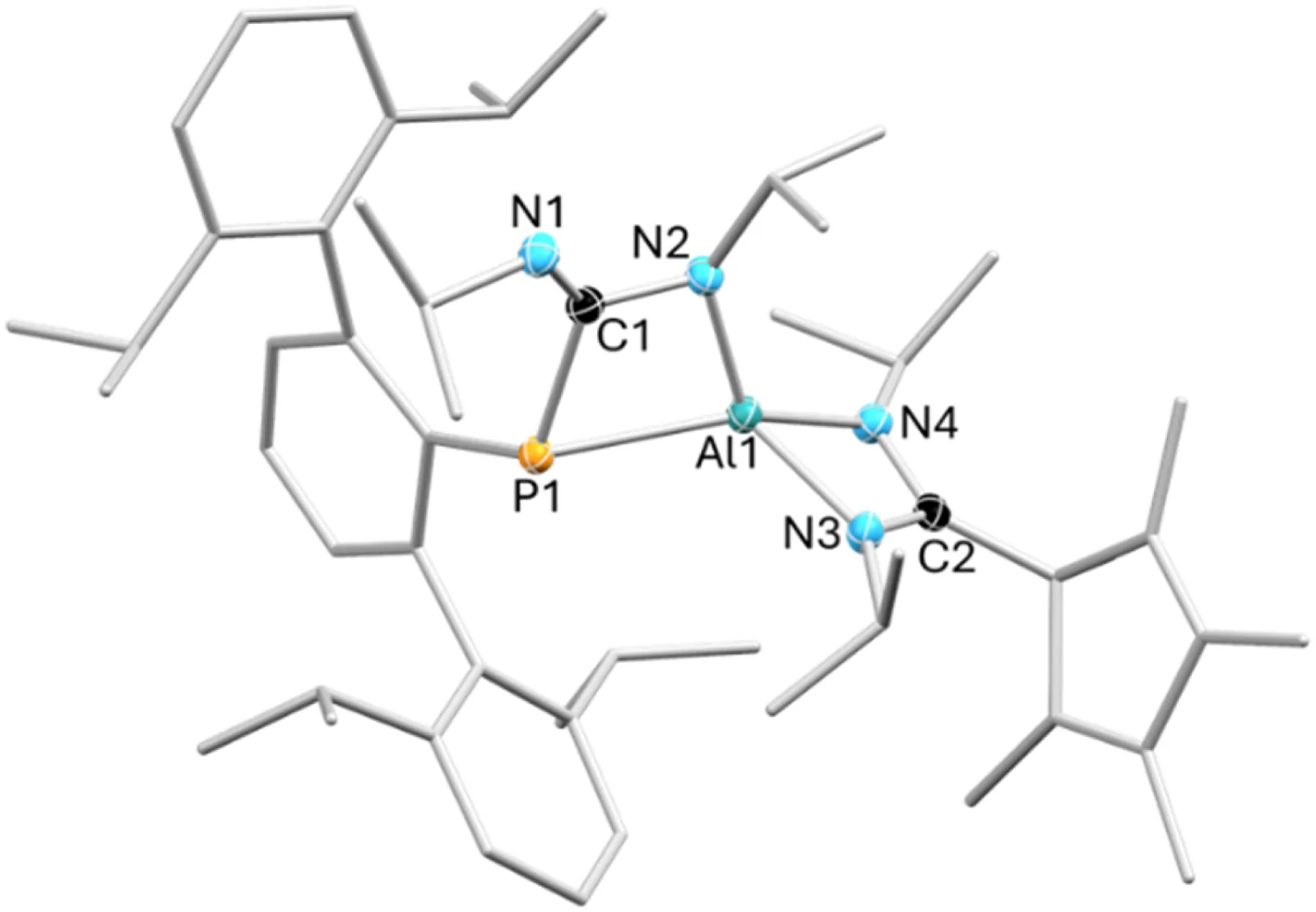
Molecular structure of 5. Ellipsoids drawn at 50% probability with C–H-atoms and solvent molecules omitted. Substituents rendered as a wire-frame for clarity. Selected bond lengths [Å] and angles [°]: P1–C1 1.925(1), P1–Al1 2.3443(5), Al1–N2 1.827(1), Al1–N4 1.891(1), Al1–N3 1.900(1), Al1–C2 2.353(1), Al1–C1 2.516(1), N1–C1 1.277(2), C1–N2 1.401(2), C2–N3 1.333(2), C2–N4 1.361(2); C1–P1–Al1 71.43(4), N2–Al1–N4 124.76(5), N2–Al1–N3 124.15(5), N4–Al1–N3 69.85(5), N2–Al1–P1 79.25(4), N1–C1–N2 121.9(1), N3–C2–N4 107.3(1), P1–C1–N2–Al1 9.03(9), N4–C2–N3–Al1 1.1(1).

When a C_6_D_6_ solution of 5 was heated at 80 °C for 14 d the colour of the solution turned from pale yellow to orange with a new species being detected in the ^31^P NMR spectrum at −14.3 ppm (*cf.* Fig. S38). Four signals were detected for the iPr-methine protons in the ^1^H NMR spectrum with only one set of signals for the ^Dip^Ter-substituent (*cf.* Fig. S36), indicating a *C*_s_ symmetric species with free rotation about the P–C_Ter_ axis in solution. Crystals suitable for scXRD experiments were afforded by slow evaporation of an *n*-hexane/hmdso (hmdso = hexamethyldisiloxane, O(SiMe_3_)_2_) solution of the crude product at ambient temperature and revealed the formation of species 6 in which the four-membered PCNAl-ring in 5 has rearranged to a κ^2^*N*-phosphaguanidinate ligand. The rearrangement from 5 to 6 is exergonic (*cf.* Δ_R_*G*° = −13.9 kJ mol^−1^, for details see Page S81). C(NiPr)_2_ is now symmetrically inserted into the former P–Al bond to give the phosphaguanidinate fragment ^Dip^Ter–P=C(NiPr)_2_ coordinating the Al atom (Scheme 6). The coordination at the four-coordinate Al atom is completed by the (iPrN)_2_CCp* amidinate ligand. Jones, Aldridge and co-workers reported the successive carbodiimide insertion into the Fe–B and B–N bonds of a cationic iron aminoborylene complex, giving a spirocyclic boronium species.^30^ A similar rearrangement was observed in the reaction of the imido complex Cp_2_Ti=NDipp with *p*-tolylcarbodiimide. While first a [2 + 2]-cycloaddition gives a titanocene guanidinato complex, a retro cycloaddition gives a *p*-tolyl imido complex, which reacts immediately with the concomitantly released asymmetric carbodiimide in a [2 + 2] cycloaddition.^31^ We suggest a comparable cycloreversion to give phosphaazaallene 6int (Scheme 6, bottom), based on a minor resonance in the ^31^P NMR spectrum of the reaction mixture at −131.3 ppm (*cf.*^Dip^TerPCN*t*Bu *δ*(^31^P) = −134.8 ppm).^32^ However, a simple P–Al dissociation, C–N bond rotation and Al–N association cannot be ruled out. Stephan and co-workers reported the reaction of the zirconocene phosphinidene complex Cp_2_Zr(PMes*)(PMe_3_) giving the κ^2^N-phosphaguanidinate complex [Cp_2_Zr(CyN)_2_C=PMes*], with the analog of 5, a κP-κN-guanidinate complex, formed intermediately.^33^ The RP=C(NiPr)_2_ unit is also related to phosphaguanidines of the type R_2_P–C(NR)N(H)R, which show a rich coordination chemistry.^34,35^ Interestingly, the reaction of PhP(SiMe_3_)_2_ with carbodiimides was shown to give after intramolecular Me_3_Si-migration phosphaguanidines of the type PhP=C(N(R)SiMe_3_)_2_, which in contrast to 6 showing rather deshielded ^31^P NMR signals at *ca.* 120 ppm.^36^

**Scheme 6:**
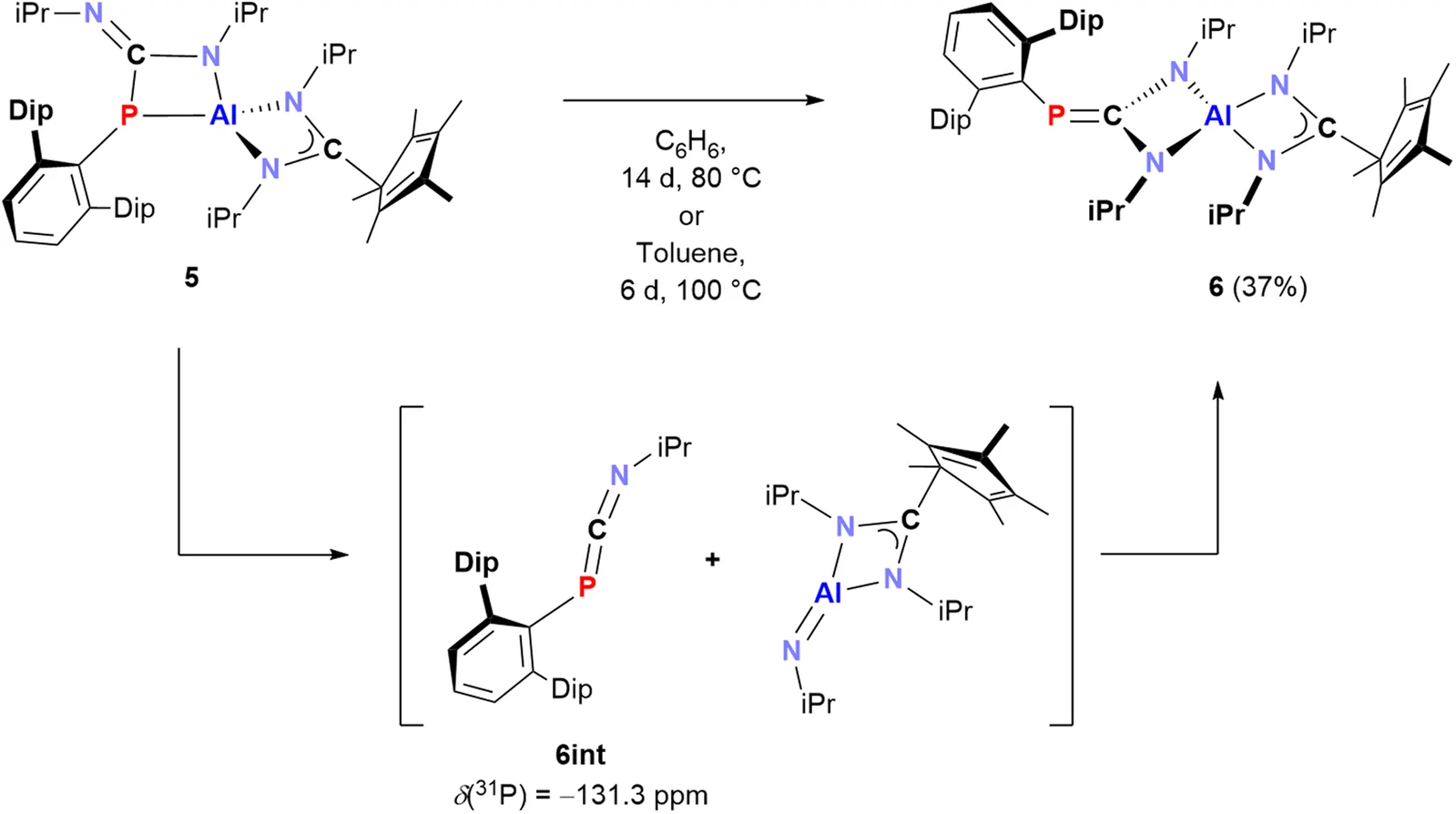
*K*-rearrangement of compound 5 to 6*via* a potential phosphaazaallene intermediate 6int.

Compound 6 crystallises in the triclinic space group *P*1̄ with four molecules in the unit cell. The aluminium amidinate fragment (Fig. 5, right fragment) showed almost unchanged structural parameters (Al1–N3 1.882(3), Al1–N4 1.891(3) Å, C2–N3 1.335(4), C2–N4 1.353(4) Å) when compared to 5. The C–N (C1–N1 1.386(4), C1–N2 1.405(4) Å) and N–Al bond lengths (N1–Al1 1.815(3), N2–Al1 1.845(3) Å) in the aluminium-κ^2^N-phosphaguanidinate fragment are in the range of shortened single bonds (Σ*r*_cov_(N–Al) = 1.97, Σ*r*_cov_(C–N) = 1.46 Å).^15^ Compared to the amidinate fragment, the Al–N distances are shorter and the C–N distances are longer in the PCN_2_Al unit. Compared to the phosphaguanidinate ligand [Ph_2_PC(NiPr)_2_]^−^ with a three-coordinate P atom the Al–N distances in 6 are significantly shorter (*cf.* (Ph_2_PC{NiPr}_2_)AlMe_2_*d*(Al–N) = 1.928(2), 1.927(2) Å).^34^ Overall, the N atoms of the phosphaguanidinate ligand are stronger σ-donors due to the electron-donating phosphinidene fragment. Both N–C–N–Al planes are almost orthogonal to each other at an angle of 83.7(1)°, which places the four-coordinate aluminium atom in a continuum between see-saw and distorted tetrahedral coordination (*τ*_4_ = 0.65).^24^ The P–C bond (1.765(3) Å) is shorter than a typical single bond (*cf.* Σ*r*_cov_(P–C) = 1.86 Å),^15^ and agrees with those reported for inversely polarized PAs, and NHC phosphinidene adducts (*cf.* MesP=IMe_2_: *d*(P=C) = 1.768(4) (Å); IMe_2_ = (HCNMe)_2_C).^37^ The ^31^P NMR shift of 6 at −14.3 ppm and the ^13^C NMR resonance of the P–CN_2_ unit at 200.2 ppm (^1^*J*_PC_ = 102 Hz) further support the classification as inversely polarized PA (*cf.* PhP=C(NiPr_2_)_2_*δ*(^31^P) = 69.5 ppm; *δ*(^13^C) = 199.7 ppm, ^1^*J*_PC_ = 68 Hz).^26^ Considering the formal [:C(NiPr)_2_Al(NiPr)_2_CCp*] carbene fragment this can be classified as moderately π-accepting according to a recent study by Bertrand and co-workers based on the ^31^P and ^13^C NMR signatures of compound 6.

**Fig. 5:**
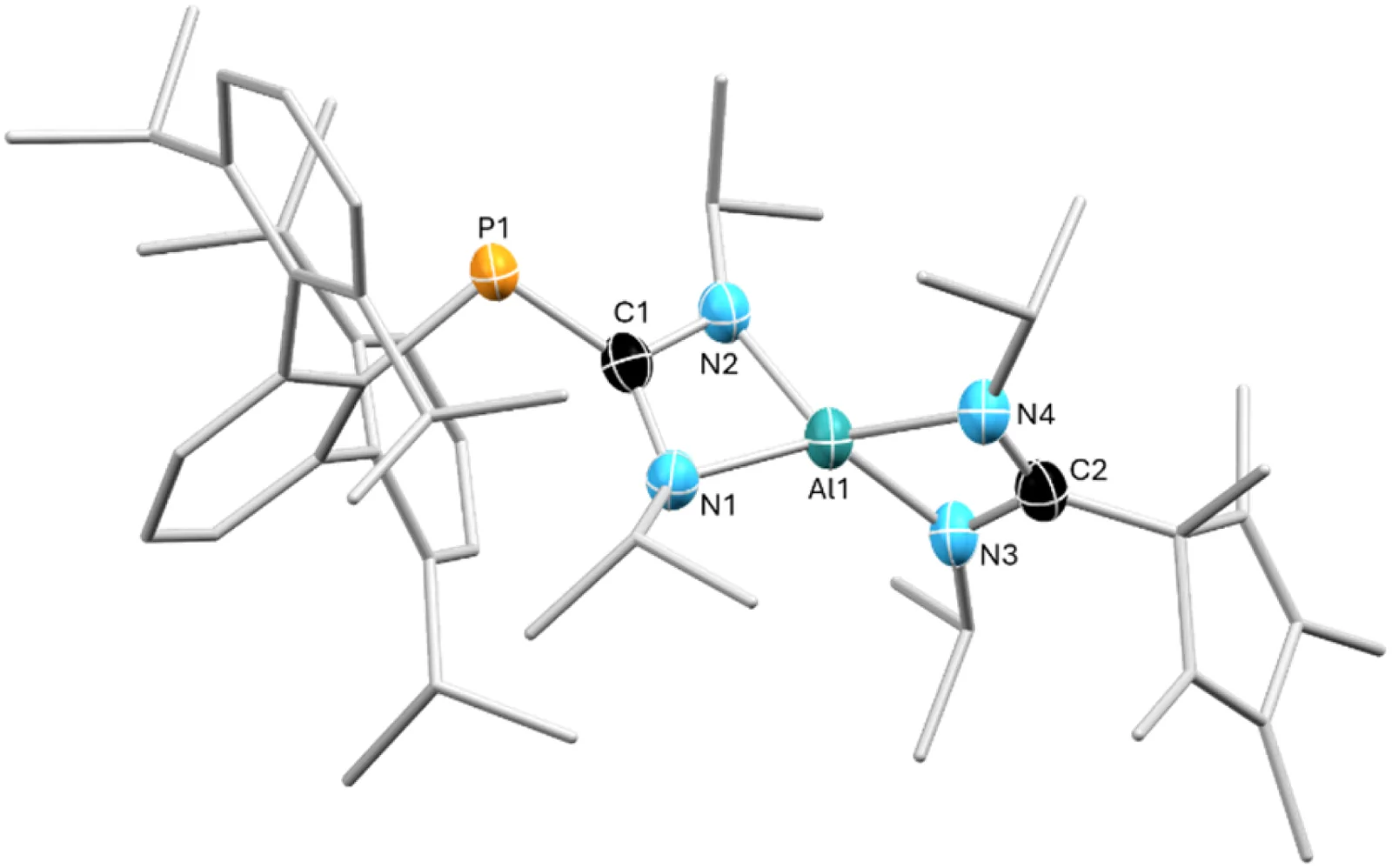
Molecular structure of 6. Ellipsoids drawn at 50% probability with C–H-atoms omitted. Substituents rendered as a wire-frame for clarity. Selected bond lengths [Å] and angles [°]: P1–C1 1.765(3), Al1–N1 1.815(3), Al1–N2 1.845(3), Al1–N3 1.882(3), Al1–N4 1.891(3), Al1–C1 2.307(3), Al1–C2 2.332(3), N1–C1 1.386(4), C1–N2 1.405(4), C2–N4 1.335(4), C2–N3 1.353(4); C(Ter)–P1–C1 106.65(15), N1–Al1–N2 74.11(12), N1–Al1–N3 135.72(13), N2–Al1–N3 132.06(13), N1–Al1–N4 123.71(14), N2–Al1–N4 130.97(13), N3–Al1–N4 70.38(12), N1–C1–N2 104.4(3), N4–C2–N3 108.0(3), N2–Al1–N1–C1 6.44(19), N4–C2–N3–Al1 0.2(3).

The assignment as inversely polarized PA is further supported by calculation of the gas-phase ^31^P NMR shift (*δ*_calc_(^31^P) = −19.5 ppm, *δ*_e*x*p_(^31^P) = −14.3 ppm) and by inspection of the frontier molecular orbitals of 6 (PBE0-D3/def2-TZVP, Fig. 6): the HOMO shows major contributions of a p-type lone pair on the P atom, which is significantly delocalized into the CN_2_ unit, inferring a certain degree of π-bonding. The HOMO−1 shows major contributions for a lone pair of electrons on the P atom with high s-character. NRT analysis of the truncated model compound 6′ revealed that the leading resonance structures show PA character (28%), while overall four resonances indicate inversely polarized PA character (20%). Rather than starting from 5, a mixture of 1 and 2.3 eq. of C(NiPr)_2_ can be directly heated to 100 °C over a period of 5 d, resulting in the formation of 6. The remaining orange solid of 6 was further heated *in vacuo* at 80 °C giving a yellow powder that was washed with hmdso and then recrystallized by slow evaporation of a saturated *n*-hexane solution. scXRD experiments revealed the formation of phosphaguanidine 7 as a racemic mixture.

**Fig. 6:**
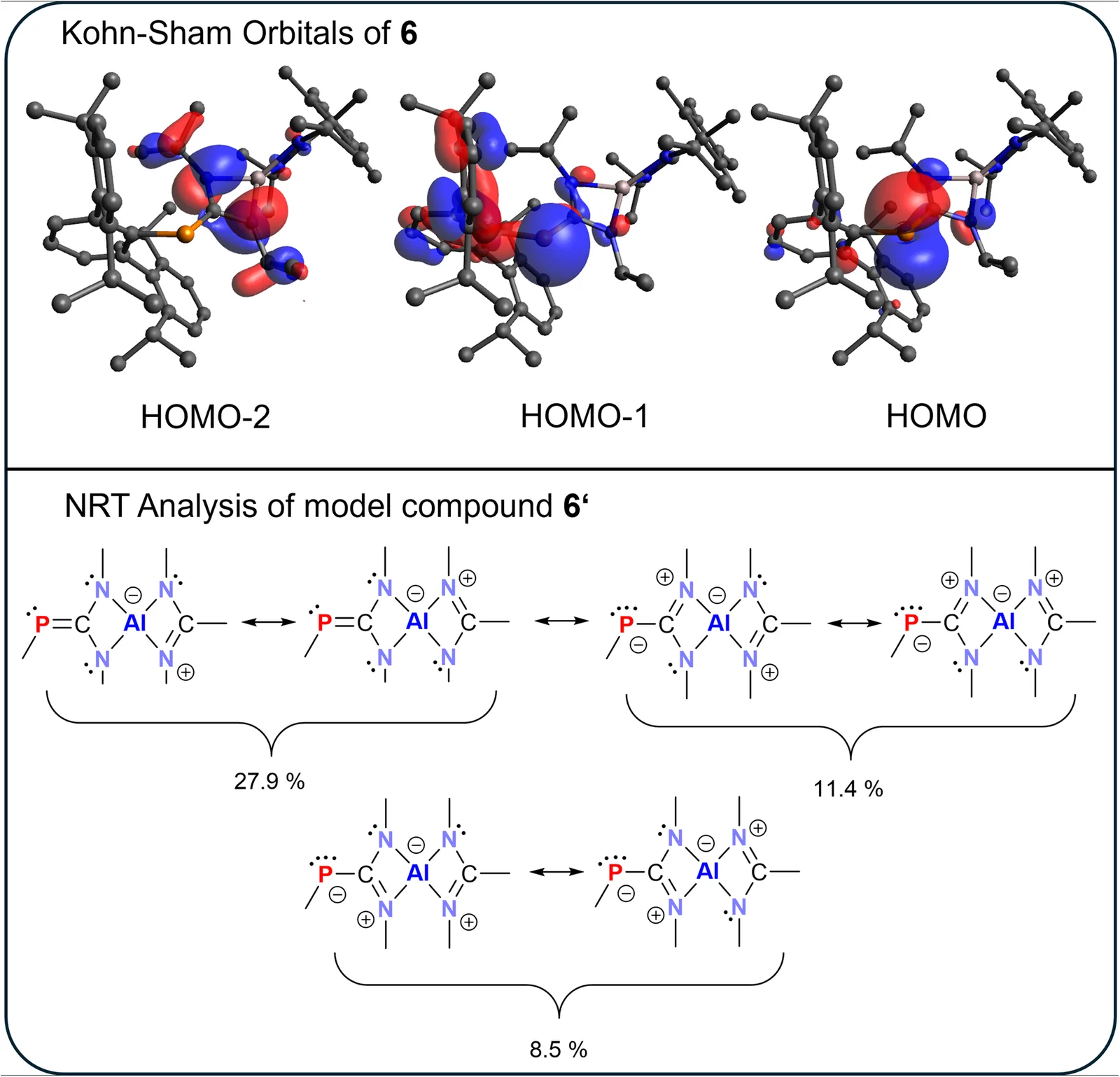
Kohn–Sham orbitals of 6 at the PBE0-D3/def2-TZVP level of theory and the NRT scheme showing the leading resonance structures of model compound 6′.

Targeted hydrolysis of compounds 5 or 6, respectively, also yielded 7 (Scheme 7). The presence of a PH unit in 7 is supported by a ^31^P{^1^H} NMR signal in C_6_D_6_ at −89.7 ppm that splits into a doublet of doublets in the ^31^P NMR spectrum with a large ^1^*J*_PH_ = 224 Hz coupling constant indicative of additional coupling to the NH proton. The corresponding signals for the P*H* proton are detected in the ^1^H NMR spectrum at 4.42 ppm and for the NH proton at 3.59 ppm. Beside 7 a minor product is detected, which corresponds to trimeric aluminoxan [OAl(NiPr)_2_CCp*]_3_, also supported by HRMS-experiments (*cf.* Fig. S47). In the ^1^H NMR spectrum this species shows four signals for the iPr Me-groups, as well as three signals for the Cp*-substituent, indicating an η^1^ Cp*-substituent. Attempts to separate [OAl{(NiPr)_2_CCp*}]_3_ from 7 were not successful, due to similar solubilities of both components. 7 crystallizes in the triclinic space group *P*1̄ as a toluene solvate with two molecules in the unit cell. The P1–C1 bond (1.8825(11) Å) is rather long with a distorted trigonal planar coordination environment at the sp^2^-hybridized C1 atom. The C1–N1 (1.2797(14) Å) and C1–N2 (1.3702(13) Å) correspond to double and single bonds, respectively. 7 is one of the few examples of amidinate-substituted phosphines or phosphaguanidines. Its formation clearly shows the potential of both 5 and 6 to act as a synthetic vehicle for the preparation of unusual phosphines by harnessing the oxophilicity of Al in hydrolysis reactions. Reactions of 1 with other carbodiimides, for example DCC or DDC, proved to be unselective. With DCC one major species with a ^31^P NMR signal at −53.3 ppm was formed (*cf.* Fig. S53), indicating the formation of ^Dip^TerP[C(NCy)_2_]Al[C(NCy)_2_]Cp* (*cf.*5, *δ*(^31^P) = −56.8 ppm). Beside this species, a signal at −67.0 ppm could not be assigned, while the signal at −132.4 ppm is in line with the additional formation of the phosphaazaallene ^Dip^TerPCNCy (*cf.*^Dipp^TerPCN*t*Bu *δ*(^31^P) = −134.8 ppm).^32^ Attempts to obtain crystals from the reaction mixture only afforded minimal amounts of the hydrolysis product ^Dip^TerP(H)C(NHCy)(NCy) (8) (*cf.* Fig. S8), which is in line with the formation of 7.

**Scheme 7:**
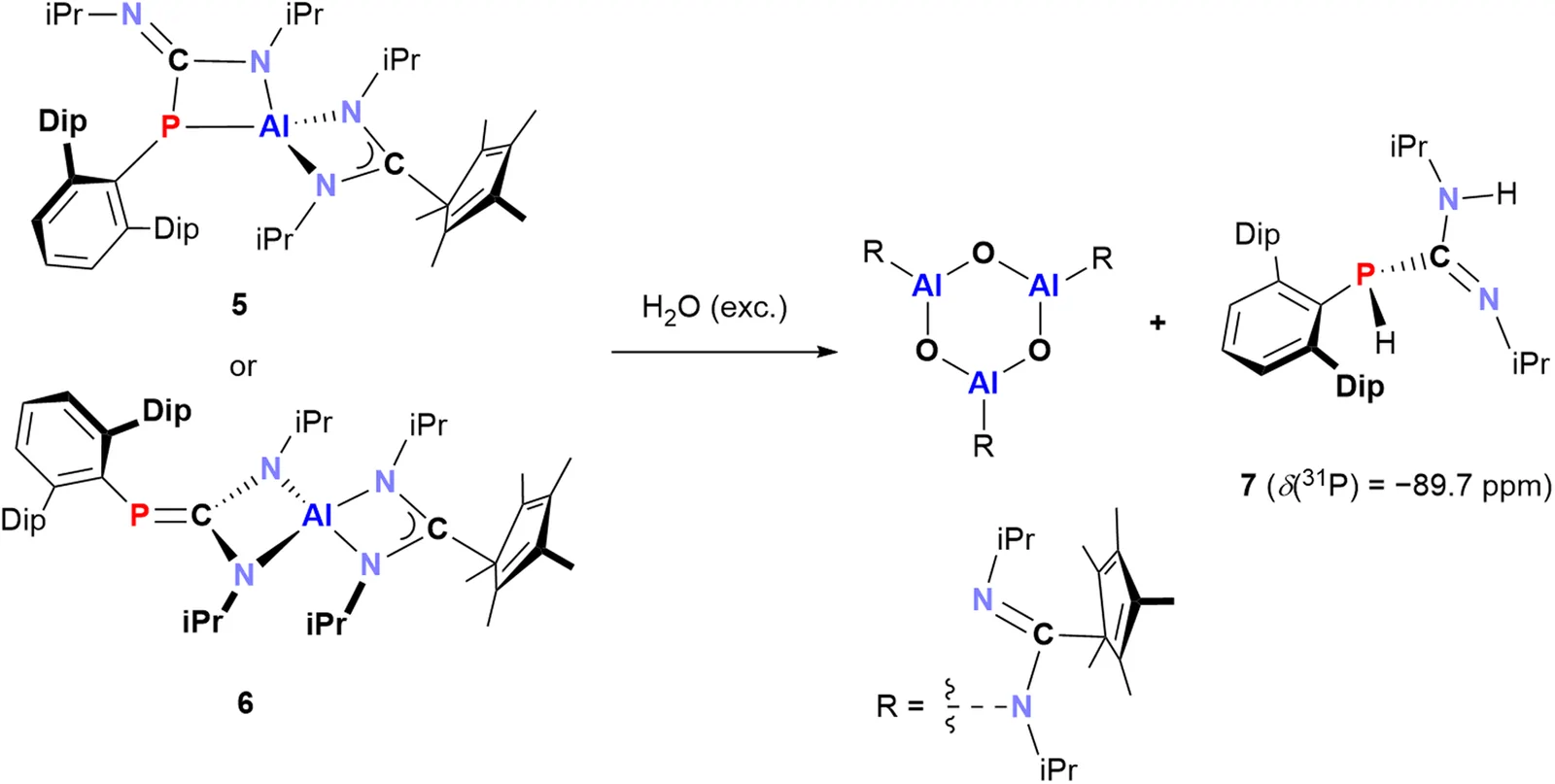
Hydrolysis of 5 or 6, resulting in the formation of phosphaguanidine 7.

## Conclusions

^Dip^TerPAlCp* (1) reacts with CO_2_ to give arylphosphaketene ^Dip^TerPCO (2) along with (CpAlO)_*x*_, which does not interfere with reactivity of 2. Thermally stable 2 is thus predestined as a synthon to build molecular complexity. Compared to the reactivity of other E^13^–P multiply bonded species with CO_2_ the formation of 2 is unusual and clearly illustrates how substitution patterns influence the reactivity of heterodiatomic multiple bonds. We show that the reaction of 2 with 1 gives the cyclic diphosphaurea derivative [(^Dip^TerP)_2_(µ-AlCp*)(µ-CO)] (3), which features a planar P_2_AlC ring. 3 marks the first example of a diphosphaureylene main group complex and was shown to react with CO_2_ to give 2. Moreover, 2 reacts with an NHC-stabilized boraketene to give upon CO extrusion oxaborirane 4, with a localized P=C double bond and a planar BOCP unit. Moreover, the reactivity of 1 with the carbodiimide DIC was evaluated in detail and resulted in the formation of spirocyclic Al species. Initially the reaction of 1 with DIC afforded compound 5 with a PCNAl four-membered ring and an additional amidinate unit, which rearranges after heating at 80 °C for 14 days to produce another spirocyclic compound 6. In 6 the PCNAl ring rearranges with two C(NiPr)_2_ units being symmetrically inserted into the former P=Al bond. Structural analysis and theoretic modelling suggest that 6 is best classified as a phosphaguanidinate or inversely polarized phosphaalkene. To the best of our knowledge, 5 and 6 are the first examples of singly substituted chelating phosphaguanidinate main group complexes showing different κ coordination modes. The hydrolysis of 5 or 6 gave phosphaguanidine ^Dip^TerP(H)C(NHiPr)NiPr (7) and trimeric [OAl(NiPr)_2_CCp*]_3_. Surprisingly, the reactivity towards sterically more hindered carbodiimides proved less selective.

Overall, this study highlights the potential of phosphaalumene 1 as a synthon for arylphosphaketenes and novel heterocyclic structures derived therefrom. The reactivity of 1 towards carbodiimides led to the formation of phosphaguanidine complexes 5 and 6, which are expected to act as ligand transfer reagents. This reactivity differs from that of related phosphagallenes due to the lability of the Al–Cp* bond and studies to harness this lability in the construction of novel phosphaalumenes are currently underway. Moreover, reactivity harnessing the oxophilicity of the aluminium atom in 1 is currently being investigated.

## Author contributions

T. W. and S. N. designed and carried out the experimental work. J. B., P. F., Y. K., D. B., and C. H.-J. carried out the experimental work. T. W. and C. H.-J. collected and analysed the data and assembled the SI. T. W., S. N. and C. H.-J. carried out the scXRD experiments. T. W. and C. H.-J. performed the DFT calculations. H. B. and C. H.-J. supervised the work and drafted the manuscript. All authors have given approval to the final version of the manuscript.

## Conflicts of interest

There are no conflicts to declare.

## Supplementary Material

SC-017-D6SC04181G-s001

SC-017-D6SC04181G-s002

SC-017-D6SC04181G-s003

## Data Availability

Calculated structures are provided in an additional *xyz*-file. Supplementary information (SI): synthetic procedures, spectral data, crystallographic and computational details. See DOI: https://doi.org/10.1039/d6sc04181g. CCDC 2553906 (2dim), 2553907 (3), 2553908 (4), 2553909 (5), 2553910 (6), 2553911 (7), 2553912 (8), and 2582337 (2dim_b) contain the supplementary crystallographic data for this paper.^38*a*–*h*^

## References

[cit1] Goicoechea J. M., Grützmacher H. (2018). Angew. Chem., Int. Ed..

[cit2] Liu L., Ruiz D. A., Munz D., Bertrand G. (2016). Chem.

[cit3] Hansmann M. M., Bertrand G. (2016). J. Am. Chem. Soc..

[cit4] Wilson D. W. N., Feld J., Goicoechea J. M. (2020). Angew. Chem., Int. Ed..

[cit5] Szych L. S., Denker L., Feld J., Goicoechea J. M. (2024). Chem.–Eur. J..

[cit6] Sharma M. K., Weinert H. M., Wölper C., Schulz S. (2024). Chem.–Eur. J..

[cit7] Sharma M. K., Wölper C., Haberhauer G., Schulz S. (2021). Angew. Chem., Int. Ed..

[cit8] Appel R., Paulen W. (1983). Angew Chem. Int. Ed. Engl..

[cit9] Taeufer T., Dankert F., Michalik D., Pospech J., Bresien J., Hering-Junghans C. (2023). Chem. Sci..

[cit10] Wellnitz T., Wüst L., Braunschweig H., Hering-Junghans C. (2025). Chem. Commun..

[cit11] Fischer M., Nees S., Kupfer T., Goettel J. T., Braunschweig H., Hering-Junghans C. (2021). J. Am. Chem. Soc..

[cit12] Szych L. S., Bresien J., Fischer L., Ernst M. J., Goicoechea J. M. (2025). Chem. Sci..

[cit13] Cha Y., Yang Z., Zhuang X., Gao P., Liu T., Luo F., Ni X., Luo Q., Lu W. (2026). Nat. Commun..

[cit14] Stelzer A. C., Hrobárik P., Braun T., Kaupp M., Braun-Cula B. (2016). Inorg. Chem..

[cit15] Pyykkö P., Atsumi M. (2009). Chem.–Eur. J..

[cit16] Heift D., Benkő Z., Grützmacher H. (2014). Dalton Trans..

[cit17] Nasrullah A. A., Täufer T., Pospech J., Baráth E., Hering-Junghans C. (2025). Dalton Trans..

[cit18] David M.-A., Glueck D. S., Yap G. P. A., Rheingold A. L. (1995). Organometallics.

[cit19] David M.-A., Wicht D. K., Glueck D. S., Yap G. P. A., Liable-Sands L. M., Rheingold A. L. (1997). Organometallics.

[cit20] Konrad Y. , Arrowsmith M. and Braunschweig H., ChemRxiv, 2025, preprint, 10.26434/chemrxiv-2025-l9qnb

[cit21] Price A. N., Cowley M. J. (2016). Chem.–Eur. J..

[cit22] Koner A., Morgenstern B., Andrada D. M. (2022). Angew. Chem., Int. Ed..

[cit23] Zhu L., Kinjo R. (2023). Chem. Commun..

[cit24] Yang L., Powell D. R., Houser R. P. (2007). Dalton Trans..

[cit25] Iglesias M., Beetstra D. J., Knight J. C., Ooi L.-L., Stasch A., Coles S., Male L., Hursthouse M. B., Cavell K. J., Dervisi A., Fallis I. A. (2008). Organometallics.

[cit26] Back O., Henry-Ellinger M., Martin C. D., Martin D., Bertrand G. (2013). Angew. Chem., Int. Ed..

[cit27] Dankert F., Hevia E. (2024). Chem.–Eur. J..

[cit28] Nees S., Wellnitz T., Dankert F., Härterich M., Dotzauer S., Feldt M., Braunschweig H., Hering-Junghans C. (2023). Angew. Chem., Int. Ed..

[cit29] Coles M. P., Swenson D. C., Jordan R. F., Young V. G. (1997). Organometallics.

[cit30] Pierce G. A., Aldridge S., Jones C., Gans-Eichler T., Stasch A., Coombs N. D., Willock D. J. (2007). Angew. Chem., Int. Ed..

[cit31] Manßen M., de Graaff S., Meyer M.-F., Schmidtmann M., Beckhaus R. (2018). Organometallics.

[cit32] Fischer M., Reiß F., Hering-Junghans C. (2021). Chem. Commun..

[cit33] Breen T. L., Stephan D. W. (1995). J. Am. Chem. Soc..

[cit34] Coles M. P., Hitchcock P. B. (2002). Chem. Commun..

[cit35] Crimmin M. R., Barrett A. G. M., Hill M. S., Hitchcock P. B., Procopiou P. A. (2008). Organometallics.

[cit36] Issleib K., schmidt H., Meyer H. (1980). J. Organomet. Chem..

[cit37] Adhikari A. K., Grell T., Lönnecke P., Hey-Hawkins E. (2016). Eur. J. Inorg. Chem..

[cit38] (a) CCDC 2553906: Experimental Crystal Structure Determination, 2026, 10.5517/ccdc.csd.cc2rqk24

