# Supplementary material for: Reactivity of a phosphaalumene towards heteroallenes
Source: Chem Sci. 2026 Aug 31;17(41):20011–8. doi: 10.1039/d6sc04181g (PMC13555904; doi:10.1039/d6sc04181g)
Supplement: Supplementary file 1 [file SC-017-D6SC04181G-s001.pdf]

Supplementary Information (SI) for Chemical Science.
This journal is © The Royal Society of Chemistry 2026

SUPPORTING INFORMATION

Reactivity of a phosphaalumene towards heteroallenes

Tim Wellnitz, Jakob Boardman, Paul Fritsche, Yannick Konrad, Diana Bröllos, Samuel Nees, Holger Braunschweig, Christian Hering-Junghans**

This file includes:

1. Experimental....3
2. Structure elucidation....6
   X-ray structure determination....6
   Special refinement details....7
3. Syntheses of compounds....21
   3.1. Improved *in-situ* generation of ^{Dip}TerPCO **2**....21
   3.2. Synthesis of **3**....29
   3.3. Synthesis of **4**....35
   3.4. Synthesis of **5**....42
   3.5. Synthesis of **6**....48
   3.6. Synthesis of **7**....53
4. Reactivity of **3** and additional substrate scope....60
   4.1. Isolation of [^{Dip}TerPCO]$_2$ (**2dim**)....60
   4.2. Reaction of **3** with CO_2....63
   4.3. Reaction of **^{Dip}TerPAlCp*** with DCC....64
   4.4. Reaction of **^{Dip}TerPAlCp*** with DDC....66
5. Computational details....67

5.1. Summary of calculated data 68

5.2. NBO analyses 69

5.3. Kohn-Sham Orbitals 76

5.3. Thermodynamic Considerations 81

6. References 82

1. Experimental

General Information. If not stated otherwise, all manipulations were carried out under oxygen- and moisture-free conditions in an inert argon atmosphere using standard Schlenk or drybox techniques. All glassware was heated three times *in vacuo* (~1×10^{-3} mbar) using a heat gun (650 °C) and cooled under argon atmosphere. Solvents were transferred using syringes, which were purged three times with argon prior to use or steel cannulas and PTFE cannulas, which were stored at 120 °C or 80 °C and purged three times with argon prior to use. Solvents and reactants were either obtained from commercial sources or synthesized as detailed in ***Table S1***.

Table S1. Origin and purification of solvents and reactants.

Substance	Origin	Purification
Benzene	Sigma Aldrich	Dried over Na/benzophenone, freshly distilled prior to use. Degassed (freeze-pump-thawn), stored and further dried over molecular sieves (4 Å)
n-Hexane	Local trade	Dried over Na/benzophenone, freshly distilled prior to use. Degassed (freeze-pump-thawn), stored and further dried over molecular sieves (4 Å)
n-Pentane	Local trade	Dried over Na/benzophenone, freshly distilled prior to use. Degassed (freeze-pump-thawn), stored and further dried over molecular sieves (4 Å)
Toluene	Fischer Chemicals, for HPLC<br>or<br>Local trade	Purified with the Grubbs-type column system "Pure Solv MD-5". Degassed (freeze-pump-thawn), further dried and stored over molecular sieves (4 Å)<br>or<br>Dried over Na/benzophenone, freshly distilled prior to use. Degassed (freeze-pump-thawn), stored and further dried over molecular sieves (4 Å)
Hexamethyldisiloxan (HMDSO)	Sigma Aldrich, ≥98.0%	Dried over Na/benzophenone, freshly distilled prior to use. Degassed (freeze-

		pump-thawn), stored and further dried over molecular sieves (4 Å)
Benzene-d_6	Euriso-top	Dried over Na, freshly distilled prior to use. Stored and further dried over molecular sieves (4 Å)
n-Hexane-d_{14}	Sigma-Aldrich	Dried over Na, freshly distilled prior to use. Stored and further dried over molecular sieves (4 Å)
Toluene-d_8	Euriso-top	Dried over Na, freshly distilled prior to use. Stored and further dried over molecular sieves (4 Å)
N,*N'*-Di(propan-2-yl)methanediimine (DIC)	Sigma-Aldrich	Re-condensed prior to use and stored over molecular sieves (4 Å)
N,N'-Dicyclohexylmethanediimine (DCC)	Sigma-Aldrich	Used as received and dried in vacuo (1×10^{-3} mbar)
N,N'-Bis-(2,6-diisopropylphenyl)methanediimine (DDC)	Sigma-Aldrich	Used as received
CO_2		Used as received
^{Dipp}TerPAlCp*	synthesized[1]	
IDipp(Cy)BCO	synthesized[2]	

NMR spectra were recorded on Bruker spectrometers (AVANCE 400, AVANCE 500, Bruker Avance Neo I 600) and were referenced internally to the deuterated solvent (C_6D_6 δ_{ref} = 128.4 ppm; toluene-d_8 $\delta_{ref,1}$ = 20.4 ppm, $\delta_{ref,2}$ = 125.5 ppm, $\delta_{ref,3}$ = 128.3 ppm, $\delta_{ref,4}$ = 129.2 ppm, $\delta_{ref,5}$ = 137.9 ppm; *n*-hexane-d_{14} $\delta_{ref,1}$ = 20.4 ppm, $\delta_{ref,2}$ = 125.5 ppm, $\delta_{ref,3}$ = 128.3 ppm), to protic impurities in the deuterated solvent (C_6HD_5 δ_{ref} = 7.16 ppm, toluene-d_8 $\delta_{ref,1}$ = 2.08 ppm, $\delta_{ref,2}$ = 6.97 ppm, $\delta_{ref,3}$ = 7.01 ppm, $\delta_{ref,4}$ = 7.09 ppm)[3] or externally (^{11}B: $BF_3\cdot OEt_2$ δ_{ref} = 0 ppm, ^{31}P: 85% H_3PO_4 δ_{ref} = 0 ppm). NMR signals were assigned using experimental data (e.g. chemical shifts, coupling constants, integrals where applicable).

IR spectra of crystalline samples were recorded on a Bruker Alpha II FT-IR spectrometer equipped with an ATR unit at ambient temperature under argon atmosphere. Relative

intensities are reported according to the following intervals: weak (w, 0–33%), medium (m, 33–66%), strong (s, 66–100%).

Mass spectra were recorded on a Thermo Scientific Exactive Plus spectrometer in LIFDI mode using crystalline samples.

2. Structure elucidation

X-ray structure determination

X-ray quality crystals of **5, 6**, **7**, **8, 3**, **4** and **2dim** were selected in Fomblin© Y perfluoroether (Sigma Aldrich) at ambient temperature under a constant stream of argon.

The samples were cooled to the temperature given in ***Table S3*** during measurement. The data of **5**, **6**, **7**, **8**, **3** and **4** were collected on a Rigaku XtaLAB Synergy-R diffractometer with a HPA area detector and multi-layer mirror monochromated CuK_{α} radiation (λ = 1.54178 Å).

The crystal data of **2dim** were collected on a BRUKER D8-QUEST diffractometer with a CPA area detector and multi-layer mirror monochromated MoK_{a} radiation (λ = 0.7107 Å).

The crystal data of **2dim_b** were collected on a BRUKER APEX2-DUO diffractometer with a CCD detector and monochromated CuK_{a} radiation (λ = 1.54178 Å) at 150(2) K.

The structures **5**, **6**, **7**, **8**, **3**, **4**, **2dim** and **2dim_b** were solved using intrinsic phasing method[4], refined with the SHELXL program[5] and expanded using Fourier techniques. All non-hydrogen atoms were refined anisotropically, hydrogen atoms were included in the refinement at calculated positions using a riding model.

Special refinement details

In Compound **2dim** the reflections [0 1 1] and [0 -1 1] were removed from refinement, because of low-resolution affected by the beamstop. One isopropyl group and two dipp groups are disordered. The displacement parameters of the atoms of the disordered isopropyl group C20_5 > C22_51, of the disordered dipp group C39_10 > C56_131 and of the disordered dipp group C45_11 > C62_151 were restrained with RIGU keyword with esd = 0.008 in ShelXL input ('enhanced rigid bond' restraint). The U_{ij} displacement parameters of the atoms of the disordered isopropyl group C20_5 > C22_51, of the disordered dipp group C39_10 > C56_131 and of the disordered dipp group C45_11 > C62_151 were restrained with similarity restraint SIMU with esd = 0.016. The U_{ii} displacement parameters of the atoms of the disordered isopropyl group C20_5 > C22_51, of the disordered dipp group C39_10 > C56_131 and of the disordered dipp group C45_11 > C62_151 were restrained with ISOR keyword with esd = 0.004 to approximate isotropic behavior. The distances between atoms C9_3 to C20_5 and C9_3 to C20_51, C40_10 to C51_12, C40_101 to C51_121, C44_10 to C54_13, C44_101 to C54_131, C50_11 to C60_15, C50_111 to C60_151, C46_11 to C57_14 and C46_111 to C57_141 were restrained to the value of 1.52 with esd = 0.005 using DFIX. The distances between atoms C38_9 to C45_11 and C38_9 to C45_111, were restrained to the value of 1.52 with esd = 0.005 using DFIX.

Compound **3** contains two disordered *n*-pentane molecules and one benzene molecule, which were located on a symmetry element and were isotropically refined as special position disorder. The displacement parameters of the benzene atoms C1_17 to C6_17 were restrained to the same value with similarity restraint SIMU (esd = 0.002). Idealized geometry of the benzene ring was restrained using AFIX 66. The distances between the *n*-pentane atoms C1 to C2, C2 to C3, C3 to C4, C4 to C5 and C1 to C3, C3 to C5 and C2 to C4 were restrained to the value of 1.531 with esd = 0.002 and 2.552 with esd = 0.003 using DFIX[6[3]].

In Compound **4** the reflections [-1 1 1] were removed from refinement as outliers. The structure contains two disordered benzene molecules and idealized geometry was applied using AFIX 66. The atomic displacement parameters of all benzene atoms C1_17 to C6_181

were restraint with RIGU keyword with esd = 0.008 in ShelXL input ('enhanced rigid bond' restraint for all bonds in the connectivity list). The displacement parameters of all benzene atoms C1_17 to C6_181 were restrained to the same value with similarity restraint SIMU with esd = 0.008. The U_{ii} displacement parameters of all benzene atoms C1_17 to C6_181 were restrained with ISOR keyword to approximate isotropic behavior with esd = 0.008.

In compound **5** the reflections [2 1 0] and [0 0 4] were removed from refinement as outliers. The *n*-pentane molecule is disordered. The atomic displacement parameters of all *n*-pentane atoms C1_14 to C5_141 were restraint with RIGU keyword with esd = 0.016 in ShelXL input ('enhanced rigid bond' restraint for all bonds in the connectivity list). The displacement parameters of all *n*-pentane atoms C1_14 to C5_141 were restrained to the same value with similarity restraint SIMU with esd = 0.016. The U_{ii} displacement parameters of all *n*-pentane atoms C1_14 to C5_141 were restrained with ISOR keyword with esd = 0.016 to approximate isotropic behavior. The distances between the *n*-pentane atoms C1 to c2, C2 to C3, C3 to C4, c4 to C5 were restrained to the value of 1.53 with esd = 0.03 and C1 to C3, C3 to C5 and C2 to C4 to the the value of 2.55 with esd = 0.03 using DFIX6[3].

Compound **6** was refined as a 2-component twin. Component 2 rotated by –179.8809° around [1.00 –0.00 –0.00] (reciprocal) or [0.99 –0.07 –0.09] (direct) The BASF parameter was refined to 36.7%. Both *n*-pentane molecules are disordered. The atomic displacement parameters of *n*-pentane atoms C1_27 to C5_281 were restraint with RIGU (esd = 0.032) keyword in ShelXL input ('enhanced rigid bond' restraint for all bonds in the connectivity list). The displacement parameters of *n*-pentane atoms C1_27 to C5_281 were restrained to the same value with similarity restraint SIMU with esd = 0.032. The U_{ii} displacement parameters of *n*-pentane atoms C1_27 to C5_281 were restrained with ISOR keyword to approximate isotropic behavior (esd = 0.032). The distances between *n*-pentane atoms c1 to c2, c2 to c3, c3 to c4 and c4 to c5 were restrained to the value of 1.52 (s = 0.01) and c1 to c3, c3 to c5 and c2 to c4 were restrained to the value of 2.52 (s = 0.04) using DFIX6[3].

Compound **7** contains two disordered toluene molecules and refined by using DSR7[4] plugin. The atomic displacement parameters of the disordered toluene atoms C1_22 to C7_231

were restraint with RIGU keyword with esd = 0.008 in ShelXL input ('enhanced rigid bond' restraint for all bonds in the connectivity list). The displacement parameters of the disordered toluene atoms C1_22 to C7_231 were restrained to the same value with similarity restraint SIMU with esd = 0.008. The U_{ii} displacement parameters of the disordered toluene atoms C1_22 to C7_231 were restrained with ISOR keyword with esd = 0.008 to approximate isotropic behavior. Idealized geometry of the disordered toluene molecules was applied using AFIX 6.

Table S2: Crystallographic details of **2dim**, **3** and **4**.

Compound	**2dim**	**3**	**4**
Chem. Formula	$C_{62}H_{74}O_2P_2$	$C_{79}H_{104}AlOP_2$	$C_{76}H_{96}BN_2OP$
Formula weight [g/mol]	913.15	1158.54	1095.32
Colour	colourless	colourless	colourless
Crystal system	triclinic	monoclinic	triclinic
Space group	$P\bar{1}$	$C2/c$	$P\bar{1}$
a [Å]	12.270(8)	45.9229(3)	12.08650(10)
b [Å]	12.573(5)	13.94590(10)	14.31360(10)
c [Å]	18.061(7)	22.14470(10)	19.6917(2)
α [°]	101.833(6)	90	93.7760(10)
β [°]	92.887(13)	102.6100(10)	101.7540(10)
γ [°]	100.15(2)	90	92.4830(10)
V [Å^3]	2674(2)	13840.17(16)	3322.40(5)
Z	2	8	2
$\rho_{calcd.}$ [g/cm^3]	1.134	1.112	1.095
μ [mm^{-1}]	0.123	1.009	0.690
T [K]	100(2)	100(2)	100(2)
Measured reflections	42465	159780	78518
Independent reflections	10985	13889	13121
Reflections with $I > 2\sigma(I)$	7272	12080	11840
R_{int}	0.0995	0.0609	0.0367
$F(000)$	984	5032	1188
$R_1(R[F^2>2\sigma(F^2)])$	0.0551	0.0513	0.0558
$wR_2(F^2)$	0.1202	0.1424	0.1573
GooF	1.022	1.020	1.022
No. of Parameters	847	749	808
CCDC #	2553906	2553907	2553908

Table S3: Crystallographic details of **5**, **6** and **7**.

Compound	**5**	**6**	**7**
Chem. Formula	$C_{59}H_{92}AlN_4P$	$C_{59}H_{92}AlN_4P$	$C_{95}H_{130}N_4P_2$
Formula weight [g/mol]	915.31	915.31	1389.96
Colour	colourless	colourless	colourless
Crystal system	monoclinic	triclinic	triclinic
Space group	$P2_1/n$	$P\bar{1}$	$P\bar{1}$
a [Å]	20.98470(10)	16.3376(3)	10.06320(10)
b [Å]	12.21880(10)	18.9455(2)	19.3958(2)
c [Å]	22.50260(10)	19.2158(2)	22.2835(2)
α [°]	90	79.2860(10)	95.2250(10)
β [°]	105.0120(10)	83.0240(10)	90.3300(10)
γ [°]	90	83.9650(10)	102.3170(10)
V [Å^3]	5572.93(6)	5779.98(14)	4230.19(7)
Z	4	4	2
$\rho_{calcd.}$ [g/cm^3]	1.091	1.052	1.091
μ [mm^{-1}]	0.873	0.841	0.807
T [K]	100(2)	100(2)	100(2)
Measured reflections	106832	40126	98748
Independent reflections	11188	40126	16441
Reflections with $I > 2\sigma(I)$	10285	26571	16047
R_{int}	0.0232	0.0752	0.0165
$F(000)$	2008	2008	1516
$R_1(R[F^2>2\sigma(F^2)])$	0.0386	0.0705	0.0352
$wR_2(F^2)$	0.1035	0.1856	0.0891
GooF	1.027	1.065	1.040
No. of Parameters	653	1306	999
CCDC #	2553909	2553910	2553911

Table S4: Crystallographic details of **8** and **2dim_b**.

Compound	**8**	**2dim_b**
Chem. Formula	$C_{50}H_{69}N_2P$	$C_{62}H_{74}O_2P_2 \cdot C_6H_{14}$
Formula weight [g/mol]	729.04	999.32
Colour	clear yellow	pale yellow
Crystal system	triclinic	monoclinic
Space group	$P\bar{1}$	$P2_1/c$
a [Å]	10.5722(2)	13.3525(5)
b [Å]	12.8146(2)	14.0624(5)
c [Å]	17.9786(3)	16.1144(5)
α [°]	71.8110(10)	90
β [°]	88.9260(10)	98.626(2)
γ [°]	68.5400(10)	90
V [Å^3]	2141.00(7)	2991.55(18)
Z	2	2
$\rho_{calcd.}$ [g/cm^3]	1.131	1.109
μ [mm^{-1}]	0.819	0.972
T [K]	100(2)	150(2)
Measured reflections	51441	32606
Independent reflections	8328	5104
Reflections with $I > 2\sigma(I)$	8207	4041
R_{int}	0.0155	0.0804
$F(000)$	796	1084
$R_1(R[F^2>2\sigma(F^2)])$	0.0340	0.0621
$wR_2(F^2)$	0.0826	0.1776
GooF	1.033	1.022
No. of Parameters	490	334
CCDC #	2553912	2582337

Figure S1: Molecular structure of **2dim**. Ellipsoids are drawn at 50% probability (100(2) K) with H-atoms and two benzene molecules omitted. Carbon atoms, except C1, rendered as wireframe for clarity. Selected bond lengths [Å] and angles [°]: P1–C1 1.851(3), P1–C1' 1.849(2), O1–C1 1.210(3), C1–C1' 2.436(3), P1–P1' 2.785(1); C1–P1–C1' 82.37(12), P1–C1–P1' 97.63(12), P1–C1–O1 131.33(19), P1'–C1–O1 131.0(2); P1–C1–P1'–C1' 0.0(1), C1'–P1–C1–O1 179.4(3).

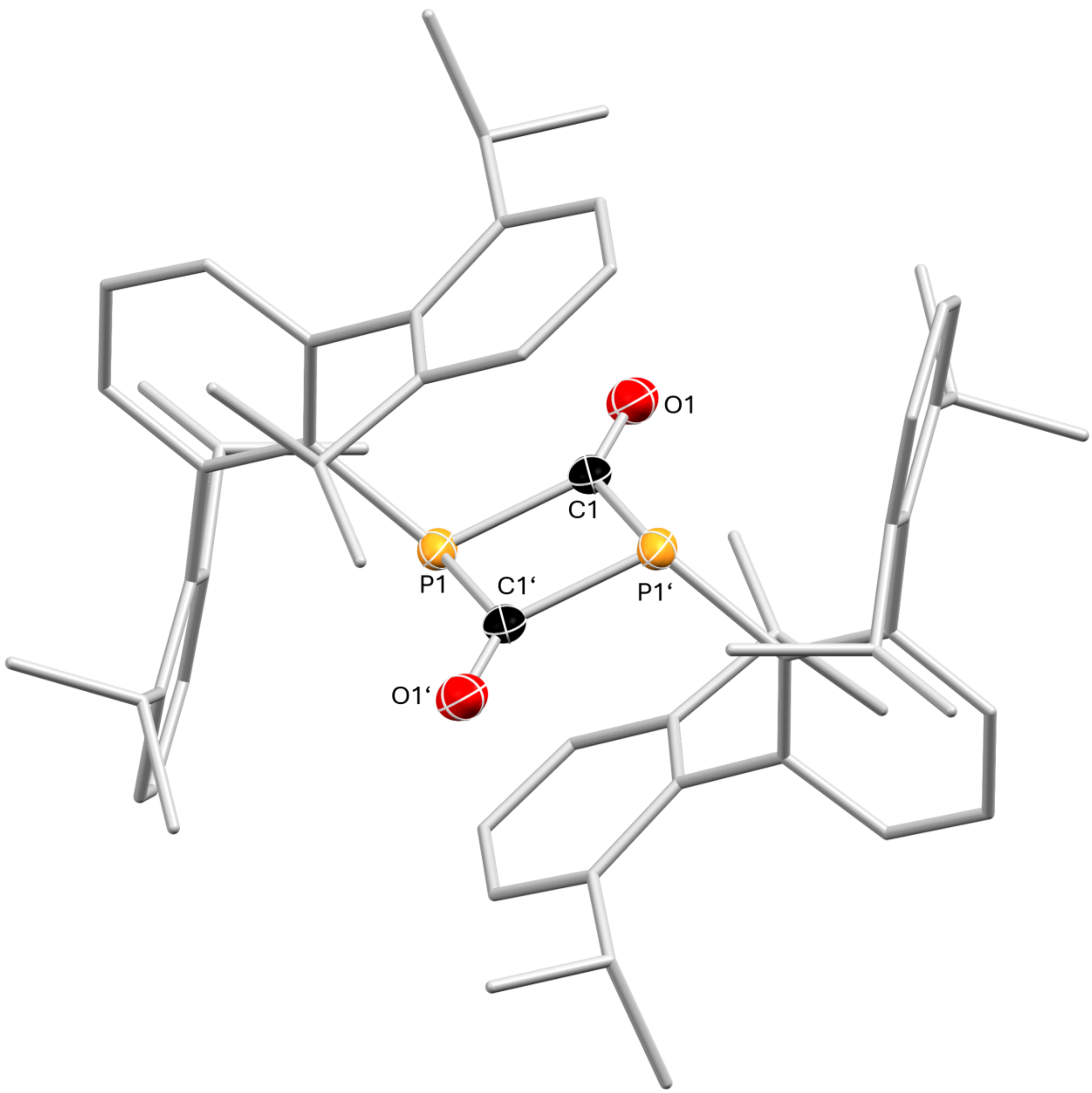

Figure S2: Molecular structure of **2dim_b**. Ellipsoids are drawn at 50% probability (150(2) K) with H-atoms and two benzene molecules omitted. Carbon atoms, except C31, rendered as wireframe for clarity. Selected bond lengths [Å] and angles [°]: P1–C1 1.857(3), 1.860(3), O1–C1 1.201(3); C1–P1–C1' 82.77(12), P1–C1–P1' 97.23(12), P1–C1–O1 131.3(2), P1'–C1–O1 131.4(2); P1–C1–P1'–C1' -0.002(0), C1'–P1–C1–O1 -177.1(3).

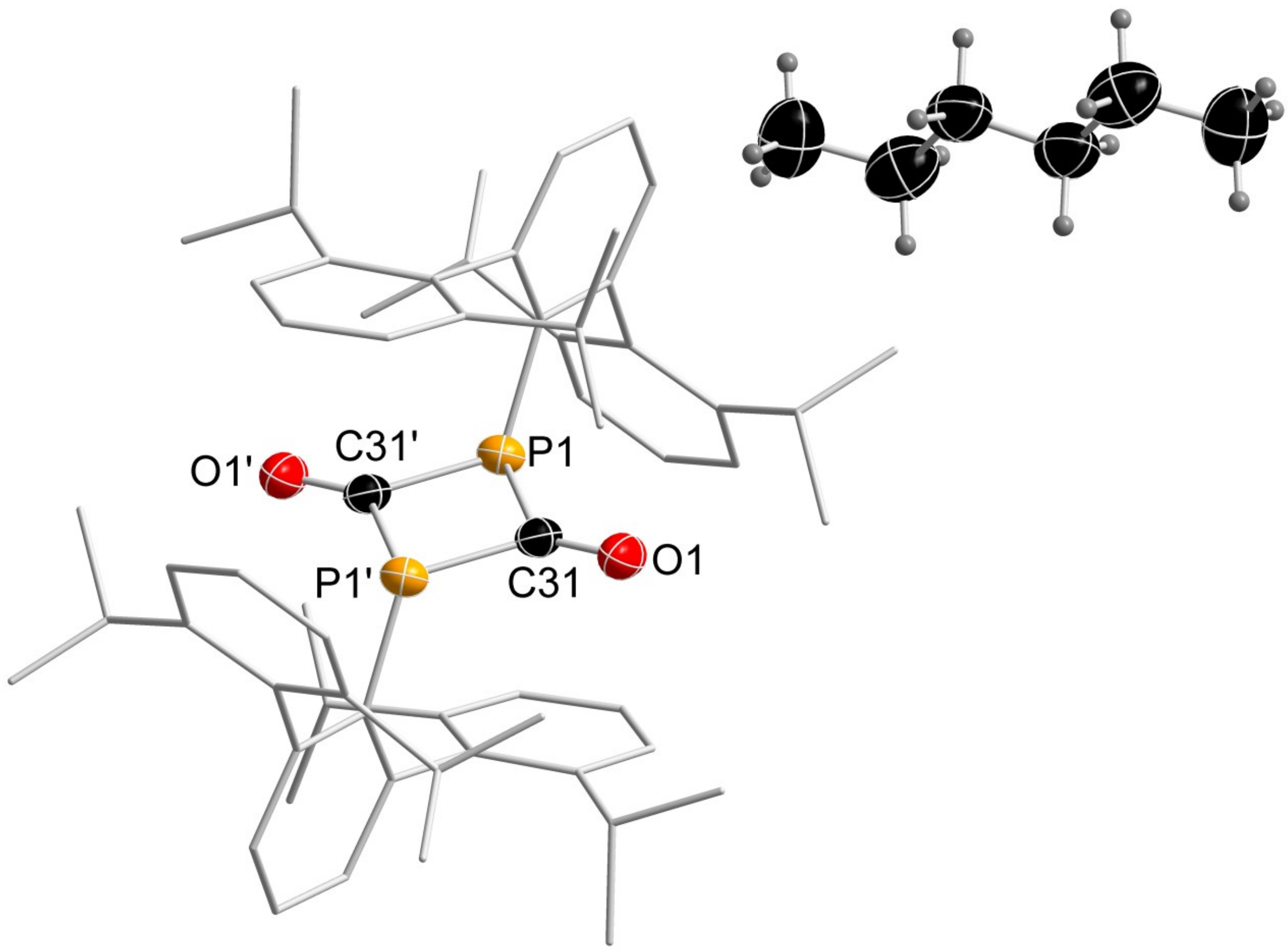

Figure S3: Molecular structure of **3**. Ellipsoids are drawn at 50% probability (100(2) K) with H-atoms, two *n*-pentane molecules and one benzene molecule omitted. Carbon atoms, except C1, rendered as wireframe for clarity. Selected bond lengths [Å] and angles [°]: P1−C1 1.8198(18), P1−C2 1.8434(19), Al1−P1 2.3693(7), Al1−P2 2.3600(7), O1−C1 1.223(2), P1−P2 2.9780(6), C1−Al1 2.904(2); P1−C1−O1 126.51(14), P2−C1−O1 124.72(14), P1−C1−P2 108.77(10), P1−Al1−P2 78.06(2), C1−P1−Al1 86.71(6), C1−P2−Al1 86.46(6); P1−C1−P2−Al1 0.03(8), O1−C1−P1−Al1 179.58(18), O1−C1−P2−Al1 179.59(17).

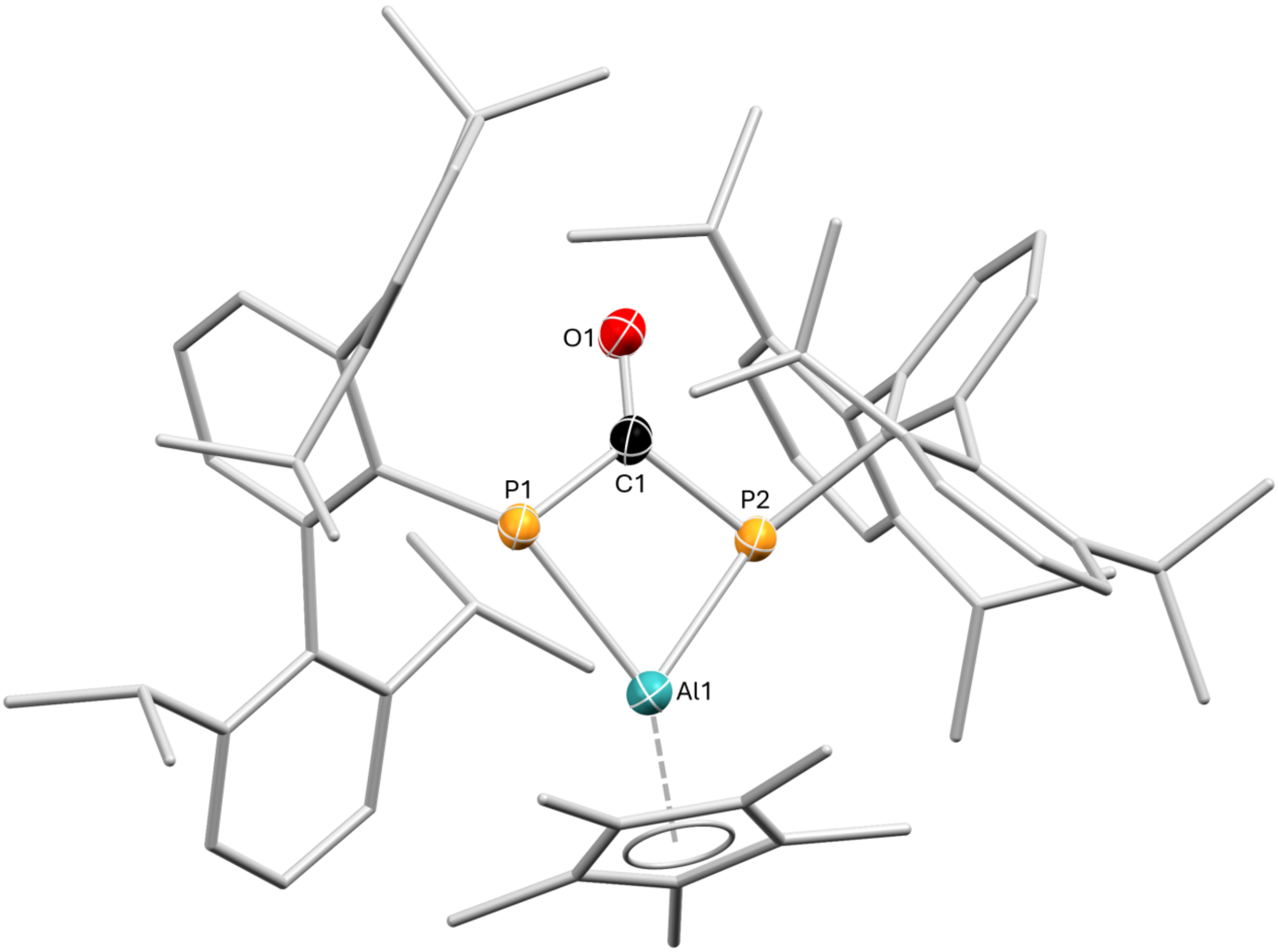

Figure S4: Molecular structure of **4**. Ellipsoids are drawn at 50% probability (100(2) K) with H-atoms and two benzene molecules omitted. Carbon atoms, except C1, rendered as wireframe for clarity. Selected bond lengths [Å] and angles [°]: P1–C1 1.6790(16), O1–C1 1.3463(19), O1–B1 1.558(2), C1–B1 1.547(2); C1–O1–B1 63.89(10), O1–C1–B1 64.73(11), O1–C1–P1 140.81(13), B1–C1–P1 154.15(13), C1–B1–O1 51.39(9), B1–O1–C1–P1 174.8(2).

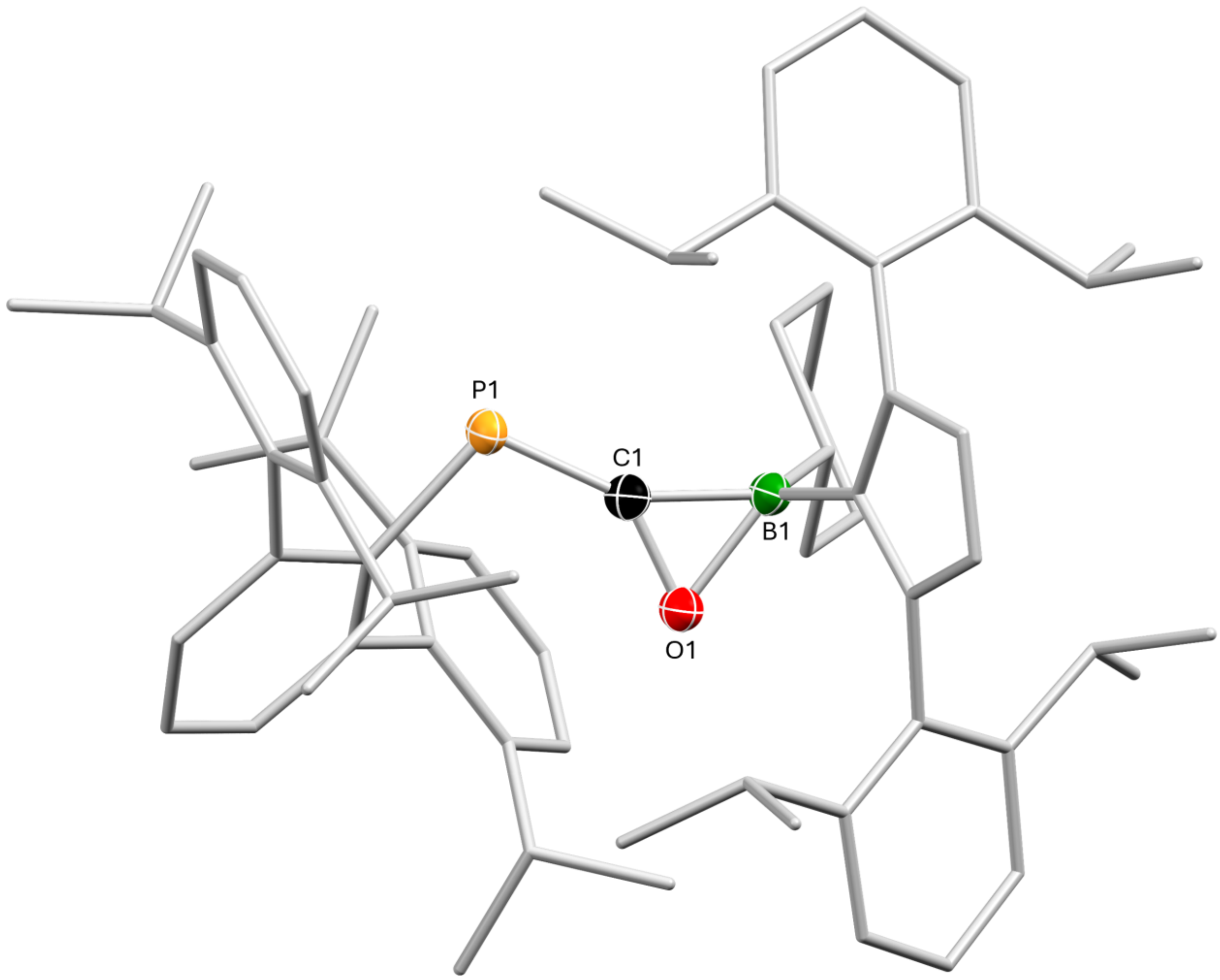

Figure S5: Molecular structure of **5**. Ellipsoids are drawn at 50% probability (100(2) K) with H-atoms and *n*-pentane molecule omitted. Carbon atoms, except C1 and C2, rendered as wireframe for clarity. Selected bond lengths [Å] and angles [°]: P1–C1 1.9254(13), P1–Al1 2.3443(5), Al1–N2 1.8268(11), Al1–N4 1.8913(11), Al1–N3 1.8999(11), Al1–C2 2.3527(13), Al1–C1 2.5157(13), N1–C1 1.2768(16), C1–N2 1.4014(16), C2–N3 1.3332(16), C2–N4 1.3614(16); C1–P1–Al1 71.43(4), N2–Al1–N4 124.76(5), N2–Al1–N3 124.15(5), N4–Al1–N3 69.85(5), N2–Al1–P1 79.25(4), N1–C1–N2 121.85(11), N3–C2–N4 107.30(11), P1–C1–N2–Al1 9.03(9), N4–C2–N3–Al1 1.09(10).

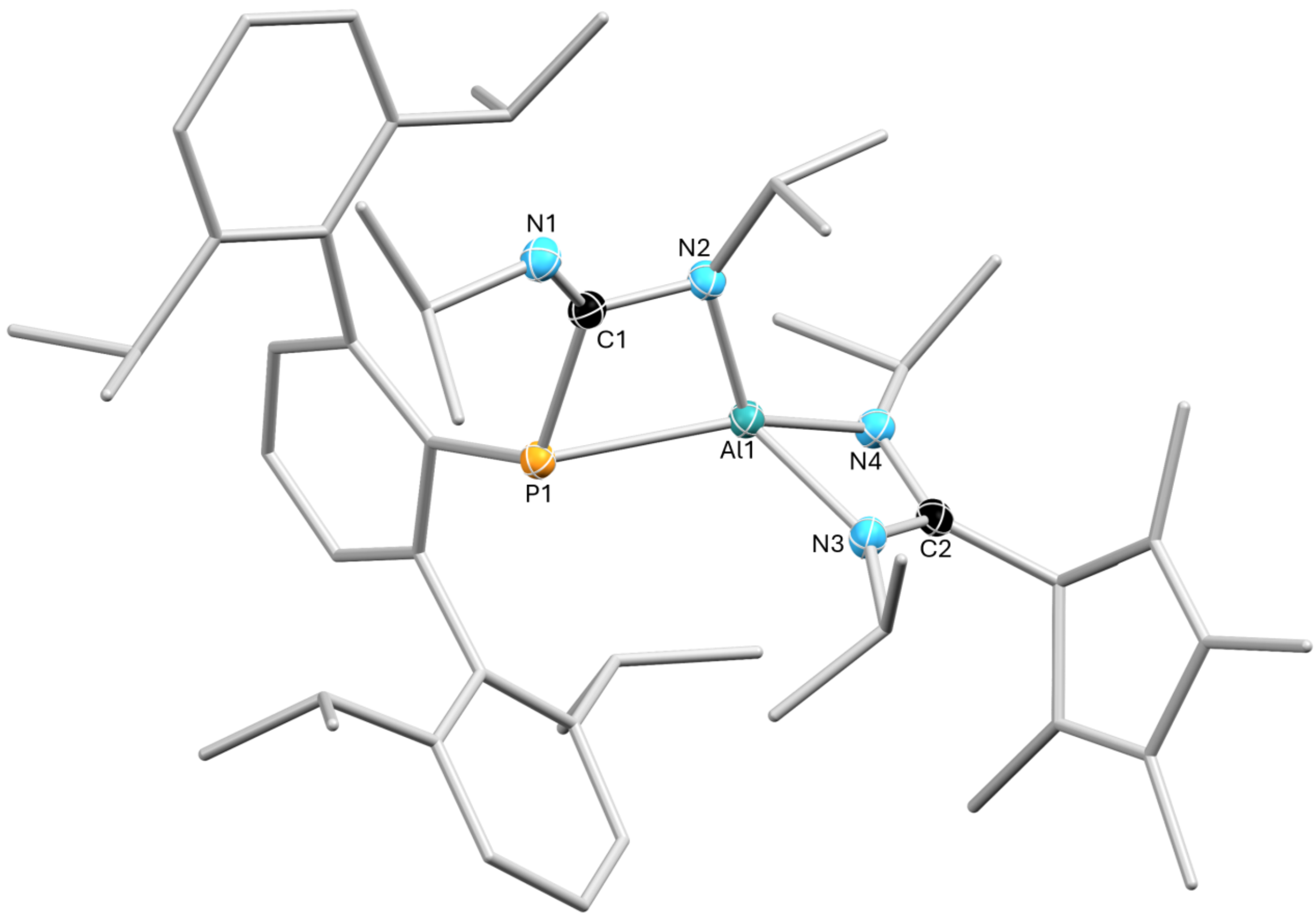

Figure S6: Molecular structure of **6**. Ellipsoids are drawn at 50% probability (100(2) K) with H-atoms and two *n*-pentane molecules omitted. Only one molecule of the two identical molecules in the asymmetric unit is shown. Carbon atoms, except C1 and C2, rendered as wireframe for clarity. Selected bond lengths [Å] and angles [°]: P1–C1 1.765(3), Al1–N1 1.815(3), Al1–N2 1.845(3), Al1–N3 1.882(3), Al1–N4 1.891(3), Al1–C1 2.307(3), Al1–C2 2.332(3), N1–C1 1.386(4), C1–N2 1.405(4), C2–N4 1.335(4), C2–N3 1.353(4); C(Ter)–P1–C1 106.65(15), N1–Al1–N2 74.11(12), N1–Al1–N3 135.72(13), N2–Al1–N3 132.06(13), N1–Al1–N4 123.71(14), N2–Al1–N4 130.97(13), N3–Al1–N4 70.38(12), N1–C1–N2 104.4(3), N4–C2–N3 108.0(3), N2–Al1–N1–C1 6.44(19), N4–C2–N3–Al1 0.2(3).

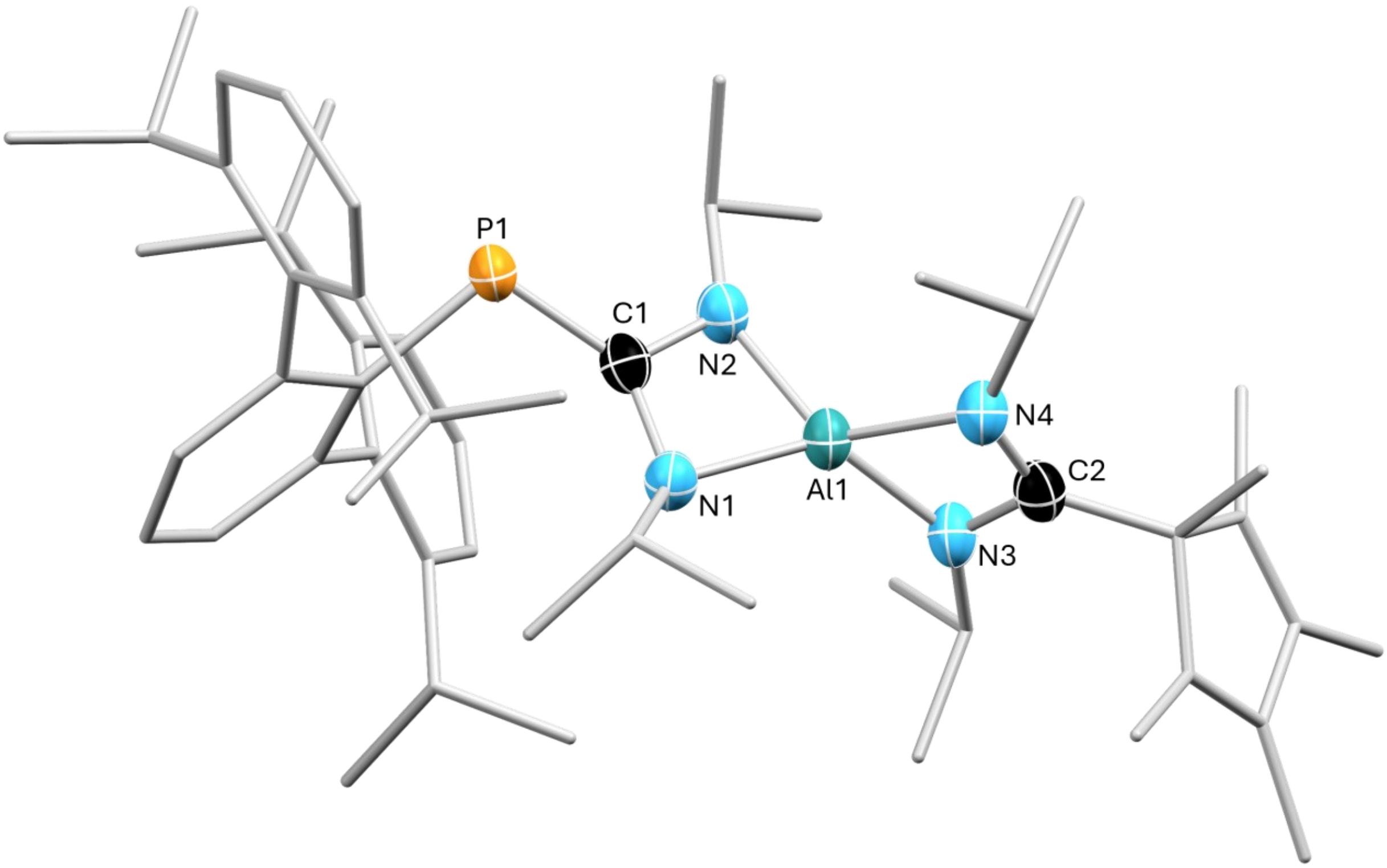

Figure S7: Molecular structure of **7-P**. Ellipsoids are drawn at 50% probability (100(2) K) with H-atoms, except on P1 and N1, and three toluene molecules omitted. Only one molecule of the two identical molecules in the asymmetric unit is shown. Carbon atoms, except C1, rendered as wireframe for clarity. Selected bond lengths [Å] and angles [°]: P1–C1 1.8753(10), N1–C1 1.2776(13), C1–N2 1.3739(13); N1–C1–N2 120.51(9), N1–C1–P1 123.74(8), N2–C1–P1 115.58(7).

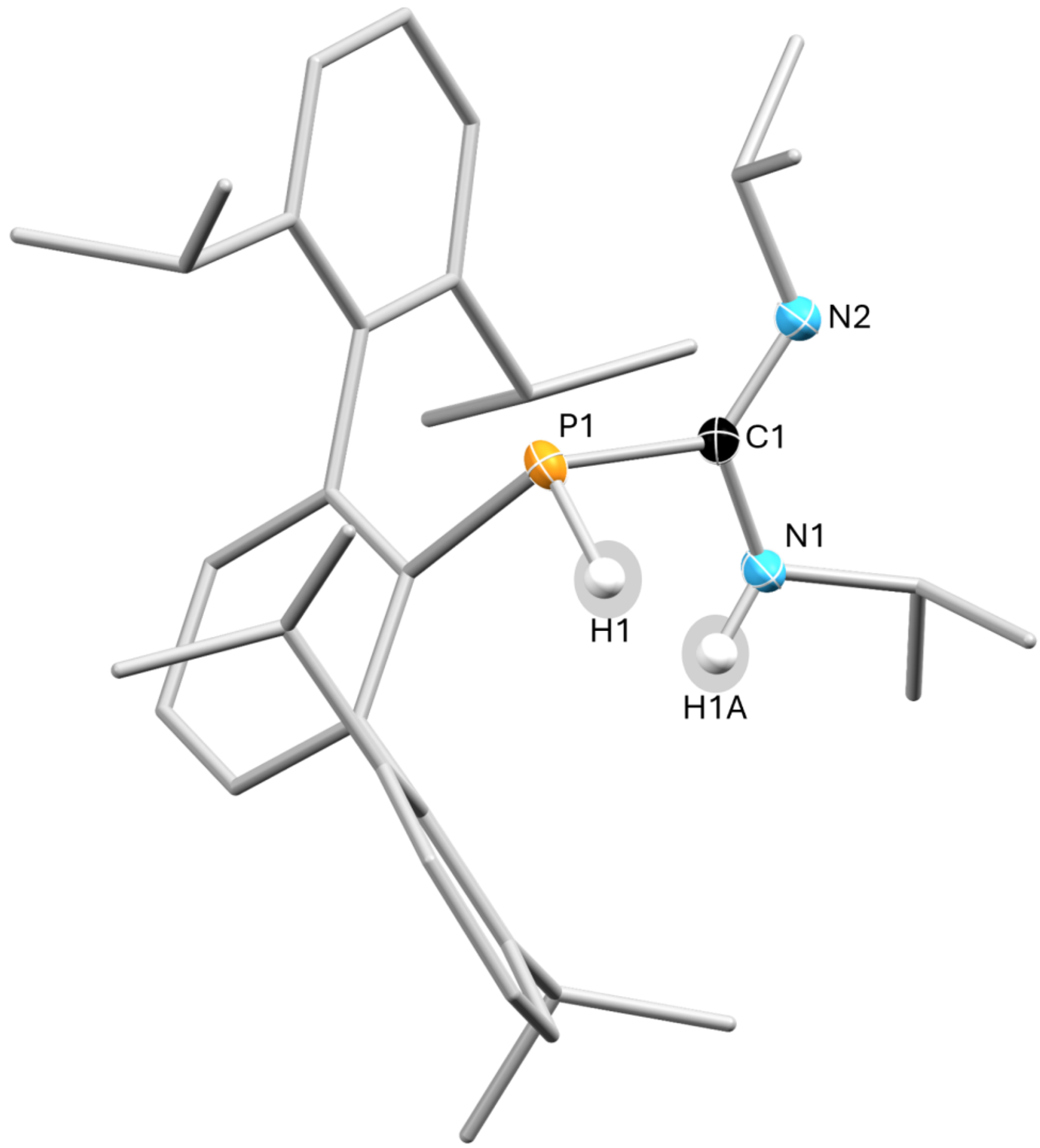

Figure S8: Molecular structure of **8**. Ellipsoids are drawn at 50% probability (100(2) K) with H-atoms, except on P1 and N2, and toluene molecule omitted. Carbon atoms, except C1, rendered as wireframe for clarity. Selected bond lengths [Å] and angles [°]: P1–C1 1.8825(11), N1–C1 1.2797(14), C1–N2 1.3702(13); N1–C1–N2 120.79(9), N1–C1–P1 123.16(8), N2–C1–P1 115.87(7).

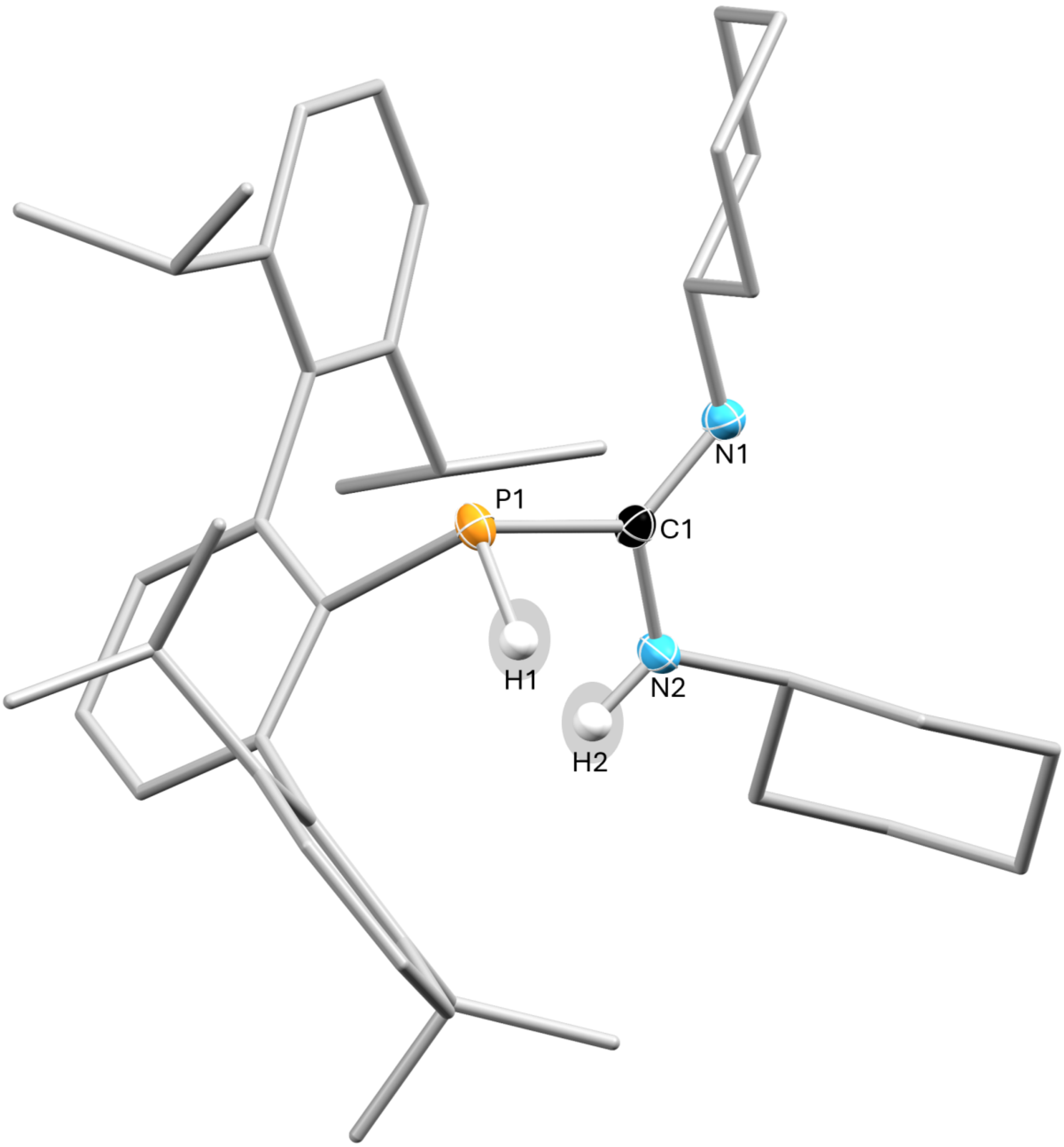

3. Syntheses of compounds

3.1. Improved *in-situ* generation of ^{Dip}TerPCO **2**

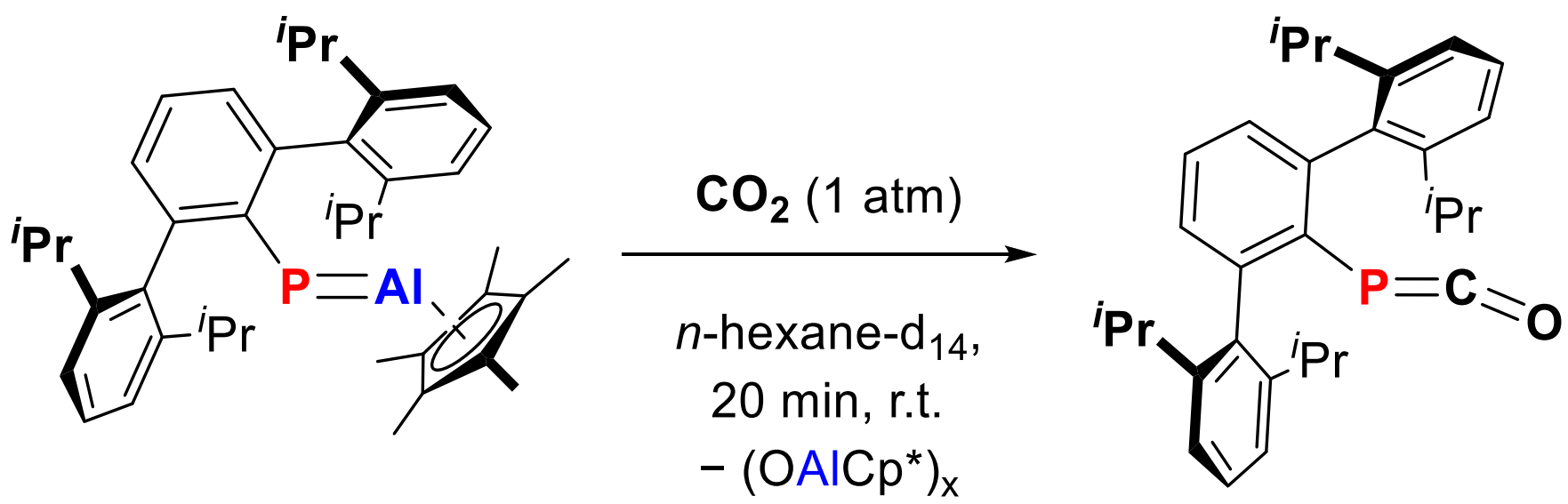

^{Dip}TerPAlCp* (20.0 mg, 33.9 µmol, 1.00 eq.) was dissolved in *n*-hexane (0.4 mL) in a *J.-Young* NMR tube. The purple solution was degassed, and CO_2 (1 atm) was added at ambient temperature. After 20 min the formation of a cloudy orange solution was observed. The solution was filtered over a pipette equipped with a glass microfiber pad to remove precipitated [Cp*AlO]$_X$. The clear orange solution of **^{Dip}TerPCO** can then be used for follow-up reactions.

We performed the reaction in *n*-hexane-d$_{14}$ (^{1}H-NMR reference in ***Figure S8***) to demonstrate complete conversion using NMR spectroscopy and to compare the results with existing data for compound **2**.[8]

*Note: We recommend concentration adjustment by integration using ^{31}P{^{1}H} NMR spectroscopy for stoichiometric reactions. The reaction can be done in n-pentane or n-hexane. The formation of **3** is favoured with lower CO_2 concentrations in the reaction mixture. In this case, degassing of the reaction mixture and subsequent addition of CO_2 (1 atm) leads to the formation of **^{Dip}TerPCO** without loss of yield.*

Scale-Up:

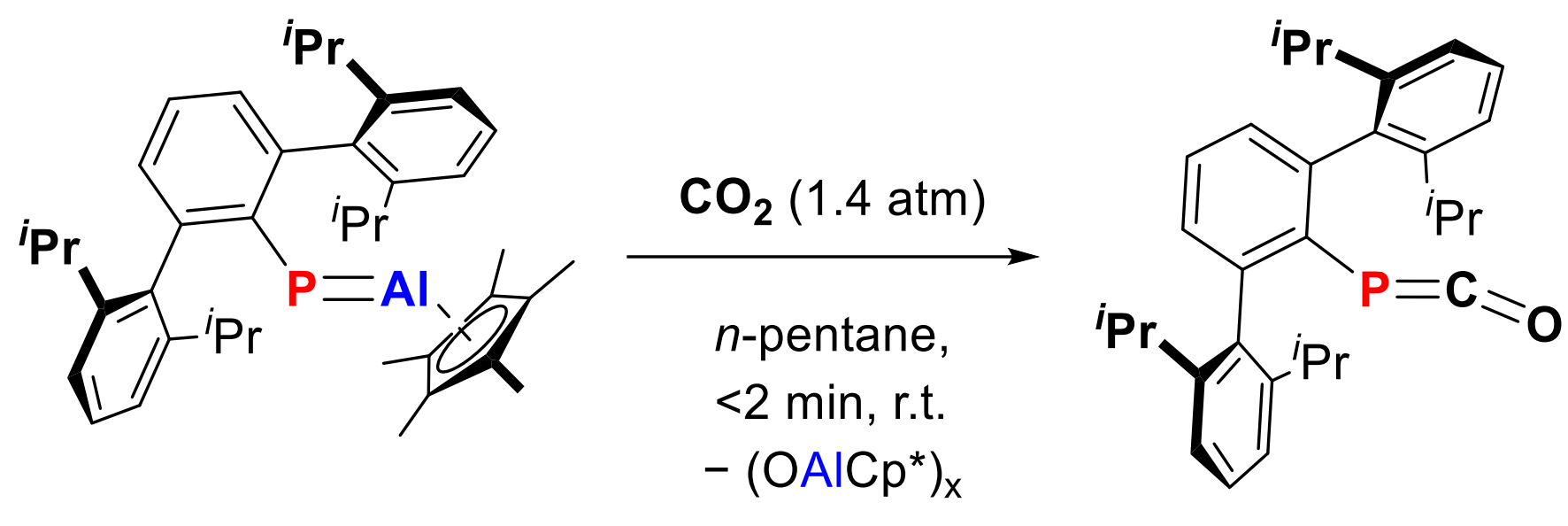

^{Dip}TerPAlCp* (200.0 mg, 0.34 mmol, 1.00 eq.) was dissolved in *n*-pentane (3 mL) and the purple solution was degassed. The Schlenk tube was then connected to a CO_2-line (1.4 atm; CO_2 dried over P_4O_{10}) and CO_2 was added at ambient temperature, resulting in a rapid colour change from purple to orange. After ca. 5 min a colourless precipitate formed, which was removed by canula filtration. An aliquot of the clear orange filtrate was taken for NMR spectroscopy and verified full conversion to **2**. When the solvent is removed from the filtrate *in vacuo* an orange powder is obtained. When this orange powder is re-dissolved in toluene-d_8, it shows that $[Cp^*AlO]_x$ is still present (Figure S10).

*Note: **^{Dip}TerPCO** is stable as orange solid, when the solvent is immediately removed in vacuo (~1×10^{-3} mbar) at ambient temperature. However, when attempting to crystallize **2** the partial formation of a colourless solid can be observed. This way colourless crystals suitable for sc-XRD experiments revealed the formation of the dimer of **^{Dip}TerPCO** (**2dim**).*

*Note: Solutions of freshly prepared **2** in toluene-d_8 are thermally stable at 80 °C in the absence of light, with only minimal decarbonylation observed after heating for 48 h according to ^{31}P NMR spectroscopy. However, when a toluene-d_8 solution of **2** is left in ambient light for the same period of time, full decarbonylation and formation a phosphafluorene is observed,[8] which is a known deactivation product of the phosphinidene ^{Dip}Ter–P (Figure S14).*

^{1}H NMR (500.1 MHz, C_6D_{14}, 298 K): δ = 1.07 (d, $^3J_{(H\text{-}H)}$ = 6.9 Hz, 12 H, CH(CH_3)(CH$_3$)), 1.25 (d, $^3J_{(H\text{-}H)}$ = 6.9 Hz, 12 H, CH(CH$_3$)(CH_3)), 2.64 (hept, $^3J_{(H\text{-}H)}$ = 6.9 Hz, 4 H, CH(CH$_3$)(CH$_3$)), 7.03 (dd,

$^{3}J_{(H\text{-}H)}$ = 7.5 Hz, $^{3}J_{(H\text{-}P)}$ = 2.6 Hz, 2 H, *m-H* (^{Dip}Ter)), 7.15 (d, $^{3}J_{(H\text{-}H)}$ = 7.8 Hz, 4 H, *m-H* (Dip)), 7.19 – 7.23 (m, 1 H, *p-H* (^{Dip}Ter)), 7.32 (t, $^{3}J_{(H\text{-}H)}$ = 7.8 Hz, 2 H, *p-H* (Dip)) ppm.

^{1}H NMR (300.1 MHz, C_7D_8, 298 K): δ = 1.07 (d, $^{3}J_{(H\text{-}H)}$ = 6.8 Hz, 12 H, CH(C*H*$_3$)(CH$_3$)), 1.34 (d, $^{3}J_{(H\text{-}H)}$ = 6.9 Hz, 12 H, CH(CH$_3$)(C*H*$_3$)), 2.75 (hept, $^{3}J_{(H\text{-}H)}$ = 6.9 Hz, 4 H, C*H*(CH$_3$)(CH$_3$)), 7.15 (d, $^{3}J_{(H\text{-}H)}$ = 7.9 Hz, 4 H, *m-H* (Dip)), 7.32 (t, $^{3}J_{(H\text{-}H)}$ = 7.9 Hz, 2 H, *p-H* (Dip)) ppm. *The signals of the aryl protons of the central aryl-ring of the DipTer substituent overlap with the solvent signals. [Cp*AlO]$_x$ is detected as a broad feature between 2.05-1.55 ppm, matching the expected integration of ca. 15 H.

^{13}C{^{1}H} NMR (125.7 MHz, C_6D_{14}, 298 K): δ = 23.0 (d, $J_{(C\text{-}P)}$ = 2 Hz, CH(CH$_3$)(*C*H$_3$)), 24.3 (s, CH(*C*H$_3$)(CH$_3$)), 30.5 (s, *C*H(CH$_3$)(CH$_3$)), 122.8 (s, *m-C*H (Dip)), 125.5 (d, $J_{(C\text{-}P)}$ = 1 Hz, *p-C*H (^{Dip}Ter)), 128.2 (d, $J_{(C\text{-}P)}$ = 4 Hz, *m-C*H (^{Dip}Ter)), 129.4 (s, *p-C*H (Dip)), 133.2 (d, $^{1}J_{(C\text{-}P)}$ = 37 Hz, *i-C* (^{Dip}Ter)), 139.8 (d, $J_{(C\text{-}P)}$ = 6 Hz, *i-C* (Dip)), 144.3 (d, $J_{(C\text{-}P)}$ = 18.0 Hz, *o-C* (^{Dip}Ter)), 147.1 (d, $J_{(C\text{-}P)}$ = 1 Hz, *o-C* (Dip)), 198.7 (d, $^{1}J_{(C\text{-}P)}$ = 104 Hz, P*C*O) ppm.

^{31}P{^{1}H} NMR (202.4 MHz, C_6D_{14}, 298 K): δ = −201.5 (s) ppm.

^{31}P{^{1}H} NMR (202.4 MHz, C_7D_8, 298 K): δ = −201.3 (s) ppm.

IR (ATR, 24 scans, cm^{-1}): $\tilde{\nu}$ = 3057 (w), 2959 (s), 2924 (m), 2866 (m), 1944 (s, CO), 1673 (w), 1560 (m), 1460 (m), 1442 (m), 1383 (m), 1361 (m), 1327 (w), 1250 (w), 1225 (w), 1057 (w), 804 (m), 790 (m), 757 (m), 661 (w), 606 (w), 585 (m), 548 (w), 511 (w), 460 (w).

LIFDI-MS: (m/z) Calc: 456.2582, 457.2616, 458.2649 [$C_{31}H_{37}PO$]; Found: 428.2627, 429.2661, 430.2694 [M−CO].

Figure S9: ^{1}H NMR spectrum of hexane-d_{14} with tetramethylsilane as reference (400.6 MHz, 298 K).

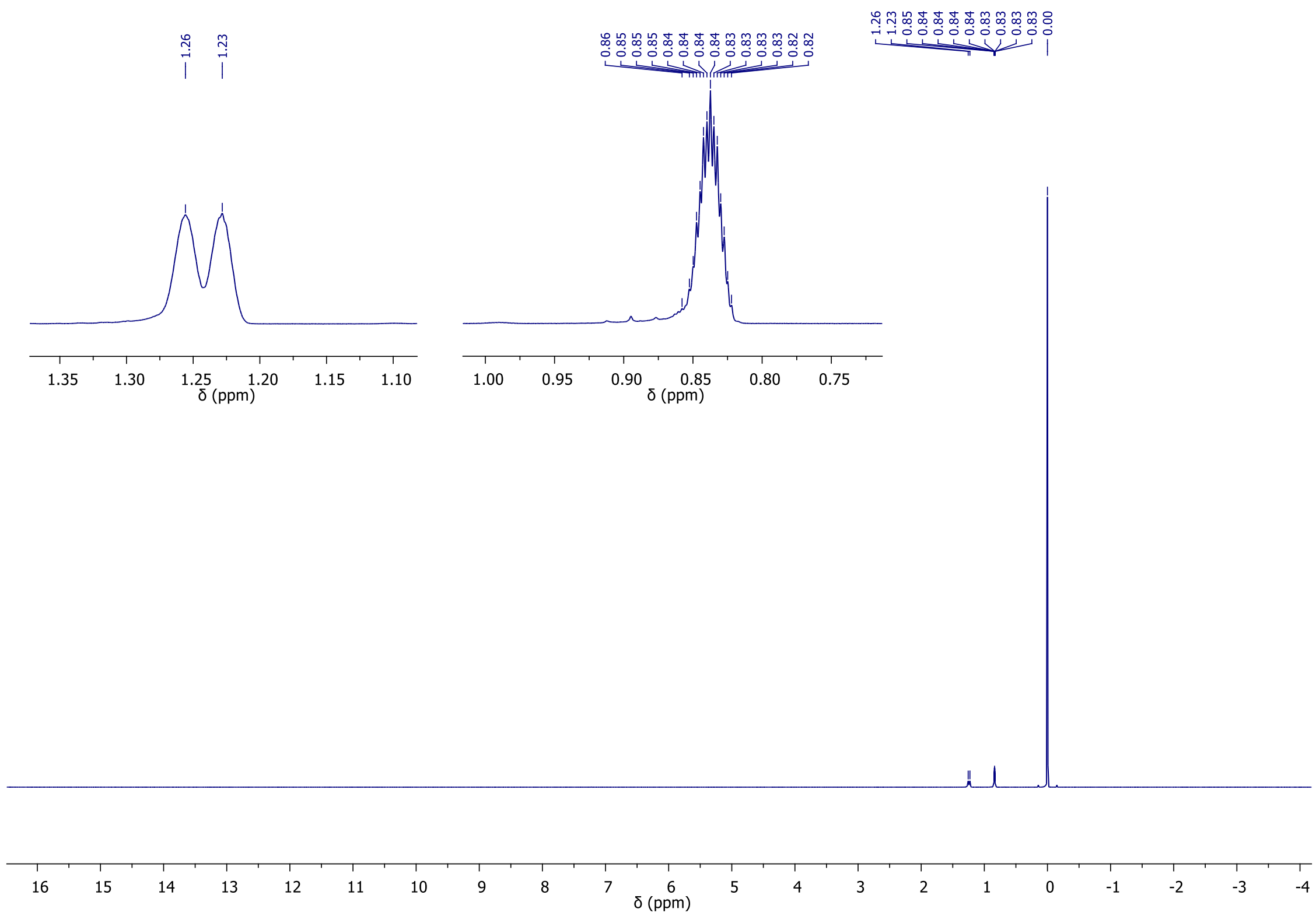

Figure S10: ^{1}H NMR spectrum of **2** (500.1 MHz, C_6D_{14}, 298 K).

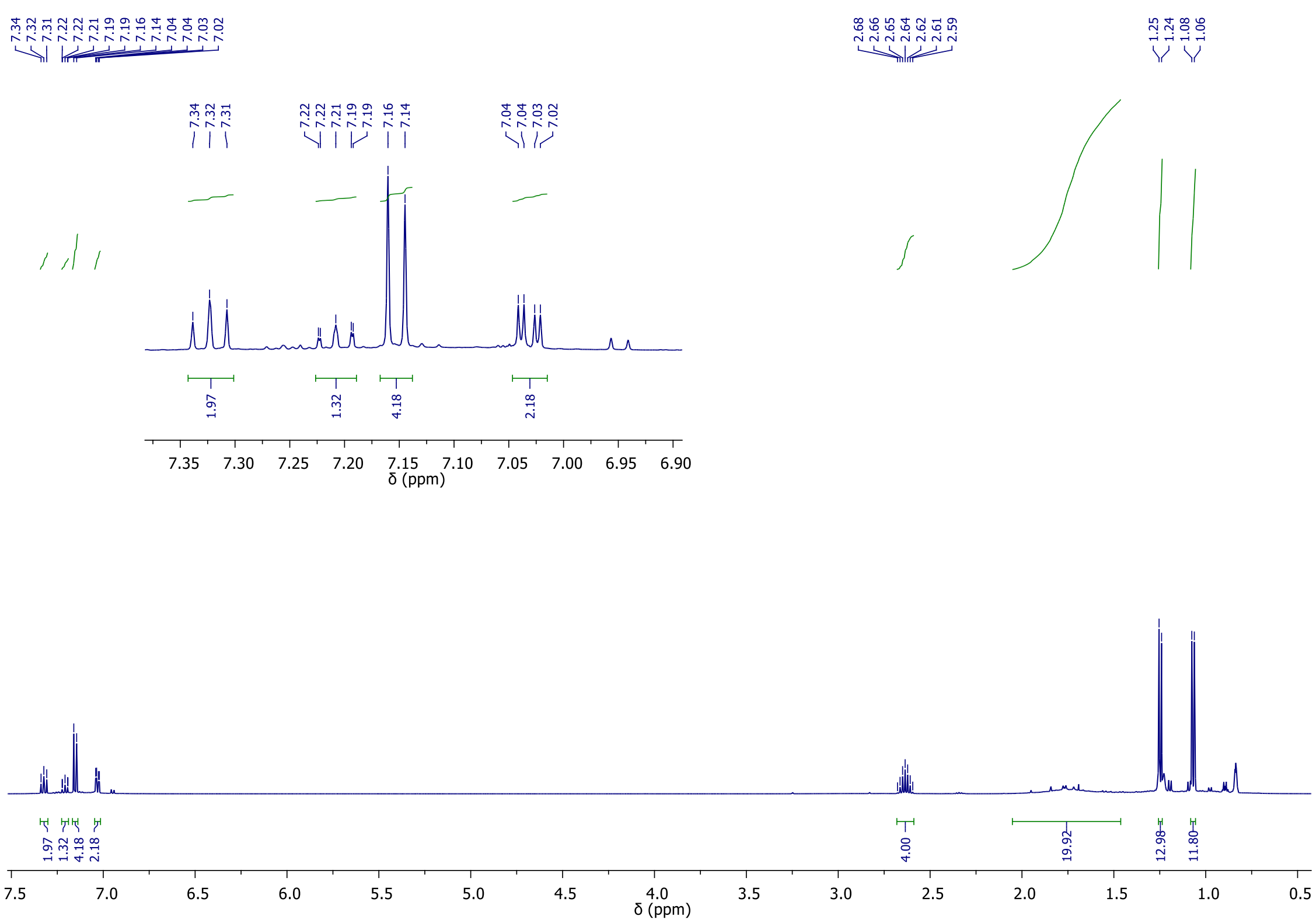

Figure S11: ^{1}H NMR spectrum of **2** (300.1 MHz, Tol-d_8, 298 K).

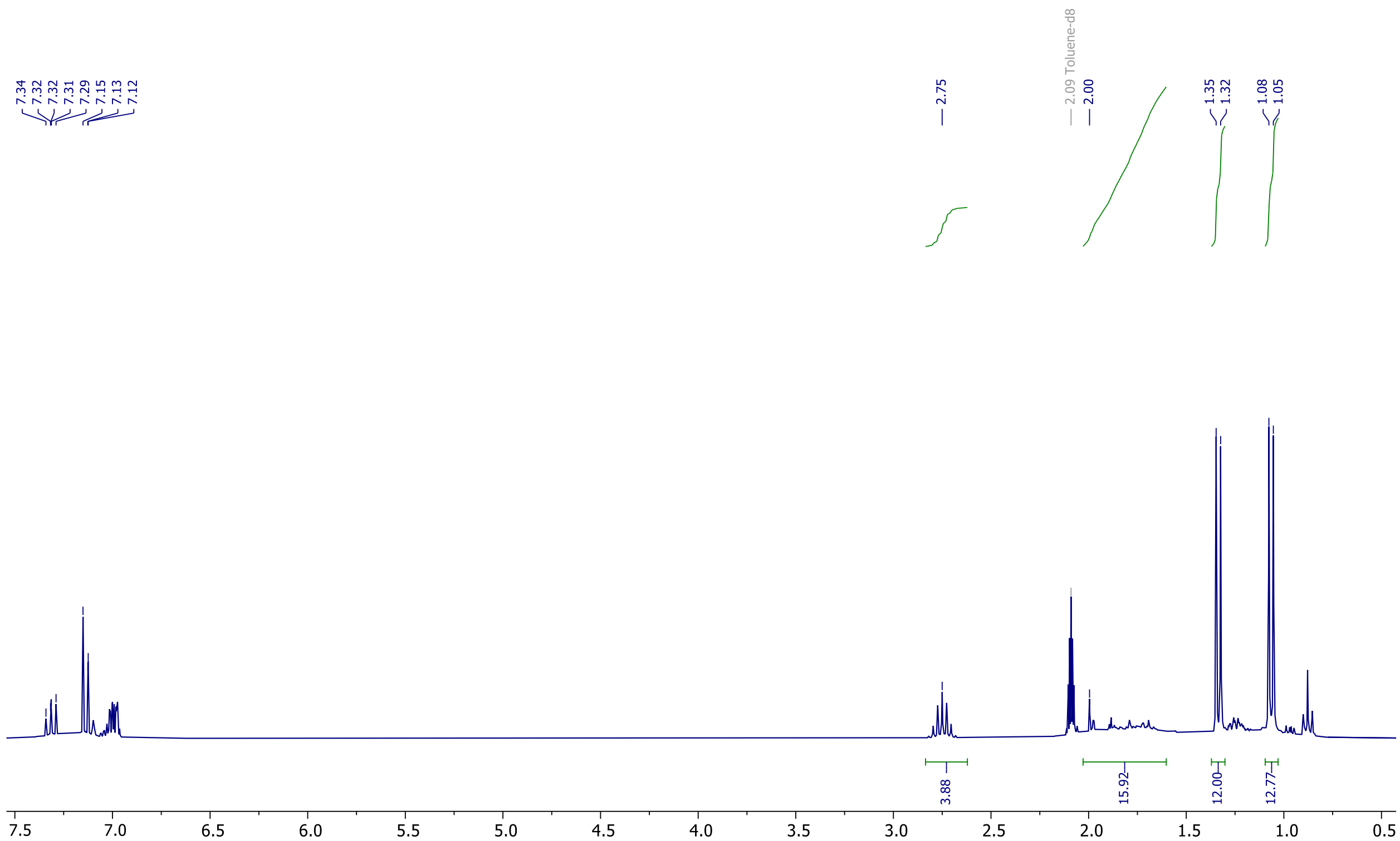

Figure S12: ^{13}C{^{1}H} NMR spectrum of **2** (125.7 MHz, C_6D_{14}, 298 K).

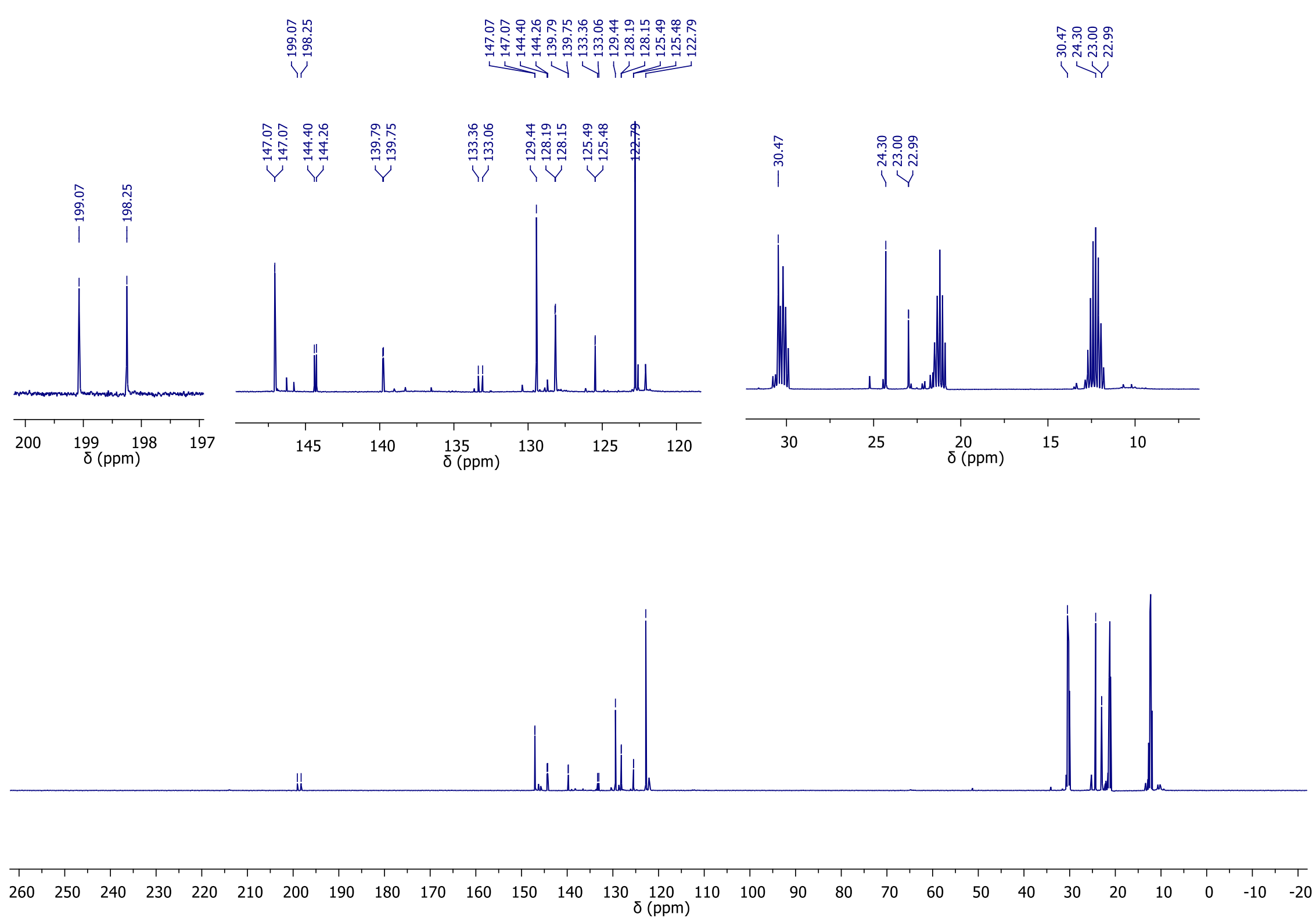

Figure S13: $^{31}P\{^{1}H\}$ NMR spectrum of **2** (202.4 MHz, C_6D_{14}, 298 K); −142.0 ppm $^{Dip}TerPH_2$.

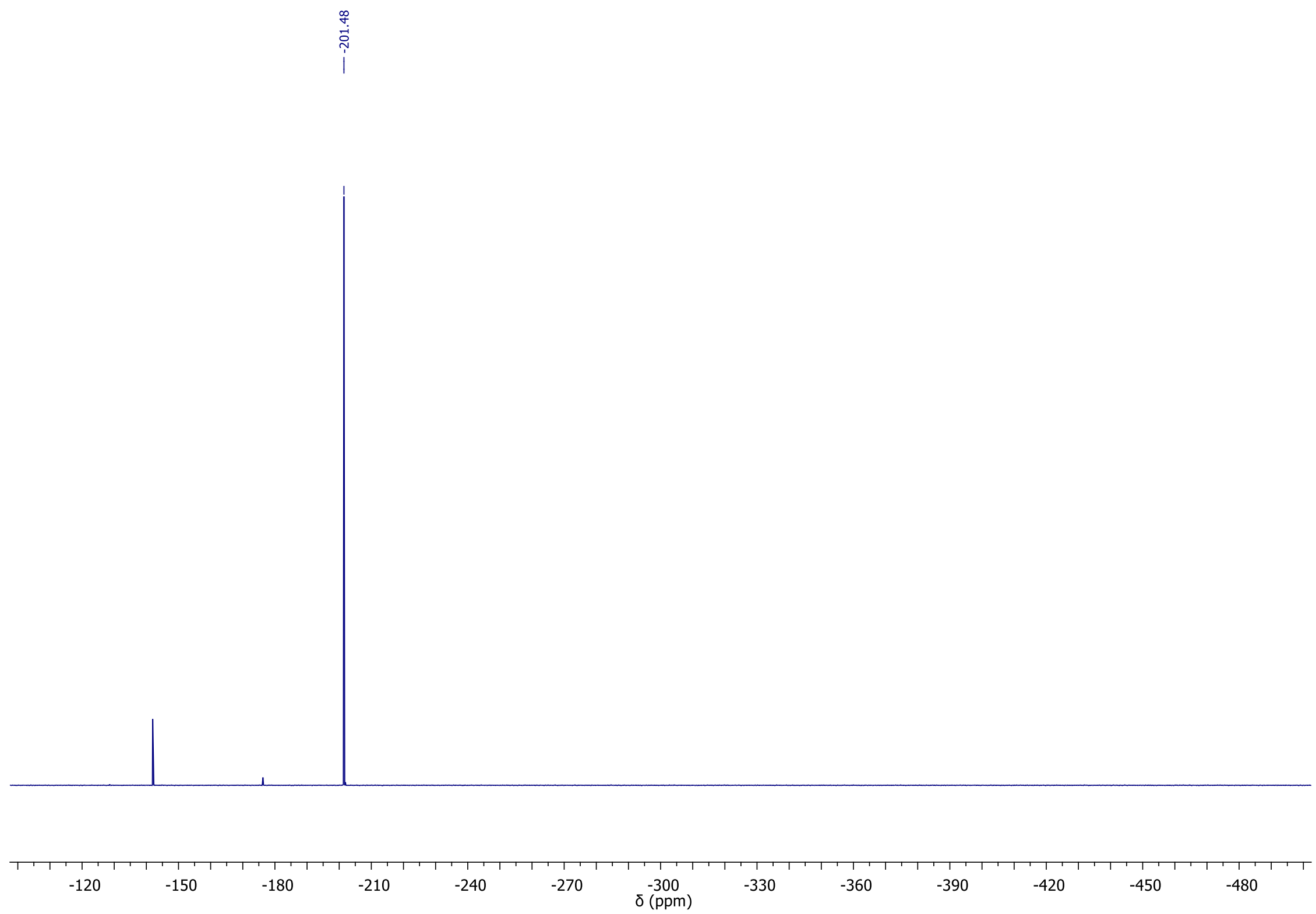

Figure S14: $^{31}P\{^{1}H\}$ NMR spectrum of **2** (121.5 MHz, Tol-d_8, 298 K); −140.7 ppm $^{Dip}TerPH_2$.

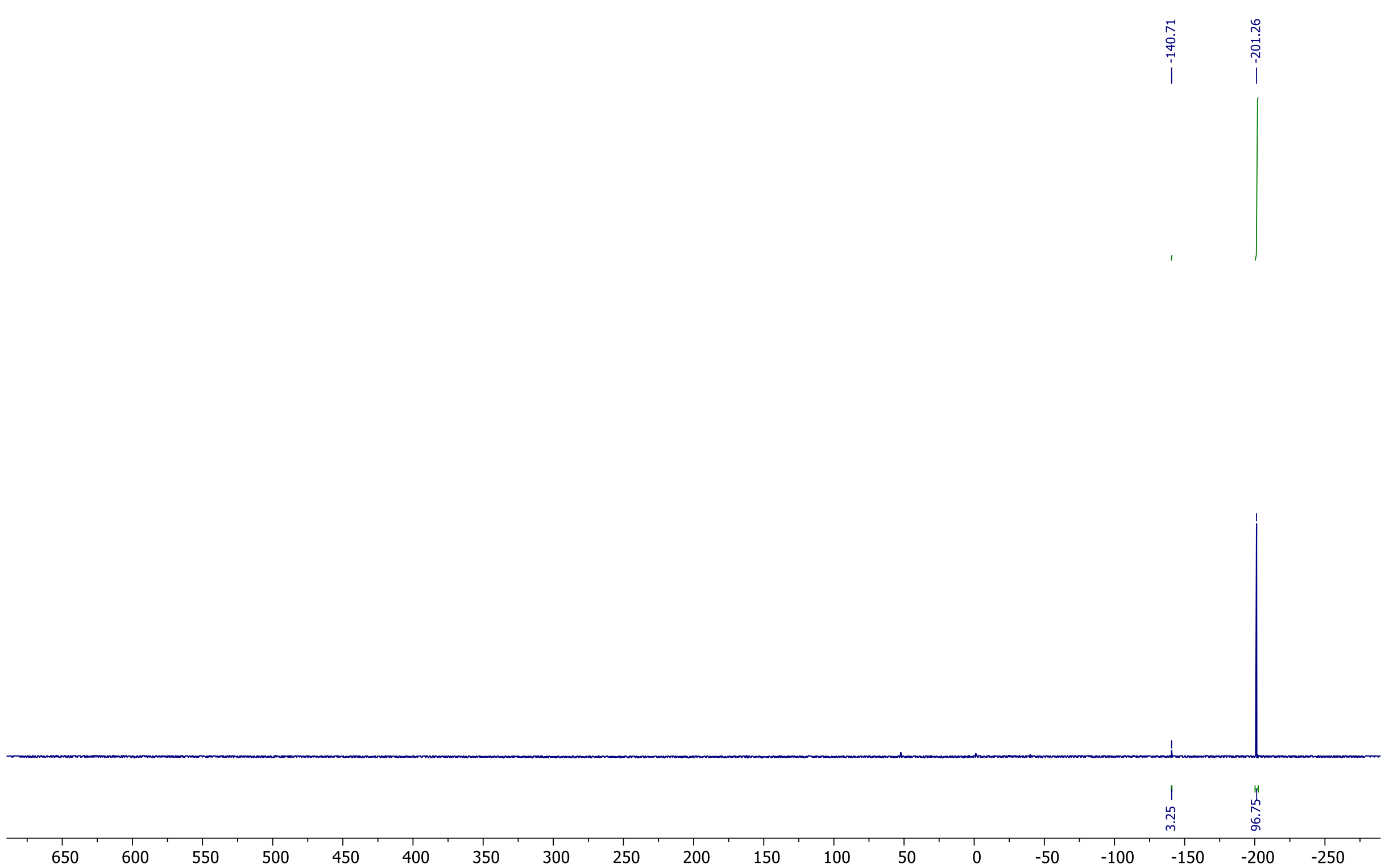

Figure S15: $^{31}P\{^1H\}$ NMR spectra of **2**: After 48 h in sunlight at ambient temperature (top); after 48 h at 80 °C in the dark (121.5 MHz, Tol-d_8, 298 K); −1 ppm phosphafluorene (light blue box), −201 ppm ^{Dip}TerPCO (light red box).

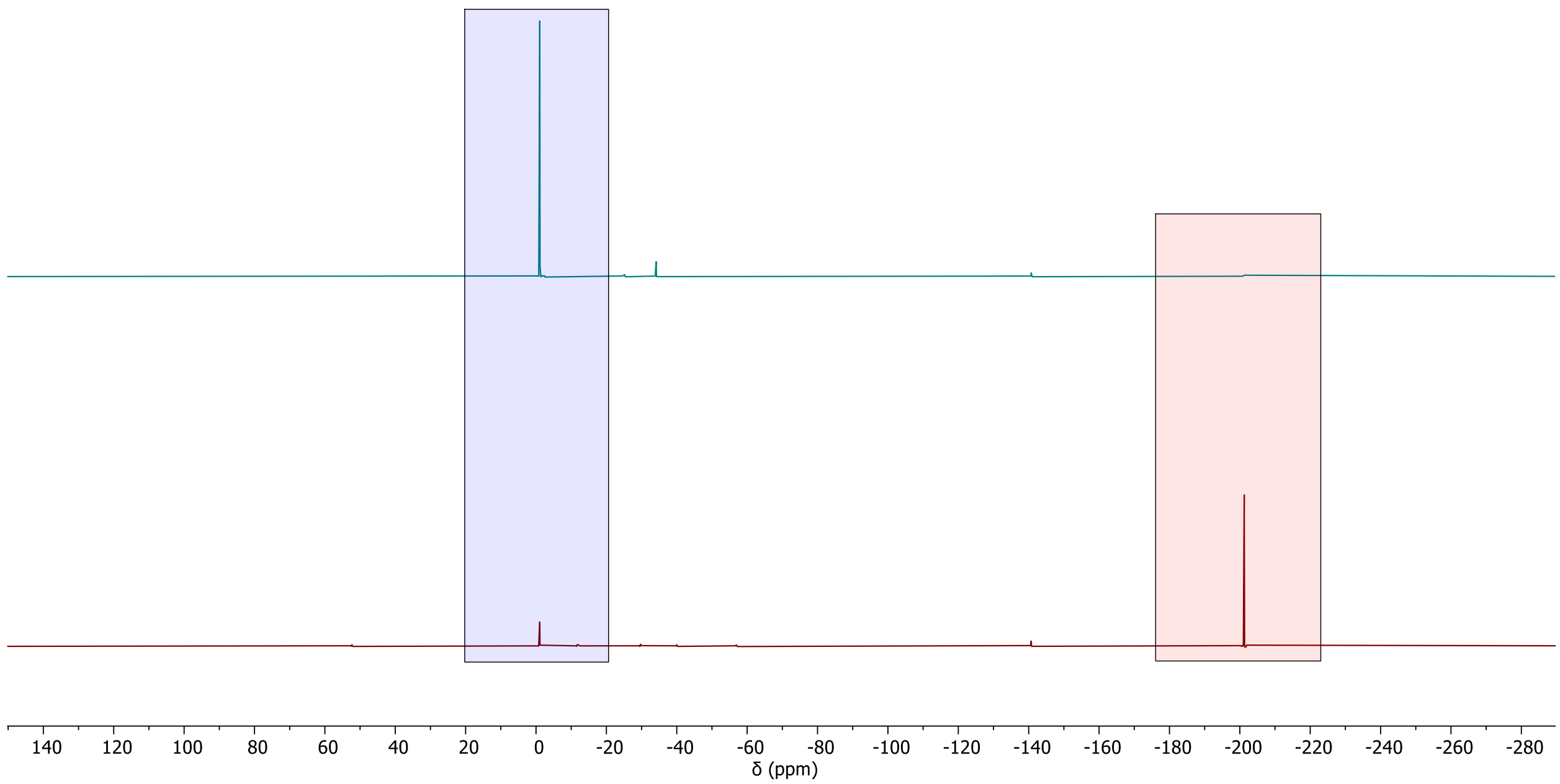

Figure S16: IR spectrum of **2** (ATR, 64 scans, cm^{-1}).

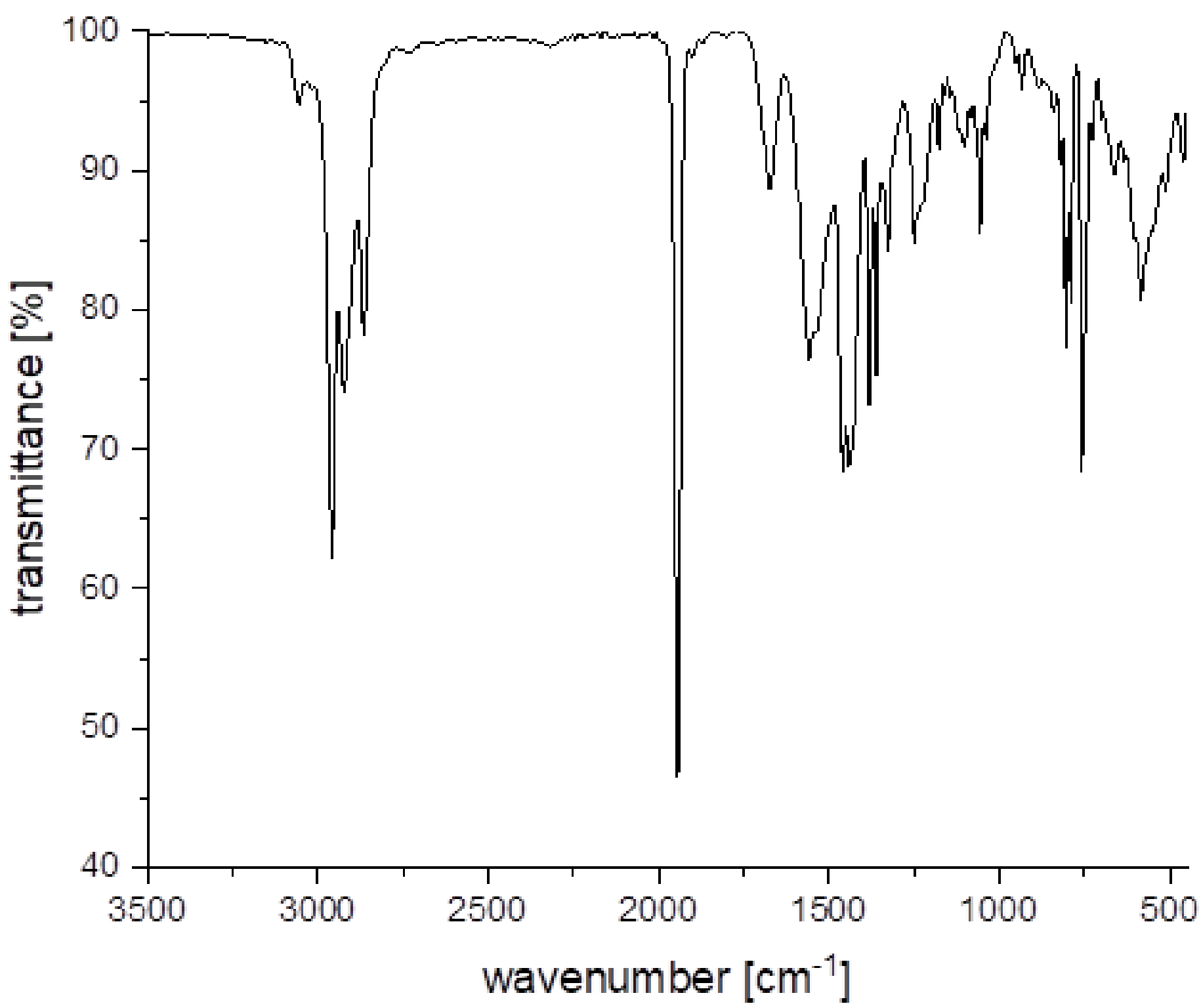

Figure S17: LIFDI mass spectrum of **2** (toluene).

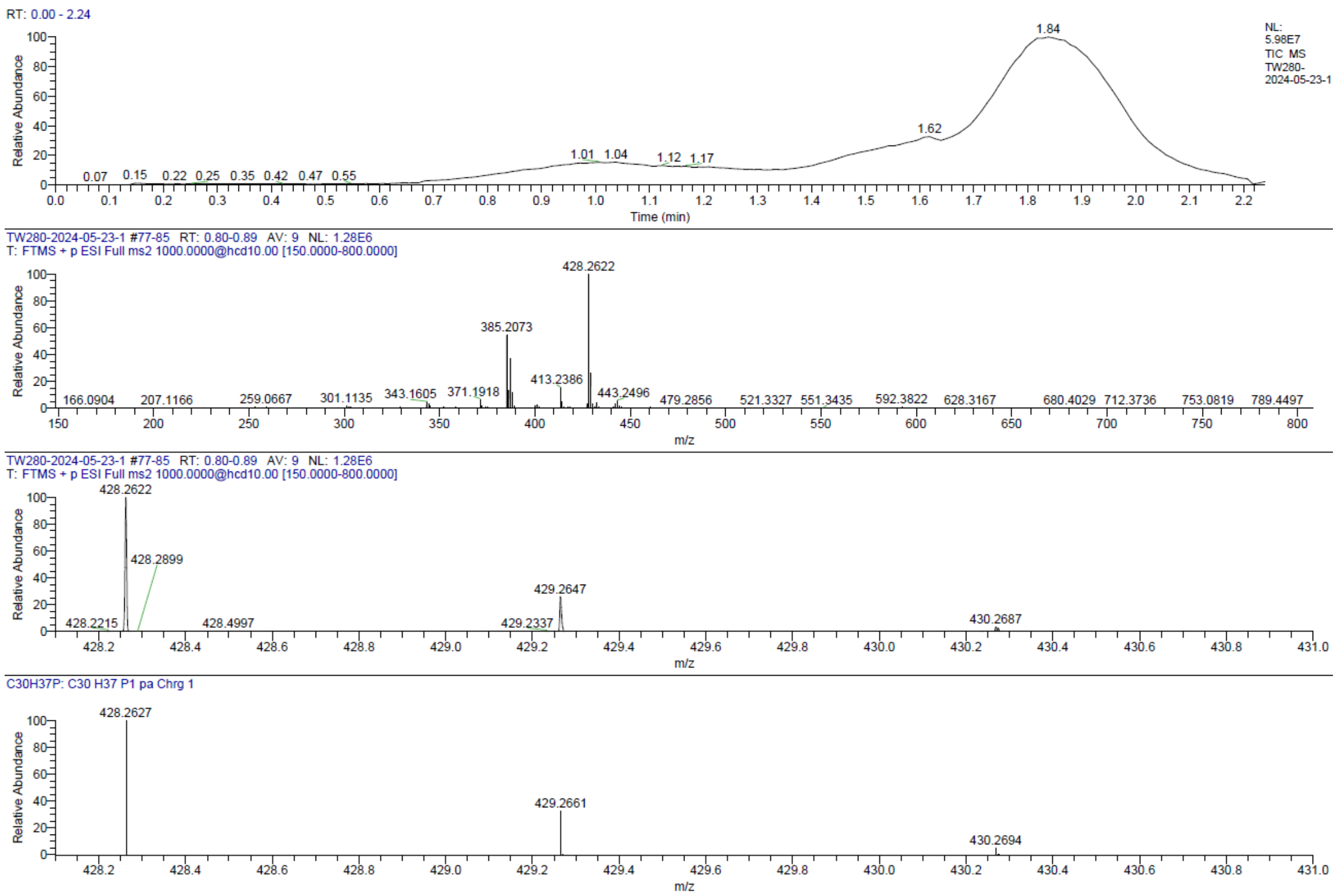

3.2. Synthesis of **3**

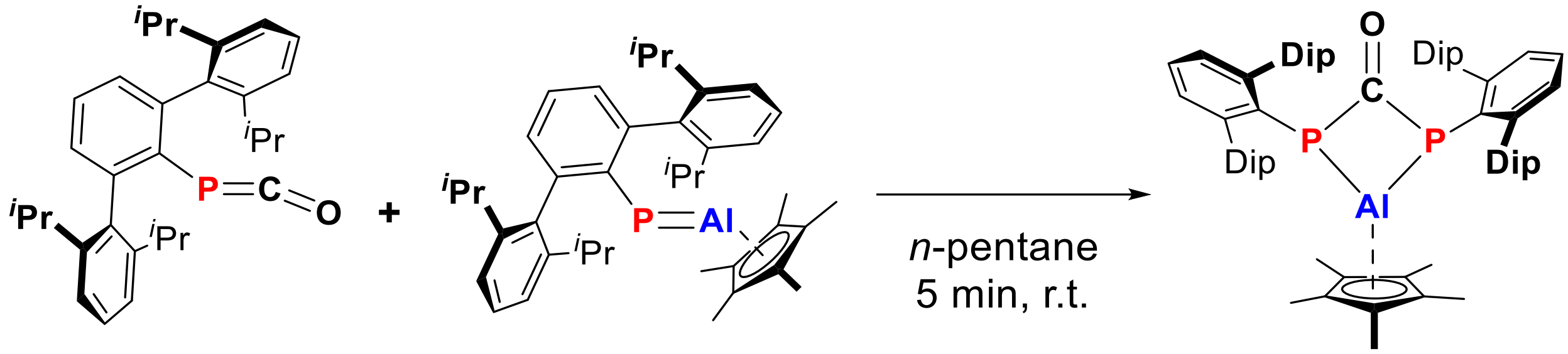

2 **1** **3** (63%)

^{Dip}**TerPAlCp*** (85 mg, 144 μmol, 1.00 eq.) was dissolved in *n*-pentane (3 mL) in a Schlenk tube. The purple solution was degassed and CO_2 (1.4 atm) was added at ambient temperature. After 2 min the formation of a cloudy orange solution was observed. The solution was degassed to remove excess of CO_2. Full conversion of ^{Dip}**TerPAlCp*** to ^{Dip}**TerPCO** was confirmed via $^{31}P\{^1H\}$ NMR spectroscopy. During the synthesis of ^{Dipp}**TerPCO**, ^{Dip}TerPH$_2$ is formed by traces of moisture and further work is carried out with the reduced amount of ^{Dip}**TerPCO** (47.7 μmol) determined by integration in the $^{31}P\{^1H\}$ NMR spectrum. Subsequently, ^{Dip}**TerPAlCp*** (81 mg, 137 μmol, 0.95 eq.) was dissolved in pentane (3 mL) and was added dropwise to the orange suspension of **2** at ambient temperature. Upon addition of ^{Dip}**TerPAlCp*** the violet colour of the phosphaalumene quickly vanished and after 5 min the formation of a pale-yellow solution was observed. After stirring for 10 minutes a yellowish solid crashed out, which was identified as microcrystalline **3** (1st fraction). The solid was removed by canula filtration, and suitable crystals for single crystal X-ray diffraction and for full characterization of **3** were obtained by storing the supernatent solution at –30 °C for 24 h (2nd fraction). The pale yellow crystals were washed with cooled *n*-pentane (–30 °C) and dried in vacuo (10^{-3} mbar) for 30 minutes.

Yield of 3 (combined fractions): 96 mg (92 μmol, 63%) as colourless crystals.

Note: While recording the NMR spectra and repeated attempts of dissolving crystalline material of ***3*** *the presence of free* ***2*** *was observed. When heating a* C_6D_6 *solution of isolated*

*crystals of **3** at 80 °C for 16 h, the formation of a yet unidentified product was observed (Figure S22).*

^{1}H NMR (500.1 MHz, C_6D_6, 298 K): δ = 0.58 (d, $^3J_{(H\text{-}H)}$ = 6.6 Hz, 6 H, CH(C*H*$_3$)(CH$_3$) (group A))[1], 0.82 (d, $^3J_{(H\text{-}H)}$ = 6.8 Hz, 6 H, CH(C*H*$_3$)(CH$_3$) (group B)), 0.95 (d, $^3J_{(H\text{-}H)}$ = 6.8 Hz, 6 H, CH(C*H*$_3$)(CH$_3$) (group C)), 1.07 (d, $^3J_{(H\text{-}H)}$ = 6.6 Hz, 6 H, CH(CH$_3$)(C*H*$_3$) (group A)), 1.17 (d, $^3J_{(H\text{-}H)}$ = 6.8 Hz, 6 H, CH(CH$_3$)(C*H*$_3$) (group C)), 1.22 (d, $^3J_{(H\text{-}H)}$ = 6.7 Hz, 6 H, CH(C*H*$_3$)(CH$_3$) (group D)), 1.38 (d, $^3J_{(H\text{-}H)}$ = 6.8 Hz, 6 H, CH(CH$_3$)(C*H*$_3$) (group B)), 1.40 (s, 15 H, C$_5$(C*H*$_3$)$_5$), 1.44 (d, $^3J_{(H\text{-}H)}$ = 6.7 Hz, 6 H, CH(CH$_3$)(C*H*$_3$) (group D)), 2.14 (hept, $^3J_{(H\text{-}H)}$ = 6.8 Hz, 2 H, C*H*(CH$_3$)(CH$_3$) (group C)), 2.64 (hept, $^3J_{(H\text{-}H)}$ = 6.6 Hz, 2 H, C*H*(CH$_3$)(CH$_3$) (group A)), 3.04 (hept, $^3J_{(H\text{-}H)}$ = 6.8 Hz, 2 H, C*H*(CH$_3$)(CH$_3$) (group B)), 3.99 (hept, $^3J_{(H\text{-}H)}$ = 6.7 Hz, 6 H, C*H*(CH$_3$)(CH$_3$) (group D)), 6.67 – 6.71 (m, 2 H, aryl-*H*), 6.80 – 6.85 (m, 2 H, aryl-*H*), 7.06 – 7.09 (m, 3 H, aryl-*H*), 7.17 – 7.21 (m, 3 H, aryl-*H*), 7.24 – 7.26 (m, 3 H, aryl-*H*), 7.31 – 7.35 (m, 2 H, aryl-*H*), 7.35 – 7.39 (m, 2 H, aryl-*H*) ppm.

^{13}C{^{1}H} NMR (125.8 MHz, C_6D_6, 298 K): δ = 10.1 (s, C$_5$(*C*H$_3$)$_5$), 23.0 (s, CH(CH$_3$)(*C*H$_3$) (group B)), 23.7 (s, superimposed signal, CH(CH$_3$)(*C*H$_3$) (group C) + CH(*C*H$_3$)(CH$_3$) (group A)), 24.5 (s, CH(*C*H$_3$)(CH$_3$) (group B)), 25.4 (s, CH(*C*H$_3$)(CH$_3$) (group D)), 25.6 (s, CH(CH$_3$)(*C*H$_3$) (group A)), 25.9 (s, CH(*C*H$_3$)(CH$_3$) (group C)), 27.3 (s, CH(CH$_3$)(*C*H$_3$) (group D)), 30.2 (s, *C*H(CH$_3$)(CH$_3$) (group C)), 30.6 (s, *C*H(CH$_3$)(CH$_3$) (group B)), 31.0 (s, *C*H(CH$_3$)(CH$_3$) (group A)), 31.30 (s, *C*H(CH$_3$)(CH$_3$) (group D)), 122.1 (s, *m*-*C*H (Dip) (group F)),123.4 (s, *m*-*C*H (Dip) (group E)), 125.0 (s, *m*-*C*H (Dip) (group F)), 126.7 (s, *m*-*C*H (^{Dip}Ter)), 128.8 (s, *p*-*C*H)[2], 131.2 (s, *p*-*C*H), 131.9 (s, *p*-*C*H), 136.5 (t, $^1J_{(C\text{-}P)}$ = 25.4 Hz, *i*-*C* (^{Dip}Ter)), 142.4 (s, *i*-*C* (Dip) (group E)), 142.7 (s, *i*-*C* (Dip) (group F)), 143.2 (s, *o*-*C* (^{Dip}Ter)), 145.8 (s, *o*-*C* (Dip) (group F)), 148.2 (s, *o*-*C* (Dip) (group E)), 149.6 (s, *o*-*C* (Dip) (group F)), 217.4 (t, $^1J_{(C\text{-}P)}$ = 36 Hz, P$_2$*C*O) ppm.

[1] Due to the similarity of the ligands, groups have been used in this case to make the assignment easier to understand. The groups A to D are used to assign the signals of the isopropyl groups. The groups E and F are used to assign the Dip groups. The isopropyl groups A and C belongs to the Dip group E and the isopropyl groups B and D belongs to the Dip group F.
[2] Superimposed with C_6D_6 signal. Assigned with ^{1}H/^{13}C HSQC and ^{1}H/^{13}C HMBC nmr.

^{31}**P{^{1}H} NMR** (202.5 MHz, C_6D_6, 298 K): δ = −27.0 (s) ppm.

LIFDI-MS: (m/z) Calc: 1046.6204 [$C_{71}H_{89}AlOP_2$]; Found: 428.2627 [^{Dip}TerP], 441.2699 [^{Dip}TerPCH]$^{+}$, 895.5851 [M−Cp*−O].

IR (ATR, 24 scans, cm^{-1}): $\tilde{v}$ = 3055 (w), 2959 (m), 2921 (m), 2863 (m), 1559 (w, C=O), 1459 (m), 1441 (m), 1426 (m), 1379 (m), 1359 (m), 1323 (w), 1250 (m), 1054 (m), 1038 (w), 844 (m), 804 (s), 792 (m), 761 (s), 745 (s), 556 (m), 538 (s), 469 (w), 419 (s).

Figure S18: ^{1}H NMR spectrum of **3** (500.1 MHz, C_6D_6, 298 K); 0.87, 1.23 ppm *n*-pentane.

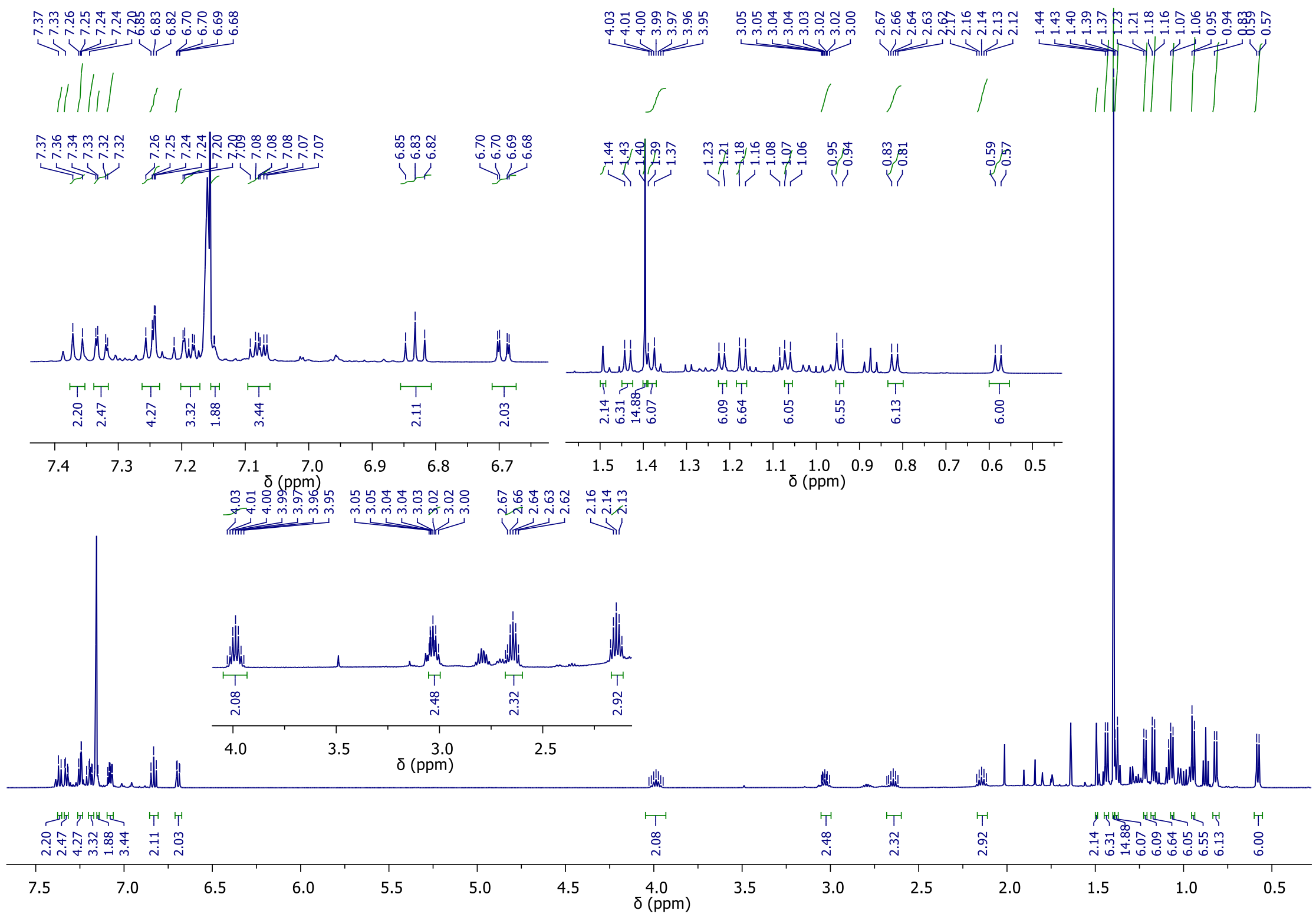

Figure S19: ^{13}C{^{1}H} NMR spectrum of **3** (125.8 MHz, C_6D_6, 298 K); 14.3, 22.7, 34.4 ppm *n*-pentane.

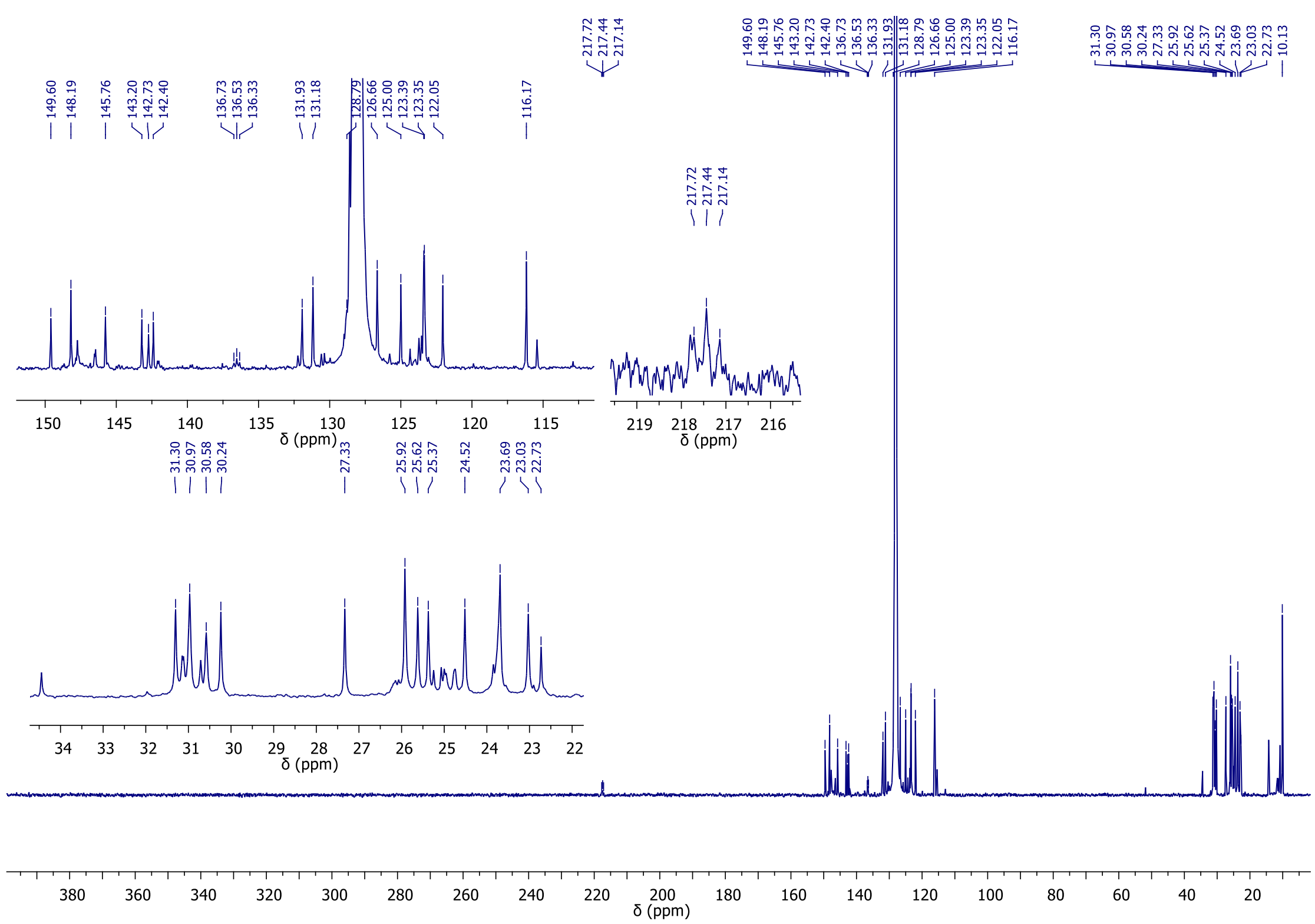

Figure S20: $^{31}P\{^1H\}$ NMR spectrum of **3** (202.5 MHz, C_6D_6, 298 K), −141.5 ppm $^{Dip}TerPH_2$, −201.3 ppm **^{Dip}TerPCO**.

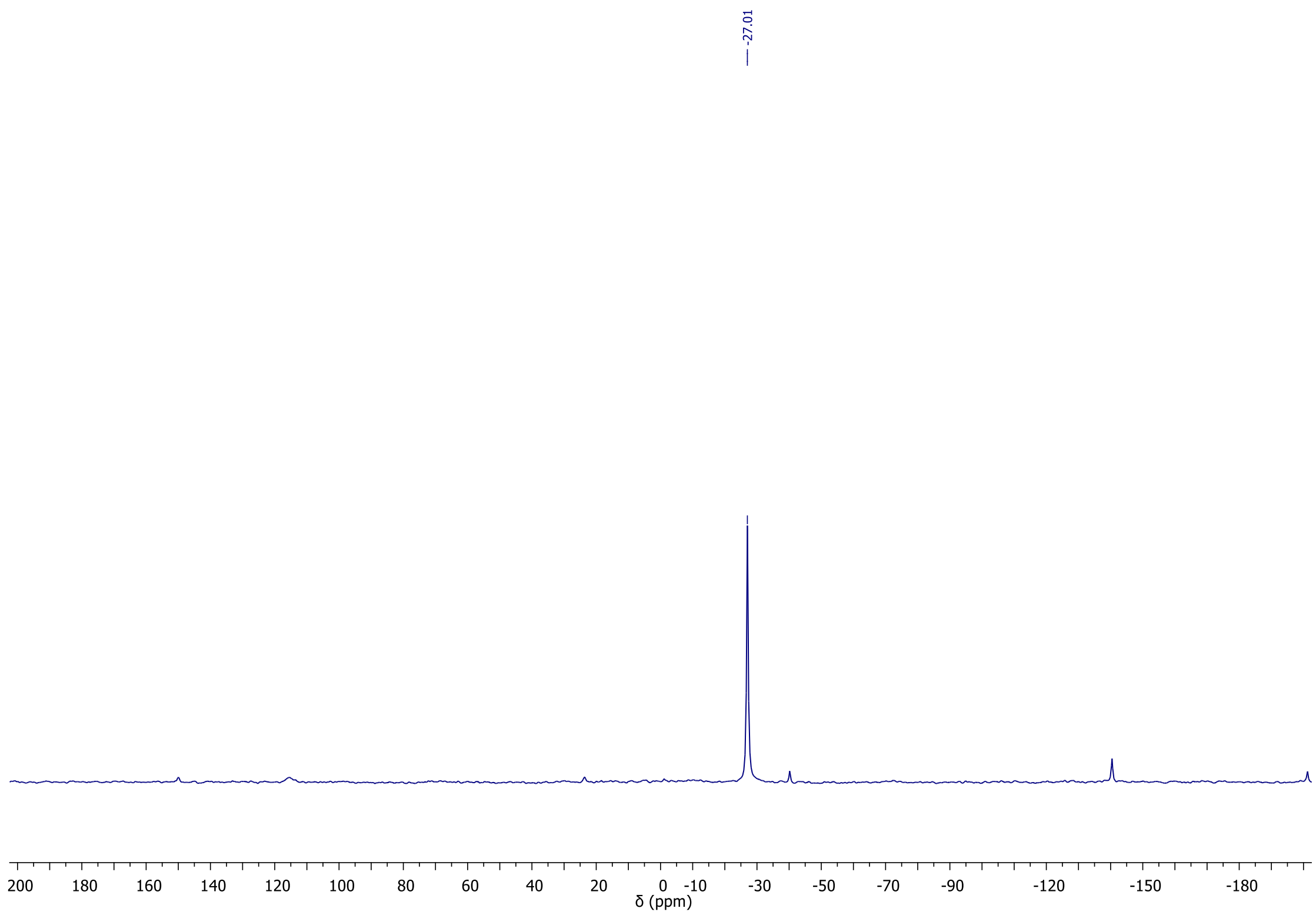

Figure S21: IR spectrum of **3** (ATR, 64 scans, cm^{-1}).

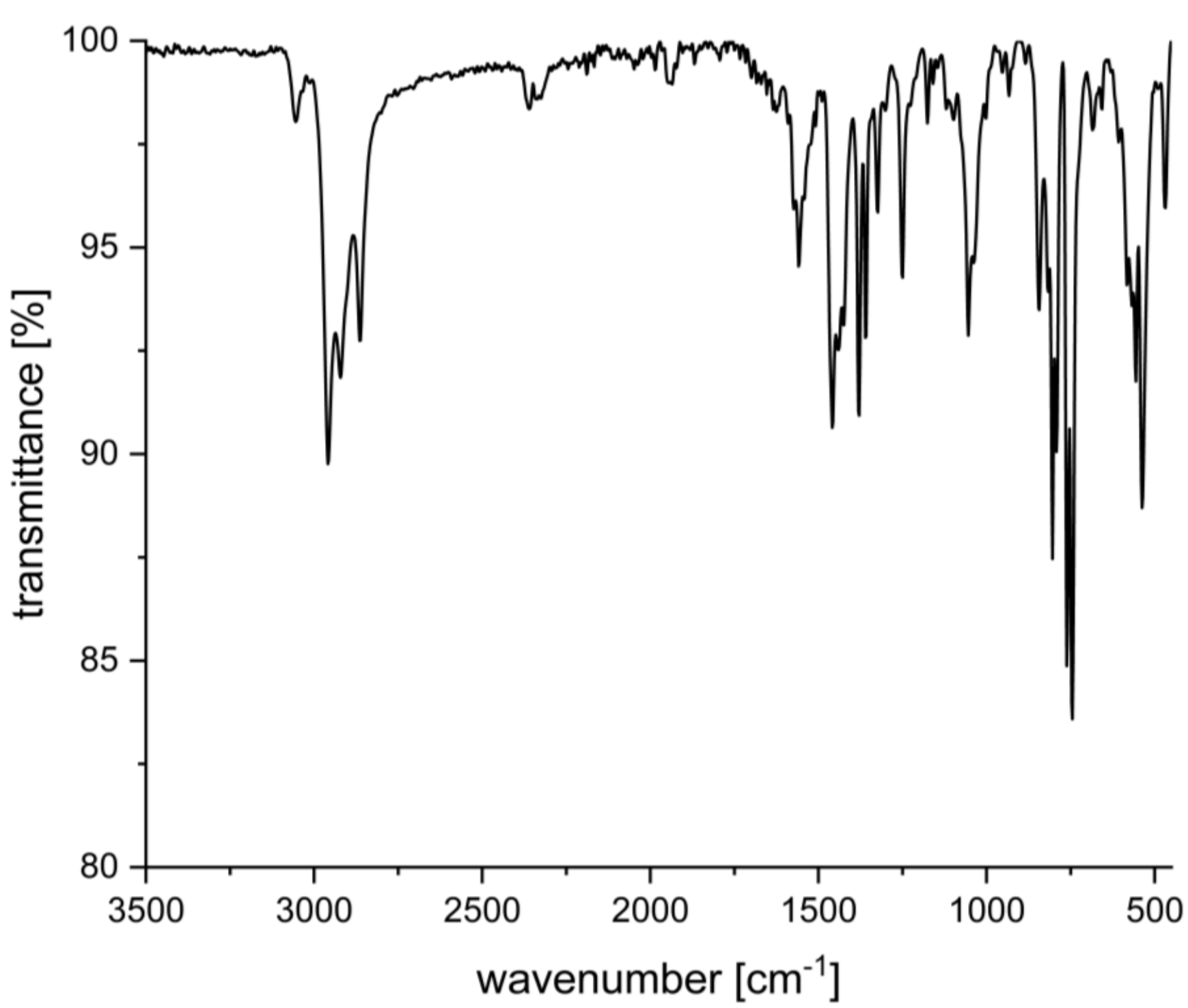

Figure S22: LIFDI mass spectrum of **3** (toluene).

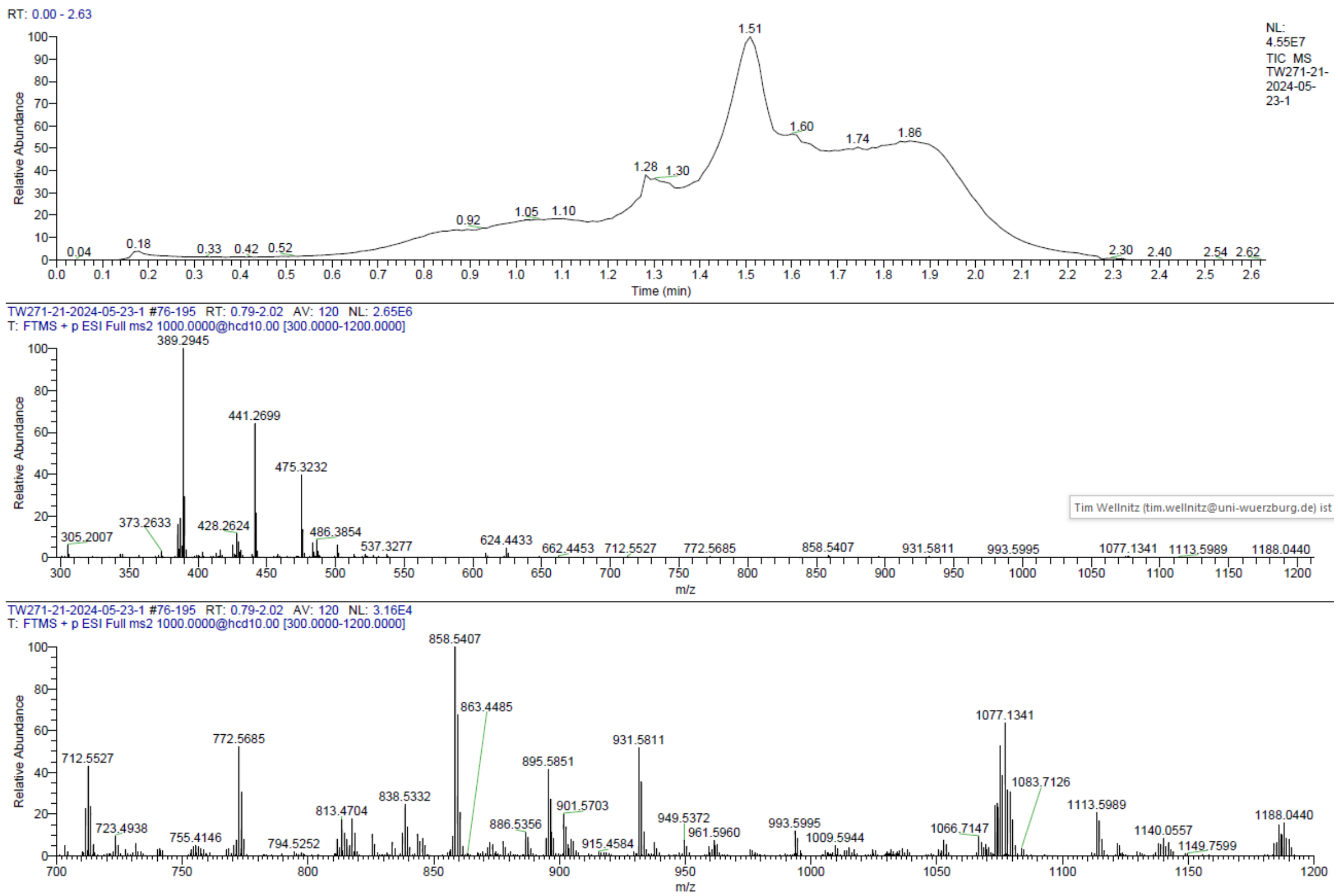

Figure S23: $^{31}P\{^1H\}$ NMR monitoring spectrum from heating **3** at 80 °C in C_6D_6 over a period of 16 h (*top*: after heating; *bottom*: before heating) (162.1 MHz, C_6D_6, 298 K), −27.0 ppm, **3** (light blue box), −140.5 ppm ^{Dip}TerPH$_2$, −201.3 ppm **^{Dip}TerPCO** (light red box). Major species at 115.6 and 9.7 ppm so far unidentified (light green box).

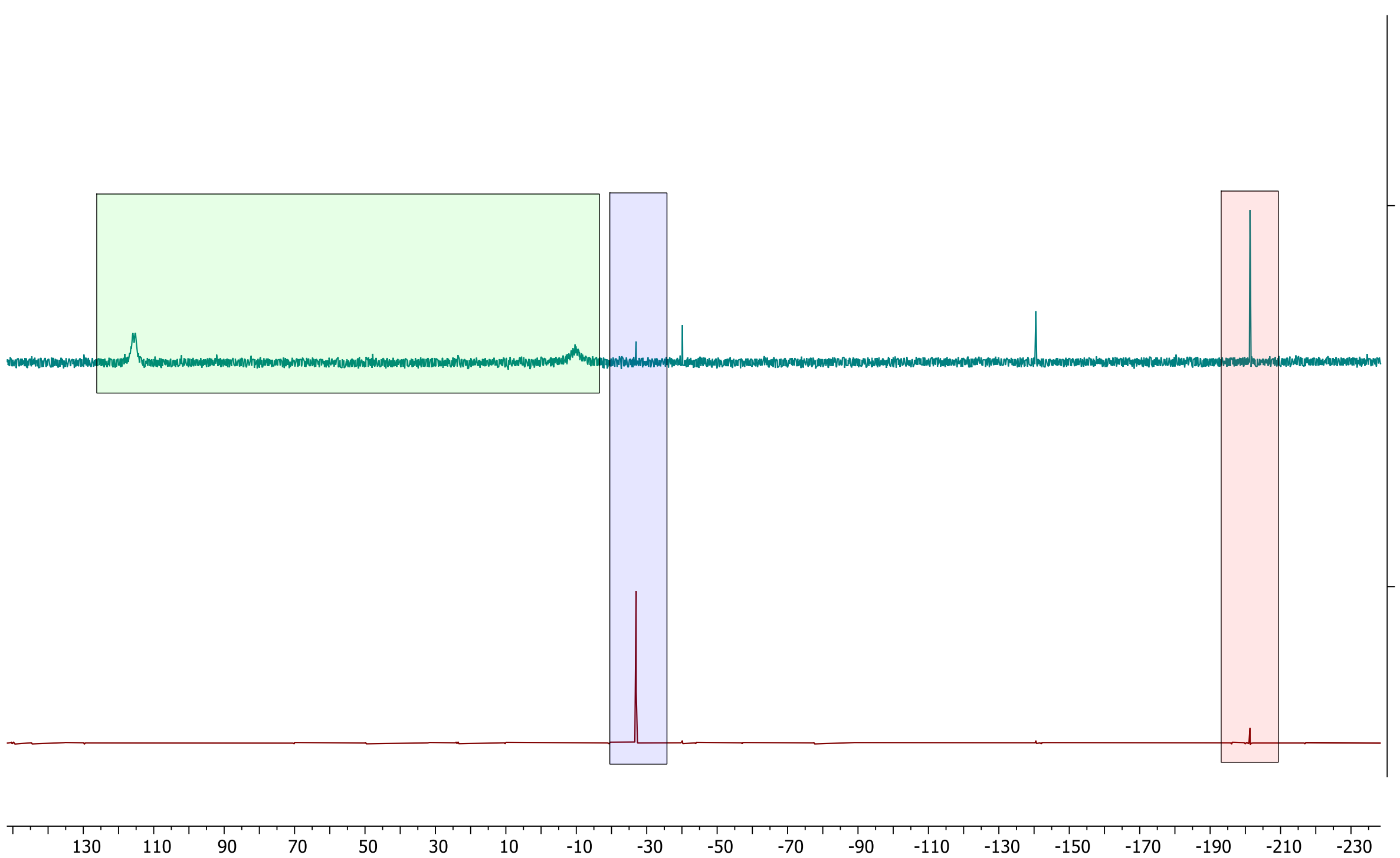

3.3. Synthesis of **4**

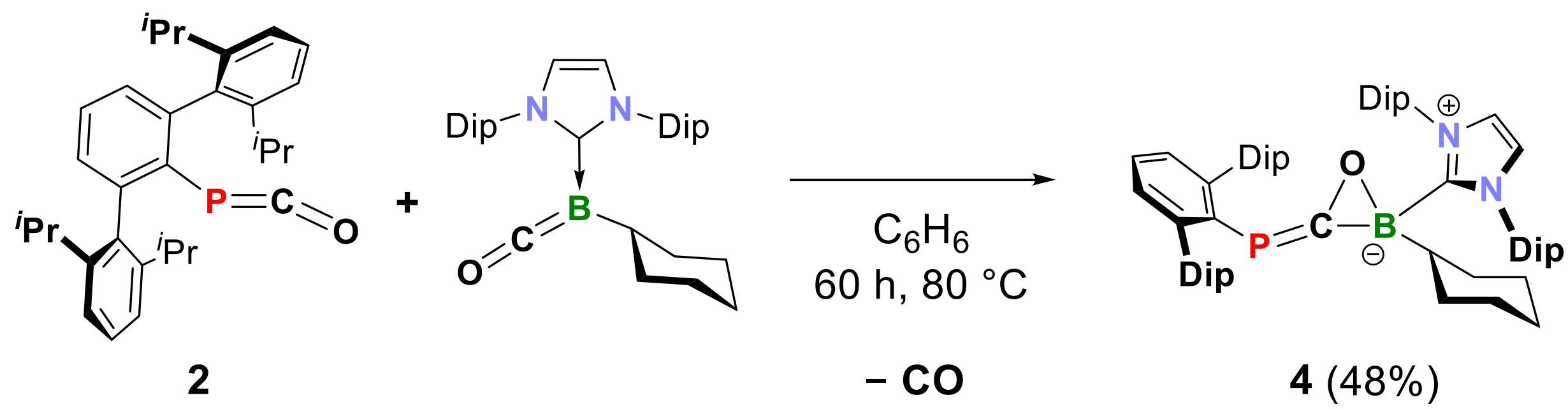

DipTerPAlCp* (22.0 mg, 37.3 μmol, 1.00 eq.) was dissolved in benzene (0.4 mL) in a *J.-Young* NMR tube. The purple solution was degassed and CO_2 (1 atm) was added at ambient temperature. After 20 min the formation of an orange solution was observed. Full conversion of **DipTerPAlCp*** to **DipTerPCO** was confirmed via $^{31}P\{^1H\}$ NMR spectroscopy. Subsequently, **IDip(Cy)BCO** (19.0 mg, 37.3 μmol, 1.00 eq.) was added at ambient temperature. After 60 h at 80 °C the formation of a red solution was observed. Full conversion of **DipTerPCO** to **4** was confirmed via $^{31}P\{^1H\}$ NMR spectroscopy. All volatile compounds were removed *in vacuo* (~1×10^{-3} mbar) at ambient temperature. The pale red solid was dissolved in *n*-pentane (0.4 mL) and filtered over a pipette equipped with a glass microfiber pad. The red solution was stored at −30 °C, which resulted in the precipitation of a white solid. The supernatant was removed and the colourless solid was washed with *n*-pentane (2×0.1 mL). The product was dried at ambient temperature in a glovebox.

Yield of 4: 16.8 mg (17.9 μmol, 48%) as white solid.

Suitable crystals for single crystal X-ray diffraction were obtained by slow diffusion of *n*-pentane in a toluene solution of **4** at ambient temperature for 24 h.

^{1}**H NMR** (600.2 MHz, Tol-d_8, 213 K): δ = −1.19 – −1.06 (m, 1 H, *H*-Cy), −0.05 – 0.03 (m, 1 H, *H*-Cy), 0.22 – 0.32 (m, 1 H, *H*-Cy), 0.41 – 0.52 (m, 1 H, *H*-Cy), 0.64 – 0.70 (m, 1 H, *H*-Cy), 0.72 (d, $^3J_{(H-H)}$ = 6.4 Hz, 3 H, CH(C*H*$_3$)(CH$_3$) (group A))[3], 0.77 (d, $^3J_{(H-H)}$ = 6.3 Hz, 3 H, CH(C*H*$_3$)(CH$_3$) (group B)), 0.84 – 0.90 (m, superimposed signal, 6 H, CH(C*H*$_3$)(CH$_3$) (group C + D)), 0.93 (d, $^3J_{(H-H)}$ = 6.7 Hz, 2 H, CH(CH$_3$)(C*H*$_3$) (group D)), 0.98 (d, $^3J_{(H-H)}$ = 6.5 Hz, 3 H, CH(C*H*$_3$)(CH$_3$) (group E)), 1.02 – 1.07 (m, 1 H, *H*-Cy), 1.09 (d, $^3J_{(H-H)}$ = 6.6 Hz, 3 H, CH(C*H*$_3$)(CH$_3$) (group F)), 1.13 – 1.15 (m, 1 H, *H*-Cy), 1.18 (d, $^3J_{(H-H)}$ = 6.7 Hz, 3 H, CH(CH$_3$)(C*H*$_3$) (group F)), 1.21 (d, $^3J_{(H-H)}$ = 6.6 Hz, 3 H, CH(C*H*$_3$)(CH$_3$) (group G)), 1.23 (d, $^3J_{(H-H)}$ = 6.4 Hz, 4 H, CH(CH$_3$)(C*H*$_3$) (group A) + *H*-Cy), 1.24 −1.29 (m, superimposed signal, 6 H, CH(CH$_3$)(C*H*$_3$) (group B + H)), 1.48 (d, $^3J_{(H-H)}$ = 6.5 Hz, 3 H, CH(C*H*$_3$)(CH$_3$) (group H)), 1.52 (d, $^3J_{(H-H)}$ = 6.6 Hz, 3 H, CH(CH$_3$)(C*H*$_3$) (group C)), 1.54 (d, $^3J_{(H-H)}$ = 6.5 Hz, 3 H, CH(CH$_3$)(C*H*$_3$) (group E)), 1.56 – 1.59 (m, 1 H, *H*-Cy), 1.63 (d, $^3J_{(H-H)}$ = 6.6 Hz, 3 H, CH(CH$_3$)(C*H*$_3$) (group G)), 1.65 – 1.68 (m, 1 H, *H*-Cy), 1.70 – 1.76 (m, 1 H, *H*-Cy), 1.90 (hept, 1 H, C*H*(CH$_3$)(CH$_3$) (group B)), 2.40 (hept, $^3J_{(H-H)}$ = 6.7 Hz, C*H*(CH$_3$)(CH$_3$) (group F)), 2.61 (hept, $^3J_{(H-H)}$ = 6.4 Hz, 1 H, C*H*(CH$_3$)(CH$_3$) (group A)), 2.68 (hept, $^3J_{(H-H)}$ = 6.6 Hz, 1 H, C*H*(CH$_3$)(CH$_3$) (group G)), 3.24 – 3.34 (m, superimposed signal, 2 H, C*H*(CH$_3$)(CH$_3$) (group C + H)), 3.37 (hept, $^3J_{(H-H)}$ = 6.7 Hz, 1 H, C*H*(CH$_3$)(CH$_3$) (group D)), 3.49 (br, 1 H, C*H*(CH$_3$)(CH$_3$) (group E)), 5.79 (d, $^3J_{(H-H)}$ = 1.5 Hz, 1 H, DipN-C*H*), 6.00 (d, $^3J_{(H-H)}$ = 1.5 Hz, 1 H, DipN-C*H*), 6.79 (dd, $^3J_{(H-H)}$ = 7.4 Hz, $^4J_{(H-H)}$ = 1.3 Hz, 1H, *m*-C*H* (NHC-Dip) (group J)), 6.85 (d, $^3J_{(H-H)}$ = 7.6 Hz, 1 H, *m*-C*H* (NHC-Dip) (group I)), 6.90 (d, $^3J_{(H-H)}$ = 7.7 Hz, 1 H, *m*-C*H* (Ter-Dip) (group L)), 6.96 (m, 2 H, *p*-/ *m*-C*H* (NHC-Dip) (group J))[4], 7.04 (m, 1 H, aryl-*H*)[5], 7.16 (m, 2 H, *m*-C*H* (NHC-Dip) (group I) + *m*-C*H* (Ter-Dip) (group K))[6], 7.22 (m, 2 H, aryl-*H*), 7.31 (d, $^3J_{(H-H)}$ = 7.7 Hz, 1 H, *m*-C*H* (^{Dip}Ter)), 7.36 (d, $^3J_{(H-H)}$ = 7.7 Hz, 1 H, *m*-C*H* (^{Dip}Ter)), 7.51 (t, $^3J_{(H-H)}$ = 7.7 Hz, 1 H, *p*-C*H* (^{Dip}Ter)) ppm.

---

[3] Due to rotational hindrance of **4**, NMR experiments were conducted at −60 °C to avoid wobbeling which leads to splitting of the NMR signals. Groups A to H are used to assign the signals to the isopropyl groups. Groups I, J, K and L are used to assign the Dip groups. I and J belongs to the NHC-Dip groups, K and L are assigned as Ter-Dip groups. The isopropyl groups A and F belongs to the NHC-Dip group I, B and D to the NHC-Dip group J, C and E to the Ter-Dip group K, G and H to the Ter-Dip group L.

[4] Superimposed with toluene-d_8 signal. Assigned with ^{1}H/^{13}C HSQC and ^{1}H/^{13}C HMBC nmr.

[5] Superimposed with toluene-d_8 signal. Assigned with ^{1}H/^{13}C HSQC and ^{1}H/^{13}C HMBC nmr.

[6] Superimposed with toluene-d_8 signal. Assigned with ^{1}H/^{13}C HSQC and ^{1}H/^{13}C HMBC nmr.

$^{11}B\{^1H\}$ NMR (192.6 MHz, Tol-d_8, 213 K): δ = −12.0 (br) ppm.

$^{13}C\{^1H, ^{11}B\}$ NMR (150.9 MHz, Tol-d_8, 213 K): δ = 21.5 (s, $CH(CH_3)(\mathit{C}H_3)$ (group A)), 22.2 (s, $CH(\mathit{C}H_3)(CH_3)$ (group D)), 22.3 (s, $CH(CH_3)(\mathit{C}H_3)$ (group B)), 22.8 (s, $CH(CH_3)(\mathit{C}H_3)$ (group F)), 22.9 (s, $CH(CH_3)(\mathit{C}H_3)$ (group E)), 23.4 (s, $CH(CH_3)(C\mathit{H}_3)$ (group H)), 24.1 (d, $J_{(C\text{-}P)}$ = 5 Hz, $CH(CH_3)(\mathit{C}H_3)$ (group G)), 24.6 (s, $CH(\mathit{C}H_3)(CH_3)$ (group C)), 25.2 (s, $CH(\mathit{C}H_3)(CH_3)$ (group E)), 25.4 (s, $CH(C\mathit{H}_3)(CH_3)$ (group G)), 25.7 (s, $CH(\mathit{C}H_3)(CH_3)$ (group F)), 26.0 (s, $CH(\mathit{C}H_3)(CH_3)$ (group H)), 26.1 (s, $CH(\mathit{C}H_3)(CH_3)$ (group A)), 26.2 (s, $CH(CH_3)(\mathit{C}H_3)$ (group D)), 26.5 (s, $CH(\mathit{C}H_3)(CH_3)$ (group B)), 26.6 (s, *C*H-Cy), 26.7 (s, *C*H-Cy), 28.0 (s, $CH(CH_3)(\mathit{C}H_3)$ (group C)), 28.3 (s, $\mathit{C}H(CH_3)(CH_3)$ (group D)), 28.4 (s, $\mathit{C}H(CH_3)(CH_3)$ (group B) + *C*H-Cy)), 28.6 (s, *C*H-Cy), 28.8 (s, $\mathit{C}H(CH_3)(CH_3)$ (group A)), 28.9 (s, $\mathit{C}H(CH_3)(CH_3)$ (group F)), 30.6 (d, $J_{(C\text{-}P)}$ = 6 Hz, $C\mathit{H}(CH_3)(CH_3)$ (group E)), 30.9 (s, $\mathit{C}H(CH_3)(CH_3)$ (group G)), 31.2 (s, $\mathit{C}H(CH_3)(CH_3)$ (group H)), 31.3 (s, $\mathit{C}H(CH_3)(CH_3)$ (group C)), 31.8 (s, (s, *C*H-Cy), 122.3 (s, *m*-*C*H (Ter-Dip) (group K)), 122.4 (s, *m*-*C*H (Ter-Dip) (group L)), 122.5 (s, DipN-*C*H), 123.2 (s, *m*-*C*H (^{Dip}Ter)), 123.3 (s, *m*-*C*H (Ter-Dip) (group L)), 123.4 (s, *m*-*C*H (NHC-Dip) (group J)), 123.7 (s, DipN-*C*H), 123.9 (s, *m*-*C*H (Ter-Dip) (group K)), 124.7 (s, *m*-*C*H (NHC-Dip) (group J)), 126.0 (s, aryl-*C*H), 126.2 (s, *m*-*C*H (NHC-Dip) (group I)), 126.8 (s, aryl-*C*H), 127.7 (s, *p*-*C*H (^{Dip}Ter)), 128.0 (s, aryl-*C*H), 130.2 (s, aryl-*C*H), 131.1 (s, aryl-*C*H), 133.2 (s, *i*-*C* (NHC-Dip) (group J)), 134.1 (s, *i*-*C* (NHC-Dip) (group I)), 142.1 (d, $J_{(C\text{-}P)}$ = 6 Hz, *i*-*C* (Ter-Dip) (group L)), 142.5 (s, *i*-*C* (Ter-Dip) (group K)), 143.7 (s, *o*-*C* (NHC-Dip) (group I)), 143.8 (d, $^1J_{(C\text{-}P)}$ = 62 Hz, *i*-*C* (^{Dip}Ter)), 144.8 (s, *o*-*C* (NHC-Dip) (group I)), 145.1 (s, *o*-*C* (^{Dip}Ter)), 145.2 (d, $J_{(C\text{-}P)}$ = 23 Hz, *o*-*C* (^{Dip}Ter)), 145.5 (s, *o*-*C* (Ter-Dip) (group K)), 145.7 (s, *o*-*C* (Ter-Dip) (group L)), 145.9 (s, *o*-*C* (NHC-Dip) (group J)), 146.0 (s, *o*-*C* (NHC-Dip) (group J)), 147.0 (s, *o*-*C* (Ter-Dip) (group K)), 148.0 (s, *o*-*C* (Ter-Dip) (group L)), 167.9 (s, DipN-*C*-B), 208.1 (d, $^1J_{(C\text{-}P)}$ = 112 Hz, P=*C*-B) ppm.

$^{31}P\{^1H\}$ NMR (243.0 MHz, Tol-d_8, 213 K): δ = 18.9 (s) ppm.

LIFDI-MS: (m/z) Calc: 937.6445, 938.6409, 939.6442, 940.6476, 941.6509 [$C_{64}H_{84}BN_2OP$]; Found: 937.6426, 938.6387, 939.6418, 940.6451, 941.6487 [M].

IR (ATR, 24 scans, cm^{-1}): $\tilde{\nu}$ = 2960 (w), 2911 (w), 2860 (w), 1458 (w), 1303 (m), 1229 (s), 1179 (s), 1120 (s), 980 s), 805 (w), 746 (m), 718 (w), 647 (w), 522 (m), 465 (w).

Figure S24: ^{1}H NMR spectrum of **4** (600.2 MHz, Tol-d$_8$, 213 K).

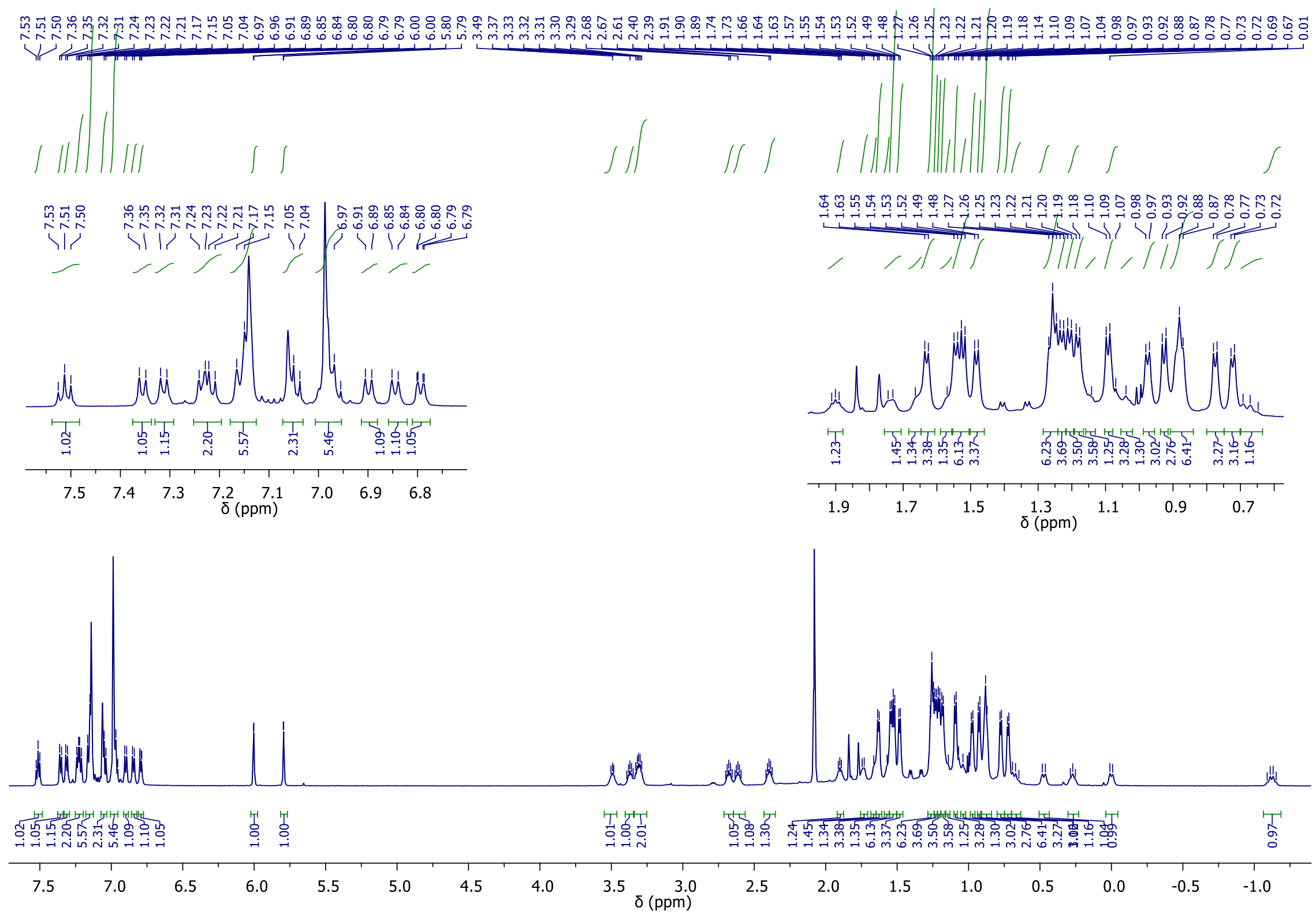

Figure S25: ^{11}B{^{1}H} NMR spectrum of **4** (192.6 MHz, Tol-d$_8$, 213 K).

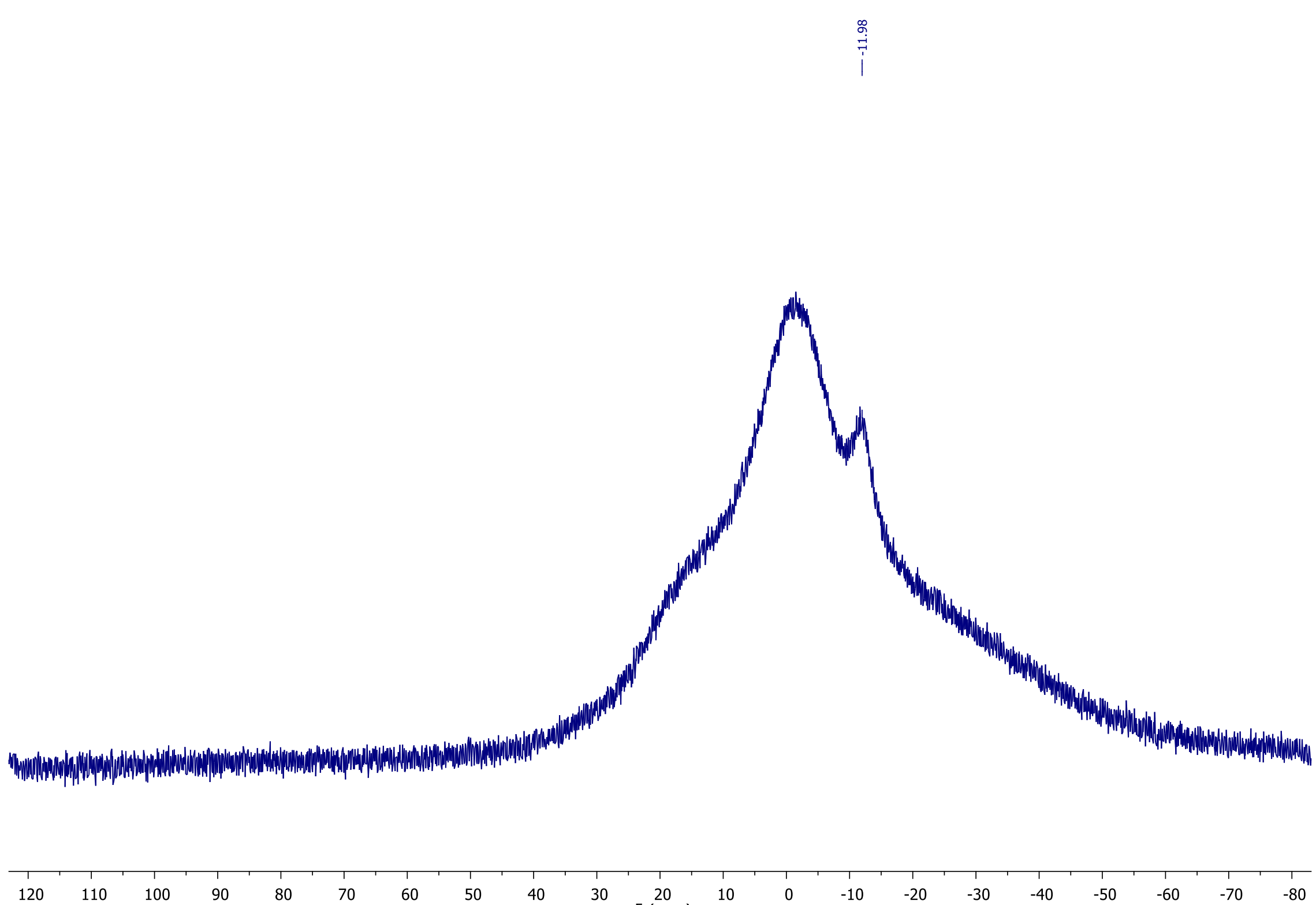

Figure S26: $^{13}C\{^{1}H, ^{11}B\}$a NMR spectrum of **4** (150.9 MHz, Tol-d_8, 213 K).

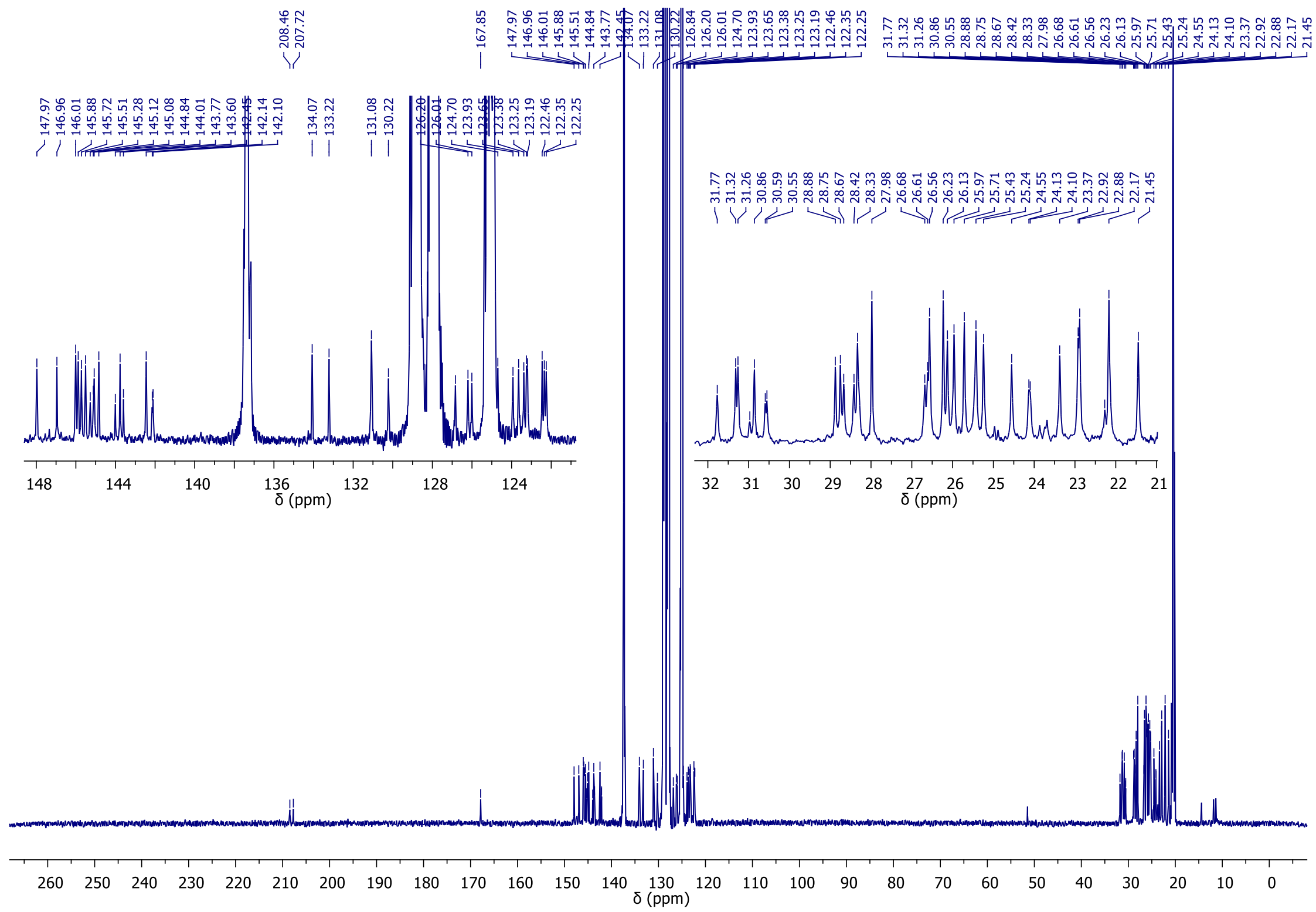

Figure S27: $^{31}P\{^{1}H\}$ NMR spectrum of **4** (243.0 MHz, Tol-d_8, 213 K), −138.8 ppm ^{Dip}TerPH$_2$, −200.2 ppm ^{Dip}TerPCO.

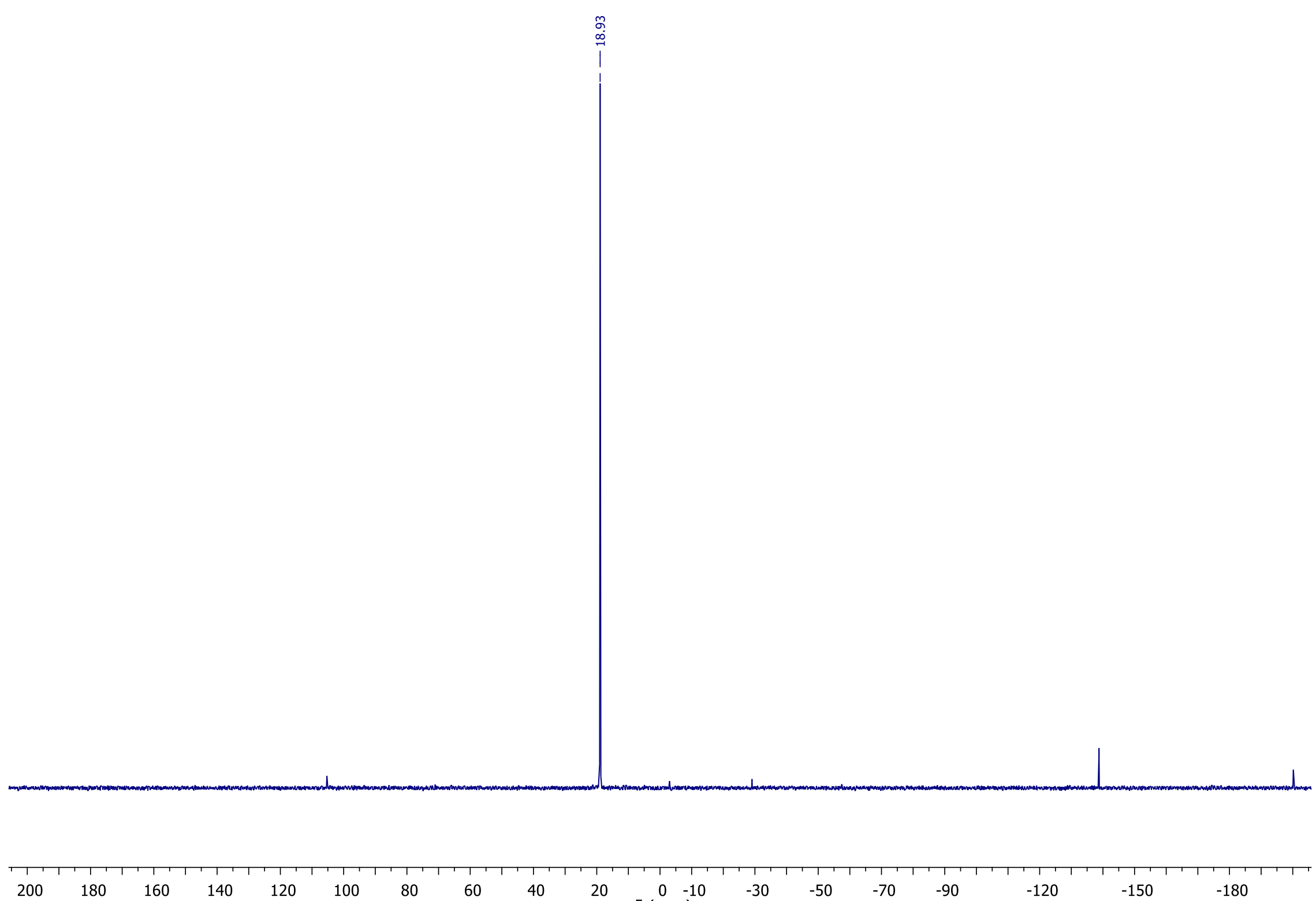

Figure S28: IR spectrum of **4** (ATR, 64 scans, cm^{-1}).

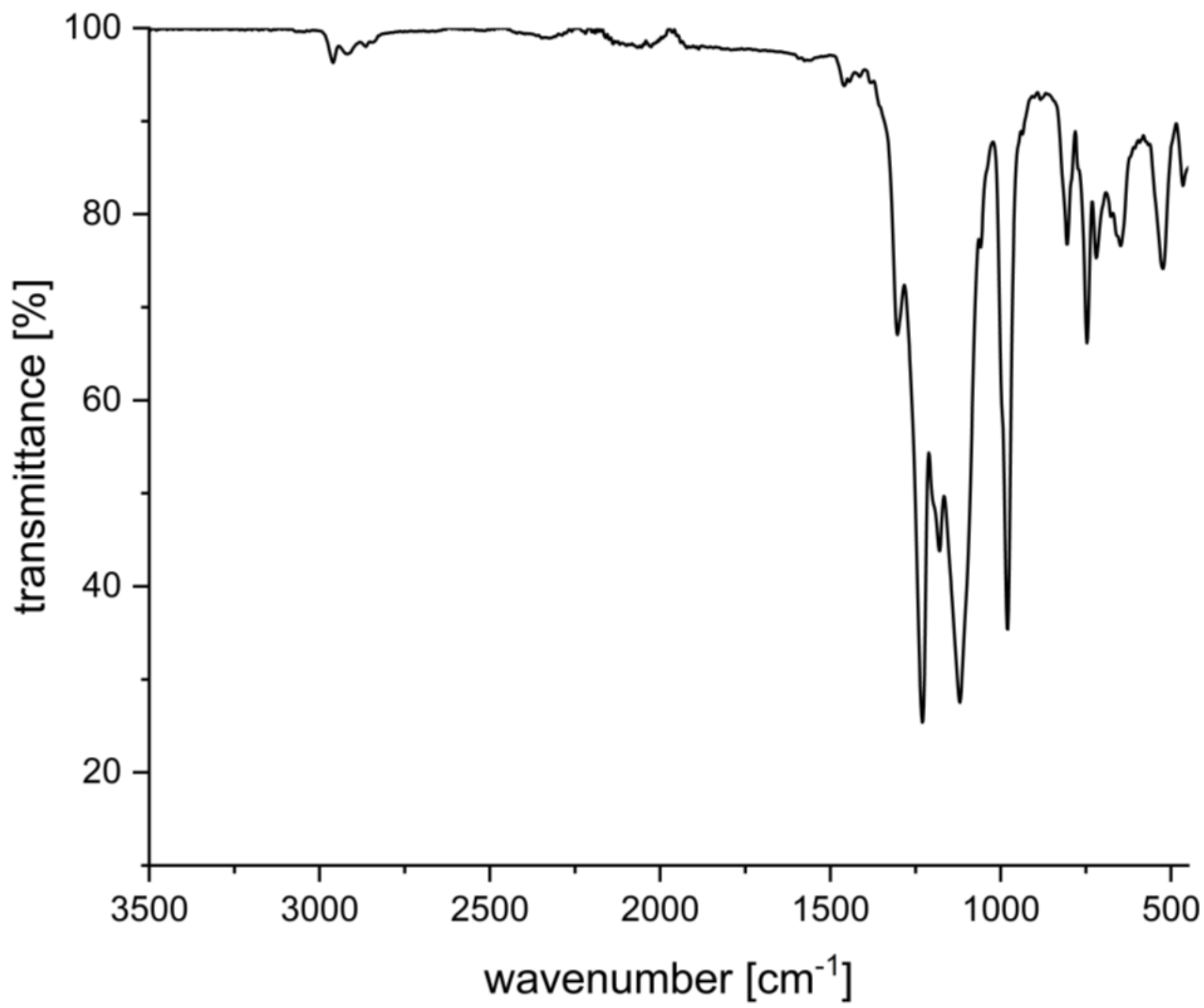

Figure S29: LIFDI mass spectrum of **4** (toluene).

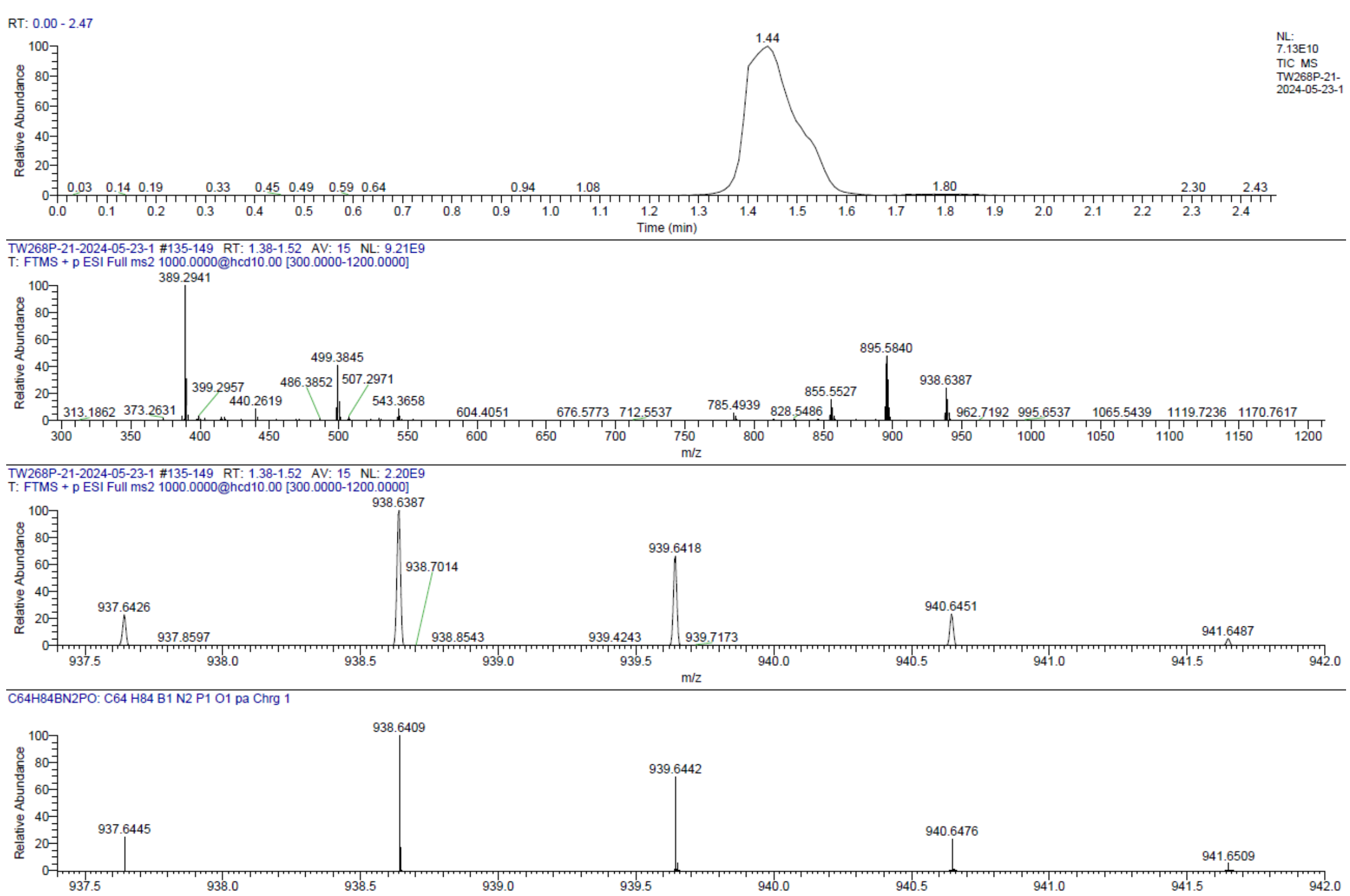

Figure S30: $^{31}P\{^1H\}$ NMR spectrum of the reaction mixture containing **4** (162.1 MHz, C_6D_6, 298 K), 102.3 ppm $\mathbf{4}^{\mathbf{inter}}$, −140.5 ppm $^{Dip}TerPH_2$, −201.3 ppm ^{Dip}TerPCO.

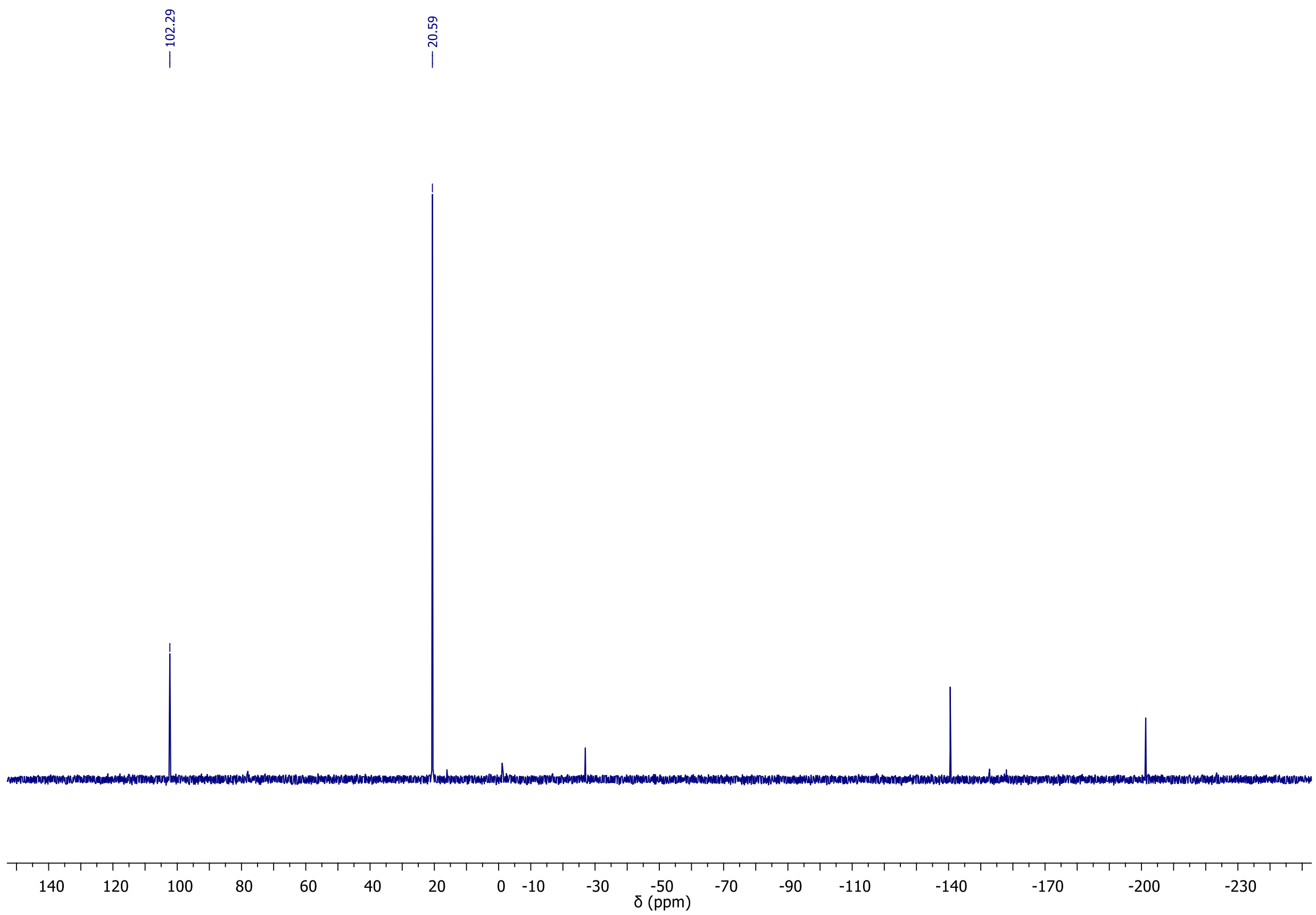

3.4. Synthesis of **5**

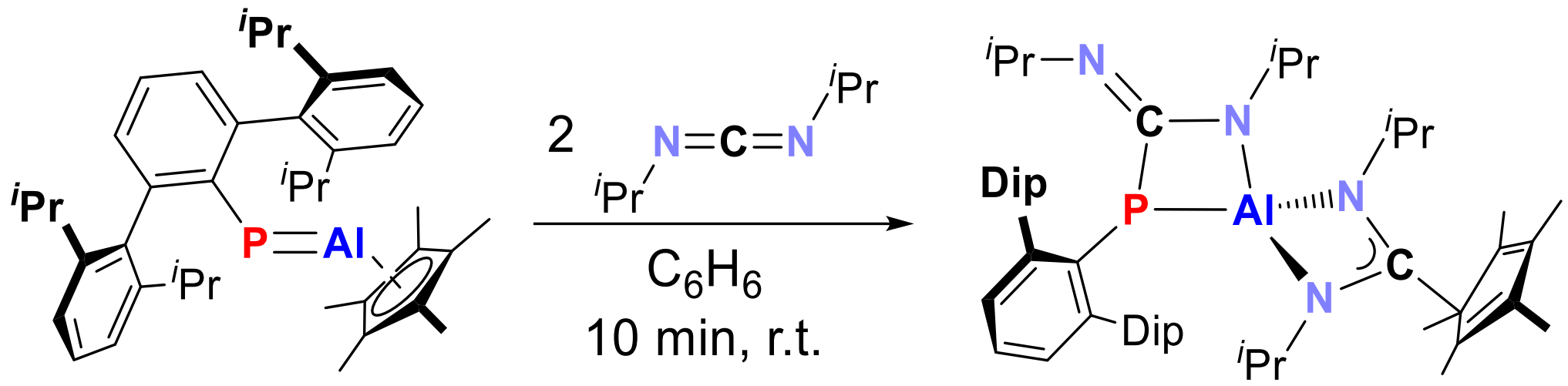

1 **5** (56%)

^{Dip}TerPAlCp* (20.0 mg, 33.9 µmol, 1.00 eq.) was dissolved in C_6H_6 (0.4 mL) in a *J.-Young* NMR tube and *N*,*N*-diisopropylcarbodiimide (DIC) (14.6 mg, 115 µmol, 3.4 eq.) was added to the purple solution at ambient temperature. Immediately the formation of a pale yellow solution was observed. Full conversion of **^{Dip}TerPAlCp*** to **5** was confirmed via $^{31}P\{^1H\}$ NMR spectroscopy. All volatile compounds were removed *in vacuo* (~1×10^{-3} mbar) at ambient temperature. The pale yellow solid was dissolved in *n*-pentane (0.4 mL) and filtered over a pipette equipped with a glass microfiber pad. Suitable crystals for single crystal X-ray diffraction and for full characterization were obtained by storing a saturated *n*-pentane solution of **5** at –30 °C for 24 h. The supernatant was removed and the colourless crystals were washed with cooled *n*-pentane (–30 °C) and dried at ambient temperature in a glovebox.

Yield of 5: 16.0 mg (19.0 µmol, 56%) as colourless crystals.

^{1}H NMR (600.2 MHz, Tol-d$_8$, 213 K): δ = 0.58 (d, $^3J_{(H-H)}$ = 6.0 Hz, 3 H, C(H)(C*H*$_3$)(CH$_3$) (group A))[7], 0.72 (d, $^3J_{(H-H)}$ = 6.0 Hz, 3 H, C(H)(CH$_3$)(C*H*$_3$) (group A)), 0.92 (m, 3 H, C(H)(C*H*$_3$)(CH$_3$) (group B)), 0.98 (d, $^3J_{(H-H)}$ = 5.7 Hz, 3 H, C(H)(C*H*$_3$)(CH$_3$) (group C)), 1.13 (br, 3H, C(H)(C*H*$_3$)(CH$_3$) (group D)), 1.15 – 1.18 (m, 6H, C(H)(CH$_3$)(C*H*$_3$) (group C + E)), 1.28 (d, $^3J_{(H-H)}$ = 6.6 Hz, 3 H, C(H)(C*H*$_3$)(CH$_3$) (group F)), 1.32 (d, $^3J_{(H-H)}$ = 5.2 Hz, 3 H, C(H)(C*H*$_3$)(CH$_3$) (group G)), 1.36 (d, $^3J_{(H-H)}$ = 6.2 Hz, 3 H, C(H)(CH$_3$)(C*H*$_3$) (group G)), 1.39 (d, $^3J_{(H-H)}$ = 5.6 Hz, 6 H, C(H)(CH$_3$)(C*H*$_3$) (group B + H)), 1.45 (br, 12 H, C$_5$(C*H*$_3$)$_2$(CH$_3$)(CH$_3$)(CH$_3$) + C(H)(CH$_3$)(C*H*$_3$) (group E + F)), 1.51 (s, 3 H, C$_5$(CH$_3$)$_2$(C*H*$_3$)(CH$_3$)(CH$_3$)), 1.67 (s, 3 H, C$_5$(CH$_3$)$_2$(CH$_3$)(C*H*$_3$)(CH$_3$)), 1.69 (d, *J* = 6.1 Hz, 3 H, C(H)(CH$_3$)(C*H*$_3$) (group D)), 1.75 (d, $^3J_{(H-H)}$ = 5.9 Hz, 3 H, C(H)(CH$_3$)(C*H*$_3$) (group H)), 1.78 (s, 3 H, C$_5$(CH$_3$)$_2$(CH$_3$)(CH$_3$)(C*H*$_3$)), 2.24 – 2.33 (m, 1 H, C(*H*)(CH$_3$)(CH$_3$) (group E)), 3.08 (br, 1 H, C(*H*)(CH$_3$)(CH$_3$) (group A)), 3.12 − 3.16 (m, 1 H, C(*H*)(CH$_3$)(CH$_3$) (group G)), 3.14 − 3.18 (m, 1 H, C(*H*)(CH$_3$)(CH$_3$) (group B)), 3.64 (br, 1 H, C(*H*)(CH$_3$)(CH$_3$) (group C)), 3.70 – 3.75 (m, 1 H, C(*H*)(CH$_3$)(CH$_3$) (group H)), 3.75 – 3.81 (m, 1 H, C(*H*)(CH$_3$)(CH$_3$) (group D)), 4.26 (br, 1 H, C(*H*)(CH$_3$)(CH$_3$) (group F)), 6.83 – 6.91 (m, 2 H, *p-/m-H* (^{Dipp}Ter)), 7.11 – 7.15 (m, 1 H, *m-H* (^{Dip}Ter))[8], 7.18 (d, $^3J_{(H-H)}$ = 7.6 Hz, 2 H, *m-H* (Dip) (group I + J), 7.27 (d, $^3J_{(H-H)}$ = 7.1 Hz, 1 H, *m-H* (Dip) (group J)), 7.30 – 7.35 (m, 2 H, *p-H* (Dip) (group J) and *m-H* (Dip) (group I), 7.37 – 7.41 (m, 1 H, *p-H* (Dip) (group I) ppm.

^{13}C{^{1}H} NMR (150.9 MHz, Tol-d$_8$, 213 K): δ = 11.0 (s, C$_5$(*C*H$_3$)$_2$(CH$_3$)(CH$_3$)(CH$_3$) + C$_5$(CH$_3$)$_2$(*C*H$_3$)(CH$_3$)(CH$_3$)), 11.6 (s, C$_5$(CH$_3$)$_2$(CH$_3$)(*C*H$_3$)(CH$_3$)), 12.8 (s, C$_5$(CH$_3$)$_2$(CH$_3$)(CH$_3$)(*C*H$_3$)), 22.0 (s, C(H)(CH$_3$)(*C*H$_3$) (group H)), 22.7 (s, C(H)(CH$_3$)(*C*H$_3$) (group D + E)), 23.1 (s, C(H)(CH$_3$)(*C*H$_3$) (group F)), 25.0 (s, C(H)(*C*H$_3$)(CH$_3$) (group F)), 25.3 (s, C(H)(CH$_3$)(*C*H$_3$) (group A)), 25.5 (s, C(H)(C*H*$_3$)(CH$_3$) (group D)), 26.1 (s, C(H)(CH$_3$)(*C*H$_3$) (group B)),26.2 (s, C(H)(*C*H$_3$)(CH$_3$) (group G)), 26.3 (s, C(H)(CH$_3$)(*C*H$_3$) (group H)), 26.4 (s, C(H)(*C*H$_3$)(CH$_3$) (group A)), 26.5 (s, C(H)(*C*H$_3$)(CH$_3$) (group C)), 26.7 (s, C(H)(*C*H$_3$)(CH$_3$)

---

[7] Due to rotational hindrance and thermal instability of **5**, NMR experiments were conducted at −60 °C to avoid wobbeling which leads to splitting of the nmr signals. Groups A to H are used to assign the signals to the isopropyl groups. The isopropyl groups A and C belongs to the amidinate unit, B and F to the other carbodiimide and D, E, G, H belongs to the terphenyl ligand. Additionally, the groups I and J belongs to the flanking Dipp groups of the terphenyl ligand. The isopropyl groups D and G are located at the Dip group J and the isopropyl groups E and H at Dip group I.

[8] Superimposed with toluene-d$_8$ signal. Assigned with the aid of ^{1}H/^{1}H COSY and ^{1}H/^{13}C HSQC NMR spectra.

(group B)), 26.9 (s, C(H)(CH_3)($\mathit{C}H_3$) (group C)), 27.5 (s, C(H)(CH_3)($\mathit{C}H_3$) (group G)), 30.7 (s, *C*(H)(CH_3)(CH_3) (group E + G)), 31.60 (d, $J_{(C-P)}$ = 12 Hz, *C*(H)(CH_3)(CH_3) (group D)), 31.9 (s, *C*(H)(CH_3)(CH_3) (group H)), 44.5 (s, *C*(H)(CH_3)(CH_3) (group A)), 44.9 (s, *C*(H)(CH_3)(CH_3) (group C)), 46.0 (s, *C*(H)(CH_3)(CH_3) (group F)), 54.9 (d, $J_{(C-P)}$ = 15 Hz, *C*(H)(CH_3)(CH_3) (group B)), 63.7 (s, *i-C*$_5$(CH_3)$_5$), 121.4 (s, *m-C*H (Dip) (group I)), 122.5 (s, *m-C*H (^{Dipp}Ter)), 123.2 (s, *m-C*H (Dip) (group J)), 123.4 (s, *m-C*H (Dip) (group I)), 124.1 (s, *m-C*H (Dip) (group J)), 127.8 (s, *p-C*H (Dip) (group J))[9], 128.3 (s, *p-C*H (Dip) (group I)), 129.7 (s, *m-C*H (^{Dip}Ter)), 133.4 (s, *p-C*H (^{Dip}Ter)), 135.8 (s, *o-C*$_5$(CH_3)$_5$), 136.6 (s, *o-C*$_5$(CH_3)$_5$), 139.8 (s, *m-C*$_5$(CH_3)$_5$), 139.9 (d, $J_{(C-P)}$ = 30 Hz, *i-C* (^{Dip}Ter)), 140.3 (s, *i-C* (Dip) (group J), 140.9 (s, *o-C* (^{Dip}Ter)), 141.0 (s, *m-C*$_5$(CH_3)$_5$), 141.8 (s, *i-C* (Dip) (group I)), 142.6 (d, $J_{(C-P)}$ = 6.4 Hz, *o-C* (^{Dip}Ter)), 145.2 (s, *o-C* (Dip) (group J)), 145.8 (s, *o-C* (Dip) (group I)), 147.6 (s, *o-C* (Dip) (group I)), 147.8 (s, *o-C* (Dip), (group J)), 161.1 (d, $J_{(C-P)}$ = 52 Hz, C_q, P-DIC-*C*), 178.2 (s, C_q, Al-DIC-*C*) ppm.

^{31}P{^{1}H} NMR (242.9 MHz, Tol-d_8, 213 K): δ = −56.8 (s) ppm.

IR (ATR, 24 scans, cm^{-1}): $\tilde{\nu}$ = 3058 (w), 2959 (m), 2925 (m), 2866 (m), 2183 (w), 2034 (w), 2014 (w), 1981 (w), 1920 (w), 1790 (w), 1658 (w), 1582 (s, C=N), 1442 (s), 1397 (s), 1373 (s), 1361 (m), 1346 (m), 1321 (s), 1258 (m), 1222 (w), 1196 (s), 1137 (m), 1122 (w), 1102 (m), 1076 (w), 1057 (m), 1036 (w), 1005 (m), 948 (w), 936 (w), 887 (w), 817 (w), 804 (m), 789 (m), 761 (s), 747 (m), 740 (m), 726 (w), 686 (w), 657 (s), 643 (m), 599 (m), 567 (w), 557 (w), 521 (w), 466 (w), 447 (w), 428 (w).

LIFDI-MS: (m/z) Calc: 842.5936, 843.5968, 844.6001, 845.6034 [$C_{54}H_{80}AlN_4P$]; Found: 843.5989, 844.6024, 845.6061, 846.6095 $[M+H]^+$.

[9] Superimposed with toluene-d_8 signal. Assigned with ^{1}H/^{13}C HSQC and DEPT-135 nmr.

Figure S31: ^{1}H NMR spectrum of **5** (600.2 MHz, Tol-d$_8$, 213 K); 0.92, 1.24 ppm *n*-pentane, 1.04, 3.29 ppm DIC.

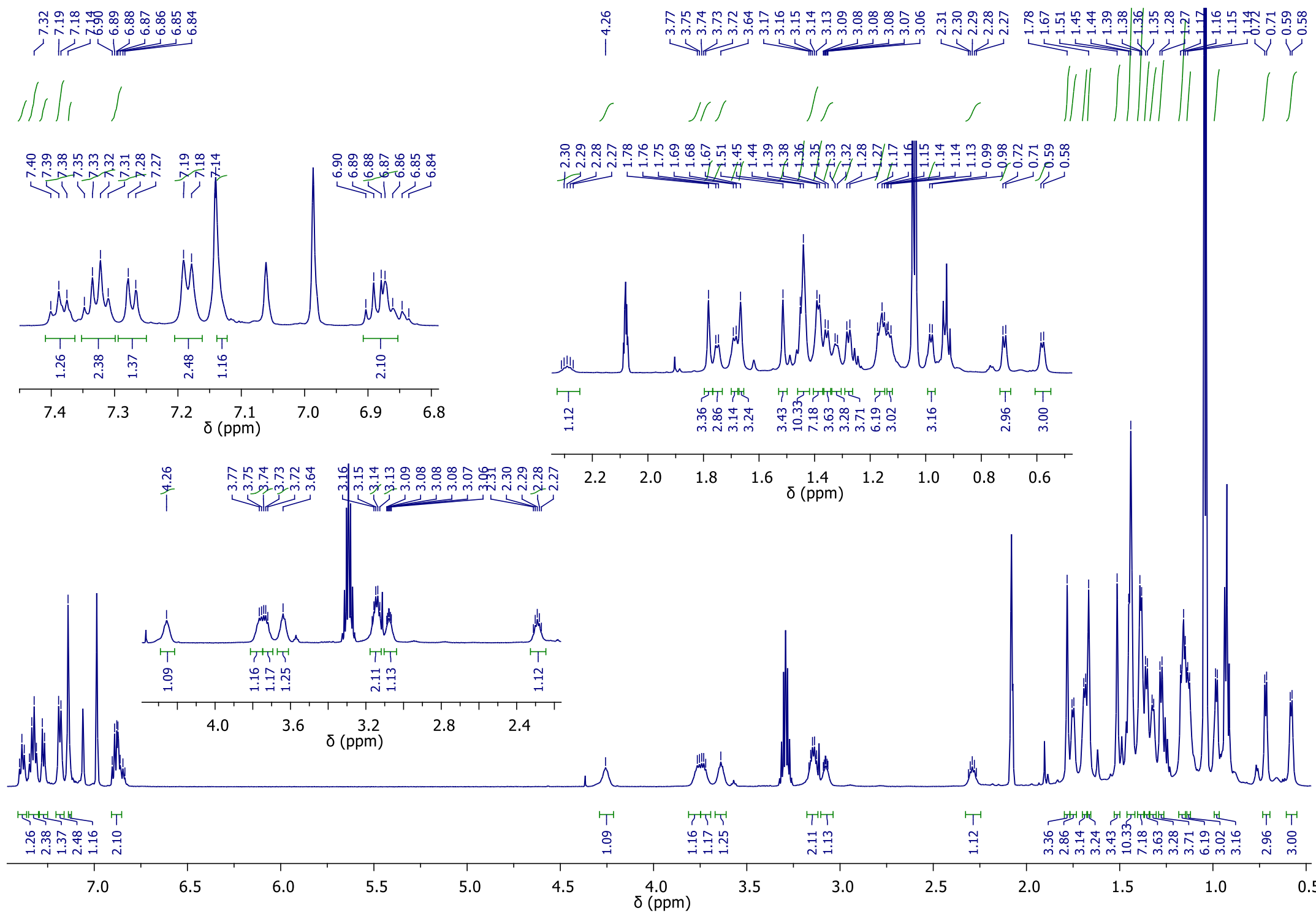

Figure S32: ^{13}C{^{1}H} NMR spectrum of **5** (150.9 MHz, Tol-d$_8$, 213 K); 14.6, 23.1, 34.5 ppm *n*-pentane; 24.6, 48.7, 140.3 – 140.6 ppm DIC.

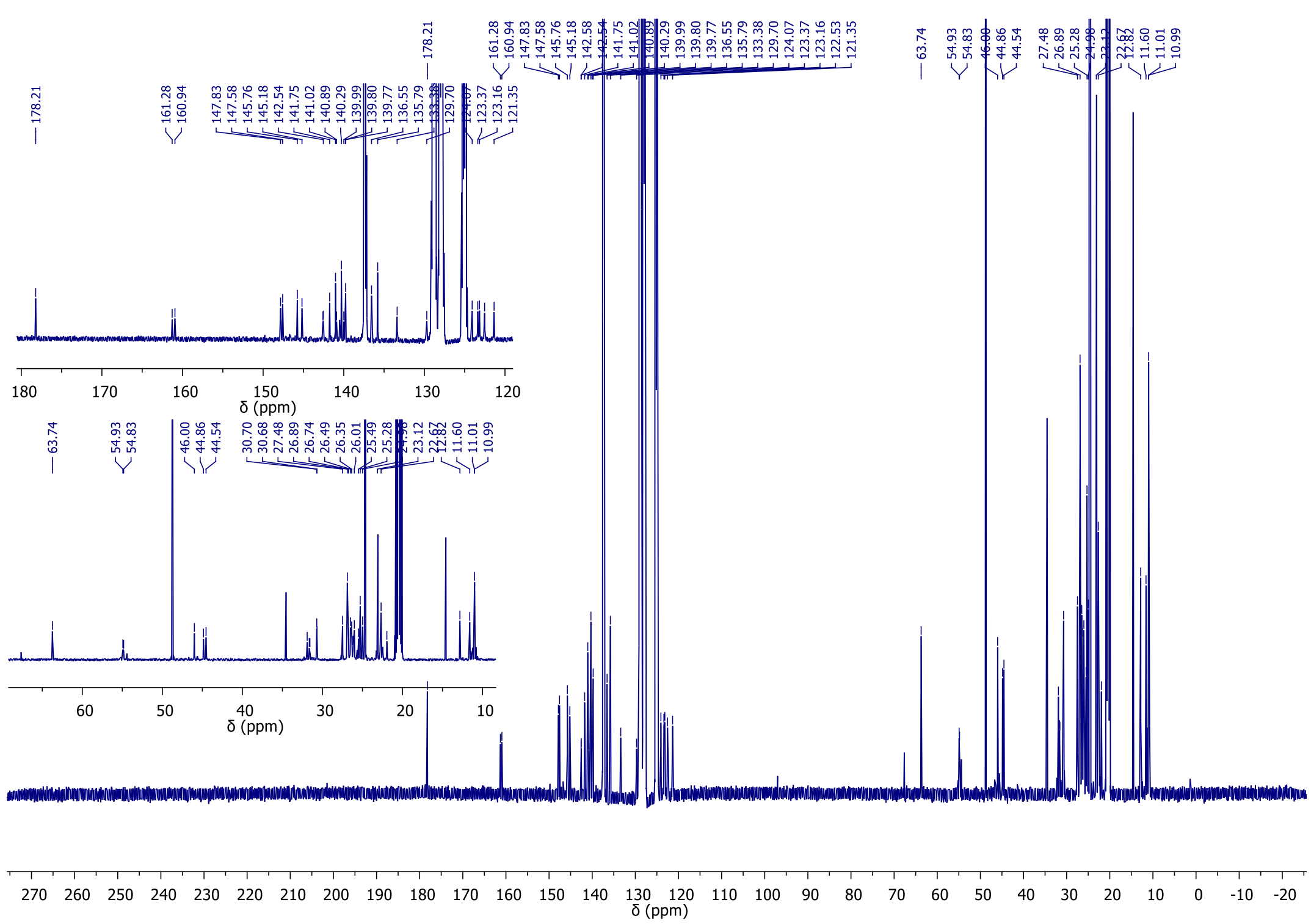

Figure S33: $^{31}P\{^1H\}$ NMR spectrum of **5** (242.9 MHz, Tol-d_8, 213 K).

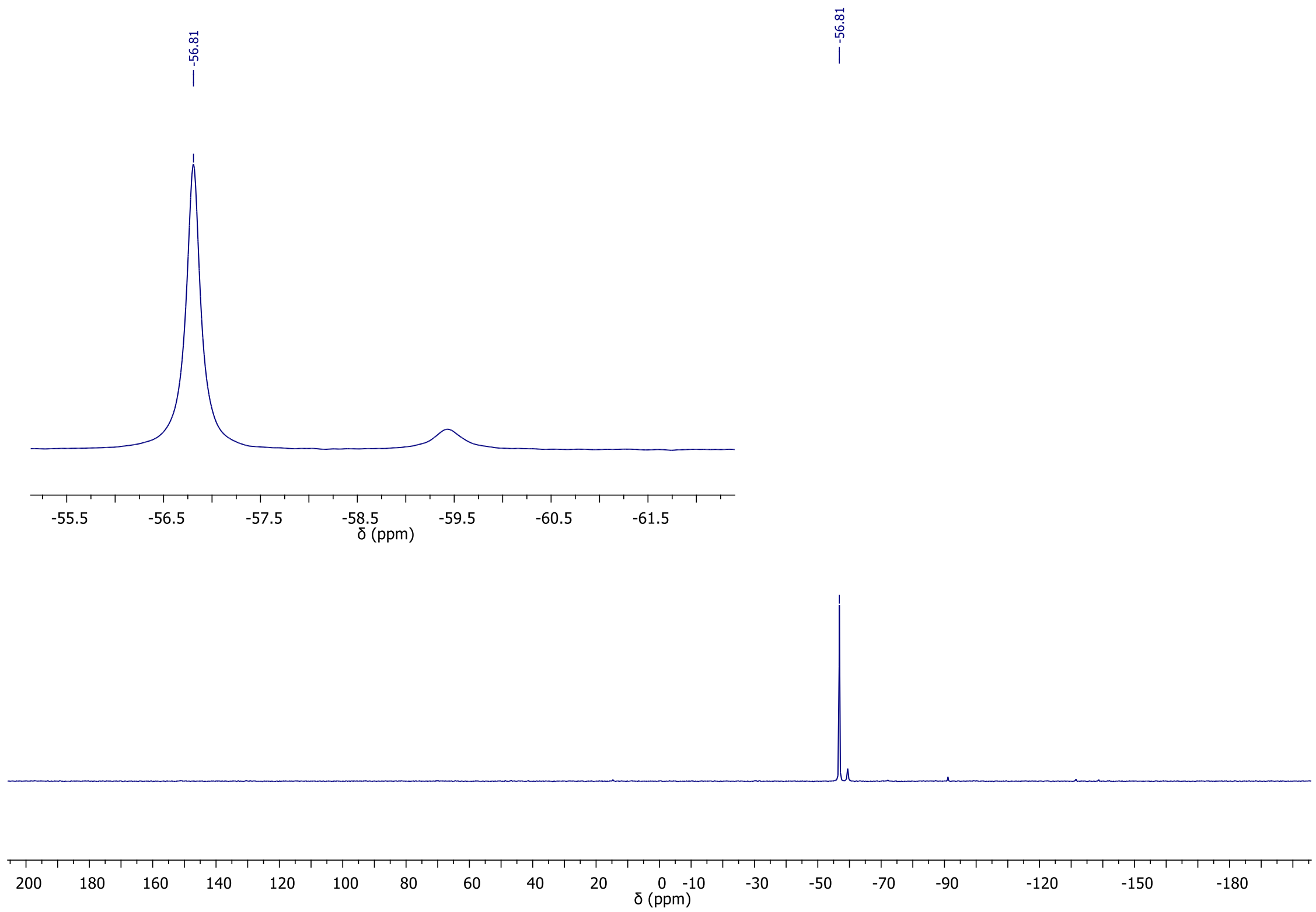

Figure S34: IR spectrum of **5** (ATR, 24 scans, cm^{-1}).

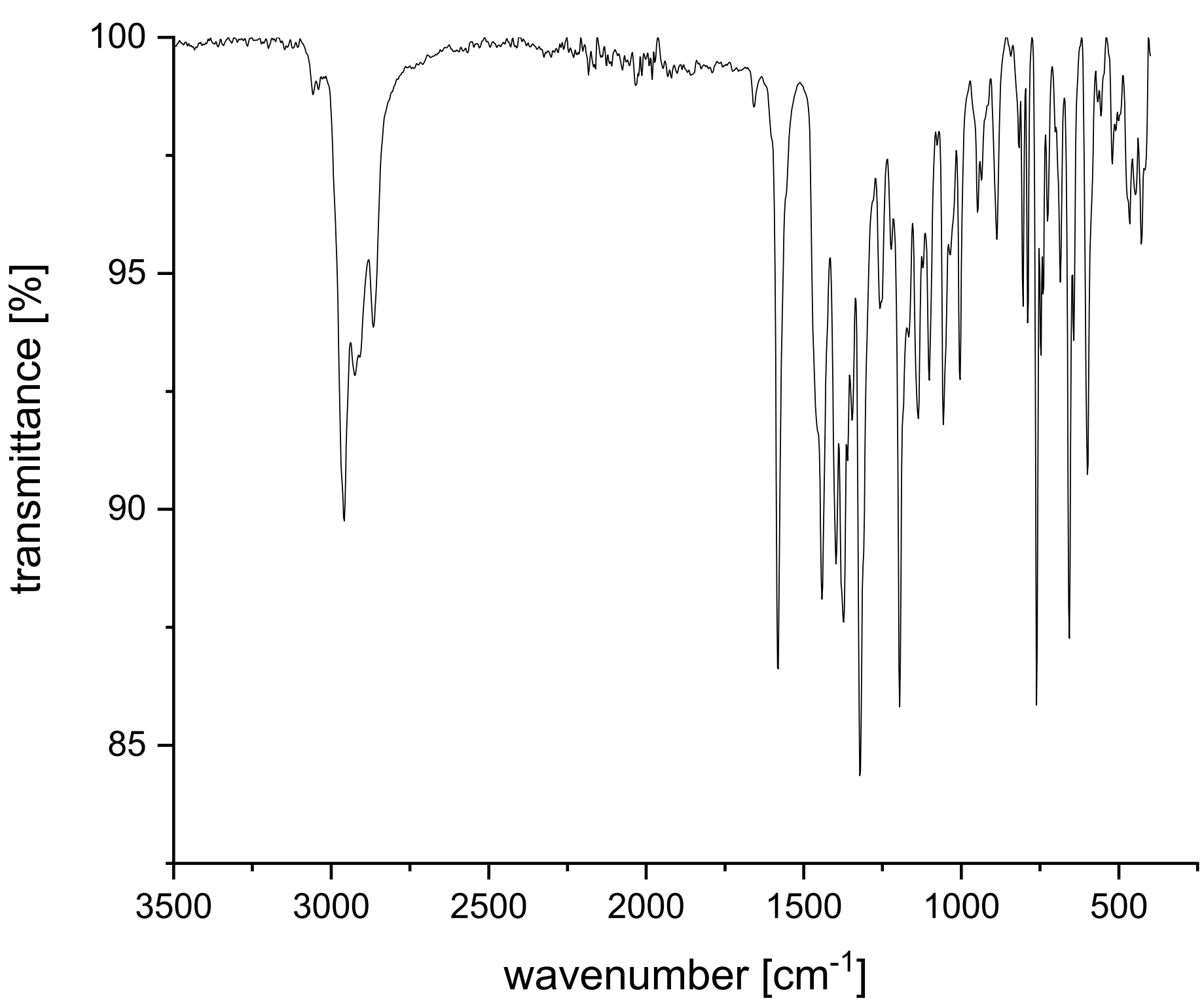

Figure S35: LIFDI mass spectrum of **5** (toluene).

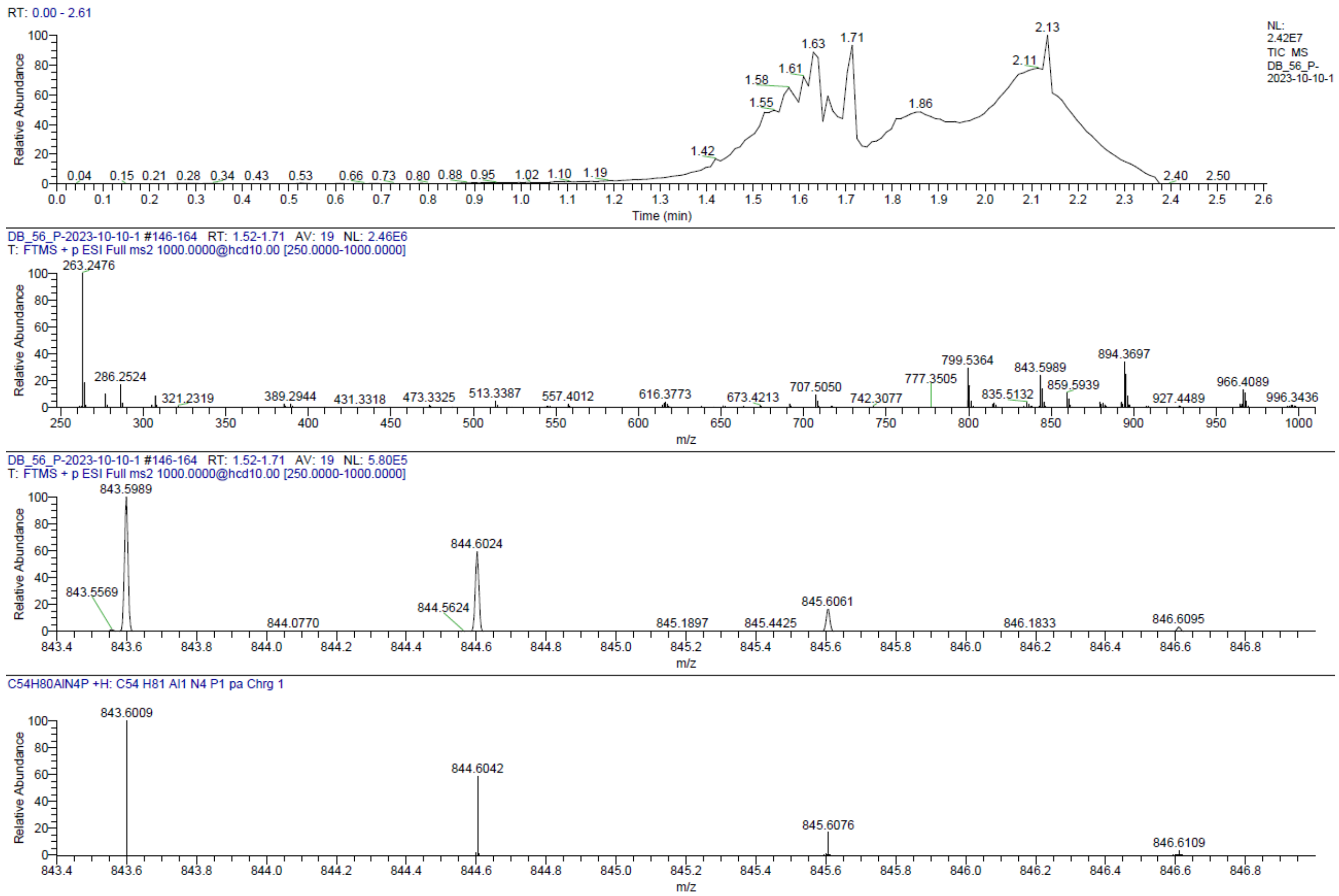

3.5. Synthesis of **6**

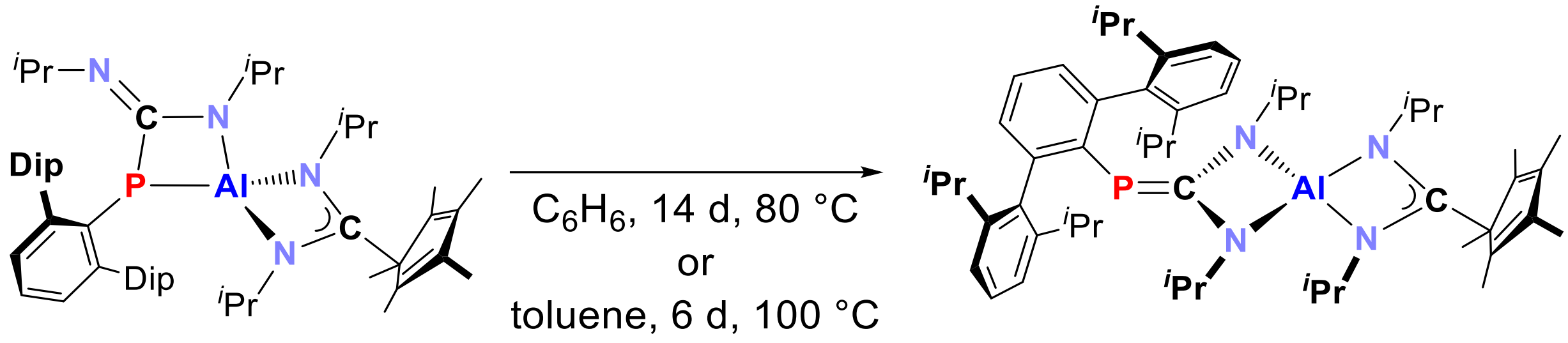

5 **6** (37%)

^{Dip}TerPAlCp* (30.0 mg, 50.8 μmol, 1.00 eq.) was dissolved in C_6H_6 (0.4 mL) in a *J.-Young* NMR tube and *N*,*N'*-Di(propan-2-yl)methanediimine (15.0 mg, 119 μmol, 2.30 eq.) was added at ambient temperature. Immediately the formation of a pale yellow solution was observed. Full conversion of **^{Dip}TerPAlCp*** to **5** was confirmed via $^{31}P\{^{1}H\}$ NMR spectroscopy. Subsequently the reaction mixture was heated for 6 days at 100 °C (oil bath) and full conversion of **5** to **6** was confirmed via $^{31}P\{^{1}H\}$ NMR spectroscopy. The solvent was evaporated in a glovebox atmosphere at ambient temperature. The orange oily residue was dissolved in *n*-hexane (0.35 mL) and hmdso (0.05 mL) and filtered over a pipette equipped with a glass microfiber pad. Suitable crystals for single crystal X-ray diffraction were obtained by evaporating a saturated *n*-hexane/hmdso solution of **6** at ambient temperature.

Yield of 6: 15.7 mg (18.6 μmol, 37%) as colourless crystals.

*Note: Compound **6** shows decomposition in dynamic vacuum (~1×10^{-3} mbar).*

^{1}H NMR (500.1 MHz, C_6D_6, 298 K): δ = 0.69 (s, $^{3}J_{(H-H)}$ = 6.3 Hz, 6 H, Al-DIC-CH(CH_3)$_2$), 0.91 (d, $^{3}J_{(H-H)}$ = 6.3 Hz, 6 H, Al-DIC-CH(CH_3)$_2$), 1.00 (s, 3 H, *i*-C_5(CH_3)), 1.09 (d, $^{3}J_{(H-H)}$ = 6.2 Hz, 6 H, P-DIC-CH(CH_3)$_2$), 1.14 (d, $^{3}J_{(H-H)}$ = 6.2 Hz, 6 H, P-DIC-CH(CH_3)$_2$), 1.26 (d, $^{3}J_{(H-H)}$ = 6.7 Hz, 12 H, CH(CH_3)$_2$), 1.43 (s, 6 H, *m*-C_5(CH_3)$_2$), 1.52 (s, 6 H, *o*-C_5(CH_3)$_2$), 1.67 (d, $^{3}J_{(H-H)}$ = 6.7 Hz, 12 H, CH(CH_3)$_2$), 2.95 (hept, $^{3}J_{(H-H)}$ = 6.4 Hz, 1 H, Al-DIC-CH(CH$_3$)$_2$), 3.42 (dhept, $^{3}J_{(H-H)}$ = 6.7 Hz, $^{1}J_{(H-P)}$ = 1 Hz, 4 H CH(CH$_3$)$_2$), 3.78 (hept, $^{3}J_{(H-H)}$ = 6.3 Hz, 1 H, Al-DIC-CH(CH$_3$)$_2$), 3.91 (dhept, $^{3}J_{(H-H)}$ = 6.2 Hz, $^{4}J_{(H-P)}$ = 2.6 Hz, 2 H, P-DIC-(CH(CH$_3$)$_2$)$_2$), 7.21 (t, $^{3}J_{(H-H)}$ = 7.5 Hz, 1 H, *p-H* (^{Dipp}Ter)), 7.32 (m, 6 H, *p-/m-H* (Dipp)), 7.38 (dd, $^{3}J_{(H-H)}$ = 7.5 Hz, $^{4}J_{(H-P)}$ = 1.7 Hz, 2 H, *m-H* (^{Dipp}Ter)) ppm.

^{13}C{^{1}H} NMR (125.8 MHz, C_6D_6, 298 K): δ = 10.6 (s, *o*-C_5(CH$_3$)$_5$), 10.8 (s, *m*-C_5(CH$_3$)$_5$), 23.1 (s, *i*-C_5(CH$_3$)$_5$), 24.8 (d, $J_{(C-P)}$ = 3 Hz, CH(CH$_3$)$_2$), 25.4 (s, Al-DIC-CH(CH$_3$)$_2$), 26.2 (d, $J_{(C-P)}$ = 2 Hz, P-DIC-CH(CH$_3$)$_2$), 26.4 (d, $J_{(C-P)}$ = 2 Hz, P-DIC-CH(CH$_3$)$_2$), 26.7 (s, Al-DIC-CH(CH$_3$)$_2$), 26.8 (s, CH(CH$_3$)$_2$), 31.4 (d, $J_{(C-P)}$ = 5.1 Hz, CH(CH$_3$)$_2$), 43.7 (s, Al-DIC-CH(CH$_3$)$_2$), 45.4 (s, Al-DIC-CH(CH$_3$)$_2$), 46.3 (d, $J_{(C-P)}$ = 10.3 Hz, P-DIC-CH(CH$_3$)$_2$), 64.4 (s, *i*-C_5(CH$_3$)$_5$), 122.9 (s, *m*-CH (Dip)), 124.1 (s, CH, *p*-CH (^{Dip}Ter)), 127.4 (s, *p*-CH (Dip)), 130.8 (s, *m*-CH (^{Dip}Ter)), 136.9 (s, *m*-C_5(CH$_3$)$_5$), 140.9 (s, *o*-C_5(CH$_3$)$_5$), 143.1 (d, $J_{(C-P)}$ = 1.7 Hz, *i-C* (Dip)), 146.75 (d, $^{1}J_{(C-P)}$ = 62 Hz, *i-C* (^{Dip}Ter)), 147.3 (s, *o-C* (Dip)), 147.6 (d, $J_{(C-P)}$ = 10.2 Hz, *o-C* (^{Dip}Ter)), 179.6 (s, Al-DIC-C), 200.2 (d, $^{1}J_{(C=P)}$ = 102 Hz, P-DIC-C) ppm.

^{31}P{^{1}H} NMR (162.2 MHz, C_6D_6, 298 K): δ = −14.32 (s) ppm.

IR (ATR, 24 scans, cm^{-1}): $\tilde{\nu}$ = 3058 (w), 2964 (s), 2926 (m), 2868 (m), 1459 (m), 1440 (m), 1412 (s), 1382 (s), 1360 (m), 1323 (s), 1256 (s), 1231 (s), 1219 (s), 1171 (w), 1139 (m), 1121 (m), 1056 (s), 1004 (w), 946 (w), 892 (w), 806 (m), 791 (m), 775 (m), 751 (s), 699 (w), 685 (m), 656 (m), 614 (w), 602 (w), 588 (w), 517 (w), 473 (w), 453 (w).

LIFDI-MS: (m/z) Calc: 842.5936, 842.5969, 844.6003 [$C_{54}H_{80}AlN_4P$]; Found: 643.4728 [M−Cp*−*i*Pr−CH$_3$+6H], 573.3948 [M−(N*i*Pr)$_2$CCp*−CH$_3$+6H], 263.2480 [M−^{Dip}Ter−PC(N*i*Pr)$_2$Al]$^+$.

Figure S36: ^{1}H NMR spectrum of **6** (500.1 MHz, C_6D_6, 298 K); 0.12 ppm 0.5 eq. HMDSO.

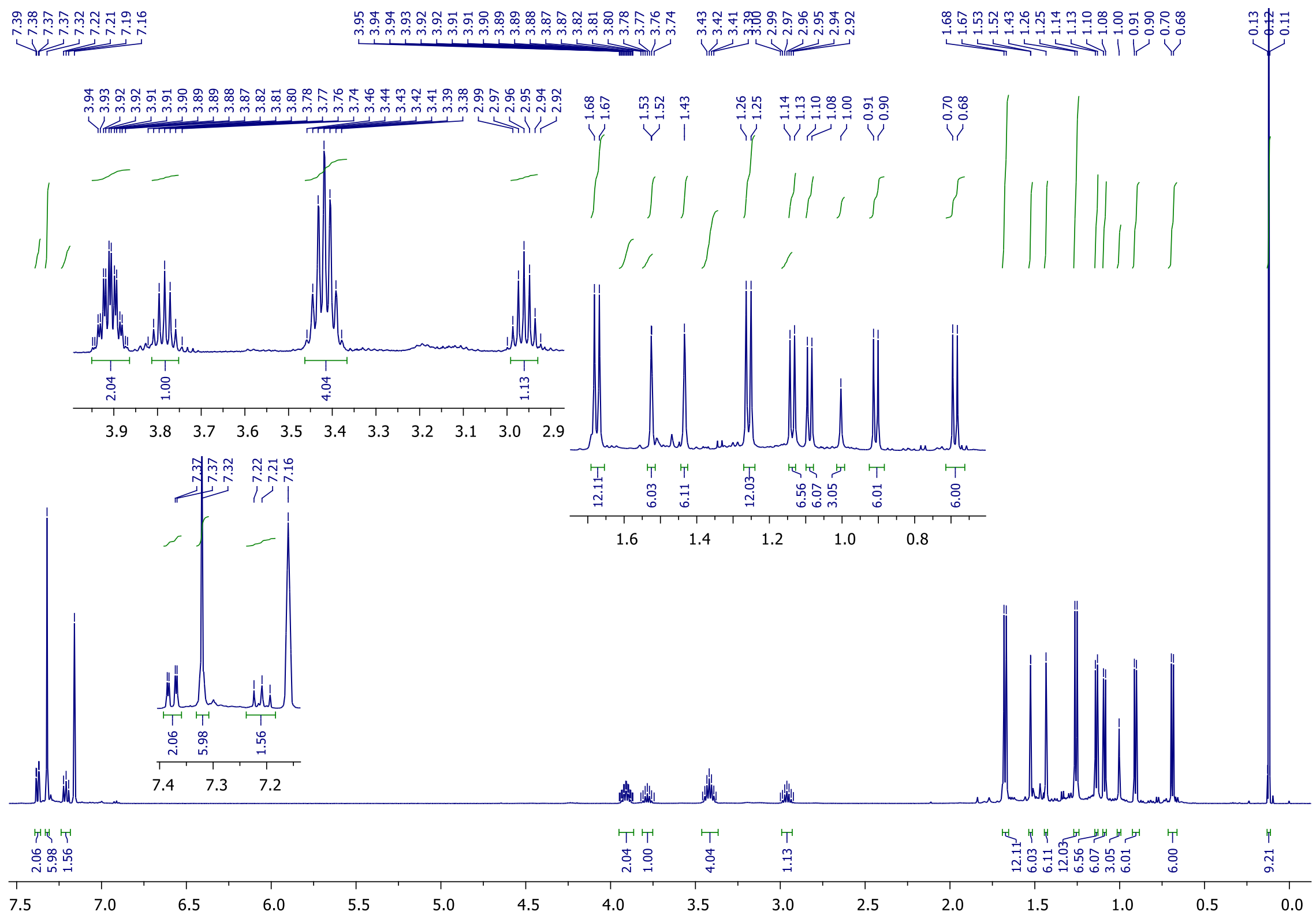

Figure S37: $^{13}C\{^{1}H\}$ NMR spectrum of **6** (125.8 MHz, C_6D_6, 298 K); 2.07 ppm HMDSO.

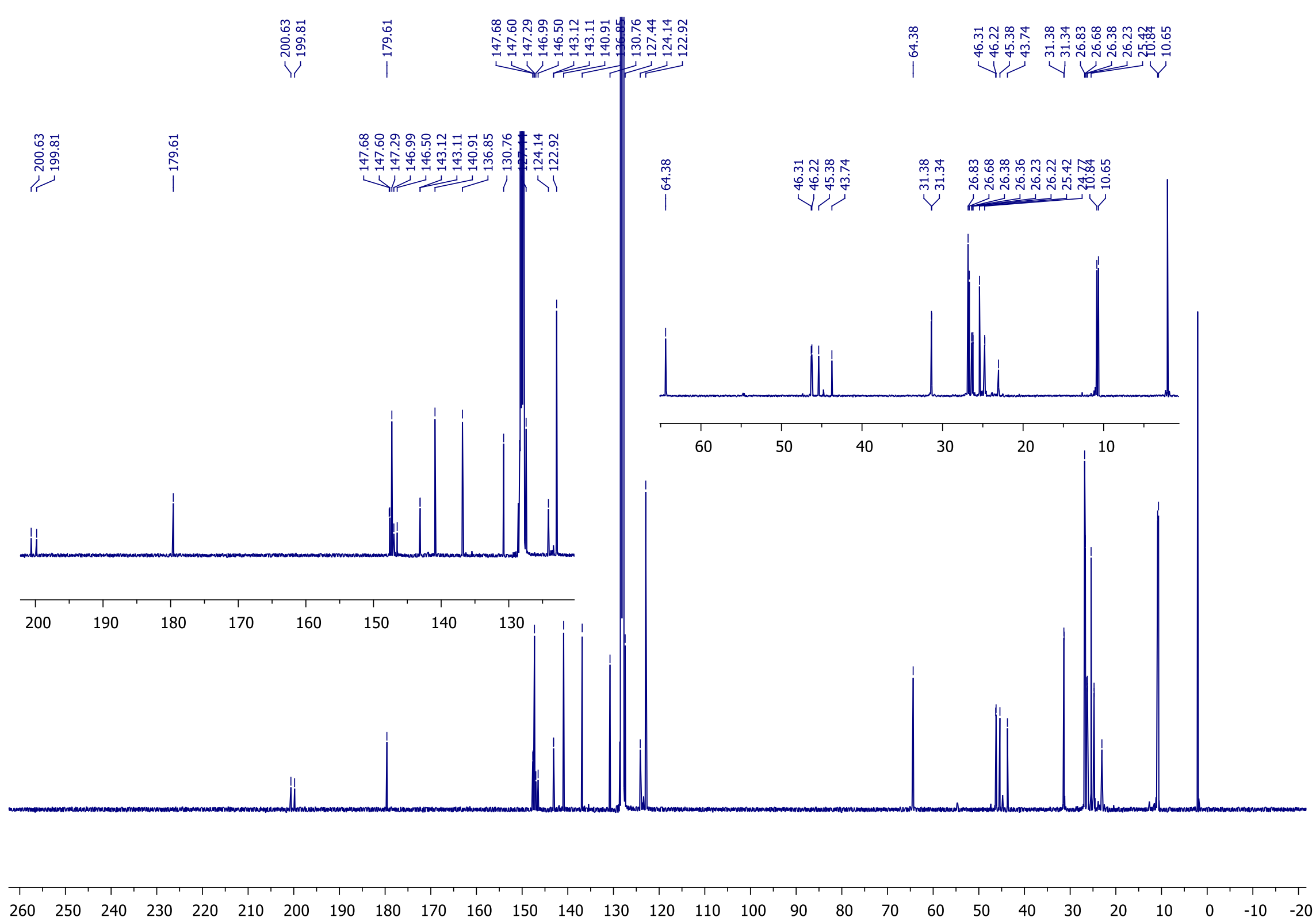

Figure S38: $^{31}P\{^{1}H\}$ NMR spectrum of **6** (162.2 MHz, C_6D_6, 298 K).

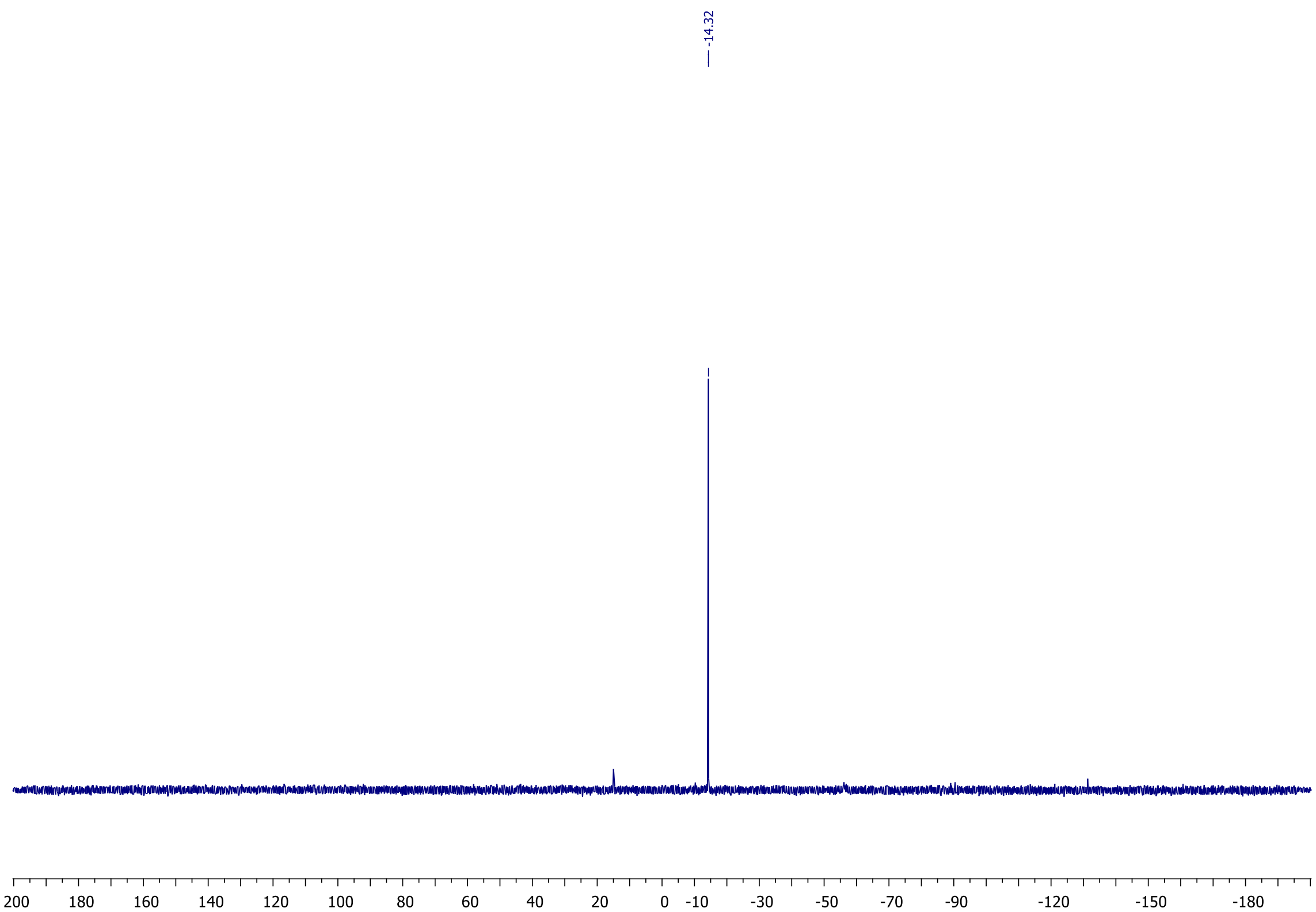

Figure S39: IR spectrum of **6** (ATR, 64 scans, cm^{-1}); 2400 – 2245, 497 cm^{-1} C_6D_6.

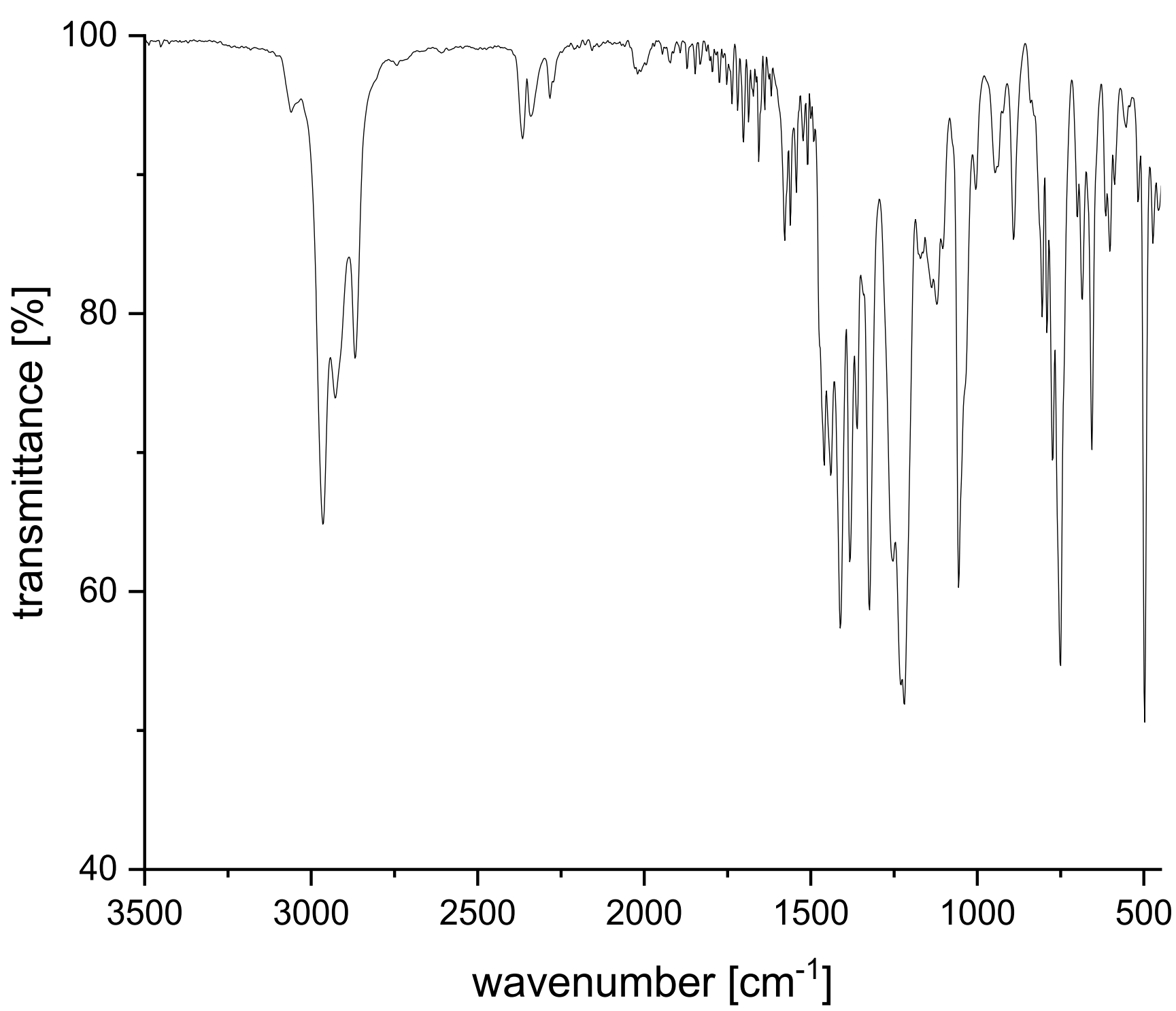

Figure S40: LIFDI mass spectrum of **6** (toluene).

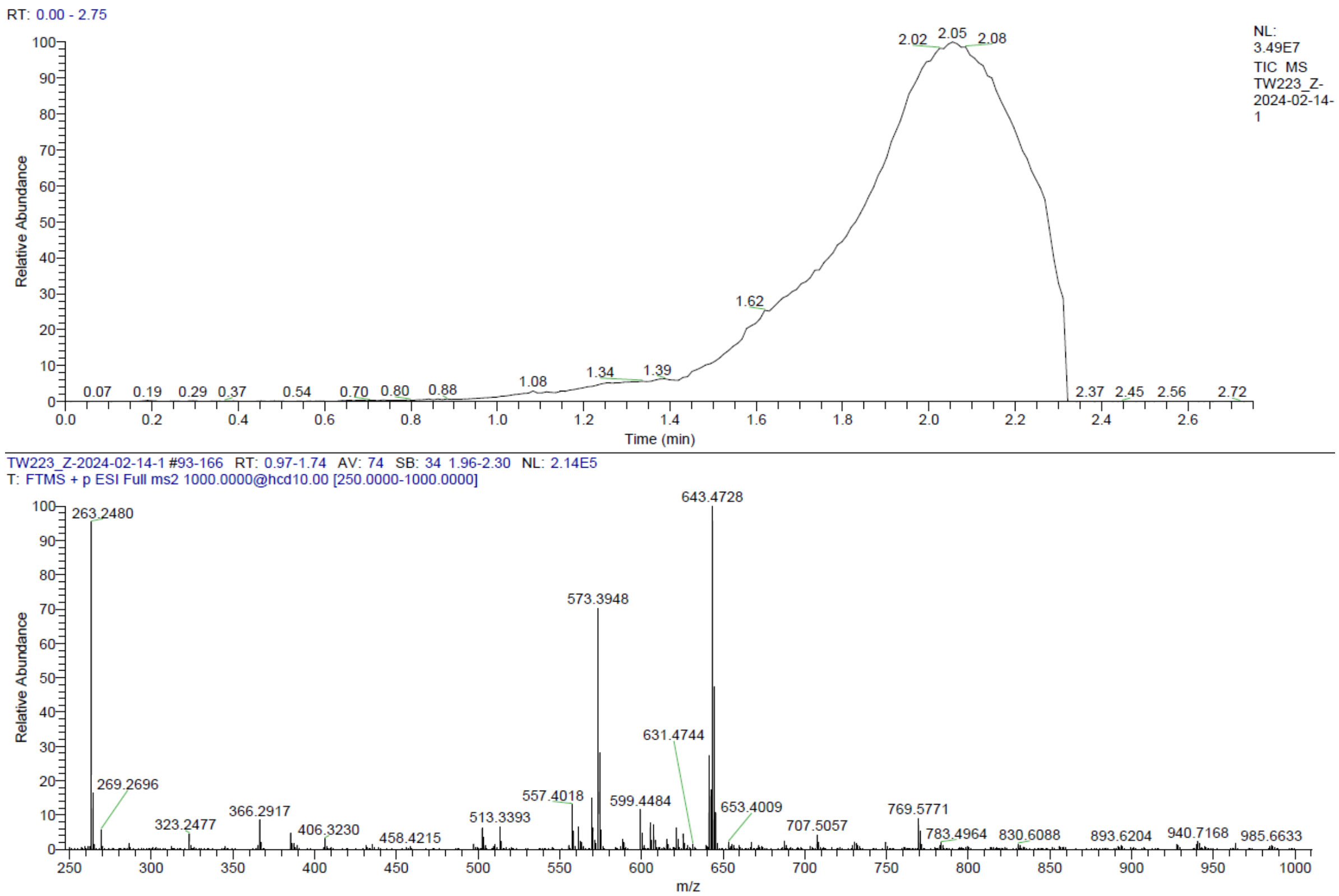

3.6. Synthesis of **7**

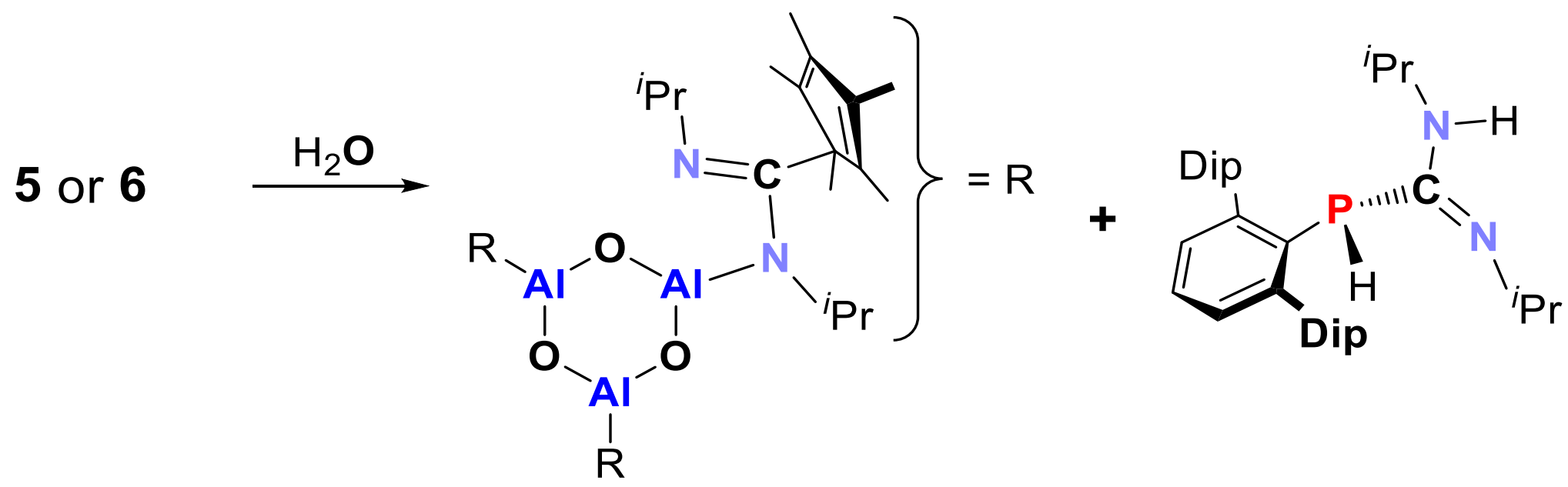

Method 1

DipTerPAlCp* (30.0 mg, 50.8 *µ*mol, 1.00 eq.) was dissolved in toluene (0.4 mL) in a *J.-Young* NMR tube and *N*,*N'*-Di(propan-2-yl)methanediimine (15.0 mg, 119 µmol, 2.30 eq.) was added at ambient temperature. Immediately the formation of a pale yellow solution was observed. Full conversion of **DipTerPAlCp*** to **5** was confirmed via $^{31}P\{^{1}H\}$ NMR spectroscopy. Subsequently the reaction mixture was heated for 5 days at 100 °C (oil bath) and full conversion of **5** to **6** was confirmed via $^{31}P\{^{1}H\}$ NMR spectroscopy. All volatile compounds were removed *in vacuo* (~1×10^{-3} mbar) and the orange solid was heated for 8 hours *in vacuo* (~1×10^{-3} mbar) at 80 °C (oil bath). The resulting yellow powder was washed with hmdso (2 × 0.2 mL) and dried at ambient temperature *in vacuo* (~1×10^{-3} mbar).

Yield of 7 (**7-P** and **7-Al**)**:** 7.00 mg (18.5 *µ*mol, 36%) of a yellow powder.

Suitable crystals for single crystal X-ray diffraction were obtained by slow evaporation of a saturated solution of **7-P** in *n*-hexane at ambient temperature in a glovebox with silicon grease as solvent absorbent.

Method 2

Direct Hydrolysis of **5** and **6** with a drop of a water/thf solution led to the formation of the phosphaguanidine derivative **7-P**.

^{1}H NMR (500.1 MHz, C_6D_6, 298 K, **7-P**): δ = 0.87 (d, $^3J_{(H-H)}$ = 6.1 Hz, 3 H, C(H)(CH_3)(CH_3), (N-*i*Pr) (group A))[10], 0.90 (d, $^3J_{(H-H)}$ = 6.1 Hz, 3 H, C(H)(CH_3)(CH_3), (N-*i*Pr) (group A)), 0.97 (d, $^3J_{(H-H)}$ = 6.7 Hz, 3 H, C(H)(CH_3)(CH_3), (N-*i*Pr) (group B)), 1.05 (d, $^3J_{(H-H)}$ = 6.7 Hz, 3 H, C(H)(CH_3)(CH_3), (N-*i*Pr) (group B)), 1.09 (superimposed d, $^3J_{(H-H)}$ = 6.9 Hz, 12 H, C(H)(CH_3)(CH_3), (Dip) (group C + D)), 1.38 (d, $^3J_{(H-H)}$ = 6.9 Hz, 6 H, C(H)(CH_3)(CH_3), (Dip) (group C)), 1.43 (d, $^3J_{(H-H)}$ = 6.9 Hz, 6 H, C(H)(CH_3)(CH_3), (Dip) (group D)), 2.79 (hept, $^3J_{(H-H)}$ = 6.9 Hz, 2 H, C(*H*)(CH_3)(CH_3), (Dipp) (group C)), 2.92 (hept, $^3J_{(H-H)}$ = 6.9 Hz, 2 H, C(*H*)(CH_3)(CH_3), (Dip) (group D)), 3.33 (hept, $^3J_{(H-H)}$ = 6.1 Hz, 1 H, C(*H*)(CH_3)(CH_3), (N-*i*Pr) (group A)), 3.59 (d, $^3J_{(H-H)}$ = 6.6 Hz, 1 H, N*H* (group B)), 4.19 (hept, $^3J_{(H-H)}$ = 6.7 Hz, 1 H, C(*H*)(CH_3)(CH_3), (N-*i*Pr) (group B)), 4.42 (d, $^1J_{(H-P)}$ = 224.0 Hz, 1 H, P*H*). 7.03 – 7.07 (m, 1 H, *p-H* (^{Dip}Ter)), 7.11 – 7.14 (m, 2 H, *m-H* (^{Dip}Ter)), 7.18 – 7.24 (m, 4 H, *m-H* (Dip)), 7.29 – 7.34 (m, 4 H, *p-H* (Dip)) ppm.

^{13}C{^{1}H} NMR (125.8 MHz, C_6D_6, 298 K, **7-P**): δ = 21.8 (s, C(H)(CH_3)(*C*H_3), (N-*i*Pr) (group B)), 22.0 (s, C(H)(*C*H_3)(CH_3), (N-*i*Pr) (group B)), 23.6 (d, $J_{(C-P)}$ = 2 Hz, C(H)(CH_3)(*C*H_3), (Dipp) (group C)), 23.96 (d, $J_{(C-P)}$ = 2 Hz, C(H)(CH_3)(*C*H_3), (Dip) (group D)), 25.2 (s, C(H)(*C*H_3)(CH_3), (N-*i*Pr) (group A)), 25.62 (d, $J_{(C-P)}$ = 1 Hz, C(H)(CH_3)(*C*H_3), (N-*i*Pr) (group A)), 26.0 (s, C(H)(CH_3)(CH_3), (Dip) (group C or D)), 26.2 (s, C(H)(CH_3)(CH_3), Dip (group C or D)), 31.4 (s, *C*(H)(CH_3)(CH_3), (Dip) (group C)), 31.4 (d, $J_{(C-P)}$ = 3 Hz, C(*H*)(CH_3)(CH_3), (Dip) (group D)), 43.0 (s, *C*(H)(CH_3)(CH_3), (N-*i*Pr) (group B)), 52.8 (d, $J_{(C-P)}$ = 35 Hz, *C*(H)(CH_3)(CH_3), (N-*i*Pr) (group A)), 123.6 (d, $J_{(C-P)}$ = 12 Hz, *m*-*C*H (Dip)), 127.8 (s, *p*-*C*H (^{Dip}Ter)), 129.1 (s, *p*-*C*H (Dip)), 130.8 (d, $J_{(C-P)}$ = 2 Hz, *m*-*C*H (^{Dip}Ter)), 134.4 (d, $^1J_{(C-P)}$ = 18 Hz, *i-C* (^{Dip}Ter)), 139.3 (d, $J_{(C-P)}$ = 2 Hz, *i-C* (Dip)), 146.0 (d, $J_{(C-P)}$ = 12 Hz, *o-C* (^{Dip}Ter)), 146.3 (s, *o-C* (Dip)), 147.5 (s, *o-C* (Dip)), 150.0 (d, $^1J_{(C-P)}$ = 37 Hz, N_2*C*) ppm.

^{31}P{^{1}H} NMR (202.5 MHz, C_6D_6, 298 K): δ = −89.7 (s) ppm.

^{31}P NMR (202.5 MHz, C_6D_6, 298 K): δ = −89.7 (dd, $^1J_{(P-H)}$ = 224 Hz, $^3J_{(P-H)}$ = 7 Hz) ppm.

---

[10] Due to the similarity of the ligands, groups have been used in this case to make the assignment easier to understand. The groups A to D are used to assign the signals of the isopropyl groups. The isopropyl groups A and B belongs to the amidine unit and C and D to the Dip group.

^{1}H NMR (500.1 MHz, C_6D_6, 298 K, **7-Al**): δ = 0.78 (d, $^3J_{(H\text{-}H)}$ = 6.1 Hz, 6 H, C(H)(CH_3)$_2$, (N-*i*Pr) (<u>group A</u>)), 1.34 (d, $^3J_{(H\text{-}H)}$ = 6.1 Hz, 6 H, C(H)(CH_3)$_2$, (N-*i*Pr) (<u>group B</u>)), 1.47 (s, 3 H, C_5((CH_3)(CH_3)$_2$(CH_3)$_2$)), 1.69 (d, $^3J_{(H\text{-}H)}$ = 0.8 Hz, 6 H, C_5((CH_3)(CH_3)$_2$(CH_3)$_2$)), 1.84 (d, $^3J_{(H\text{-}H)}$ = 0.8 Hz, 6 H, C_5((CH_3)(CH_3)$_2$(CH_3)$_2$)), 3.35 – 3.41 (m, 1 H, C(*H*)(CH_3)$_2$, (N-*i*Pr) (<u>group A</u>)), 3.73 (hept, $^3J_{(H\text{-}H)}$ = 6.1 Hz, 1 H, C(*H*)(CH_3)$_2$, (N-*i*Pr) (group B)) ppm.

^{13}C{^{1}H} NMR (125.8 MHz, C_6D_6, 298 K, **7-Al**): δ = 10.6 (s, C_5((CH_3)(*C*H_3)$_2$(CH_3)$_2$)), 11.2 (s, C_5((CH_3)(CH_3)$_2$(*C*H_3)$_2$)), 20.5 (s, C_5((*C*H_3)(CH_3)$_2$(CH_3)$_2$)), 24.6 (s, C(H)(*C*H_3)$_2$, (N-*i*Pr) (<u>group A</u>)), 25.0 (s, C(H)(*C*H_3)$_2$, (N-*i*Pr) (<u>group B</u>)), 44.7 (s, *C*(H)(CH_3)$_2$, (N-*i*Pr) (<u>group A</u>)), 47.4 (s, *C*(H)(CH_3)$_2$, (N-*i*Pr) (<u>group B</u>)), 65.1 (s, (s, *i*-*C*$_5$(CH_3)$_5$), 135.5 (s, C_5(*C*H_3)$_5$), 141.9 (s, C_5(*C*H_3)$_5$), 153.0 (s, N_2*C*-Cp*) ppm.

LIFDI-MS (toluene): m/z calc: 556.3946 (100.0%) [$C_{37}H_{53}N_2P$] and 912.6285 [$C_{51}H_{87}Al_3N_6O_3$]; m/z found: 557.4017 $[M]^+$ and 912.9763 [$C_{51}H_{87}Al_3N_6O_3$].

Figure S41: ^{1}H NMR spectrum of **7** (500.1 MHz, C_6D_6, 298 K); 0.12 ppm: HMDSO. Marked Signals corresponds to **7-P**.

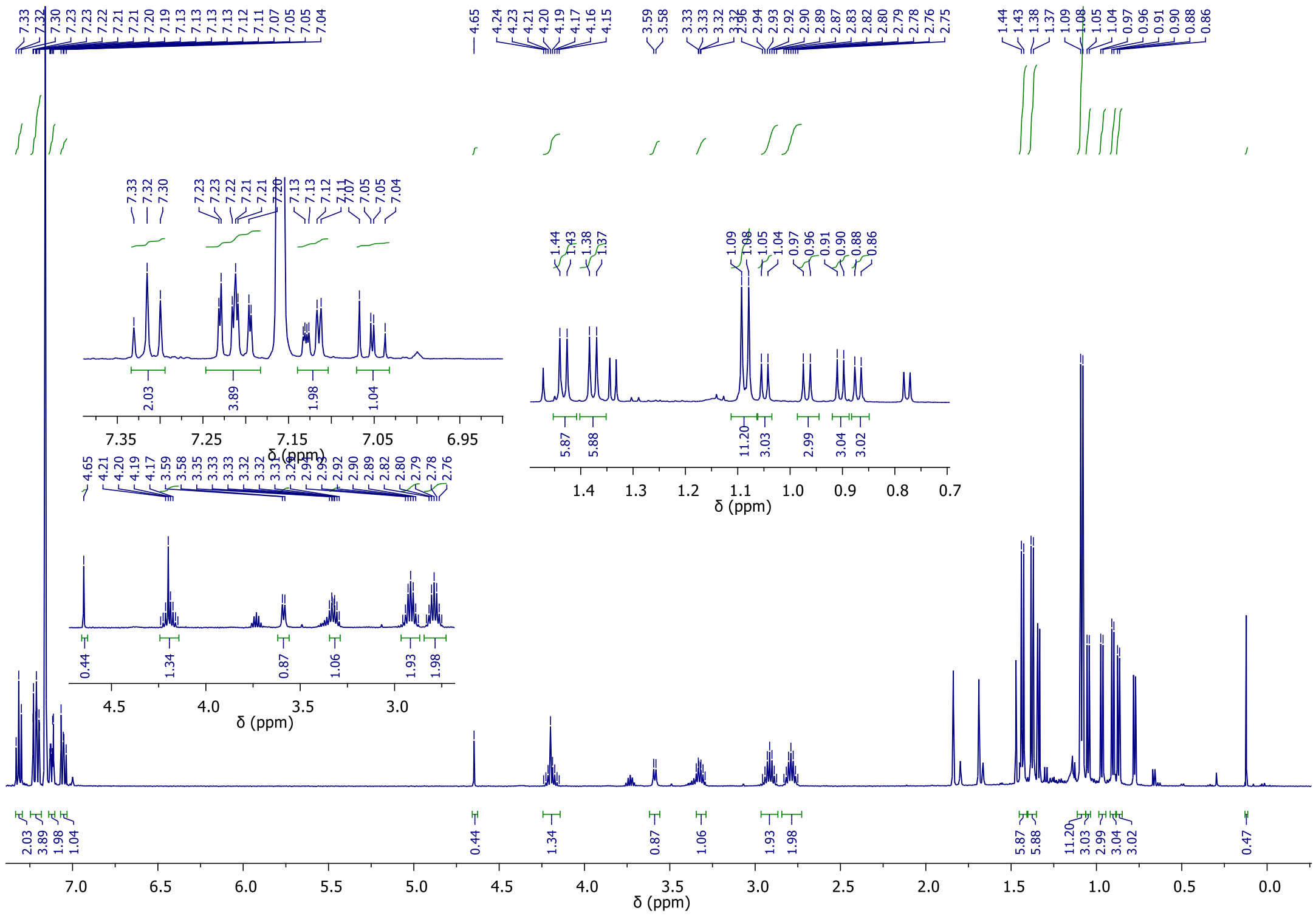

Figure S42: $^{13}C\{^{1}H\}$ NMR spectrum of **7** (125.8 MHz, C_6D_6, 298 K); 2.07 ppm: HMDSO. Marked Signals corresponds to **7-P**.

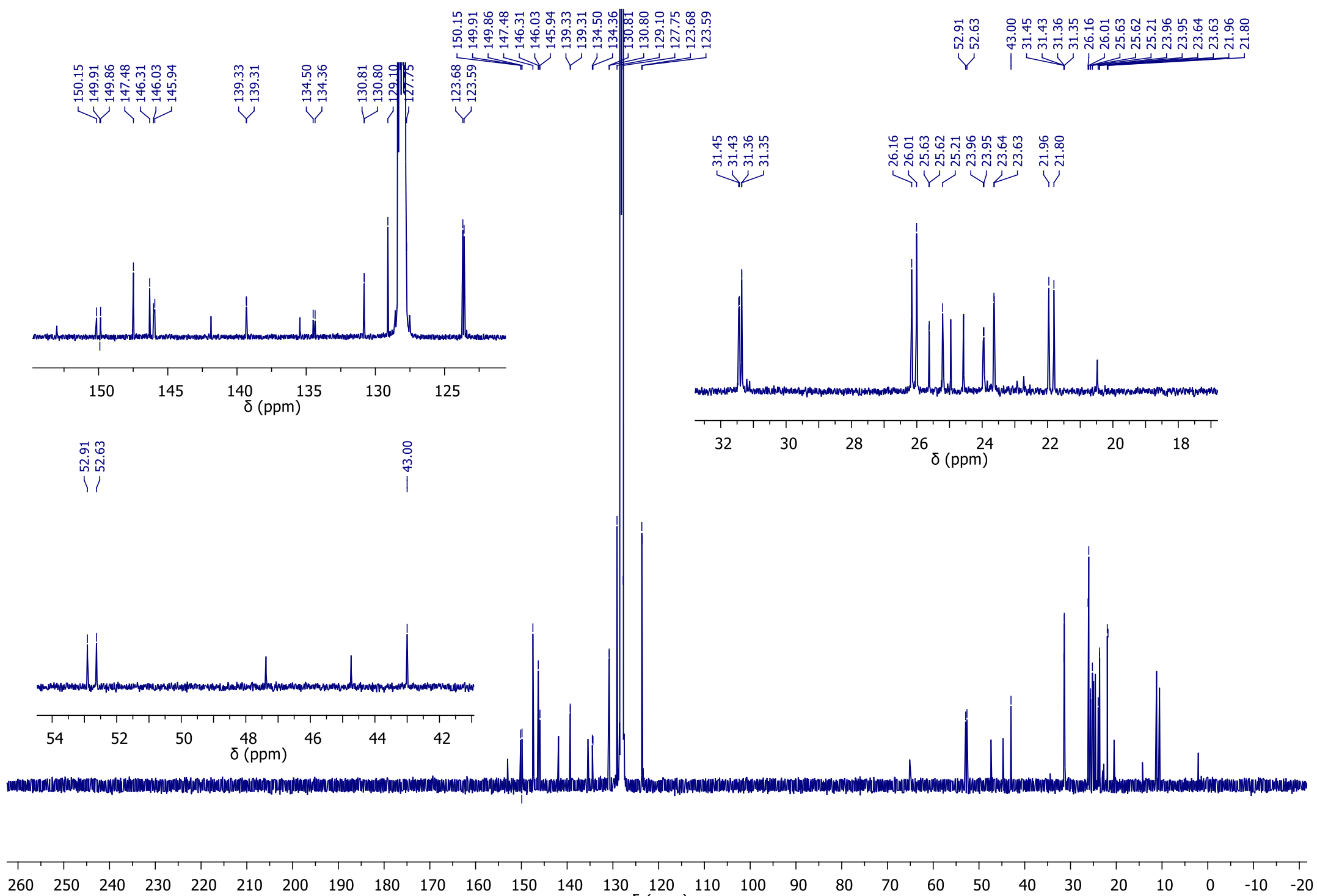

Figure S43: $^{31}P\{^{1}H\}$ NMR spectrum of **7** (202.5 MHz, C_6D_6, 298 K).

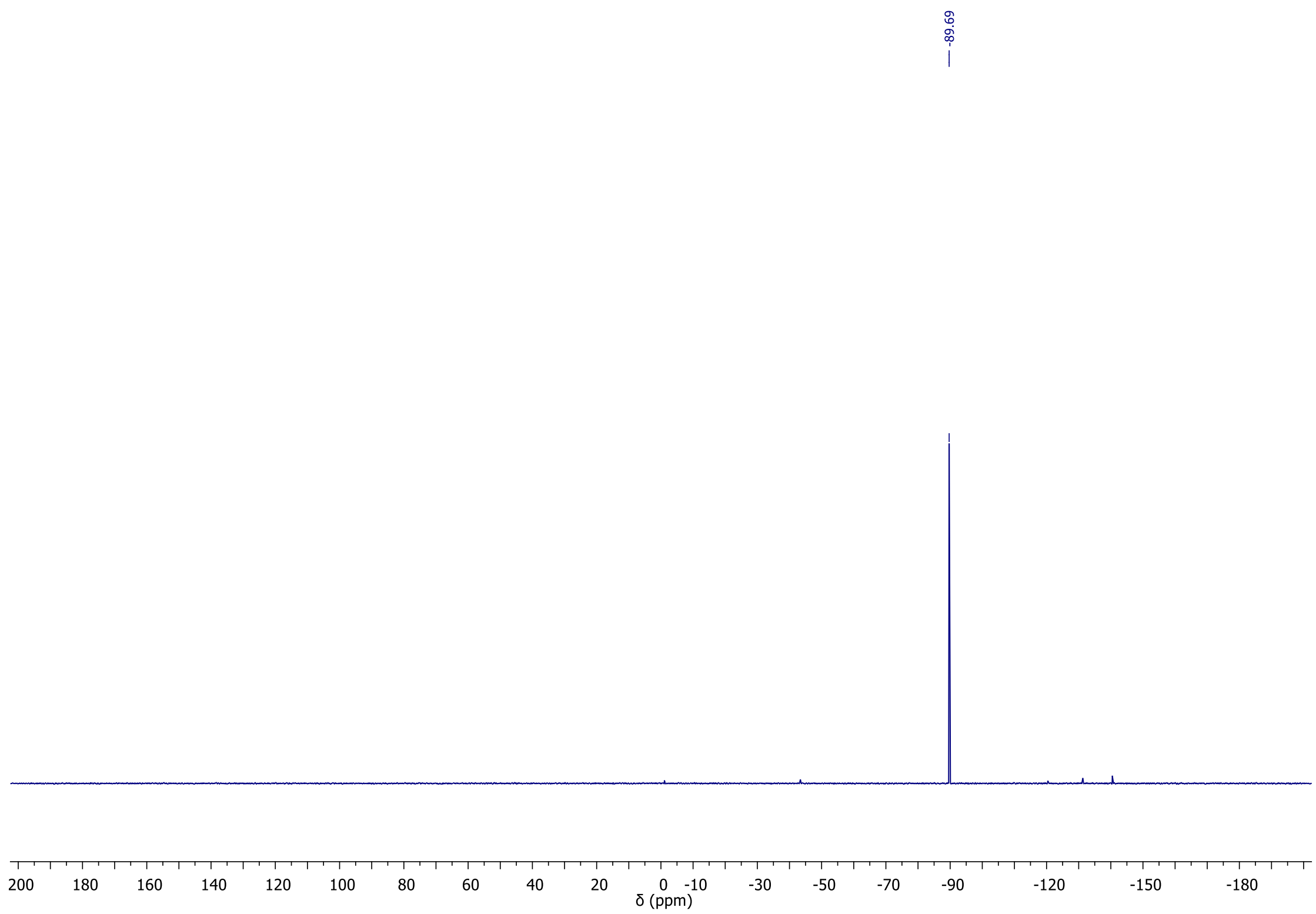

Figure S44: ^{31}P NMR spectrum of **7** (202.5 MHz, C_6D_6, 298 K).

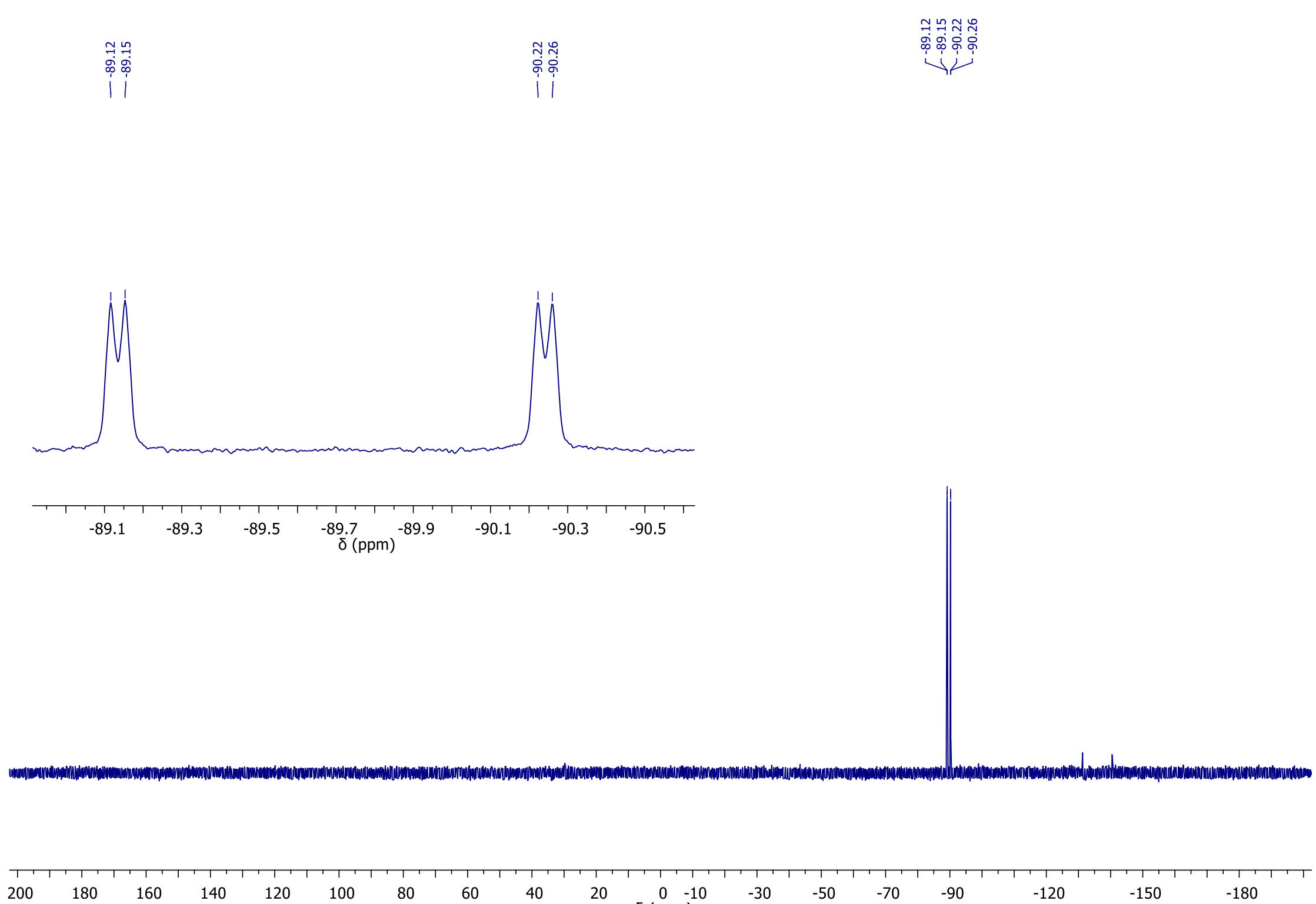

Figure S45: ^{1}H NMR spectrum of **7** (500.1 MHz, C_6D_6, 298 K); 0.12 ppm: HMDSO. Marked Signals corresponds to **7-Al**.

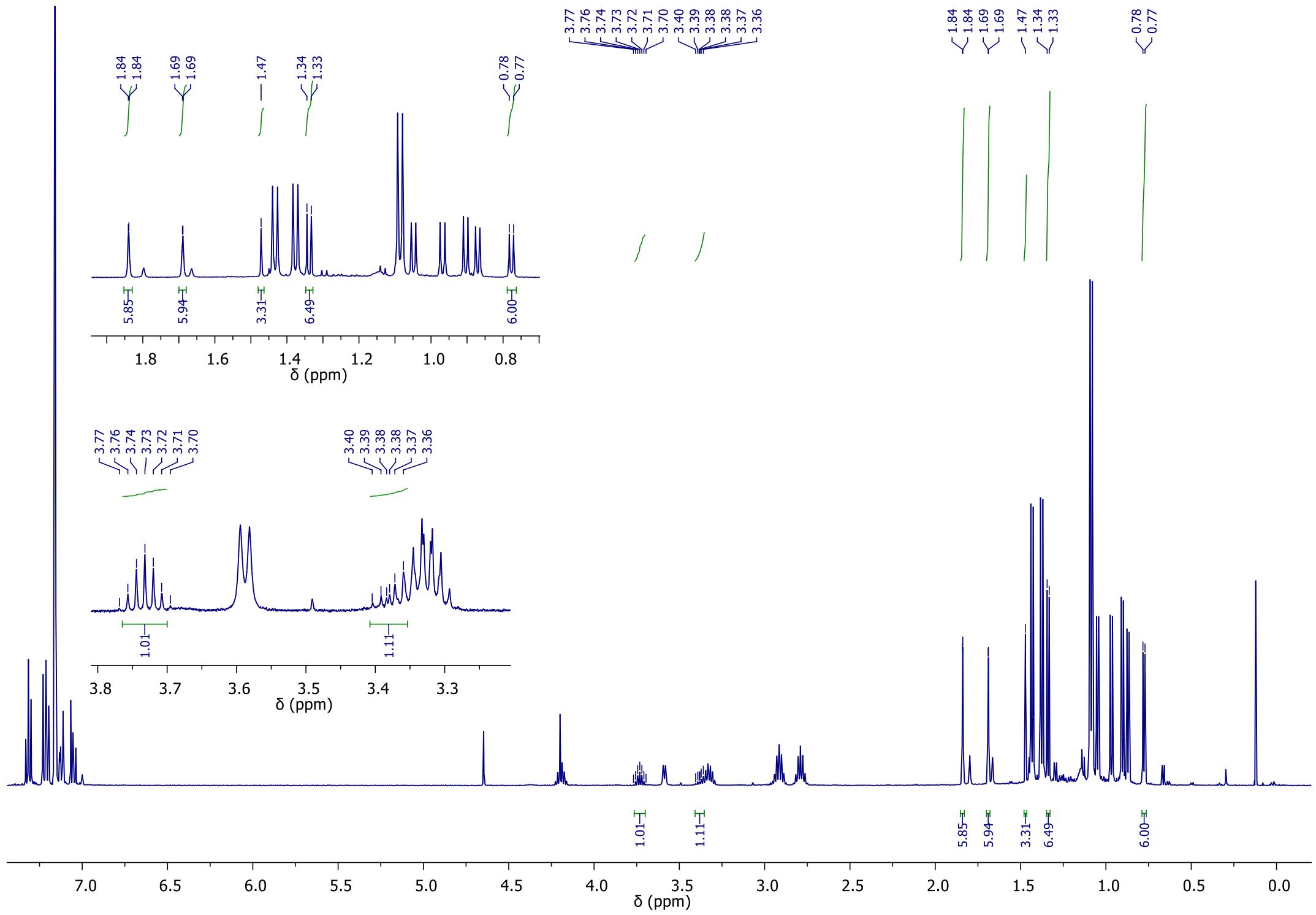

Figure S46: $^{13}C\{^{1}H\}$ NMR spectrum of **7** (125.8 MHz, C_6D_6, 298 K); 2.07 ppm: HMDSO. Marked Signals corresponds to **7-Al**.

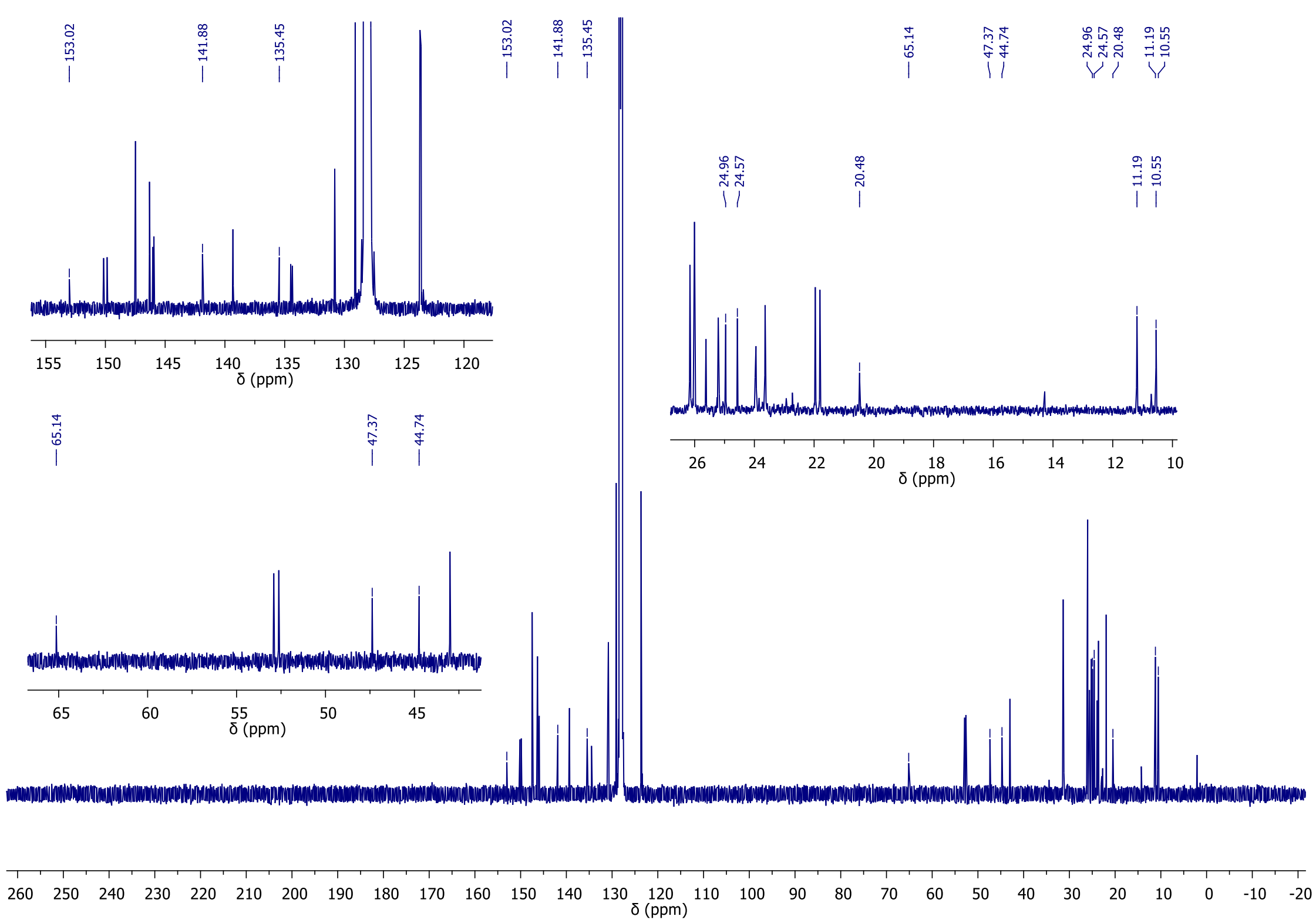

Figure S47: LIFDI mass spectrum of **7** (toluene).

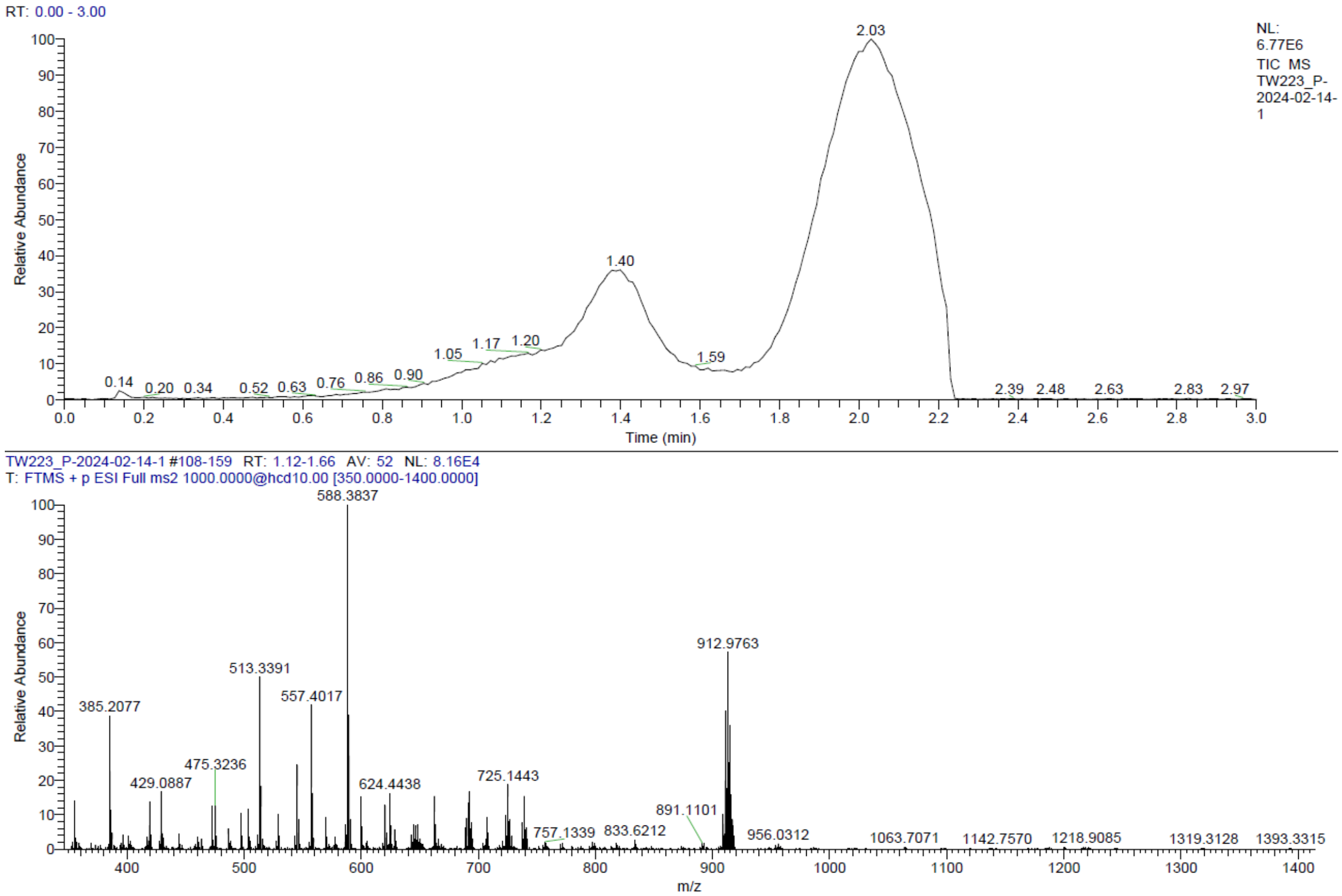

4. Reactivity of **3** and additional substrate scope

4.1. Isolation of [^{Dip}TerPCO]$_2$ (**2dim**).

^{Dip}TerPAlCp* (85 mg, 144 µmol, 1.00 eq.) was dissolved in *n*-pentane (5 mL) in a Schlenk tube. The purple solution was degassed, and CO_2 (1.4 atm) was added at ambient temperature. After 2 min the cloudy orange solution was filtered through a canula fitted with a glass filter paper into another Schlenk tube (10 mL). This Schlenk tube was placed in the Glovebox and the valve was opened so that the *n*-pentane could slowly evaporate over a period of three weeks. This resulted in the formation of a sticky brown solid. To this solid *n*-hexane was added (2 x 3 mL) and the supernatant solution was again removed by filtration. The red supernatant solution was placed in the freezer (−30 °C) for 24 h to afford pale yellow crystals of **2dim_b** (as an *n*-hexane solvate, details see Table S4) suitable for scXRD. The residue from the *n*-hexane wash is an off-white solid (ca. 10 mg), which turned out to be mainly **2dim**. Nevertheless, the sample was contaminated with minimal amounts of **2** and the diphosphene (^{Dip}TerP)$_2$. When leaving a C_6D_6 solution of **2dim** at ambient temperature and ambient light for 24 h, decomposition is observed. The main decomposition product is the diphosphene (^{Dip}TerP)$_2$,[9] while **2** and its decarbonylation product, a phosphafluorene are also detected. The NMR data of 2dim listed in the following is of those signals that could be unambiguously identified.

^{1}H NMR (400.1 MHz, C_6D_6, 298 K): δ = 0.98 (d, $^3J_{(H-H)}$ = 6.7 Hz, 12 H, CH(C*H*$_3$)(CH$_3$)), 1.31 (d, $^3J_{(H-H)}$ = 6.7 Hz, 12 H, CH(CH$_3$)(C*H*$_3$)), 2.59 (hept, $^3J_{(H-H)}$ = 6.8 Hz, 4 H, C*H*(CH$_3$)(CH$_3$)), 7.07* (d, $^3J_{(H-H)}$ = 7.8 Hz, 4 H, *m-H* (Dip)), 7.23 (t, $^3J_{(H-H)}$ = 7.8 Hz, 2 H, *p-H* (Dip)) ppm. *The signal of the meta protons of the central aryl-ring of the ^{Dip}Ter substituent overlap with this signal.

^{13}C{^{1}H} NMR (75.5 MHz, C_6D_6, 298 K): δ = 22.9 (CH(CH$_3$)(*C*H$_3$)), 26.1 (s, CH(*C*H$_3$)(CH$_3$)), 31.2 (s, *C*H(CH$_3$)(CH$_3$)), 123.0 (s, *m-C*H (Dip)), 129.5 (s, *p-C*H (Dip)), 133.2 (d, $^1J_{(C-P)}$ = 37 Hz, *i-C* (^{Dip}Ter)), 147.1 (s , *o-C* (Dip)).

^{31}P{^{1}H} NMR (162.0 MHz, C_6D_{14}, 298 K): δ = 52.4 (s) ppm.

Figure S48: ^{1}H NMR spectrum of **2dim** (400.1 MHz, C_6D_6, 298 K).

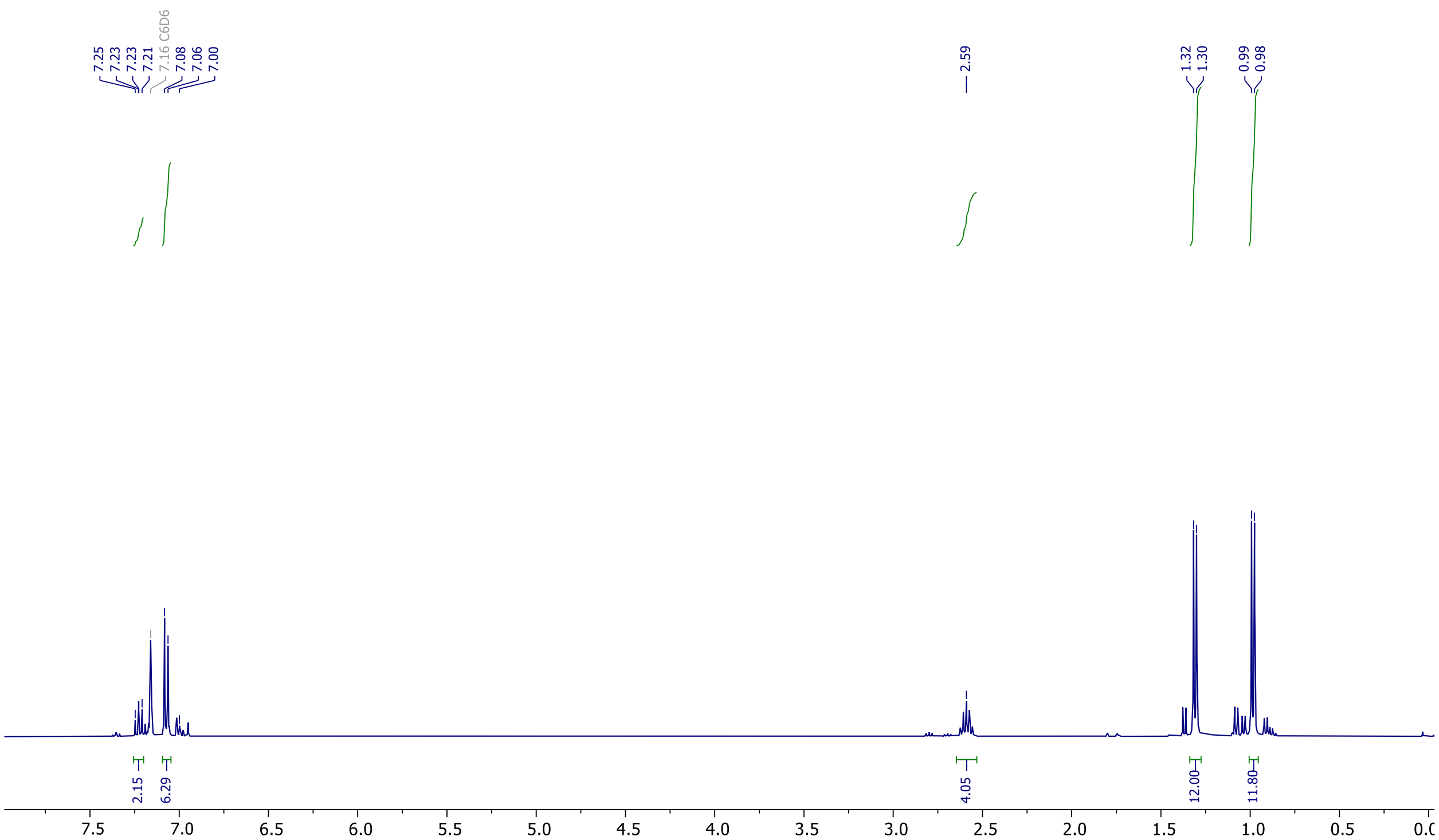

Figure S49: ^{13}C{^{1}H} NMR spectrum of **2dim** (75.5 MHz, C_6D_6, 298 K).

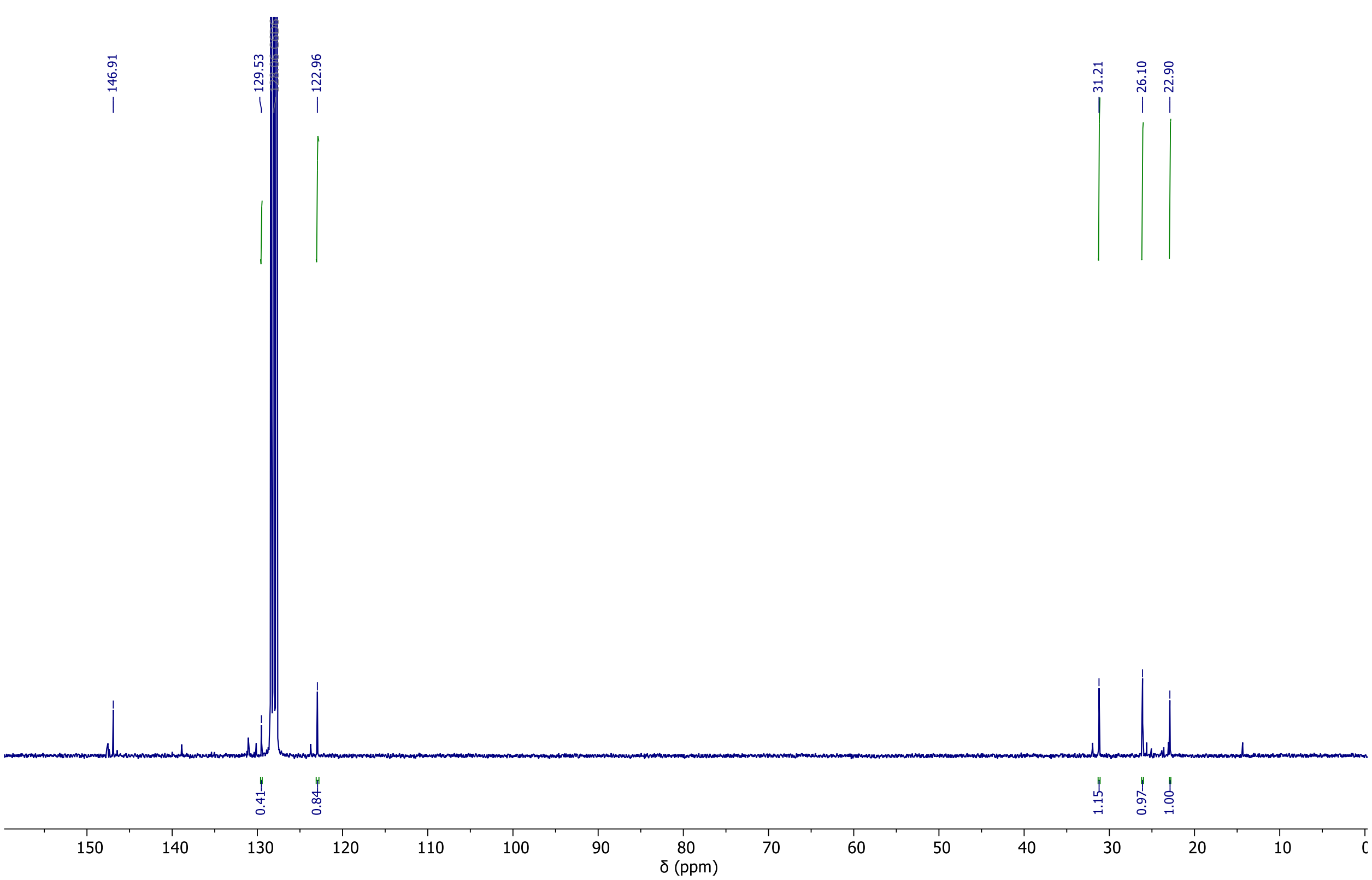

Figure S50: $^{31}P\{^1H\}$ NMR spectrum of **2dim** (162.0 MHz, C_6D_6, 298 K).

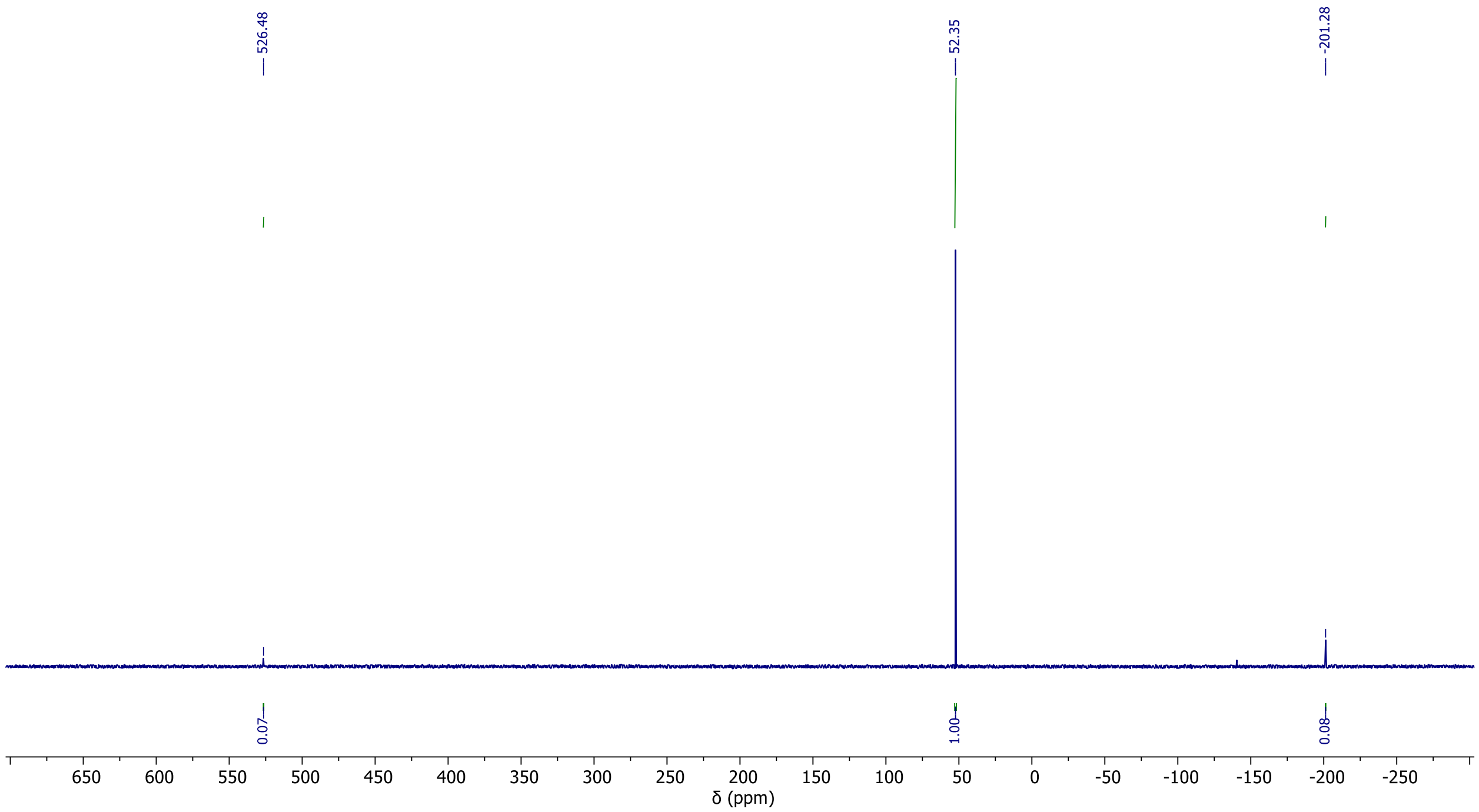

Figure S51: $^{31}P\{^1H\}$ NMR spectrum of **2dim** after standing at ambient temperature and under light for 24 h (162.1 MHz, C_6D_6, 298 K), 526.5 ppm (^{Dip}TerP)$_2$, 52.4 ppm (**2dim**), −1.1 ppm Phosphafluorene, −201.3 ppm ^{Dip}TerPCO (**2**).

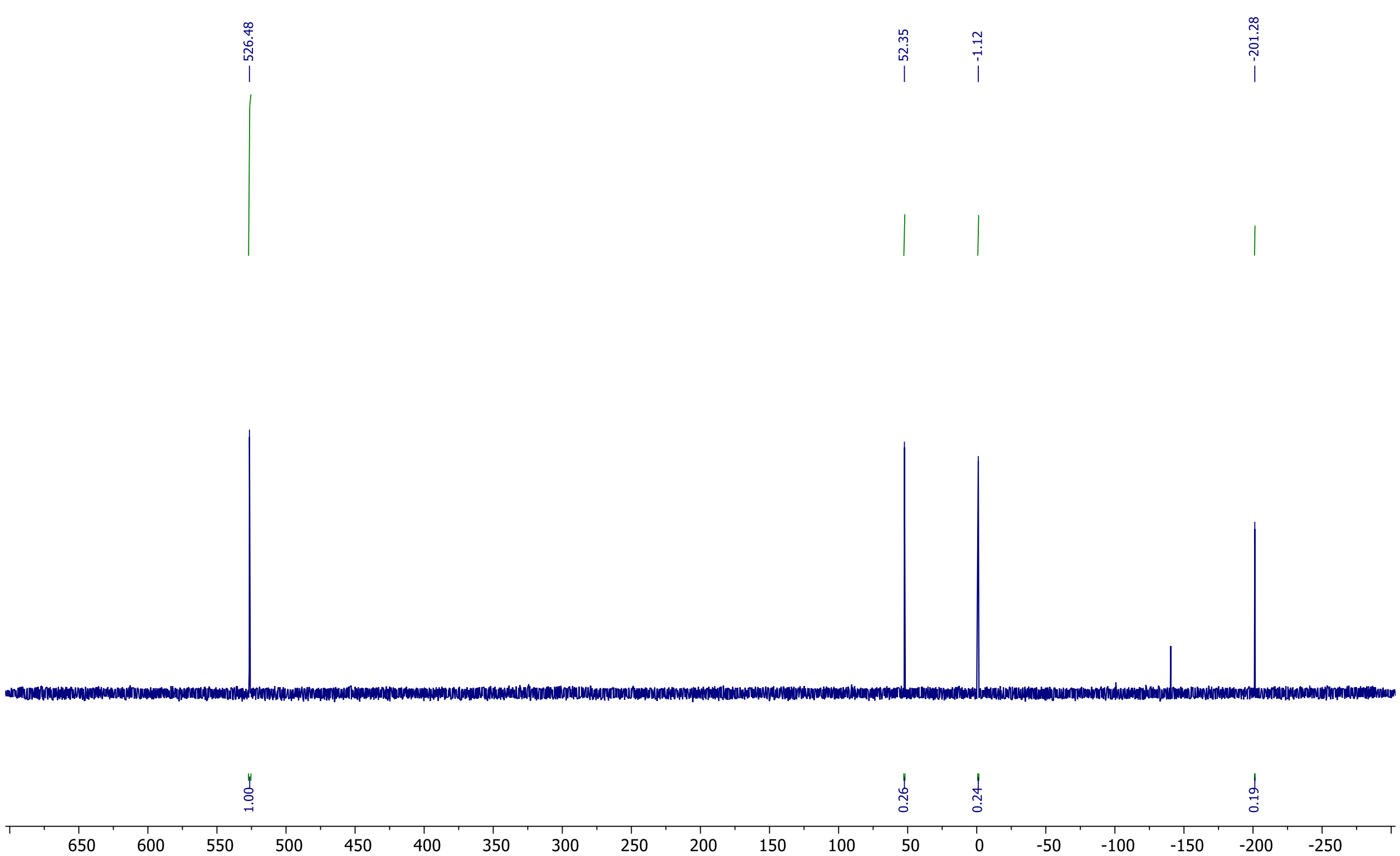

4.2. Reaction of **3** with CO_2

3 (11 mg, 33.9 μmol, 1.00 eq.) was dissolved in C_6D_6 (0.4 mL) in a *J.-Young* NMR tube and the solution was degassed and subsequently CO_2 was added (1.4 atm) over a period of 1 min. A color change from pale yellow to orange was observed. Afterwards the sample was analyzed by ^1H and ^{31}P NMR spectroscopy, showing clean conversion to ^{Dip}TerPCO (**2**).

Figure S52: Crude $^{31}P\{^1H\}$ NMR reaction spectrum of **3** with CO_2 (162.1 MHz, C_6D_6, 298 K), −27.0 ppm **3** −140.5 ppm $^{Dip}TerPH_2$, −201.3 ppm ^{Dip}TerPCO (**2**).

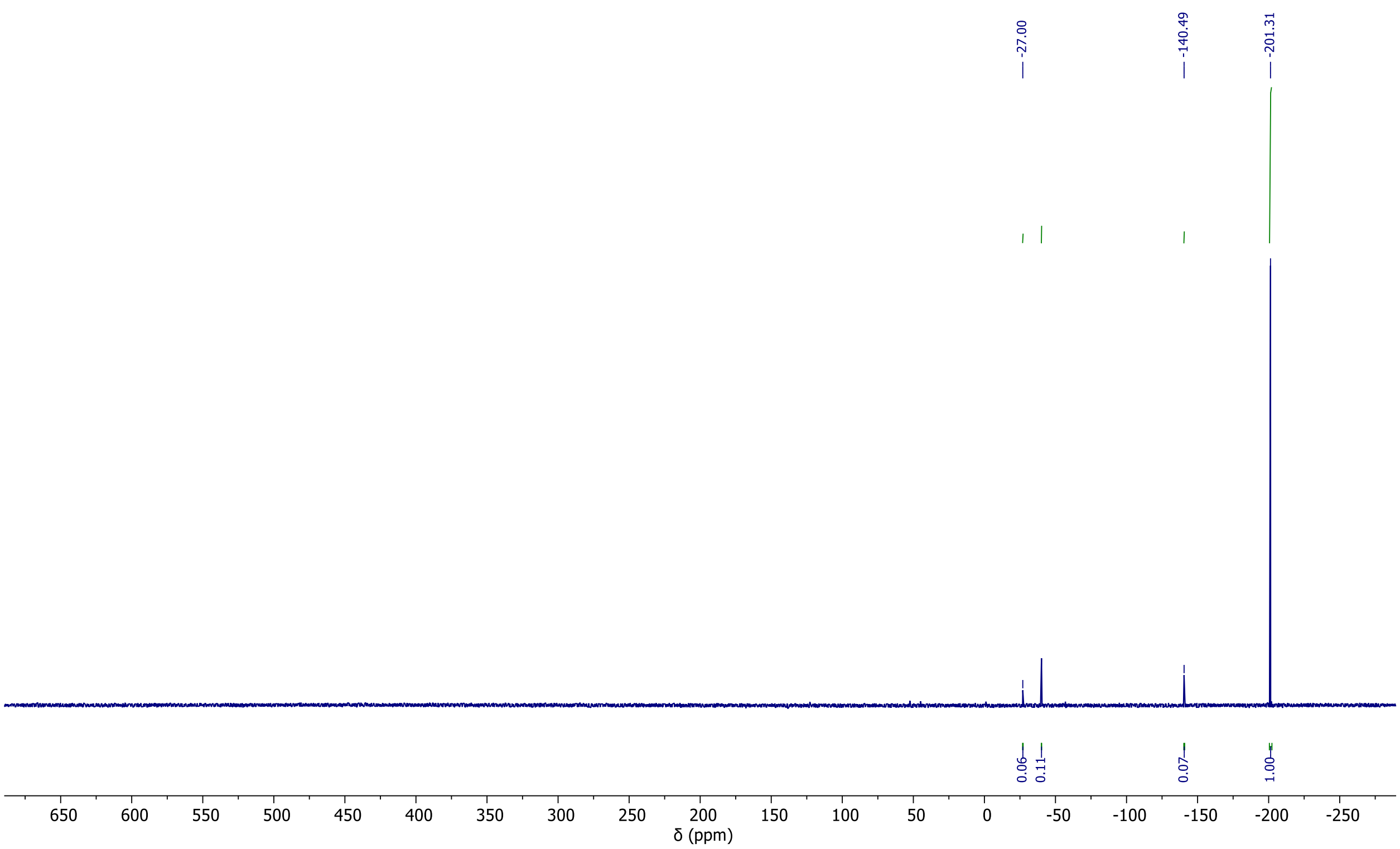

4.3. Reaction of ^{Dip}**TerPAlCp*** with DCC

^{Dip}**TerPAlCp*** (20.0 mg, 33.9 μmol, 1.00 eq.) was dissolved in C_6D_6 (0.4 mL) in a *J.-Young* NMR tube and N,N-dicyclohexylcarbodiimide (DCC) (8.00 mg, 38.8 μmol, 1.14 eq.) was added to the purple solution at ambient temperature. Immediately the formation of a pale yellow solution was observed. Full consumption of ^{Dip}**TerPAlCp*** was confirmed via $^{31}P\{^{1}H\}$ NMR spectroscopy. The two main product show rather broad ^{31}P NMR signals at −53.3 ppm (cf. **5** 56.3 ppm) and −66.9 ppm, respectively. By comparison with the ^{31}P NMR signal of **5**, the major product at −53.3 ppm was assigned to the corresponding DCC-derived species ^{Dip}TerP[C(NCy)$_2$]Al[C(NCy)$_2$]Cp*(Cy = cyclohexyl), while the signal at 6 is assumed to be the [2+2]-cyclisation product with one equivalent of DCC. Attempts to cleanly obtain one or the other have not been successful.

Afterwards all volatile components were removed *in vacuo* (~1×10^{-3} mbar). The pale-yellow residue was extracted with *n*-pentane (0.4 mL) over a pipette equipped with a glass microfiber pad. By storing a saturated *n*-pentane solution at −30 °C for 24 h only **8** was crystallized as a hydrolysis product (Figure S4).

Figure S53: Crude $^{31}P\{^{1}H\}$ NMR reaction spectrum of $^{\mathbf{Dip}}$**TerPAlCp*** with DCC (162.1 MHz, C_6D_6, 298 K).

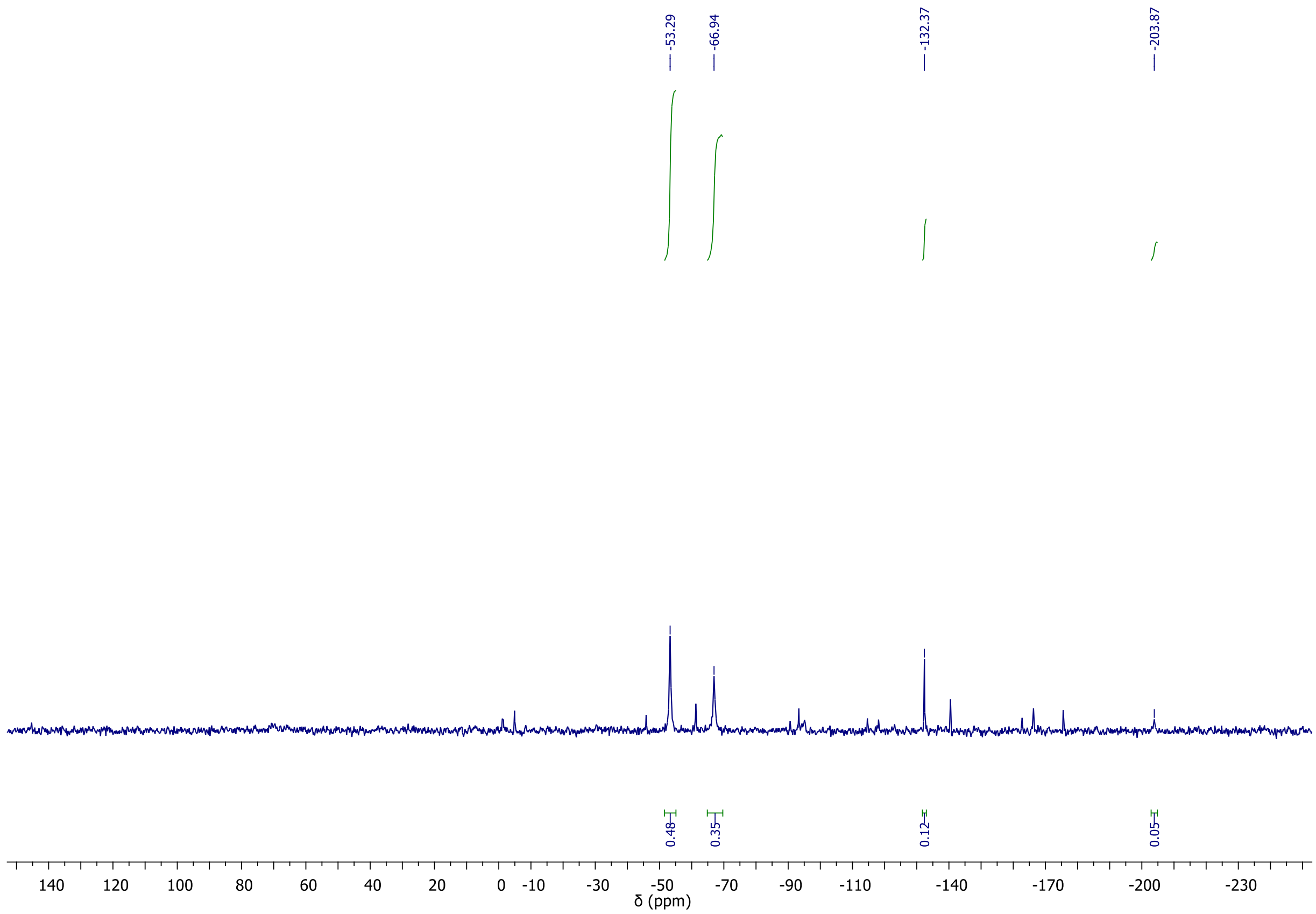

4.4. Reaction of **DipTerPAlCp*** with DDC

DipTerPAlCp* (20.0 mg, 33.9 µmol, 1.00 eq.) was dissolved in C_6D_6 (0.4 mL) in a *J.-Young* NMR tube and N,N-bis(2,6-diisopropylphenyl)carbodiimide (DDC) (12.3 mg, 33.9 µmol, 1.00 eq.) was added at ambient temperature. $^{31}P\{^1H\}$ NMR spectroscopy revealed a very slow, unselective reaction between **DipTerPAlCp*** and DDC. Heating the reaction mixture to 80 °C for several days or irradiating it with a Hg/Xe UV lamp (I = 19 A, U = 26 V, P = 500 W, λ = 210–600 nm) at ambient temperature did not accelerate the reaction. Heating to 105 °C for several days resulted in an unselective and incomplete reaction mixture.

Figure S54: $^{31}P\{^1H\}$ NMR reaction spectra of **DipTerPAlCp*** with DDC (162.1 MHz, C_6D_6, 298 K).

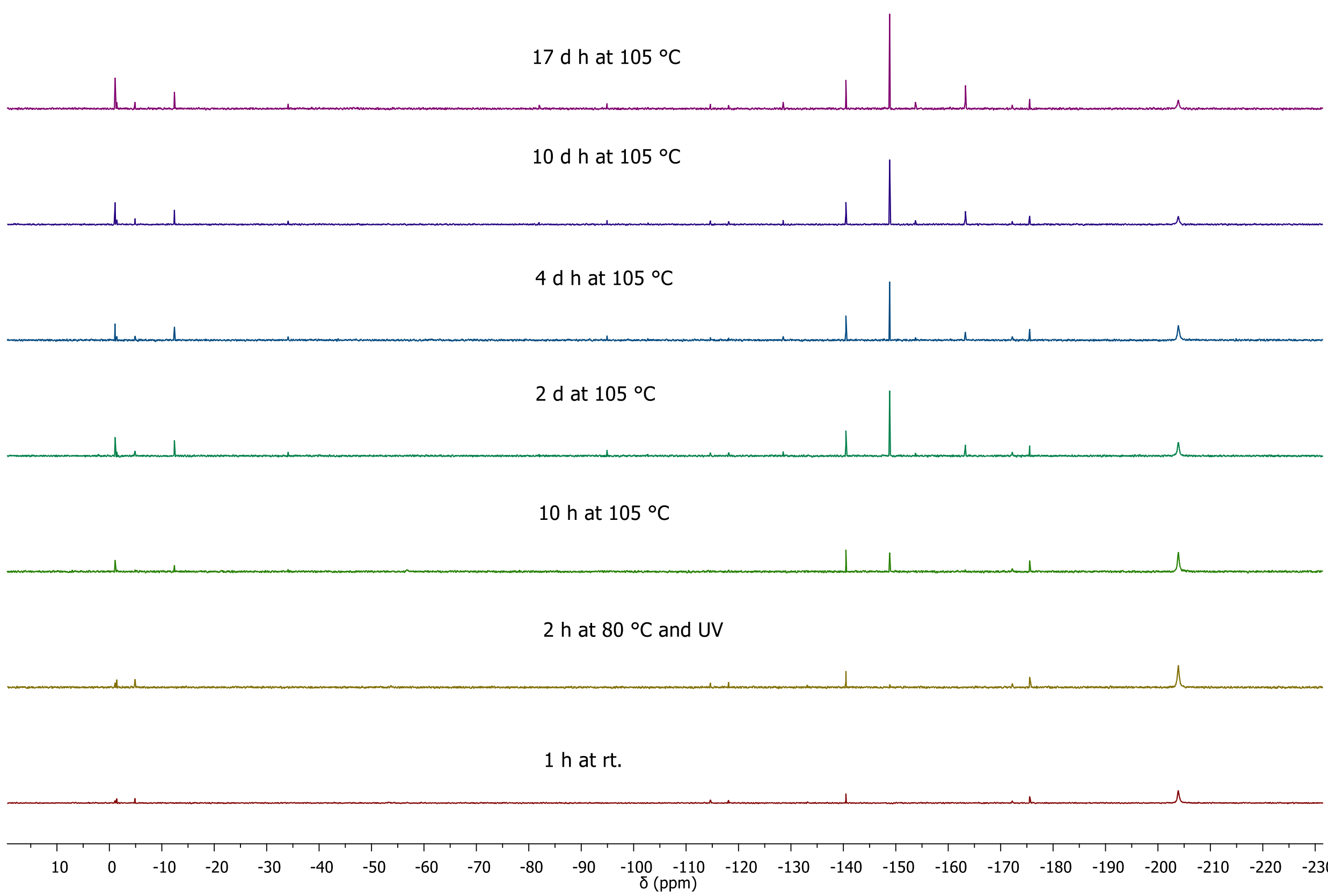

5. Computational details

General remarks

Computations were carried out using Gaussian16,[10] and the standalone version of NBO 6.0[11] in conjunction with Gaussian09.[12]

Structure optimizations employed the hybrid DFT functional PBE0[13] in conjunction with Grimme's dispersion correction D3(BJ)[14] and the def2-TZVP basis set[15] (notation e.g. PBE0-D3/def2-TZVP). All structures were fully optimized and confirmed as minima by frequency analyses.

Chemical shifts were derived by the GIAO method.[16] The calculated absolute shifts ($\sigma_{calc,X}$) were referenced to the experimental absolute shift of 85 % H_3PO_4 in the gas phase ($\sigma_{ref,1}$ = 328.35 ppm),[17] using PH_3 ($\sigma_{ref,2}$ = 594.45 ppm) as a secondary standard:

$$\delta_{calc,X} = (\sigma_{ref,1} - \sigma_{ref,2}) - (\sigma_{calc,X} - \sigma_{calc,PH3})$$

$$= \sigma_{calc,PH3} - \sigma_{calc,X} - 266.1 \text{ ppm}$$

At the PBE0-D3/def2-TZVP level of theory, $\sigma_{calc,PH3}$ amounts to +567.77 ppm.

Molecular orbitals were plotted using Avogadro the formatted checkpoint files from the optimizations at the PBE0-D3/def2-TZVP level of theory.

Optimized Structures. In addition to the electronic supporting information we provide a multi-structure xyz-file including all calculated molecules. For a better understanding and a more intuitive view of the calculated 3D structures, we strongly recommend using this file e.g. with the free program MERCURY.

Please note that most of the computations were carried out for single, isolated molecules in the gas phase (ideal gas approximation). There may well be significant differences between gas phase and condensed phase.

5.1. Summary of calculated data

Table S5. Summary of calculated data.

Compd.	PG	N_{imag}	E_{tot}[a]	U_0[a]	U_{298}[a]	H_{298}[a]	G_{298}[a]
**1**	C_1	0	–2137.9762	–2137.1615	–2137.1140	–2137.1130	–2137.2444
**2**	C_1	0	–1619.0634	–1618.4640	–1618.4291	–1618.4282	–1618.5334
**3**	C_1	0	–3757.1032	–3755.6836	–3755.6019	–3755.6010	–3755.7996
**TS_3**	C_1	1	–3757.0615	–3755.6444	–3755.5623	–3755,5613	–3755,7612
**4**	C_1	0	–3038.1969	–3036.8547	–3036.7830	–3036.7820	–3036.9606
**5**	C_1	0	–2906.8171	–2905.5849	–2905.5159	–2905.5150	–2905.6861
**6**	C_1	0	–2906.8191	–2905.5873	–2905.5183	–2905.5174	–2905.6914

[a] energy in a.u.

Table S6: Calculated absolute shifts ($\sigma_{calc,X}$) as obtained from a GIAO calculation at the PBE0-D3/def2TZVP level of theory in the gas phase and chemical shift using 85% H_3PO_4 and PH_3 as standards.

	$\sigma_{calc,X}$	$\delta_{calc.}$(^{31}P)	$\delta_{exp.}$(^{31}P)
**2**	503.5	−201.8	−201.5
**3**	324.3	−22.7	−27.0
**4**	278.6	23.1	20.6
**5**	354.9	−53.2	−56.1
**6**	321.2	−19.5	−14.3

5.2. NBO analyses

NBO analyses were carried out using the structures optimized at the PBE0-D3/def2-TZVP level of theory. The densities were obtained at the PBE0-D3/def2-TZVP level of theory. In the following the results of the NBO analyses are discussed in detail for compounds 2, 3, 4, 5 and 6. Atom numbers are denoted as "(X)", while natural charges are in bold and the WBIs are in italic numbers.

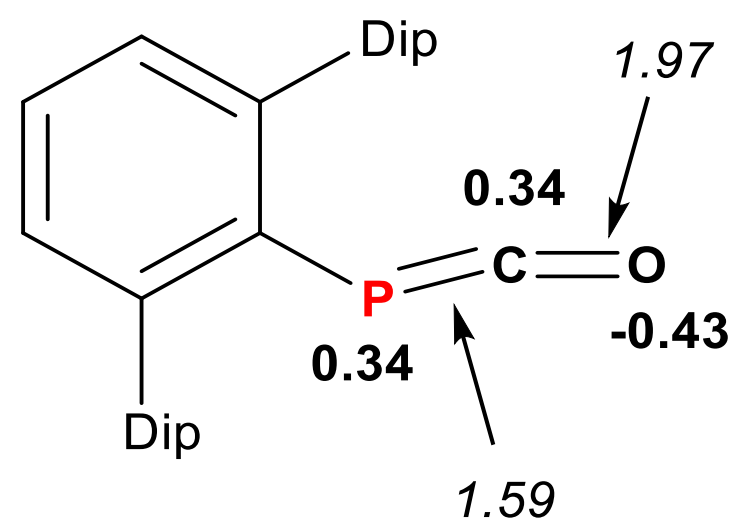

Aryl phosphaketene **2** shows positive natural charges for the P (0.34 e) and C (0.34 e) atoms of the PCO-unit, while the O (−0.43 e) atom carries a negative charge, indicating an overall positive charge on the PCO unit (0.25 e). Wiberg bond analysis shows a diminished WBI for the formal P=C double bond (1.59) with a C=O bond (1.98). Within the PCO-unit the P=C bond shows a σ- and π-component. While the P−C σ-bond is polarized towards the C atom (C: 66%; P: 34%) with the C atom being sp-hybridized and mainly p-orbital character at the P atom. The π-component is inversely polarized (C: 36%; P: 64%) and formed by p-orbitals on C and P. The σ- and π-components of the C=O bond are both polarized towards the O atom (C: ≈ 30 %; O: ≈ 70 %). The diminished WBI of the P=C bond stems from back donation of a p-type lone pair of electrons on the O atom into the π*-orbital of the P=C-unit (E(2) = 128.8 kcal·mol^{-1}). Additionally, the lone pair of electrons on the P atom minimally donates into the C=O π*-orbital (E(2) = 23.7 kcal·mol^{-1}).

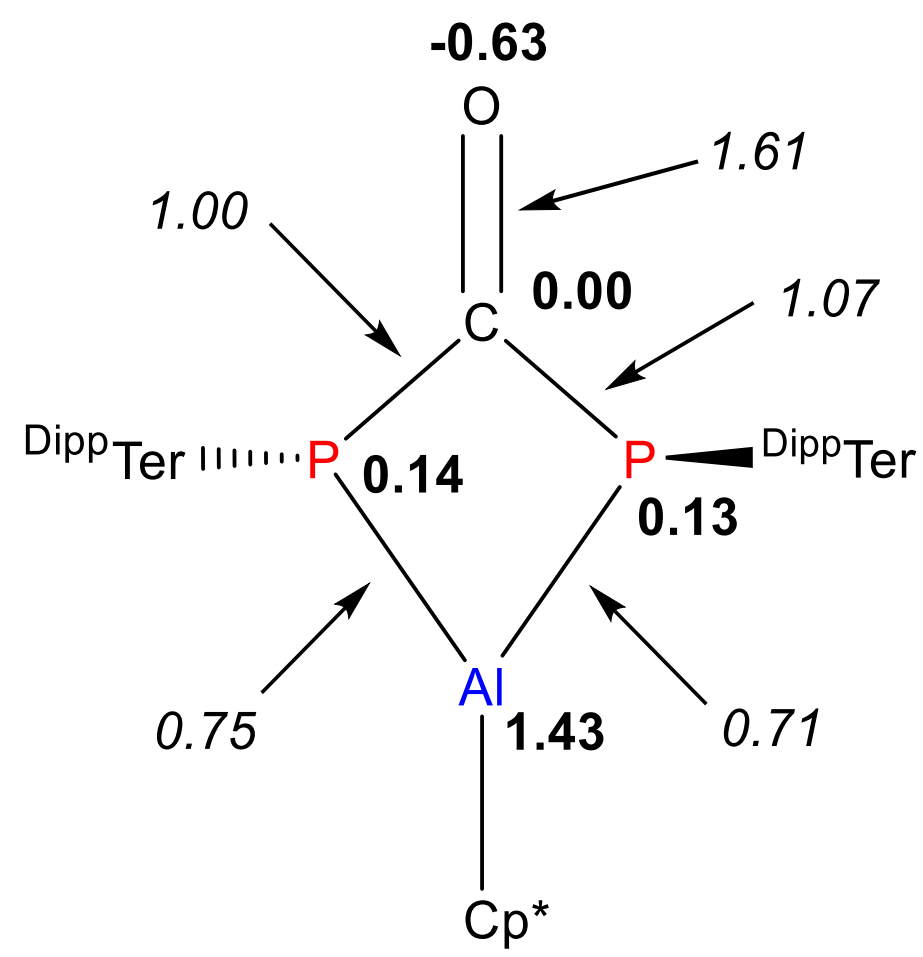

Diphosphaureylene aluminium species **3** shows alternating natural charges in the P2AlC ring (P: 0.14 e, 0.13 e; C: 0.00; Al: 1.43 e) according to NPA. The Al−P bonds have WBIs (0.71, 0.75) indicative of polarized covalent single bond, while the WBIs (1.07, 1.00) of the P−C bonds are close to unity and indicate less polarization. The exocyclic C=O bond can be described as double bond, supported by a WBI of 1.61. The P atoms each feature an sp-type lone pair of electrons, while the oxygen atom of the diphosphaureylene unit shows two LPs, one with sp-character, while the other resides in a pure p-orbital. As suggested by the WBIs the P−Al bonds (P1: 79%, Al: 21%; P2: 76%, Al: 24%) are strongly polarized towards the P atoms, respectively. By contrast the P−C bonds (P1: 60%,C: 40%; P2: 59%, Al: 41%) are only minimally polarized towards the C atom, respectively.

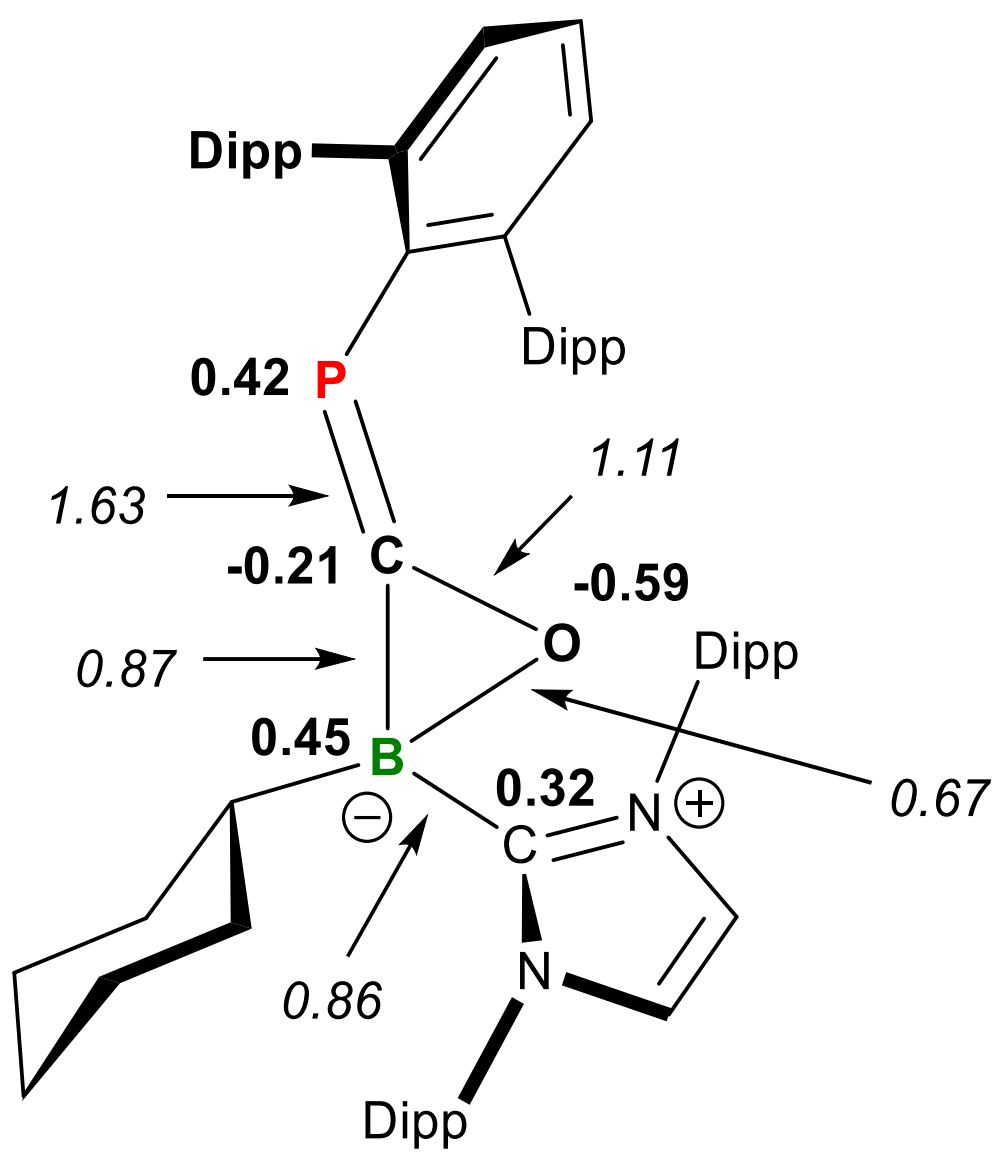

Phosphaalkene-appended oxaborirane **4** shows a strongly polarized BCO-ring with a positive charge on boron (0.45e) and negative charges on C (−0.21e) and O (−0.59e), while the P atom of the phosphaalkene unit bears a positive charge (0.42e). The P=C bond can be classified as double bond (WBI: 1.63), while the three bonds in the oxaborirane unit show WBIs close to unity (B−O: 0.67; O−C: 1.11; C−B: 0.87). The B−C_{NHC} bond has a WBI of 0.86 with a positive charge on the C_{NHC} atom (0.32e), indicating a charge transfer from the NHC to the oxaborirane unit. This is further supported by a net electron transfer of 0.54e (overall charge of the NHC unit: +0.54e). As expected, the O atom in the BCO-ring carries to LPs one in an sp hybrid orbital the other in a pure p-orbital. This p-type LP is significantly delocalized into the π*-orbital of the P=C unit with a significant stabilization energy (E(2) = 48.6 kcal·mol^{-1}). This is in line with a bond order <2 for the P=C bond of which the σ-component (P: 35%, C: 65%) is polarized towards the C atom, while the π-component (P: 58%, C: 42%) is polarized towards the P atom. The bonds within the three-membered ring O−B (O: 79%, O: 21%), B−C (B: 36%, C: 64%) and C−O (C: 33%, O: 67%) are significantly polarized and are in line with polar covalent bonds.

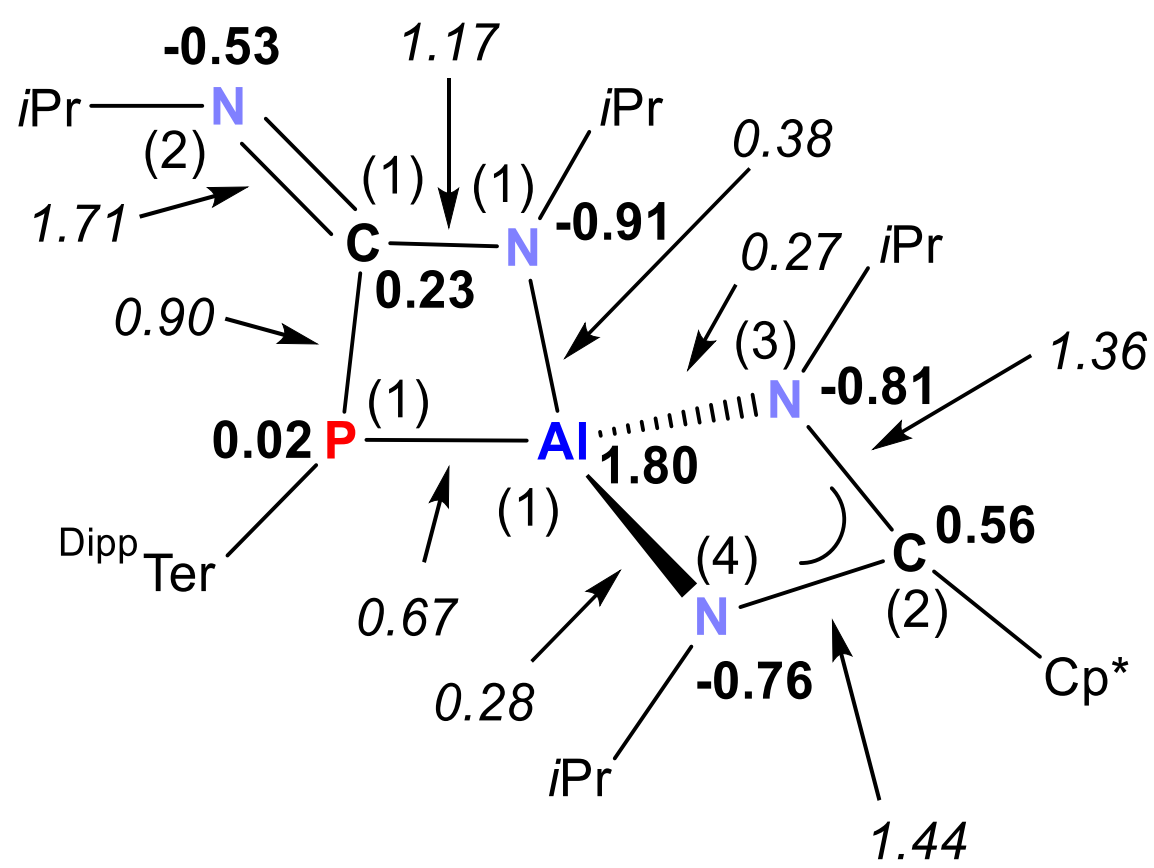

Aluminium species **5** with a κ*N,P*-phosphaguanidinate and amidinate ligand on the tetracoordinate aluminium center, which carries a significant positive charge (1.80 e), in line with an oxidation state of +III. The amidinate unit carries an overall charge of −0.58 e, while the phosphaguanidate unit is more negatively charged at −1.22 e. The P–Al bond (P: 77%, Al: 23%) is strongly polarized towards the P atom implying a high ionic character in the bond, which is further supported by a WBI of 0.67. The Al–N1 bond has a WBI of 0.38, which is in line with related 1,3-diaza-2-phospha-4-alumetidines, obtained in the reaction of ^{Dipp}TerPAlCp* with two equivalents of azides, which are best described as amino-amido Al(III) complexes. The Al-N1 bond is strongly polar and mainly localized at the N atom and best described by delocalization of this LP into the lone valencies at the Al atom with an overall stabilization energy of 82.2 kcal·mol^{-1}. The Wiberg bond indices in the N2–C1–N1 unit are in line with localized single and double bonds. The coordination of the amidinate unit to aluminium can be classified as ionic, with significantly diminished Al1–N3 (0.27) and Al1–N4 (0.28) bond orders and no bond orbitals are found. The Al1–N3 and Al1–N4 bonds are again best described as donation from sp^2-type LPs on the nitrogen atoms into lone valencies on aluminium.

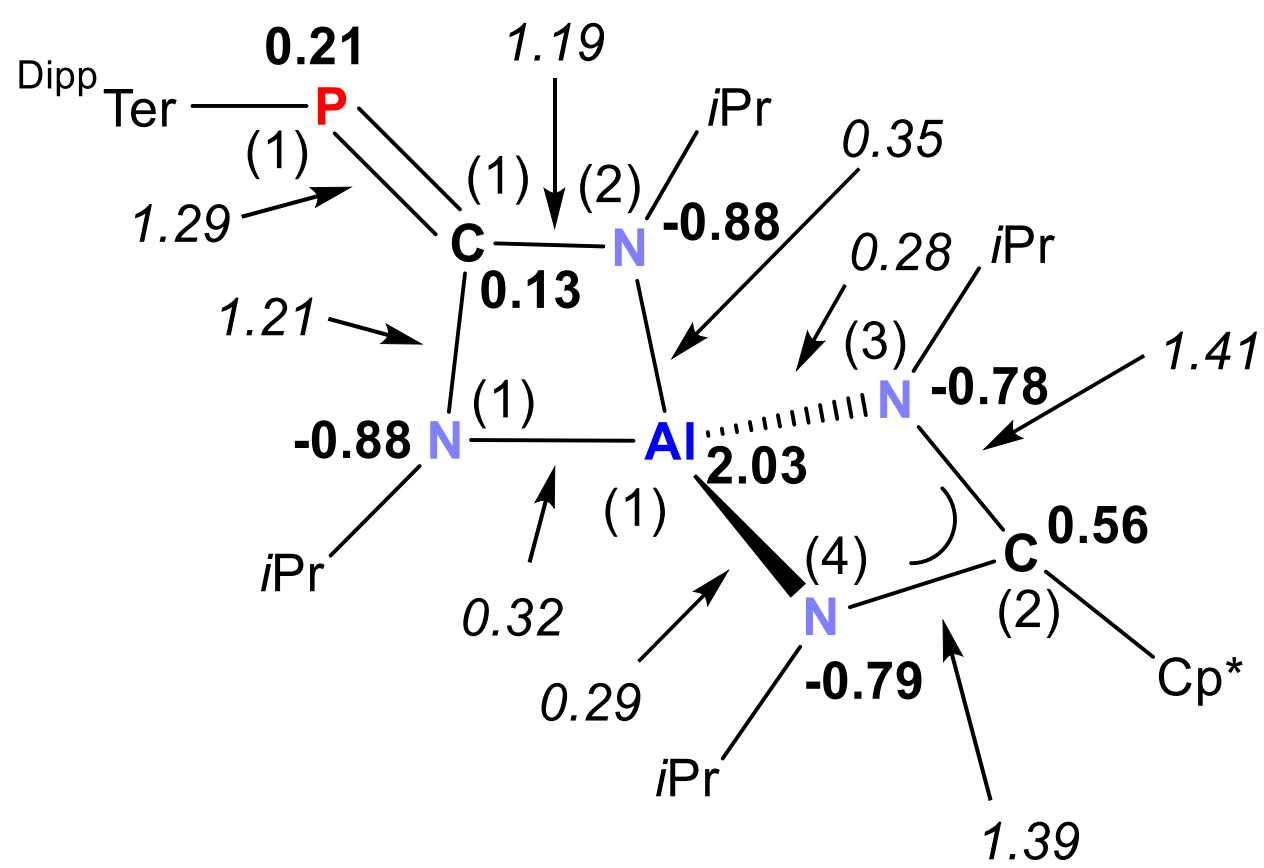

Following thermal rearrangement κ*N,N*-phosphaguanidinate species **6** is formed. The coordination of the amidinate unit is nearly unchanged with ionic Al–N interactions and a charge transfer of 0.43 e from the amidinate unit to Al is indicated by an overall charge of the $[(i\text{PrN})_2\text{CCp*}]^-$ unit of −0.57 e. Considering this, the overall charge of the phosphaguanidinate unit is −1.46 e. The phosphaguanidinate unit changes from κ*P,N* to a κ*N,N* coordination mode, which results in an exocyclic P1=C1 double bond (WBI: 1.29), in which the π-component is significantly polarized towards the P atom. The diminished bond order is a result from delocalization of the p-type LP on the N1 and N2 atoms into the P1–C1 π*-orbital with significant overall stabilization energies of ca. 127 $\text{kcal}\cdot\text{mol}^{-1}$. This is in line with the classification as an inversely polarized phosphaalkene. The Al–N1 and Al–N2 interactions are again formed by sp^2 type lone pairs of electrons on N1 and N2 donating into lone valencies at the Al atom. For each N atom overall stabilization energies of ca. 100 $\text{kcal}\cdot\text{mol}^{-1}$ were determined by 2nd order perturbation theory analysis. The same result is obtained for the Al–N3 and Al–N4 interactions.

NRT Analyses of model compounds **4’** and **6’**.

Model compound **4’** was generated from the optimized structure of **4** at the PBE0-D3/def2-TZVP level of theory by replacing the [Dipp]Ter- and Cyclohexyl-moieties with a methyl group. Moreover, the Dipp-groups of IPr were replaced with methyl groups. In the following the results from the NRT analysis are shown:

Leading resonance structure:

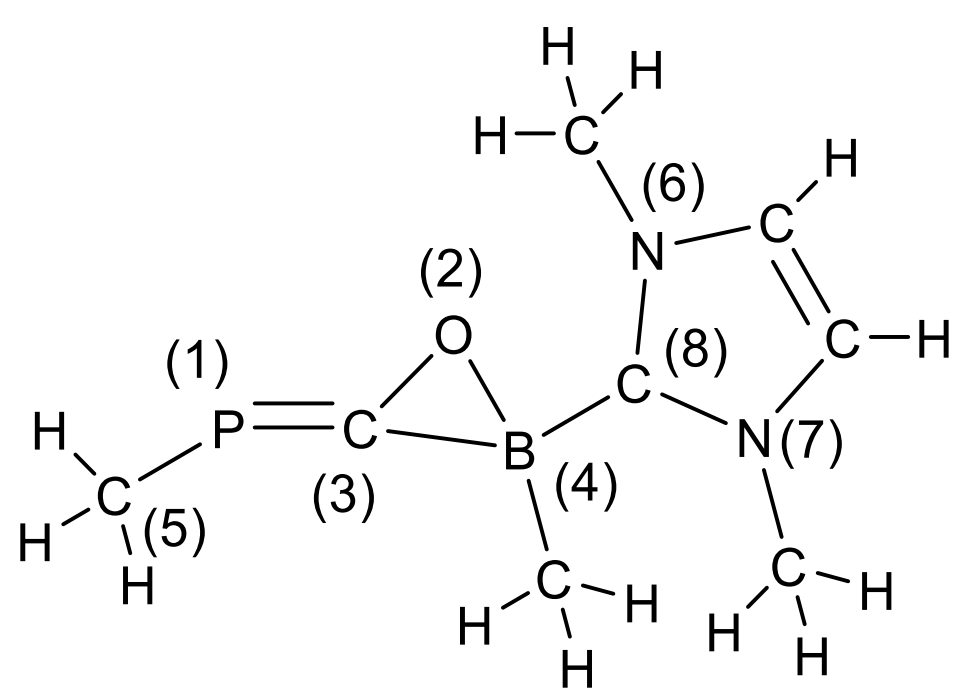


```
     Resonance
  RS   Weight(%)              Added(Removed)
-----------------------------------------------------------------------------
   1*    18.65
   2*    18.56   N  6- C  8, ( N  7- C  8), ( N  6),  N  7
   3     5.28  ( P  1- C  3),  O  2- C  3,  N  6- C  8, ( N  7- C  8),
                 P  1, ( O  2), ( N  6),  N  7
   4     5.26  ( P  1- C  3),  O  2- C  3,  P  1, ( O  2)
   5     4.40   N  6- C  8, ( N  7- C  8),  N  7- C 11, ( C  9- C 11),
                ( N  6),  C  9
   6     4.40   N  6- C  9, ( C  9- C 11), ( N  6),  C 11
```

Model compound **6'** was generated from the optimized structure of **6** at the PBE0-D3/def2-TZVP level of theory by replacing the ^{Dipp}Ter-, *i*Pr- and Cp*-moieties with a methyl group. In the following the results from the NRT analysis are shown:

Leading resonance structure (1) with atom numbering scheme in parentheses:

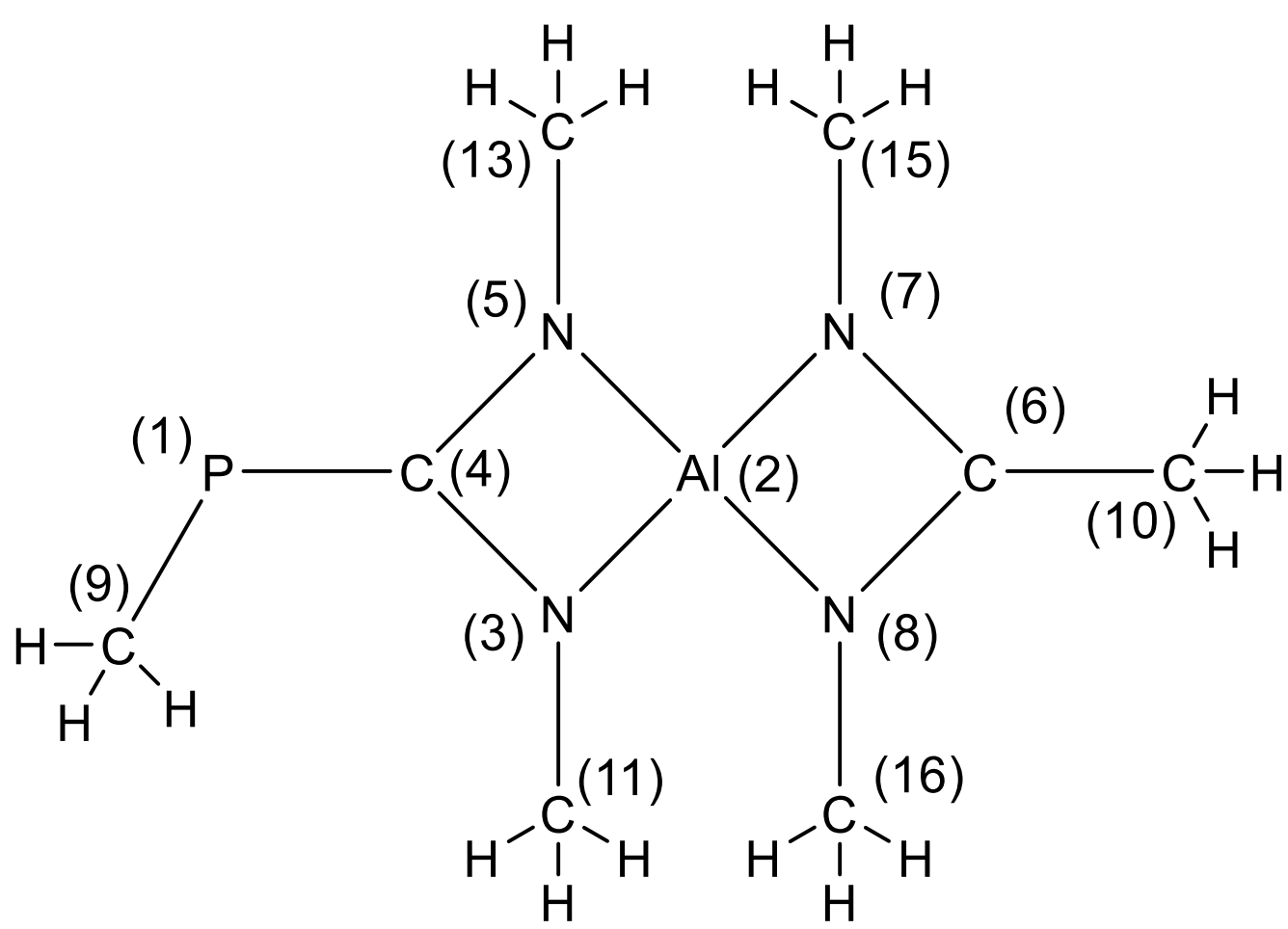

Resonance

```
RS   Weight(%)          Added(Removed)
---------------------------------------------------------------------------
  1*   14.39
  2*   13.49  ( C  6- N  7),  C  6- N  8,  N  7, ( N  8)
  3 (2)  5.90  ( P  1- C  4),  C  4- N  5, ( C  6- N  7),  C  6- N  8,
               P  1, ( N  5),  N  7, ( N  8)
  4 (2)  5.50  ( P  1- C  4),  C  4- N  5,  P  1, ( N  5)
  5 (2)  4.35  ( P  1- C  4),  N  3- C  4, ( C  6- N  7),  C  6- N  8,
               P  1, ( N  3),  N  7, ( N  8)
  6 (2)  4.10  ( P  1- C  4),  N  3- C  4,  P  1, ( N  3)
```

5.3. Kohn-Sham Orbitals

In the following selected frontier molecular orbitals at the PBE0-D3/def2-TZVP level of theory for **2**-**6** are shown.

Figure S55: Selected Kohn-Sham orbitals of **2** at the PBE0-D3/def2-TZVP level of theory.

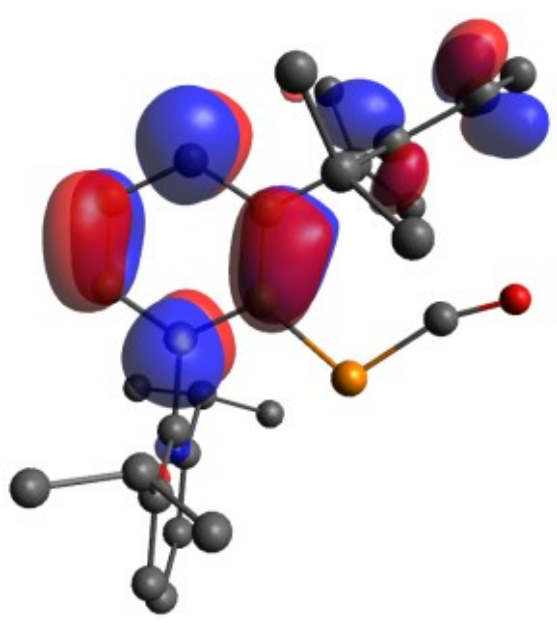

LUMO+2

−0.58 eV

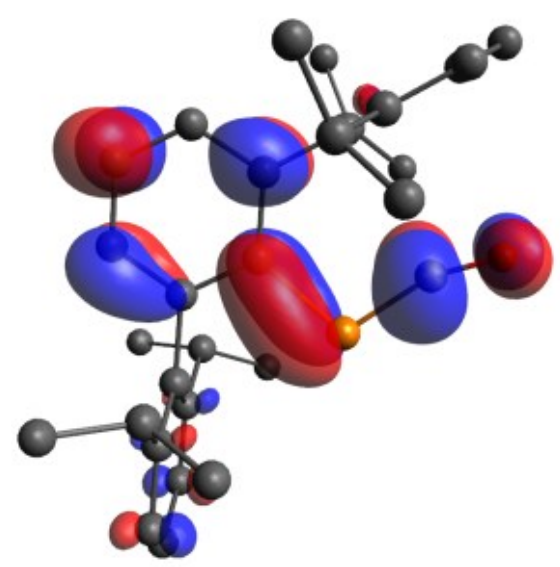

LUMO+1

−1.11 eV

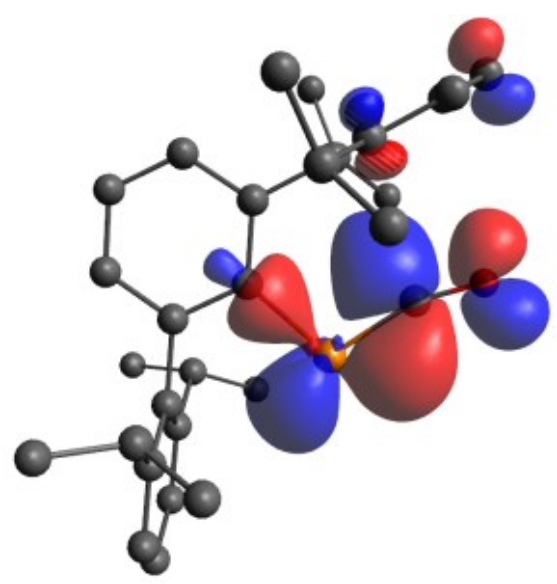

LUMO

−1.63 eV

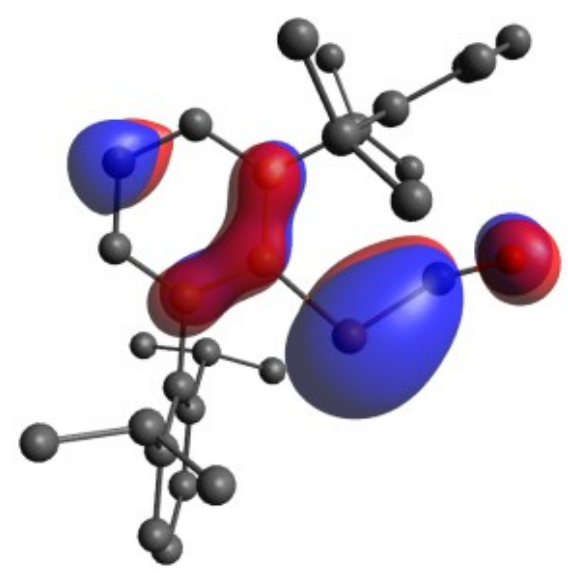

HOMO

−5.94 eV

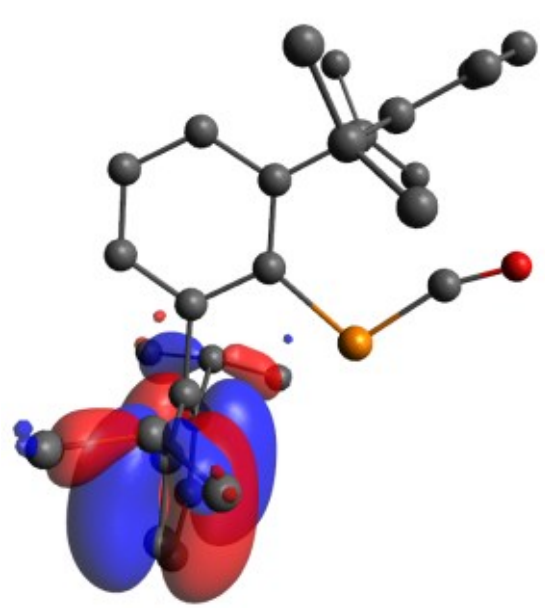

HOMO-1

−6.83 eV

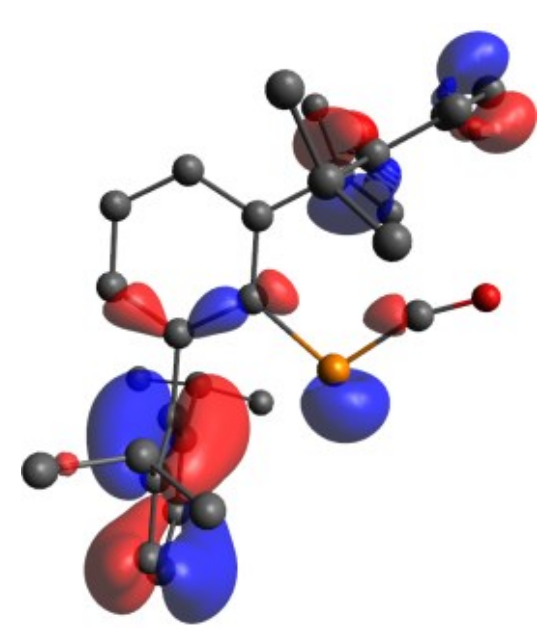

HOMO-2

−6.87 eV

Figure S56: Selected Kohn-Sham orbitals of **3** at the PBE0-D3/def2-TZVP level of theory.

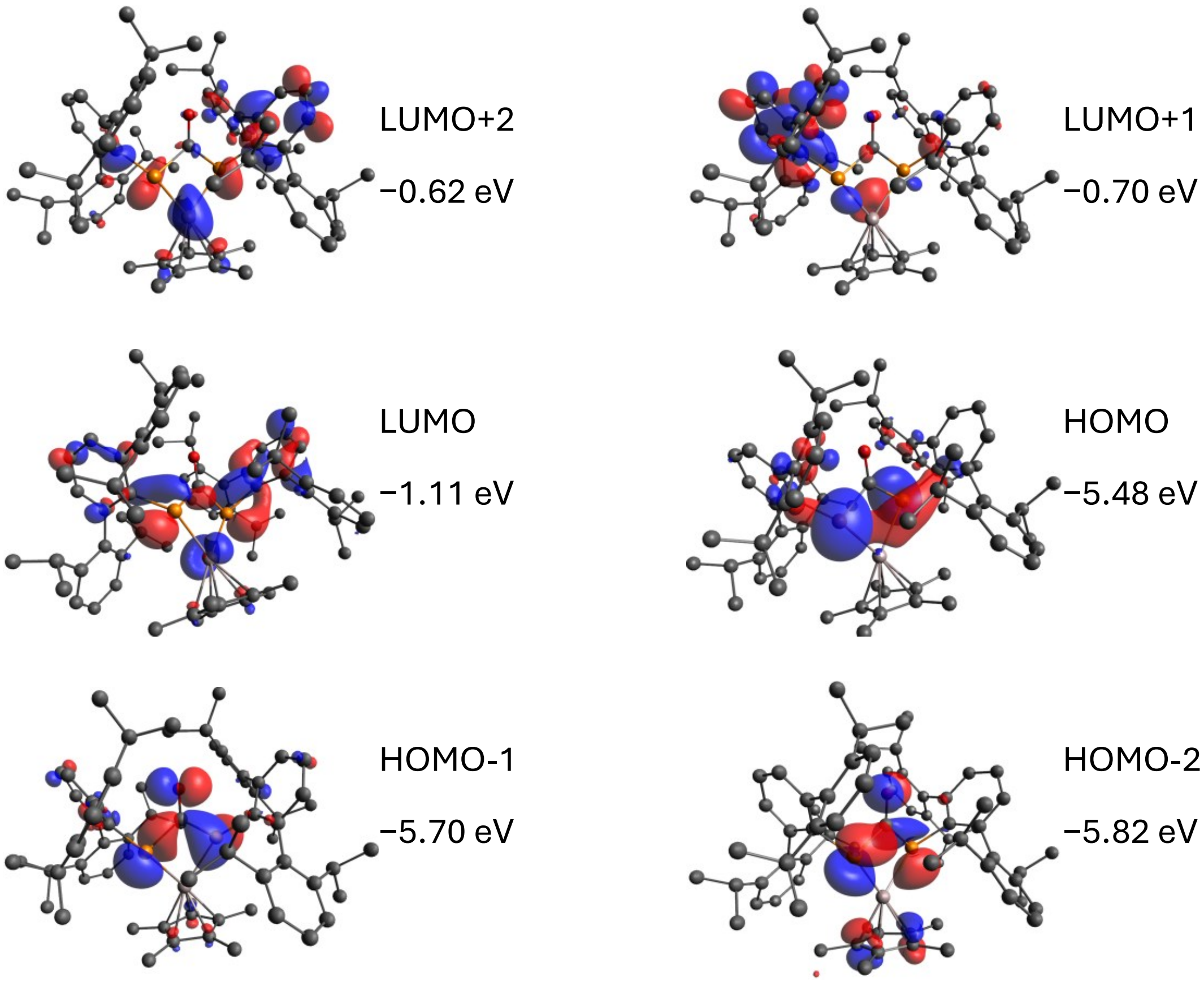

Figure S57: Selected Kohn-Sham orbitals of **4** at the PBE0-D3/def2-TZVP level of theory.

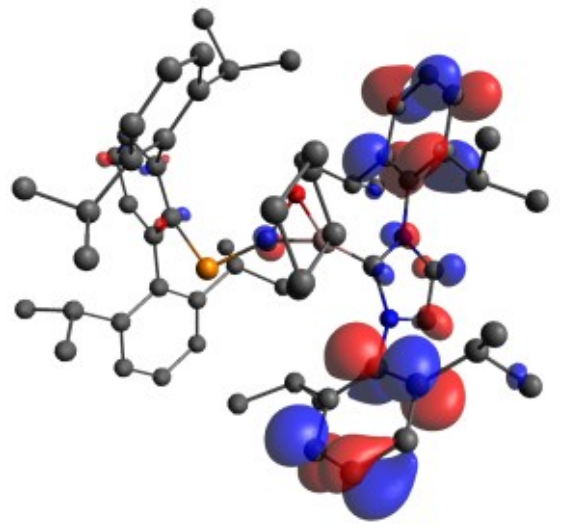

LUMO+2

−0.59 eV

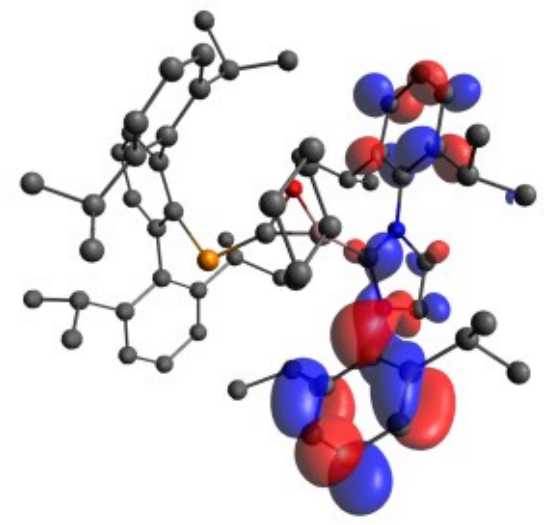

LUMO+1

−0.65 eV

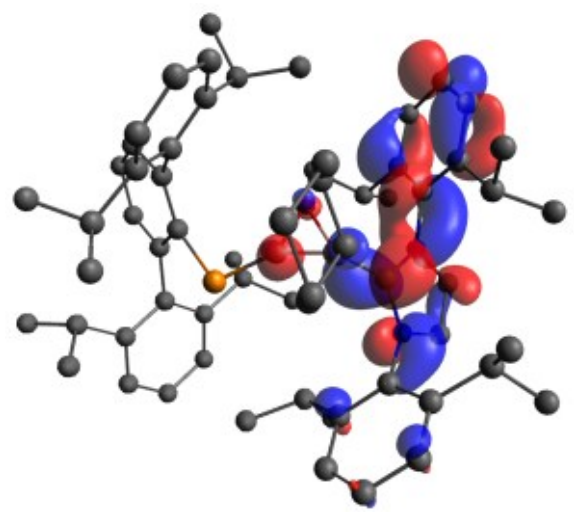

LUMO

−0.86 eV

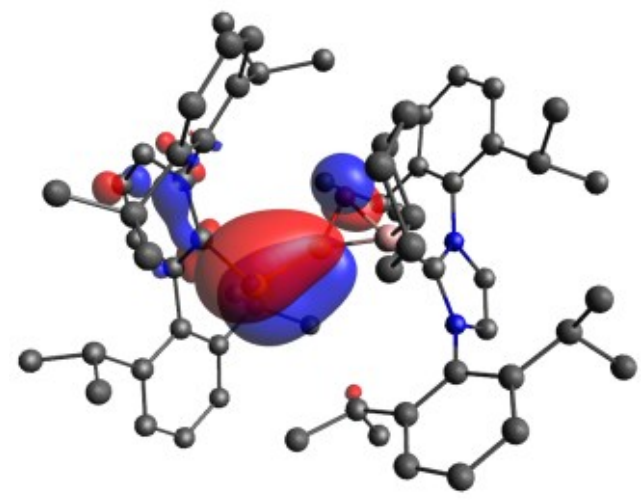

HOMO

−5.20 eV

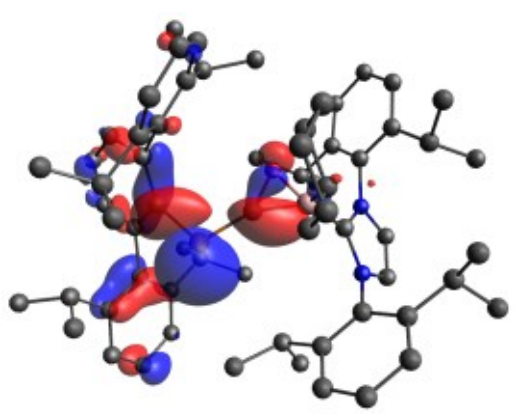

HOMO-3

−6.14 eV

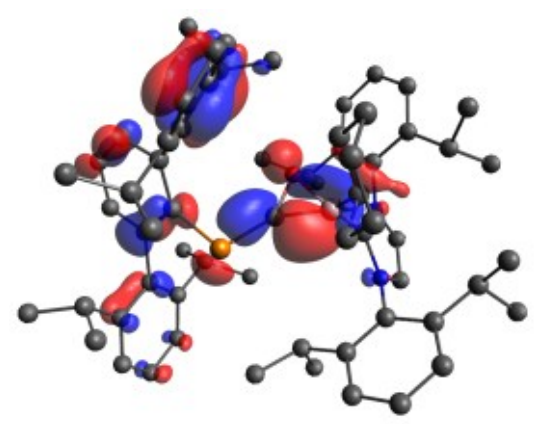

HOMO-4

−6.37 eV

Figure S58: Selected Kohn-Sham orbitals of **5** at the PBE0-D3/def2-TZVP level of theory.

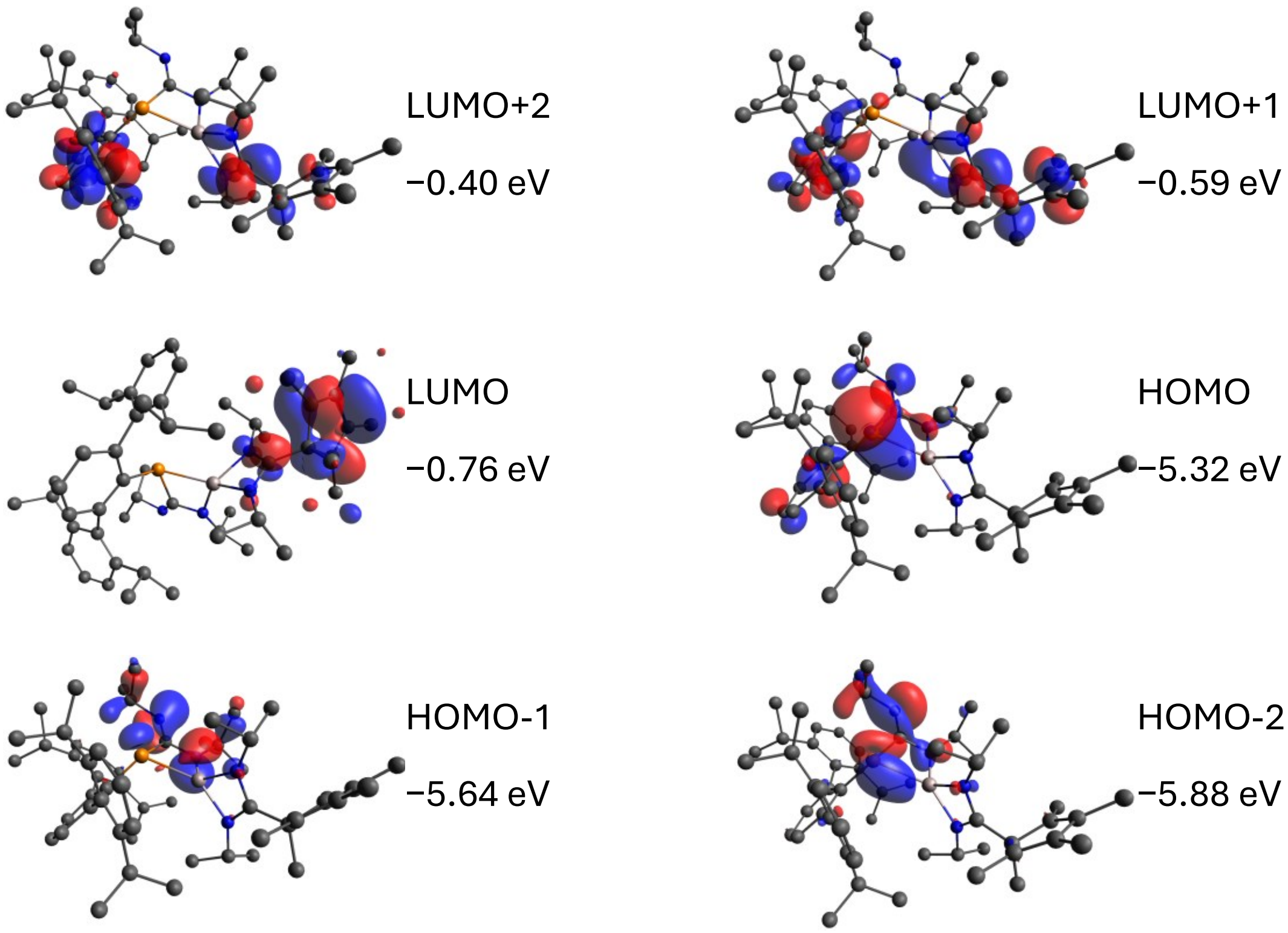

Figure S59: Selected Kohn-Sham orbitals of **6** at the PBE0-D3/def2-TZVP level of theory.

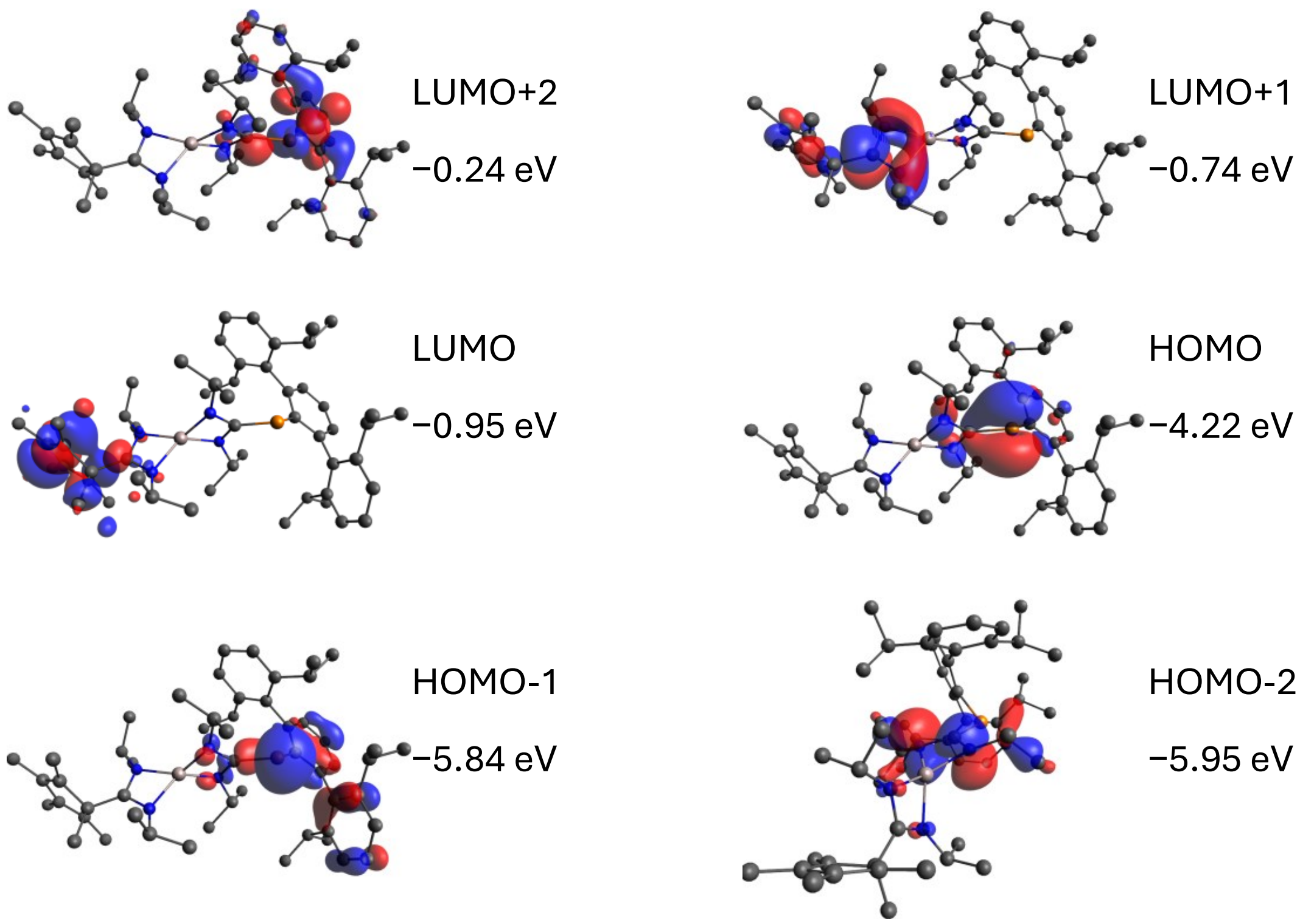

5.3. Thermodynamic Considerations

The Gibbs free energy for the formation of 3 from a formal [2+2]-cycloaddition between 1 and 2 was estimated in the gas phase at the PBE0-D3/def2-TZVP level of theory. The reaction path was investigated using a combination of relaxed potential energy surface scans, as well as transition state searches using the eigenvector-following method.[18] This clearly showed that the formation of **3** is exergonic ($\Delta G° = -57.3\ kJ \cdot mol^{-1}$), with a transition state that shows a close contact between the P atom in **1** and the C atom of the PCO-unit in **2**. In the gas phase the activation energy is 43 $kJ \cdot mol^{-1}$, in line with a facile transformation at room temperature.

Figure S60: Calculated reaction profile of the formation of **3** at the PBE0-D3/def2-TZVP level of theory, with the gas-phase-structure of the transition state shown in the top right corner.

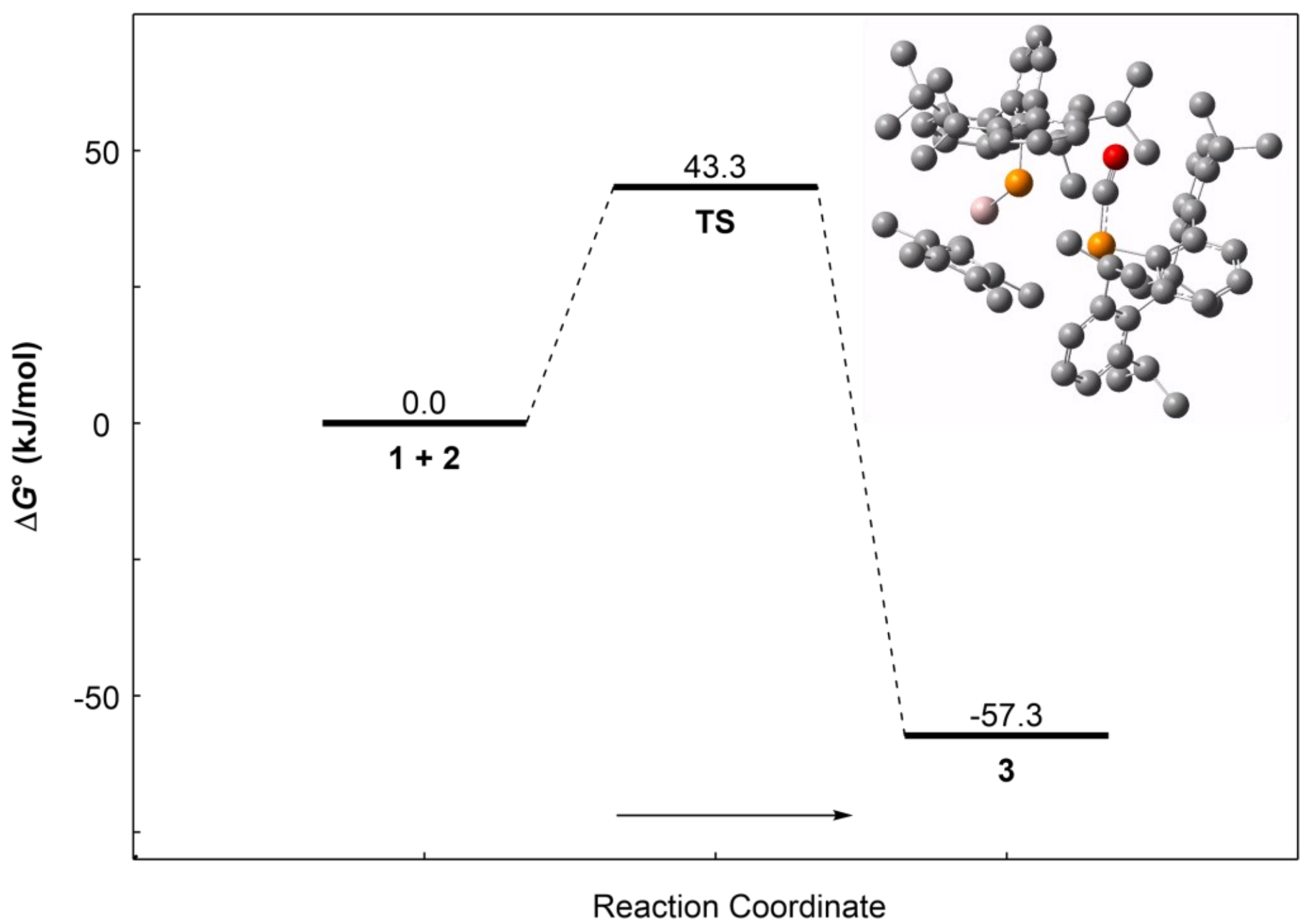

Furthermore, the rearrangement of **5** to **6** was evaluated. While no transition state could be found for this transformation, the reaction was found to be slightly exergonic ($\Delta_R G° = -13.9\ kJ \cdot mol^{-1}$). This suggests a rather high activation barrier, as the reaction proceeds slowly at 80°°C.

6. References

1 a) M. Fischer, S. Nees, T. Kupfer, J. T. Goettel, H. Braunschweig, C. Hering-Junghans, *J. Am. Chem. Soc.* **2021**, *143*, 4106–4111; b) S. Nees, T. Wellnitz, F. Dankert, M. Härterich, S. Dotzauer, M. Feldt, H. Braunschweig, C. Hering-Junghans, *Angew. Chem. Int. Ed.* **2023**, *62*, e202215838.

2 Y. Konrad, M. Arrowsmith and H. Braunschweig, *ChemRxiv* **2025***,* DOI: 10.26434/chemrxiv-2025-l9qnb.

3 G. R. Fulmer, A. J. M. Miller, N. H. Sherden, H. E. Gottlieb, A. Nudelman, B. M. Stoltz, J. E. Bercaw, K. I. Goldberg, *Organometallics* **2010**, *29*, 2176–2179

4 G. Sheldrick, *Acta Crystallogr., Sect. A* **2015**, *71*, 3–8.

5 G. Sheldrick, *Acta Crystallogr., Sect. A* **2008**, *64*, 112–122.

6 R. A. Bonham, L. S. Bartell, D. A. Kohl, *J. Am. Chem. Soc.* **1959**, *81*, 4765–4769.

7 a) D. Kratzert, I. Krossing, *J. Appl. Cryst.* **2018**, *51*, 928–934; b) D. Kratzert, J. J. Holstein, I. Krossing, *J. Appl. Cryst.* **2015**, *48*, 933–938.

8 T. Taeufer, F. Dankert, D. Michalik, J. Pospech, J. Bresien, C. Hering-Junghans, *Chem. Sci.* **2023**, *14*, 3018–3023.

9 B. Twamley, C. D. Sofield, M. M. Olmstead, P. P. Power, *J. Am. Chem. Soc.* **1999**, *121*, 3357–3367.

10 Gaussian 16, Revision C.01, M. J. Frisch, G. W. Trucks, H. B. Schlegel, G. E. Scuseria, M. A. Robb, J. R. Cheeseman, G. Scalmani, V. Barone, G. A. Petersson, H. Nakatsuji, X. Li, M. Caricato, A. V. Marenich, J. Bloino, B. G. Janesko, R. Gomperts, B. Mennucci, H. P. Hratchian, J. V. Ortiz, A. F. Izmaylov, J. L. Sonnenberg, D. Williams-Young, F. Ding, F. Lipparini, F. Egidi, J. Goings, B. Peng, A. Petrone, T. Henderson, D. Ranasinghe, V. G. Zakrzewski, J. Gao, N. Rega, G. Zheng, W. Liang, M. Hada, M. Ehara, K. Toyota, R. Fukuda, J. Hasegawa, M. Ishida, T. Nakajima, Y. Honda, O. Kitao, H. Nakai, T. Vreven, K. Throssell, J. A. Montgomery, Jr., J. E. Peralta, F. Ogliaro, M. J.

Bearpark, J. J. Heyd, E. N. Brothers, K. N. Kudin, V. N. Staroverov, T. A. Keith, R. Kobayashi, J. Normand, K. Raghavachari, A. P. Rendell, J. C. Burant, S. S. Iyengar, J. Tomasi, M. Cossi, J. M. Millam, M. Klene, C. Adamo, R. Cammi, J. W. Ochterski, R. L. Martin, K. Morokuma, O. Farkas, J. B. Foresman, and D. J. Fox, Gaussian, Inc., Wallingford CT, **2016**.

11 a) E. D. Glendening, J. K. Badenhoop, A. E. Reed, J. E. Carpenter, J. A. Bohmann, C. M. Morales, C. R. Landis, F. Weinhold, *NBO 6.0*, Theoretical Chemistry Institute, University of Wisconsin, Madison, **2013**; b) J. E. Carpenter, F. Weinhold, *J. Mol. Struct.: THEOCHEM* **1988**, *169*, 41–62; c) F. Weinhold, J. E. Carpenter, in *The Structure of Small Molecules and Ions* (Eds.: R. Naaman, Z. Vager), Springer, Boston, MA, **1988**, pp. 227–236; d) F. Weinhold, C. R. Landis, *Valency and Bonding. A Natural Bond Orbital Donor-Acceptor Perspective*, Cambridge University Press, **2005**.

12 Gaussian 09, Revision A.02, M. J. Frisch, G. W. Trucks, H. B. Schlegel, G. E. Scuseria, M. A. Robb, J. R. Cheeseman, G. Scalmani, V. Barone, G. A. Petersson, H. Nakatsuji, X. Li, M. Caricato, A. Marenich, J. Bloino, B. G. Janesko, R. Gomperts, B. Mennucci, H. P. Hratchian, J. V. Ortiz, A. F. Izmaylov, J. L. Sonnenberg, D. Williams-Young, F. Ding, F. Lipparini, F. Egidi, J. Goings, B. Peng, A. Petrone, T. Henderson, D. Ranasinghe, V. G. Zakrzewski, J. Gao, N. Rega, G. Zheng, W. Liang, M. Hada, M. Ehara, K. Toyota, R. Fukuda, J. Hasegawa, M. Ishida, T. Nakajima, Y. Honda, O. Kitao, H. Nakai, T. Vreven, K. Throssell, J. A. Montgomery, Jr., J. E. Peralta, F. Ogliaro, M. Bearpark, J. J. Heyd, E. Brothers, K. N. Kudin, V. N. Staroverov, T. Keith, R. Kobayashi, J. Normand, K. Raghavachari, A. Rendell, J. C. Burant, S. S. Iyengar, J. Tomasi, M. Cossi, J. M. Millam, M. Klene, C. Adamo, R. Cammi, J. W. Ochterski, R. L. Martin, K. Morokuma, O. Farkas, J. B. Foresman, and D. J. Fox, Gaussian, Inc., Wallingford CT, **2016**.

13 a) J. P. Perdew, K. Burke, M. Ernzerhof, *Phys. Rev. Lett.* **1996**, *77*, 3865–3868; b) J. P. Perdew, K. Burke, M. Ernzerhof, *Phys. Rev. Lett.* **1997**, *78*, 1396–1396; c) C. Adamo, V. Barone, *J. Chem. Phys.* **1999**, *110*, 6158–6170.

14 a) S. Grimme, J. Antony, S. Ehrlich, H. Krieg, *J. Chem. Phys.* **2010**, *132*, 154104; b) S. Grimme, S. Ehrlich, L. Goerigk, *J. Comput. Chem.* **2011**, *32*, 1456–1465.

15 F. Weigend, R. Ahlrichs, *Phys. Chem. Chem. Phys.* **2005**, *7*, 3297–305.

16 F. London, *Journal de Physique et le Radium* **1937**, *8*, 397–409; b) R. McWeeny, *Phys. Rev.* **1962**, *126*, 1028–1034; c) R. Ditchfield, *Mol. Phys.* **1974**, *27*, 789–807; d) K. Wolinski, J. F. Hinton, P. Pulay, *J. Am. Chem. Soc.* **1990**, *112*, 8251; e) J. R. Cheeseman, G. W. Trucks, T. a Keith, M. J. Frisch, *J. Chem. Phys.* **1996**, *104*, 5497–5509.

17 C. J. Jameson, A. De Dios, A. Keith Jameson, *Chem. Phys. Lett.* **1990**, *167*, 575–582.

18 (a) J. Baker, *J. Comput. Chem.* **1986**, *7*, 385–395; (b) H. B. Schlegel, In *Advances in Chemical Physics: Ab Initio Methods in Quantum Chemistry Part I, Volume 67*; Lawley, K. P., Ed.; John Wiley & Sons, **1987**; pp 249–286; (c) H. B. Schlegel, In *Modern Electronic Structure Theory*; Yarkony, D. R., Ed.; World Scientific, **1995**; pp 459–500; (d) F. Eckert, P. Pulay, H.-J. Werner, *J. Comput. Chem.* **1997**, *18* (12), 1473–1483.
